# Supplementary figures and images for: A CRISPR-based rapid DNA repositioning strategy and the early intranuclear life of HSV-1 (part 1 of 2)
Source: eLife. 2023 Sep 13;12:e85412. doi: 10.7554/eLife.85412 (PMC10522339; doi:10.7554/eLife.85412)

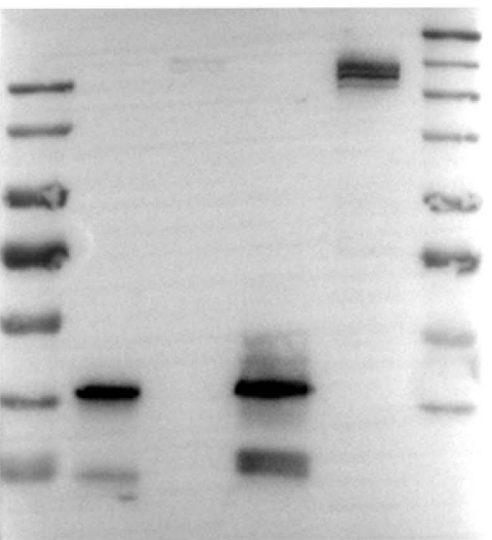

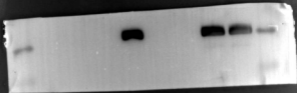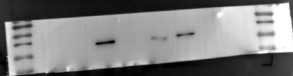

Supplement: Figure 1—source data 1. — This zip archive contains source data for western blot in panel B, original data collected for statistical analysis in panel F and H, and processed images (TIF files) in panel C, D, E and G. Original fluorescence images and confocal images in panel C, D, E, and G are deposited on DRYAD (https://doi.org/10.5061/dryad.vmcvdncxd). [file elife-85412-fig1-data1.pdf]

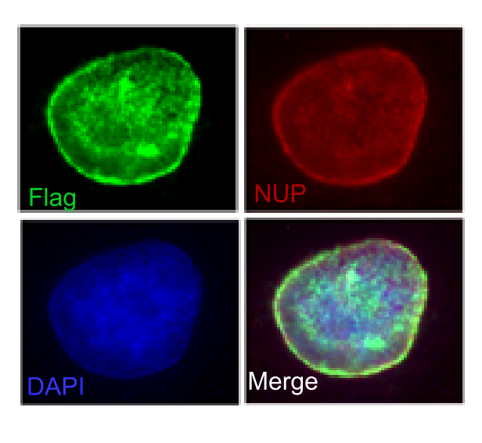

Supplement: Figure 1—source data 3. [file elife-85412-fig1-data3.zip › Fig 1- source data 3 Fig 1 C/fig1-c.tif]

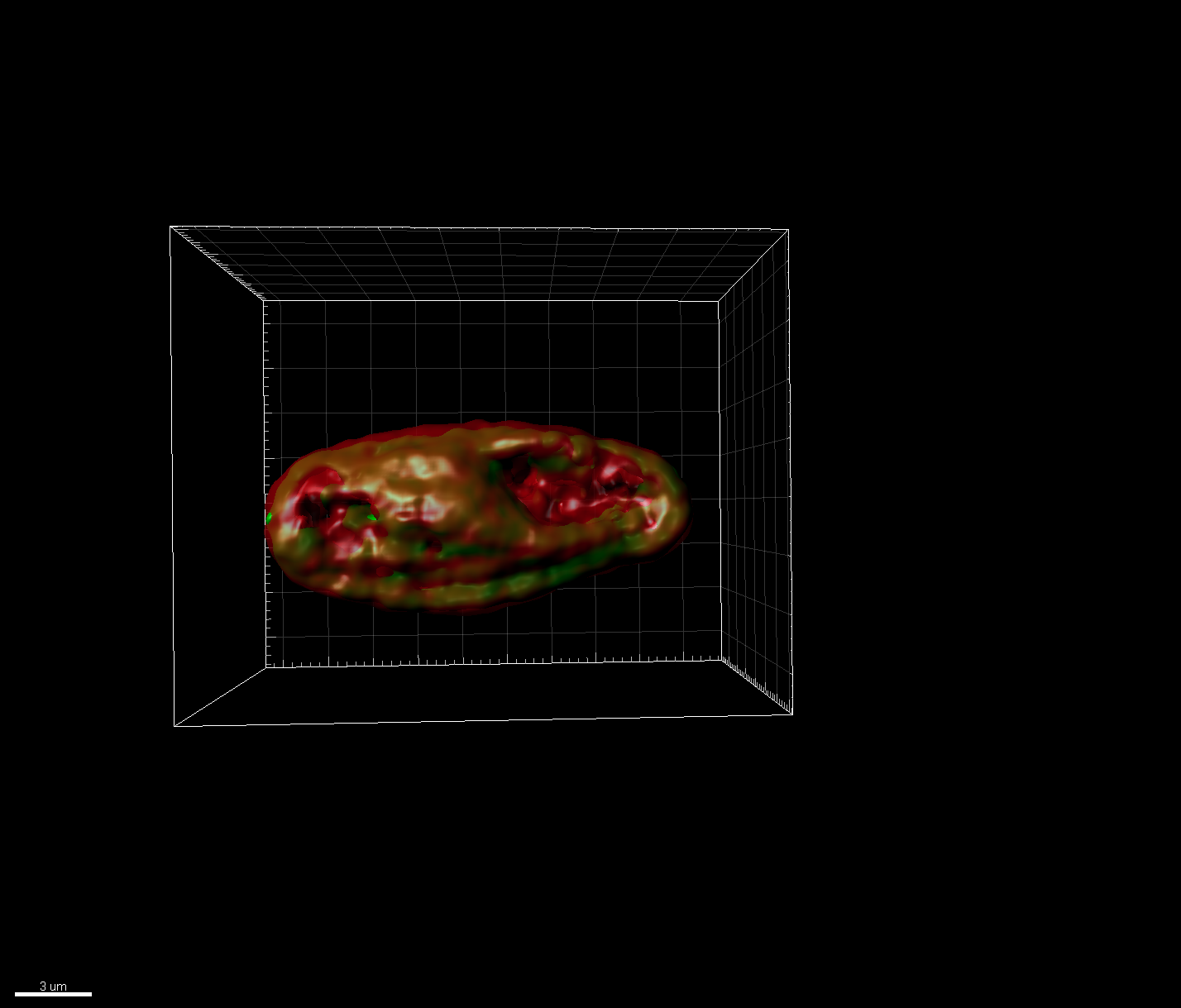

Supplement: Figure 1—source data 4. [file elife-85412-fig1-data4.zip › Fig 1- source data 4 Fig 1 D/3D reconstruction.tif]

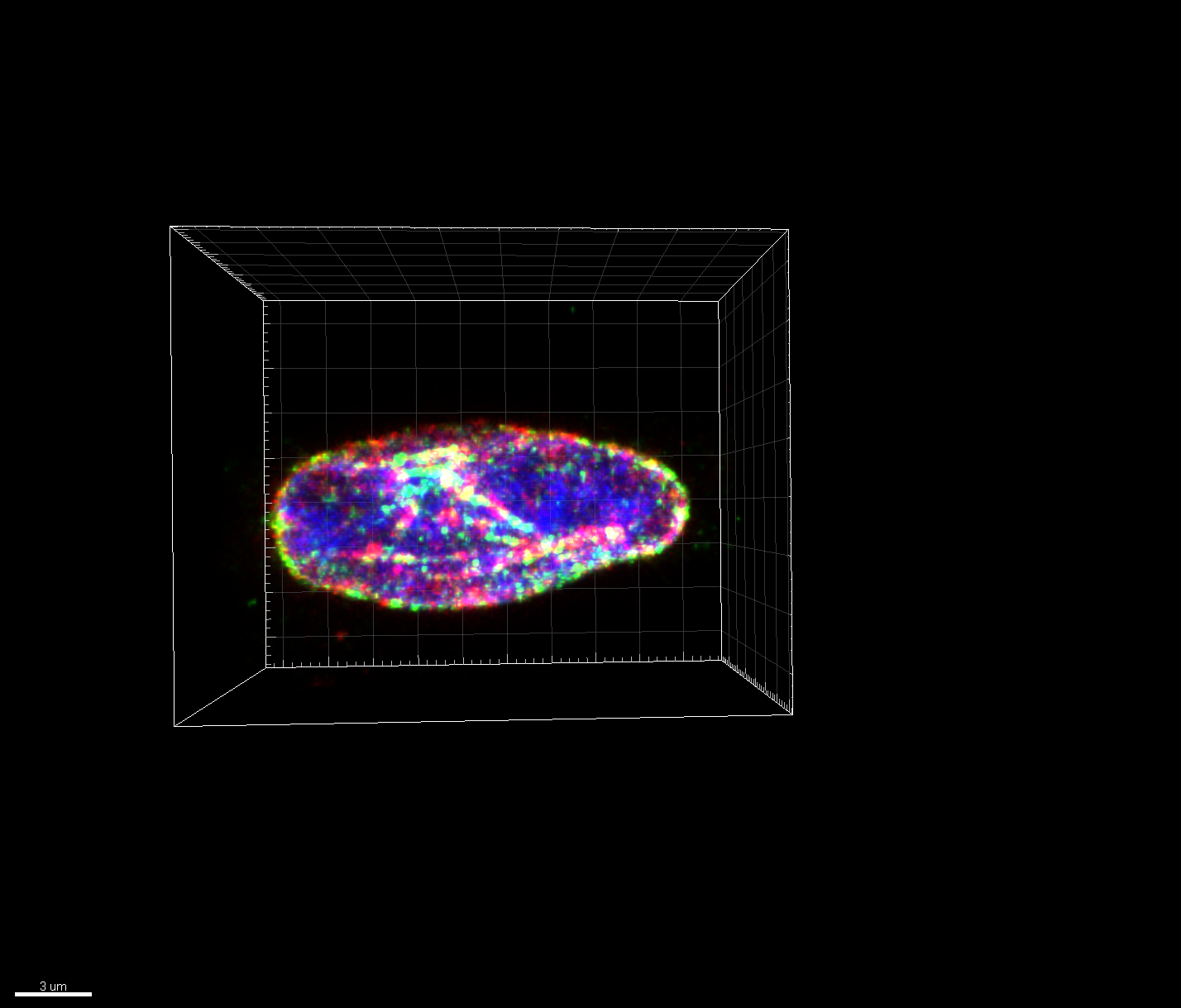

Supplement: Figure 1—source data 4. [file elife-85412-fig1-data4.zip › Fig 1- source data 4 Fig 1 D/CONFOCAL .tif]

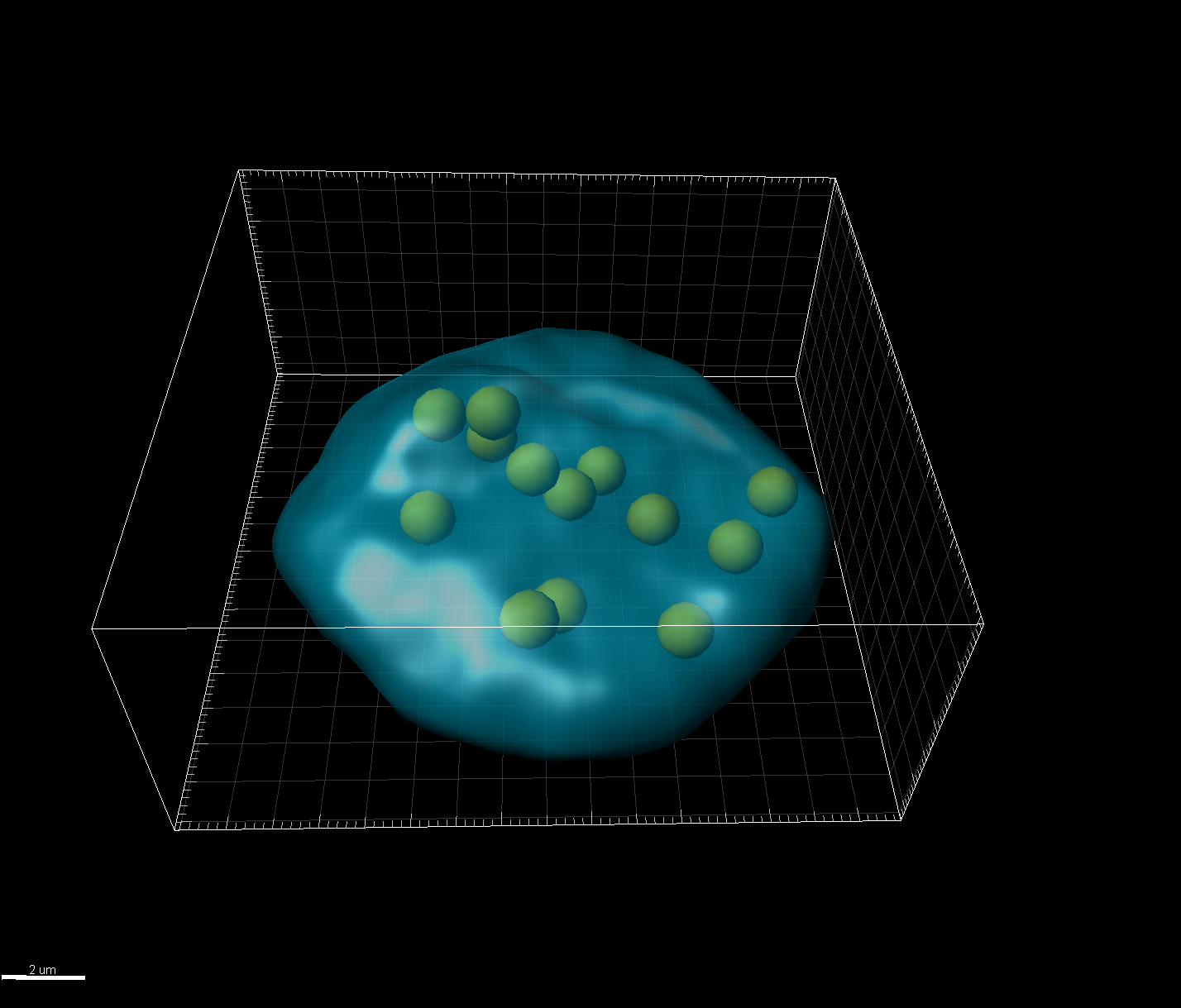

Supplement: Figure 1—source data 5. [file elife-85412-fig1-data5.zip › Fig 1- source data 5 Fig 1 E/0S 3D.tif]

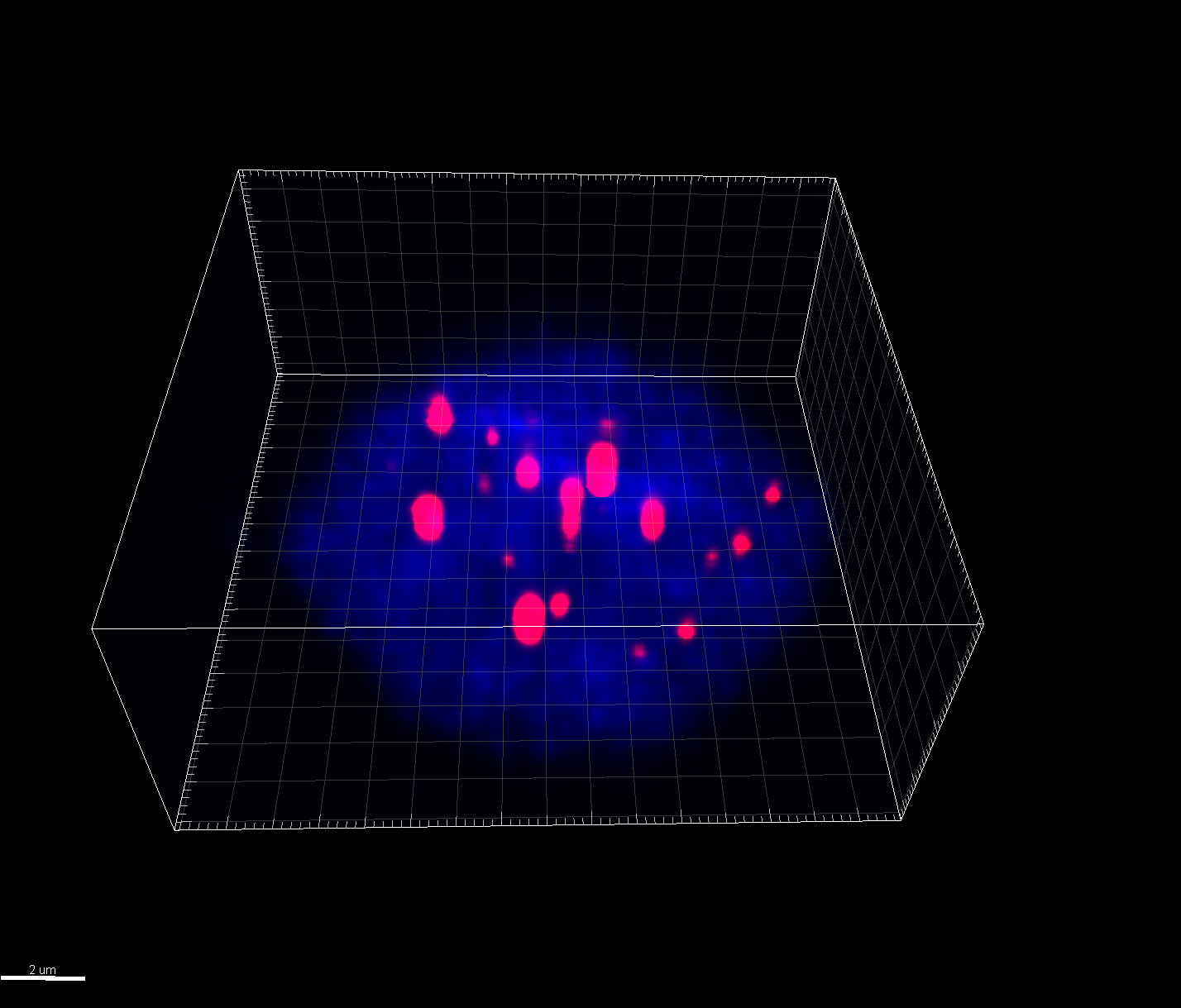

Supplement: Figure 1—source data 5. [file elife-85412-fig1-data5.zip › Fig 1- source data 5 Fig 1 E/0S.tif]

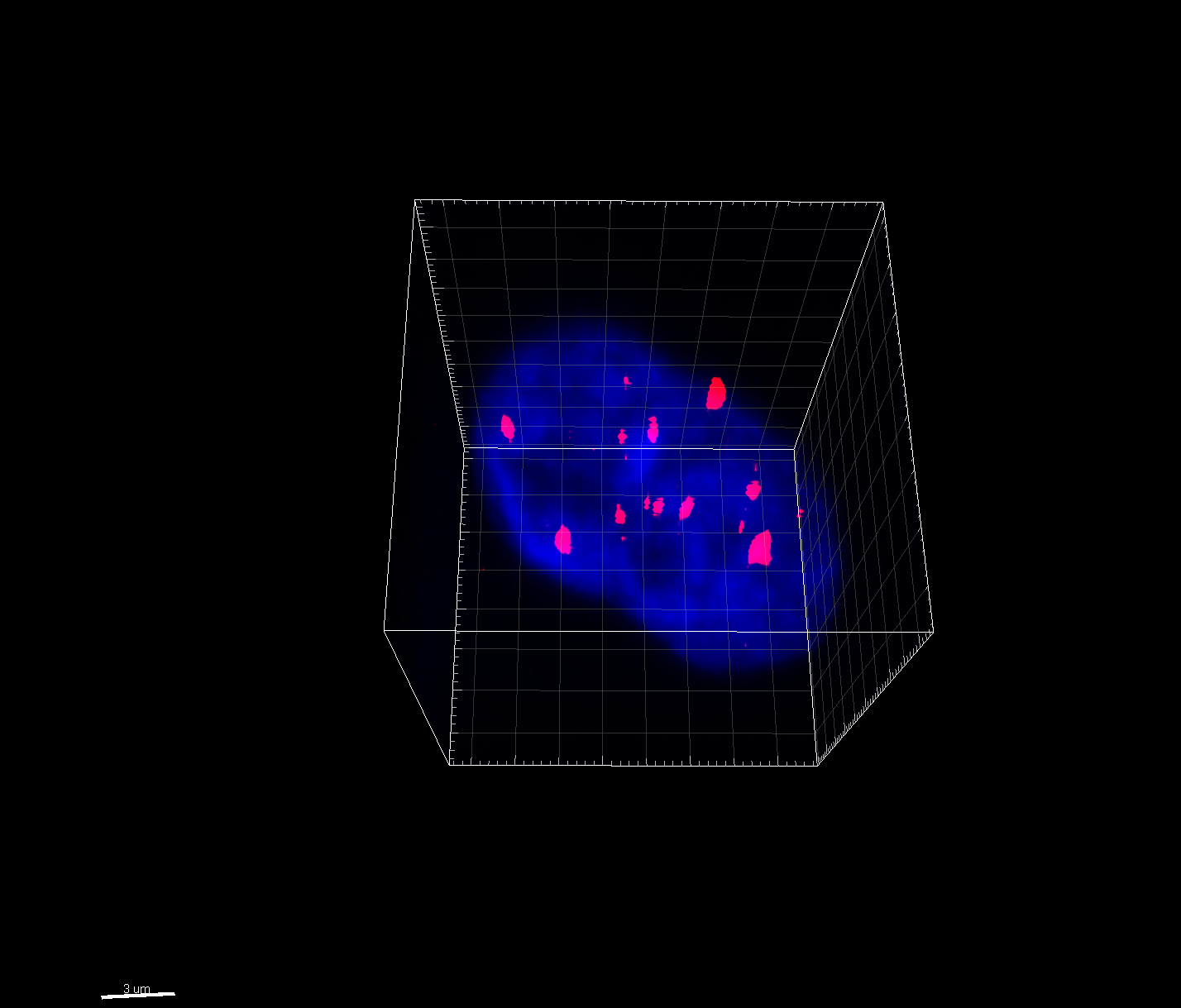

Supplement: Figure 1—source data 5. [file elife-85412-fig1-data5.zip › Fig 1- source data 5 Fig 1 E/10 s.tif]

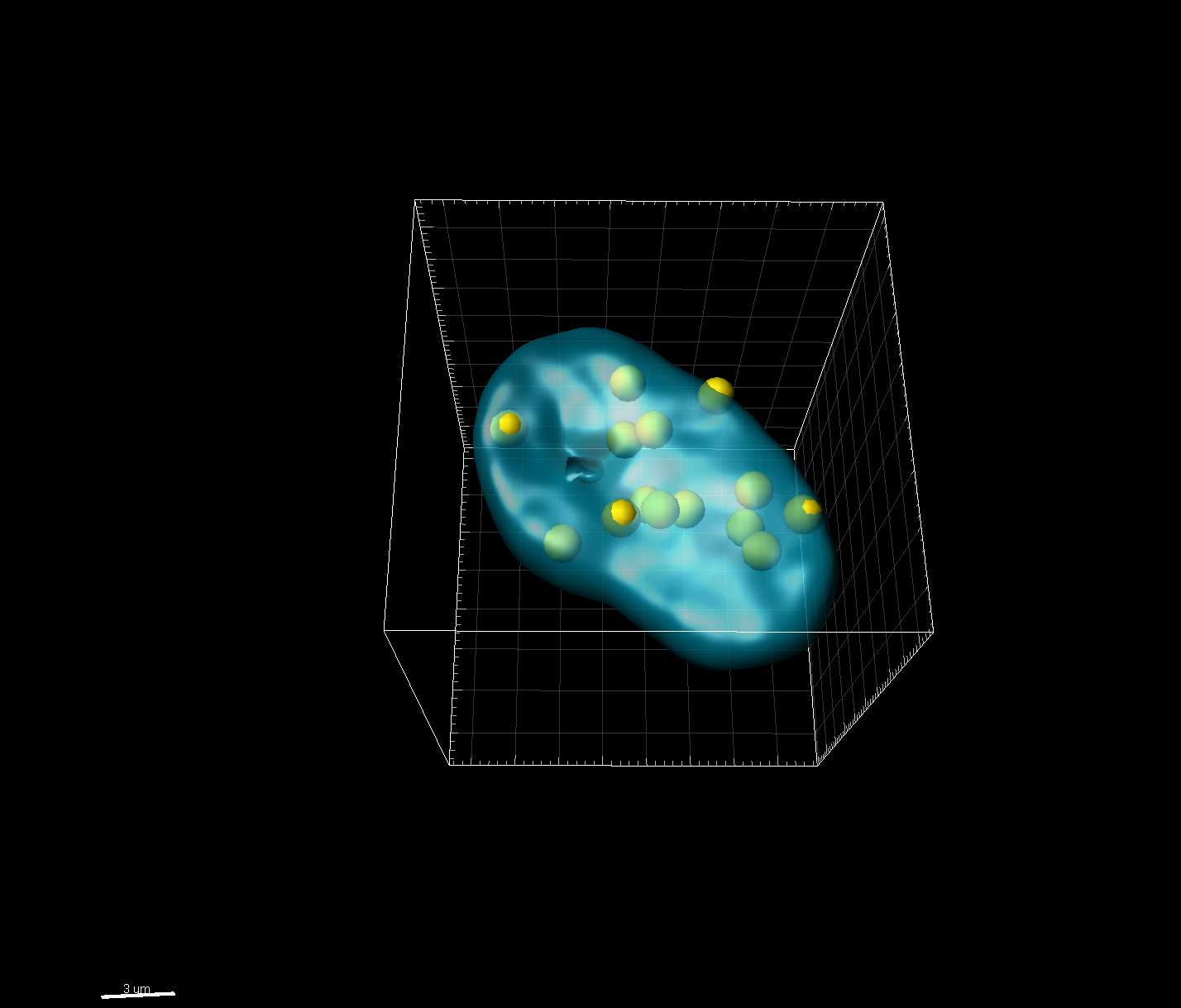

Supplement: Figure 1—source data 5. [file elife-85412-fig1-data5.zip › Fig 1- source data 5 Fig 1 E/10s 3D.tif]

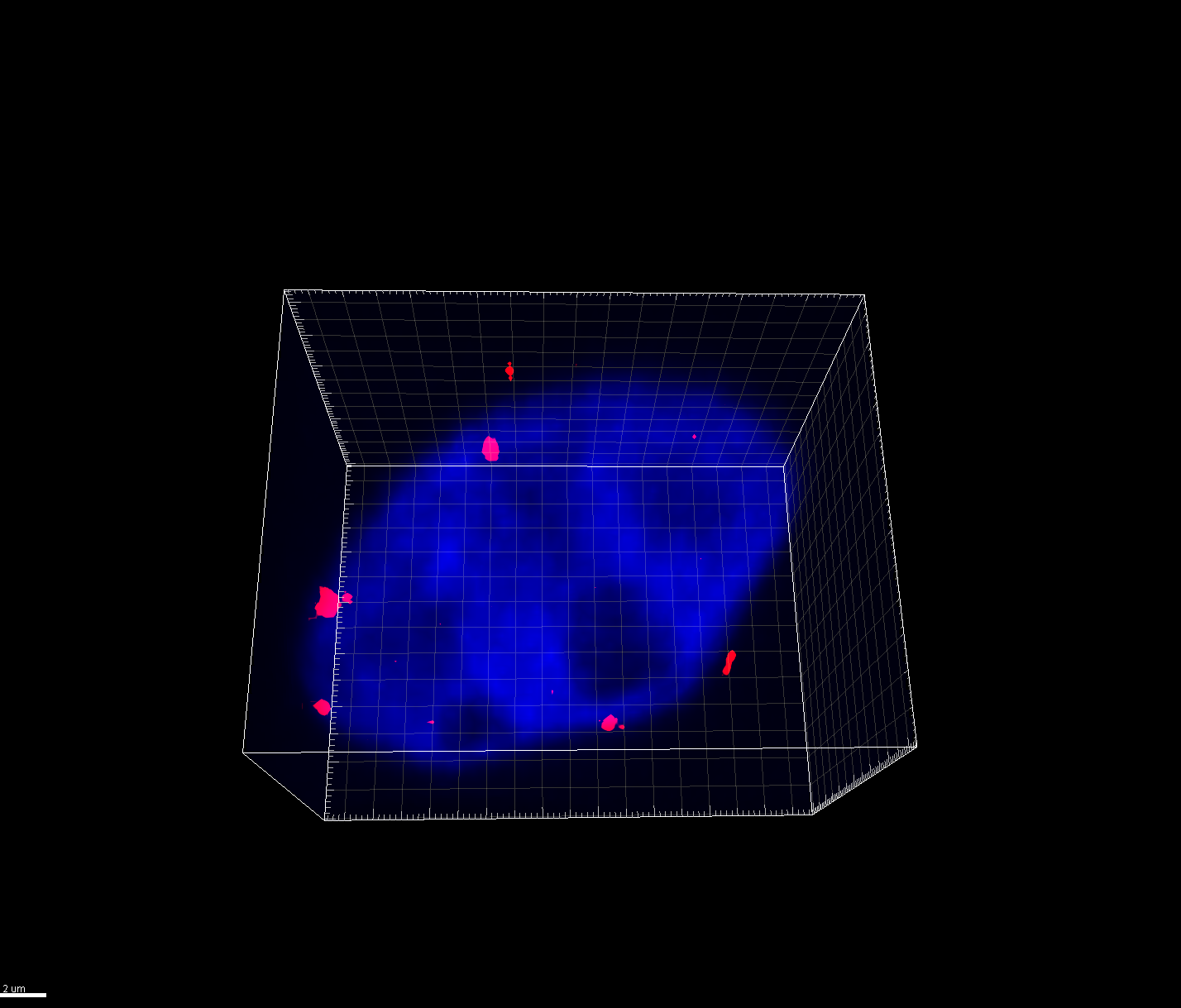

Supplement: Figure 1—source data 5. [file elife-85412-fig1-data5.zip › Fig 1- source data 5 Fig 1 E/180S .tif]

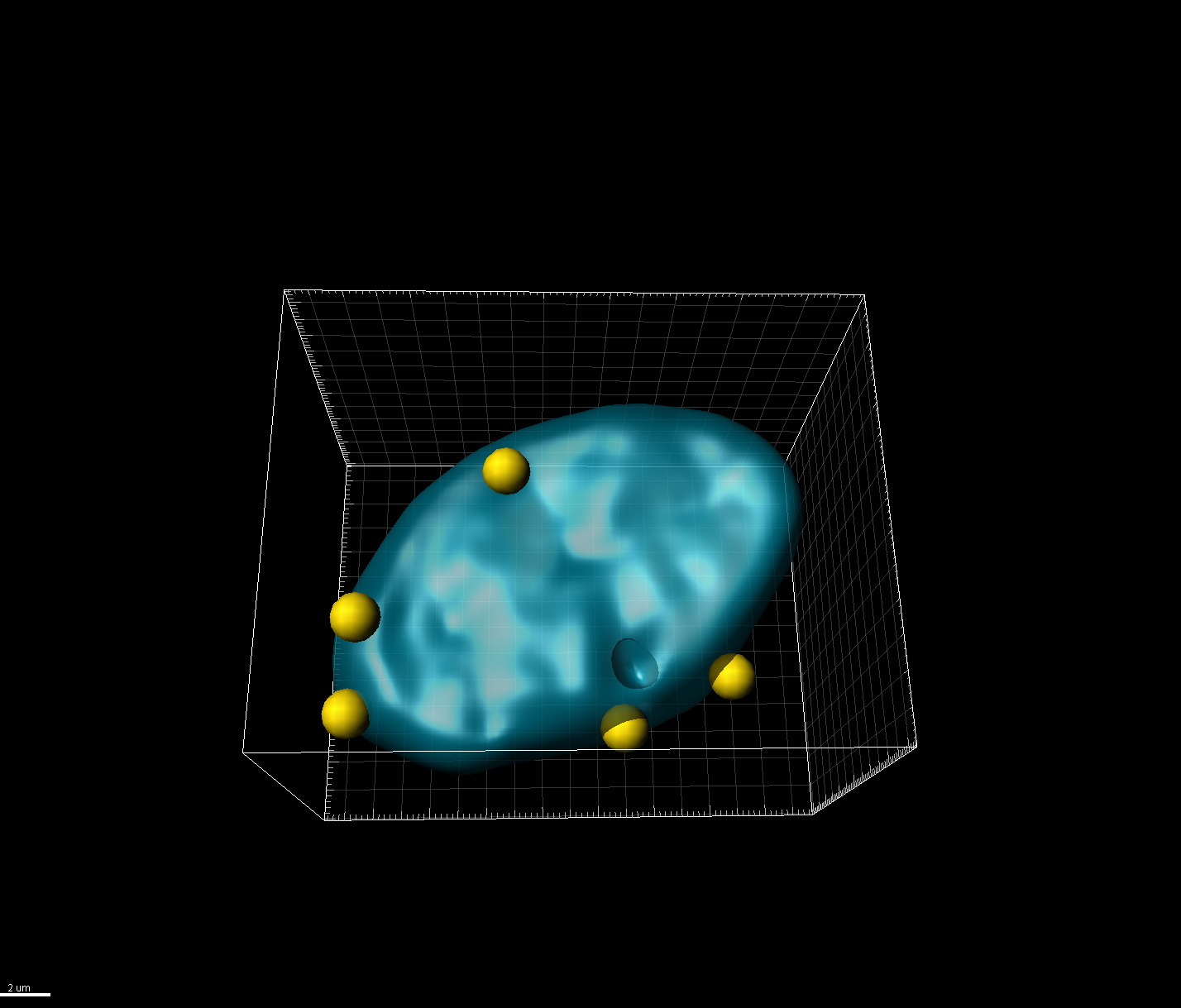

Supplement: Figure 1—source data 5. [file elife-85412-fig1-data5.zip › Fig 1- source data 5 Fig 1 E/180s 3D.tif]

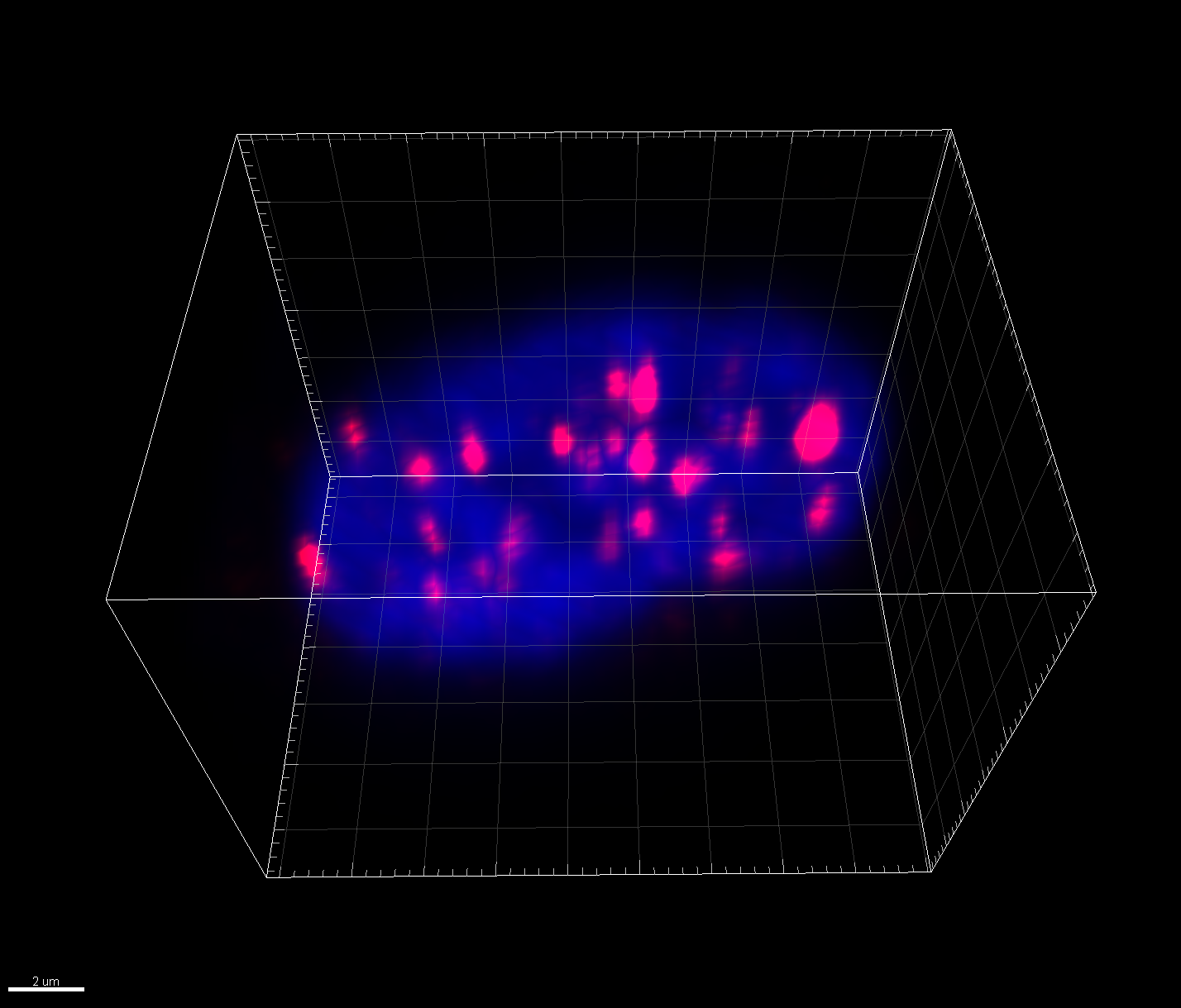

Supplement: Figure 1—source data 5. [file elife-85412-fig1-data5.zip › Fig 1- source data 5 Fig 1 E/20 s.tif]

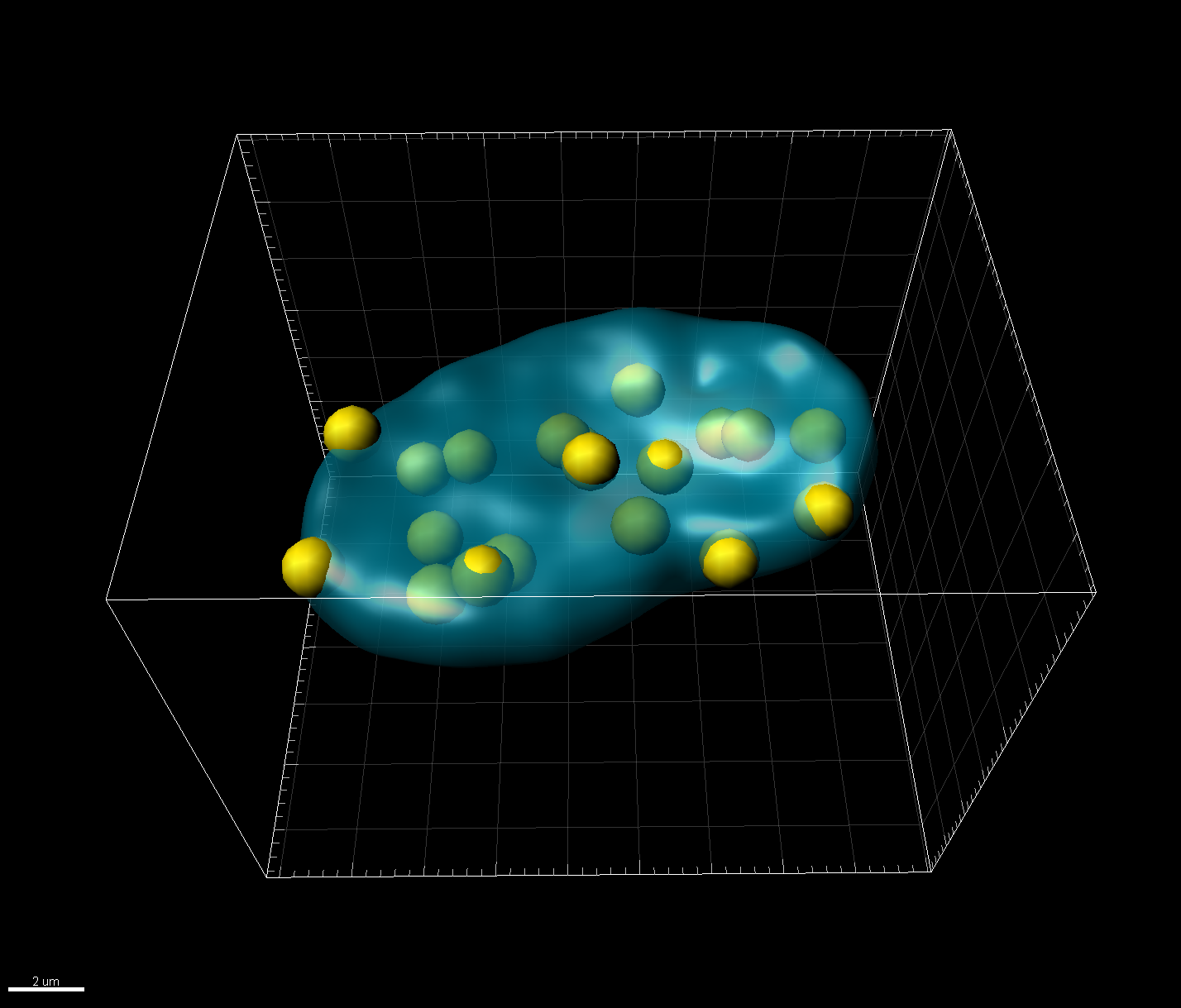

Supplement: Figure 1—source data 5. [file elife-85412-fig1-data5.zip › Fig 1- source data 5 Fig 1 E/20s 3D.tif]

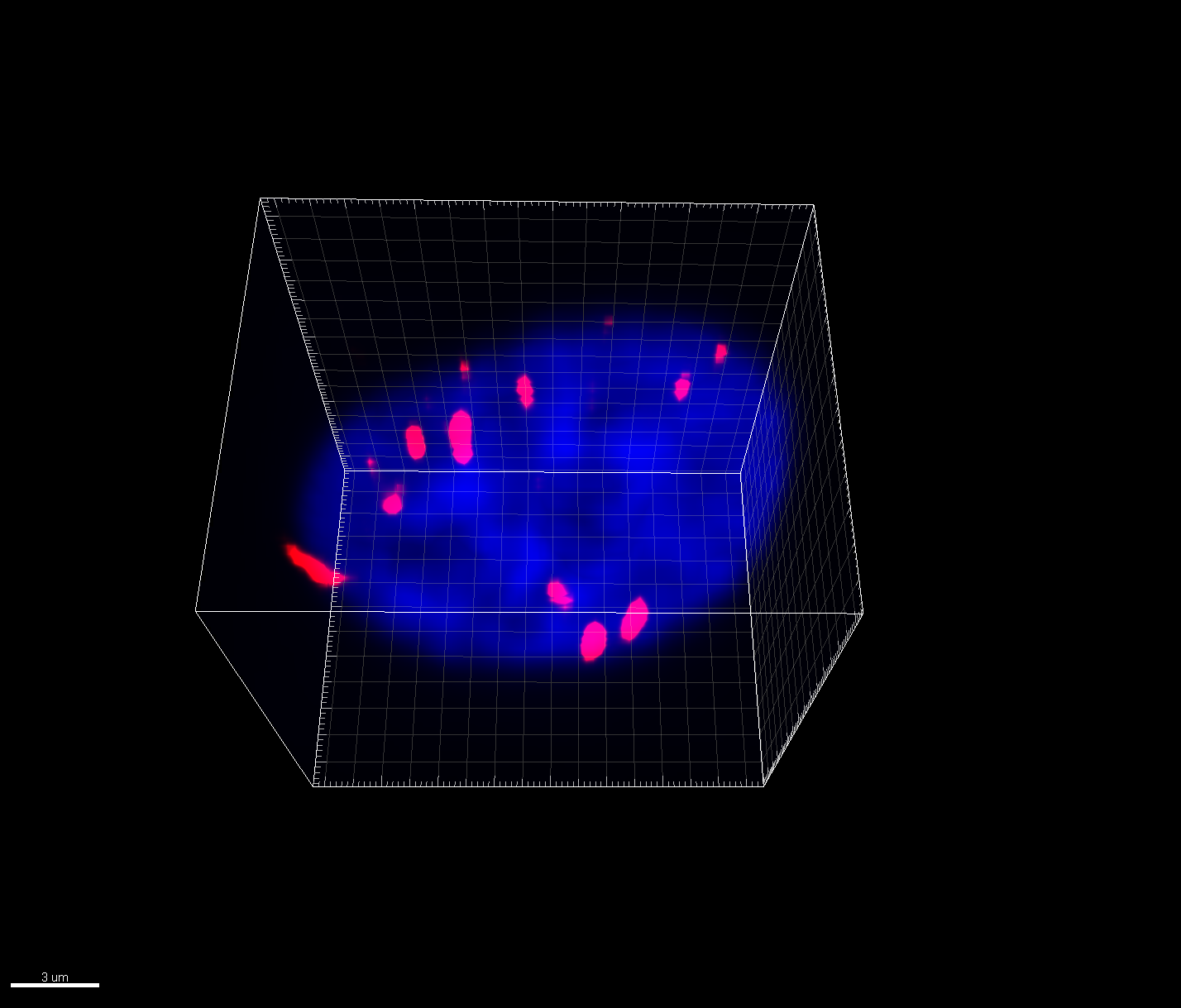

Supplement: Figure 1—source data 5. [file elife-85412-fig1-data5.zip › Fig 1- source data 5 Fig 1 E/40s .tif]

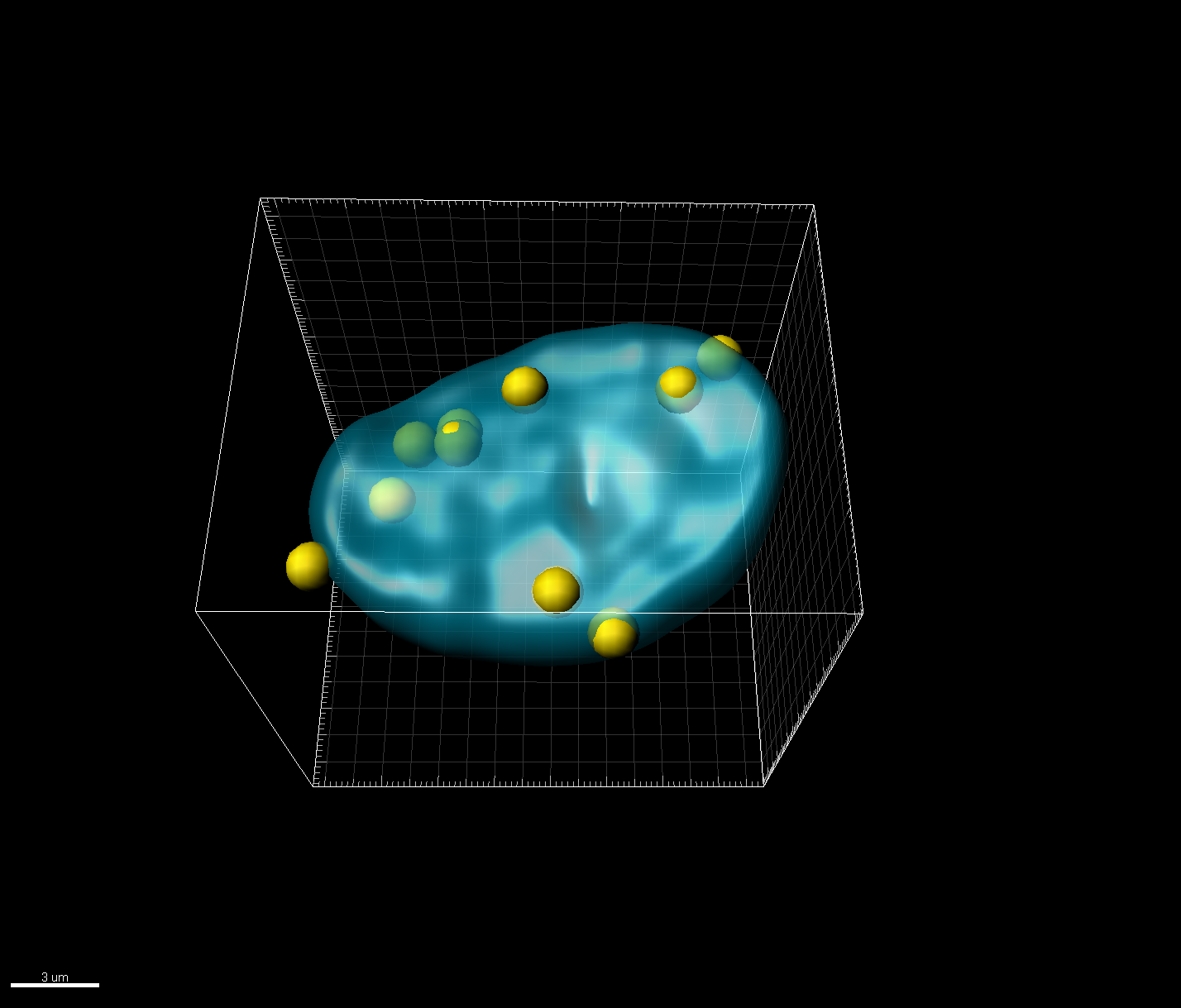

Supplement: Figure 1—source data 5. [file elife-85412-fig1-data5.zip › Fig 1- source data 5 Fig 1 E/40s 3 D.tif]

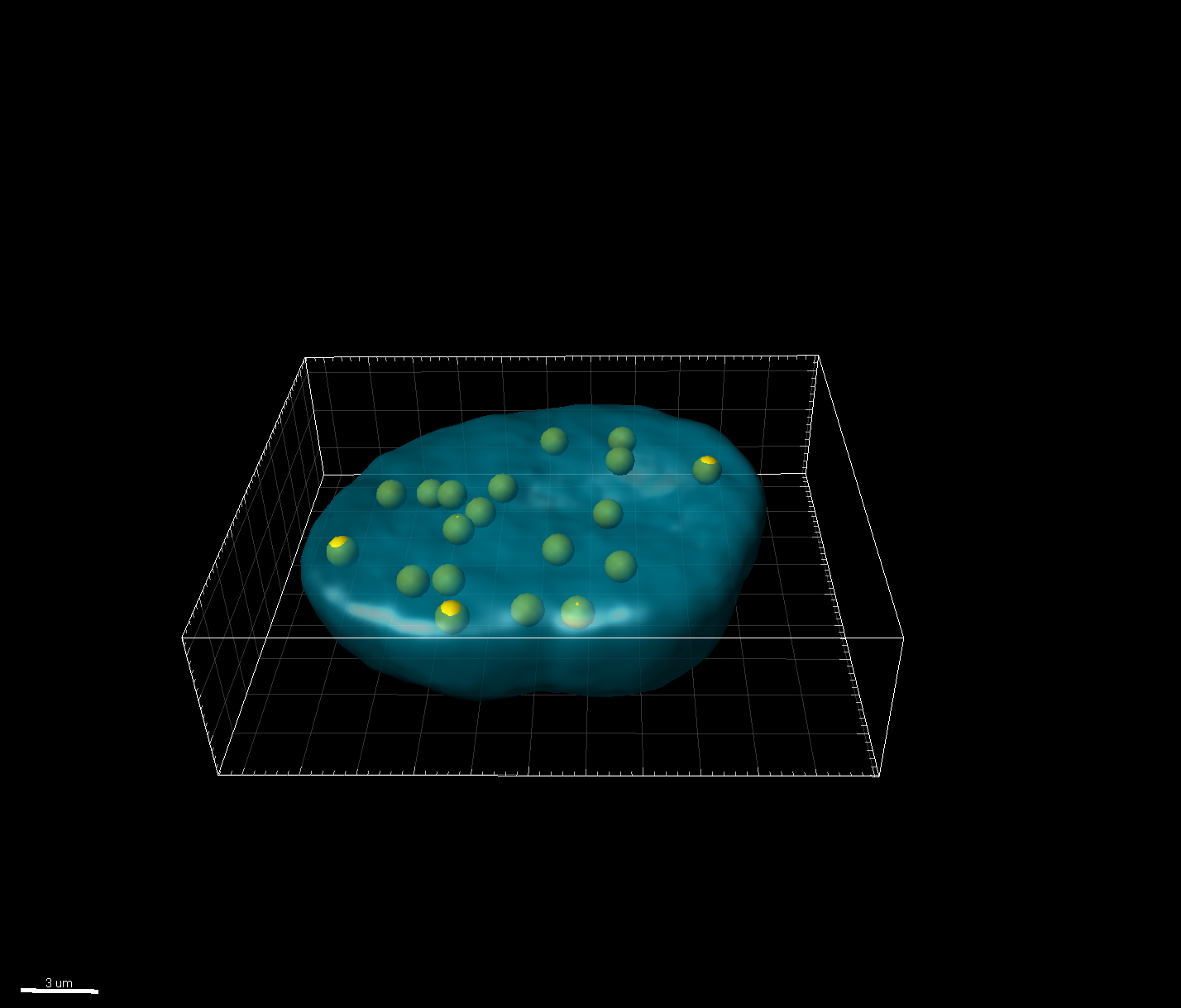

Supplement: Figure 1—source data 5. [file elife-85412-fig1-data5.zip › Fig 1- source data 5 Fig 1 E/Ctr sgRNA 3D.tif]

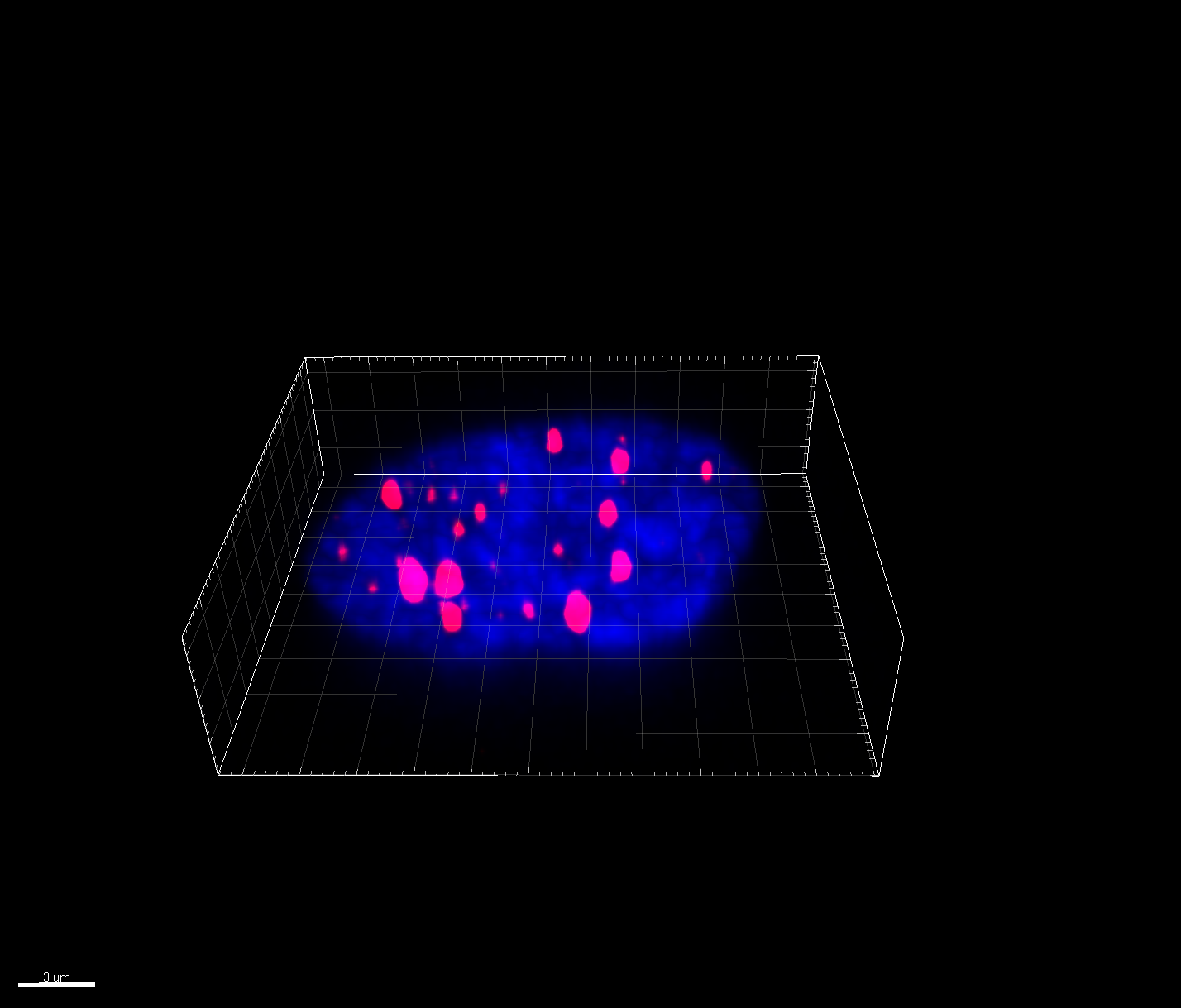

Supplement: Figure 1—source data 5. [file elife-85412-fig1-data5.zip › Fig 1- source data 5 Fig 1 E/ctr sgRNA.tif]

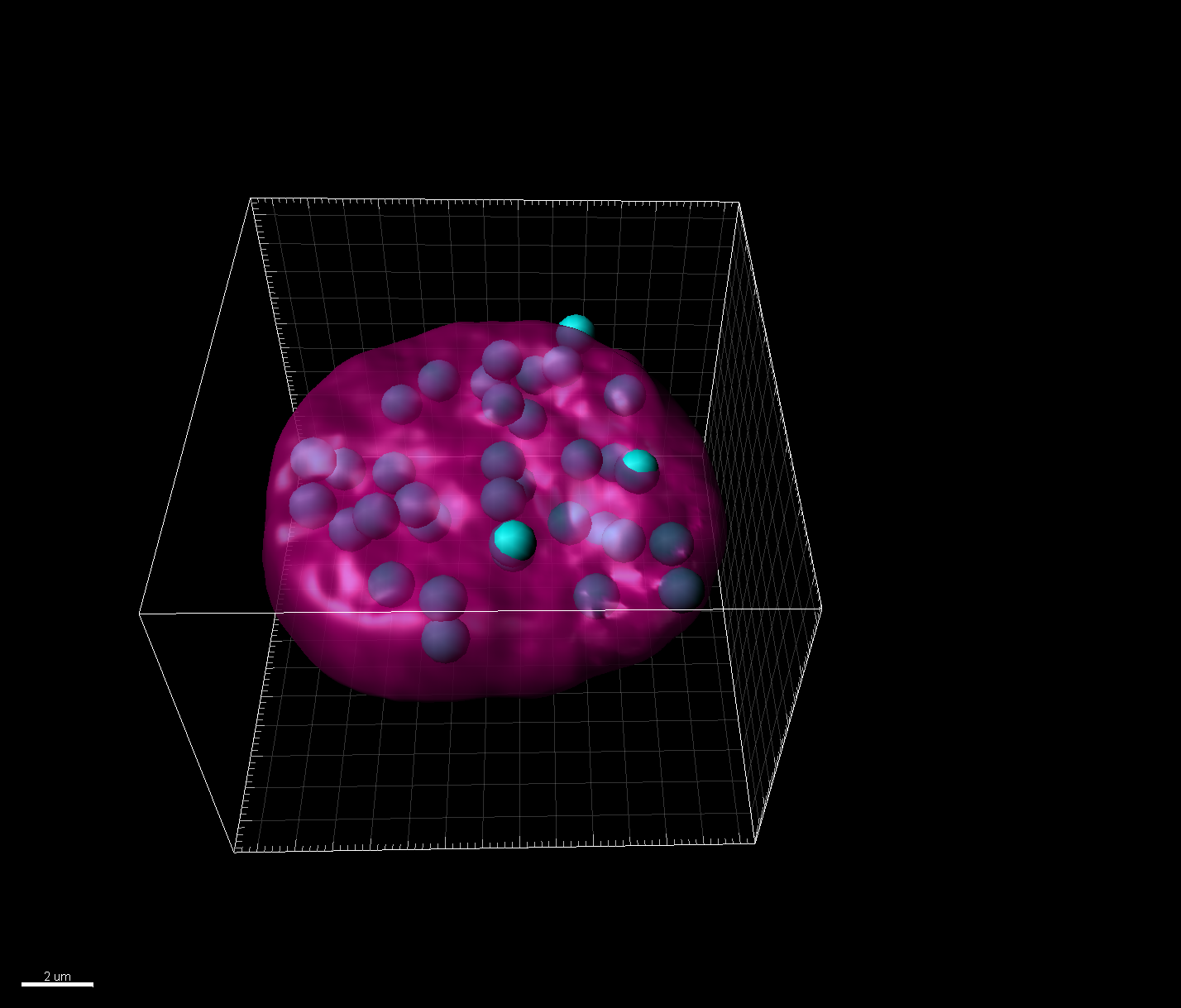

Supplement: Figure 1—source data 7. [file elife-85412-fig1-data7.zip › Fig 1- source data 7 Fig 1 G/0h (1).tif]

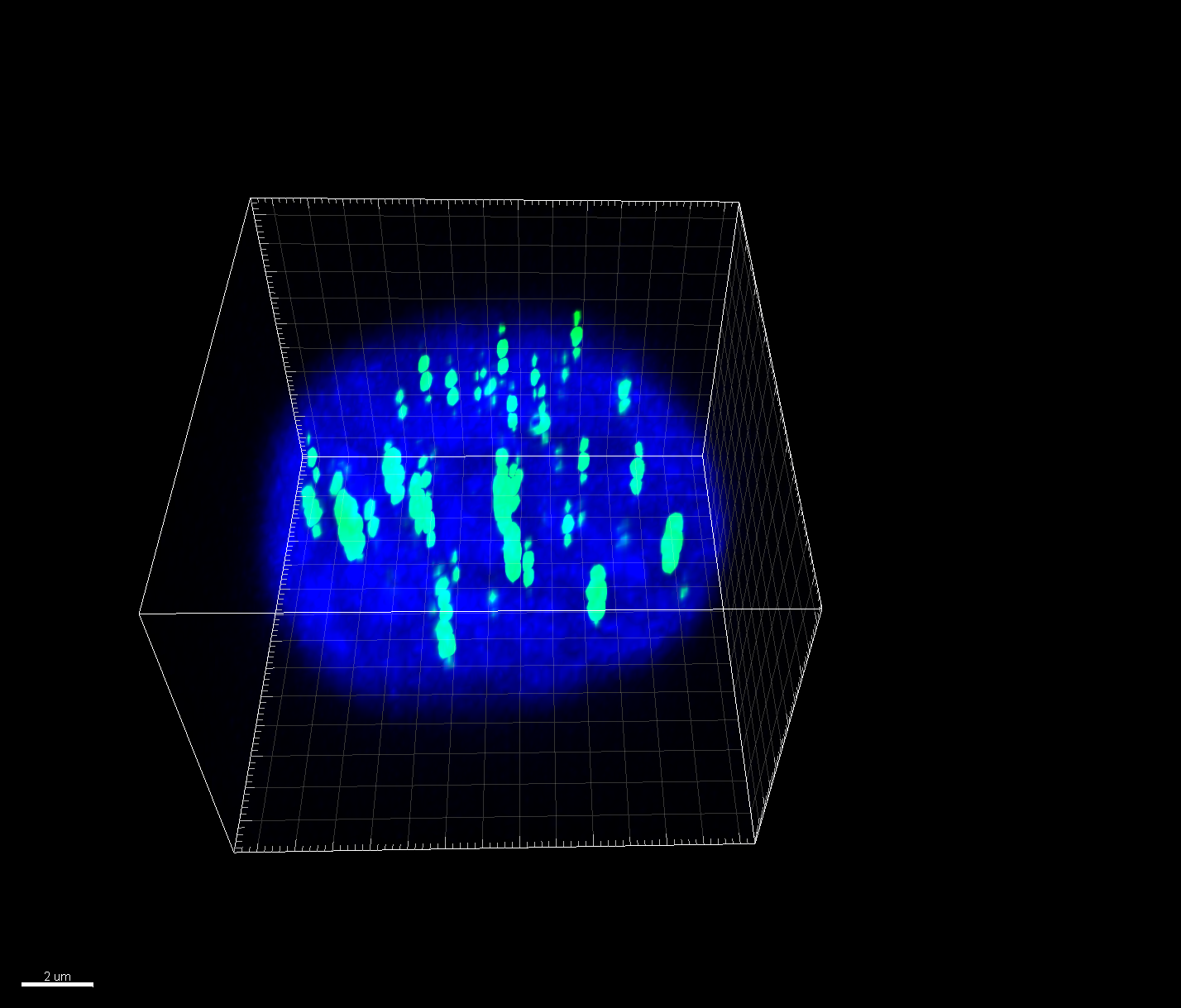

Supplement: Figure 1—source data 7. [file elife-85412-fig1-data7.zip › Fig 1- source data 7 Fig 1 G/0h (2).tif]

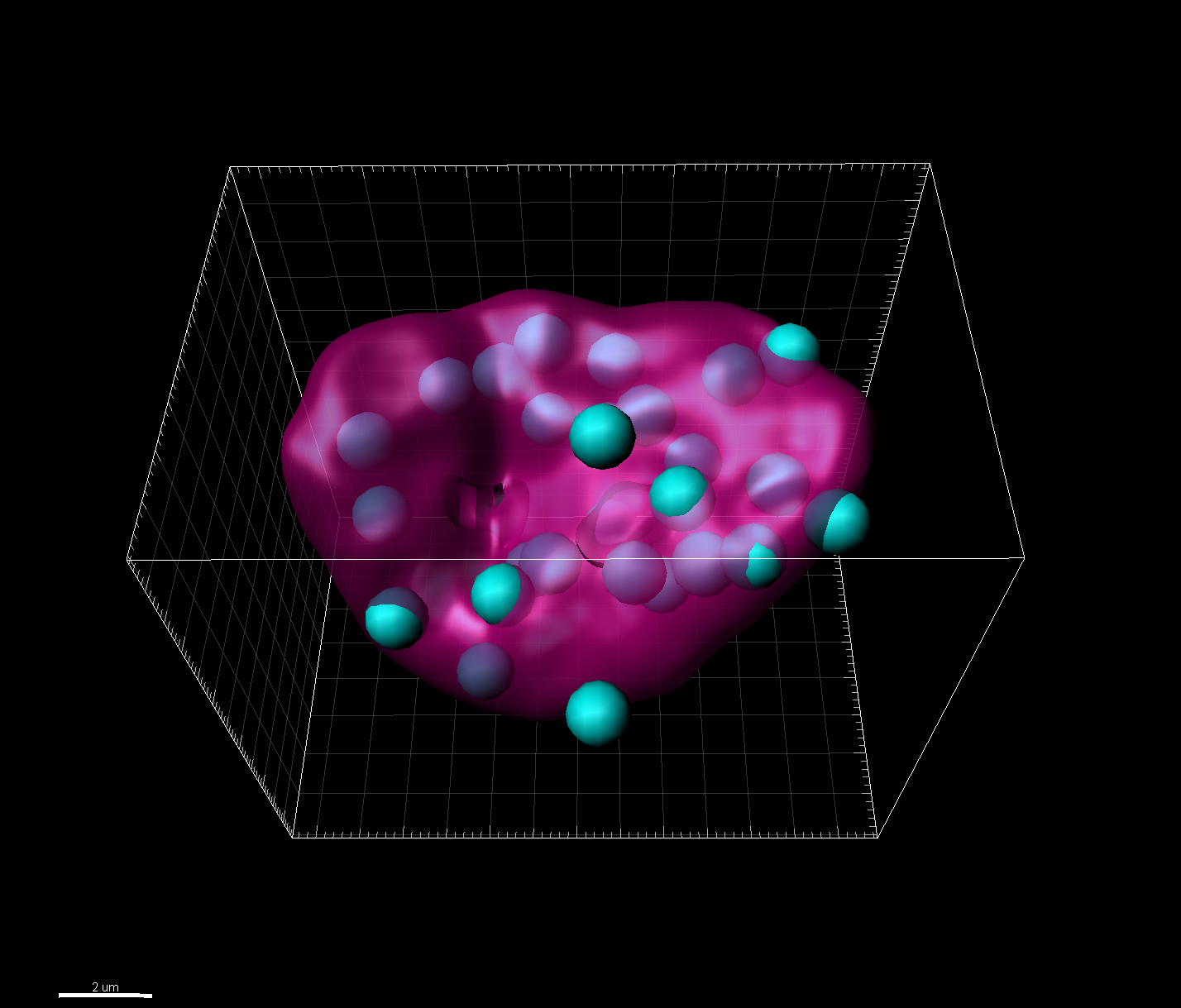

Supplement: Figure 1—source data 7. [file elife-85412-fig1-data7.zip › Fig 1- source data 7 Fig 1 G/1 hr (1).tif]

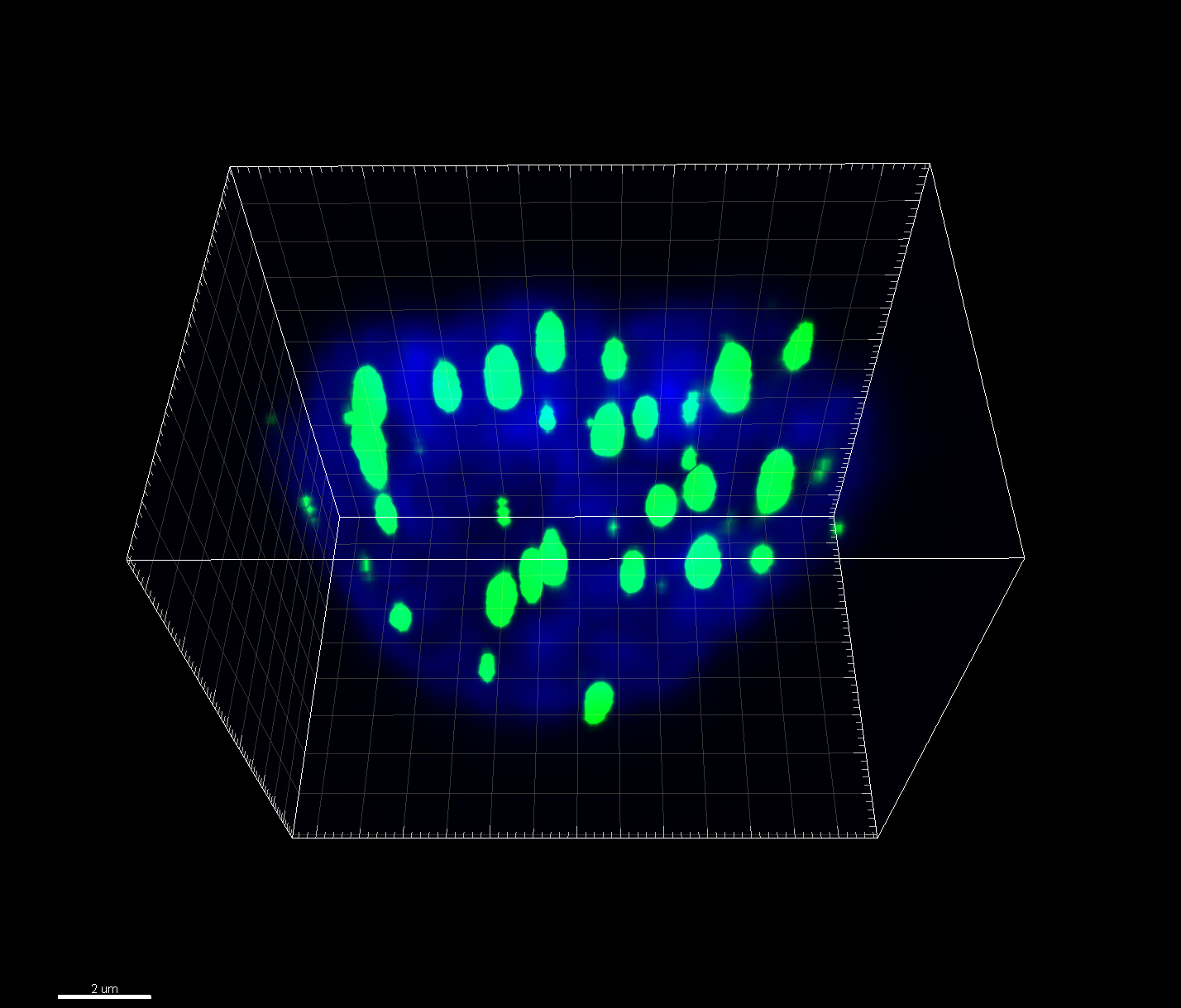

Supplement: Figure 1—source data 7. [file elife-85412-fig1-data7.zip › Fig 1- source data 7 Fig 1 G/1 hr (2).tif]

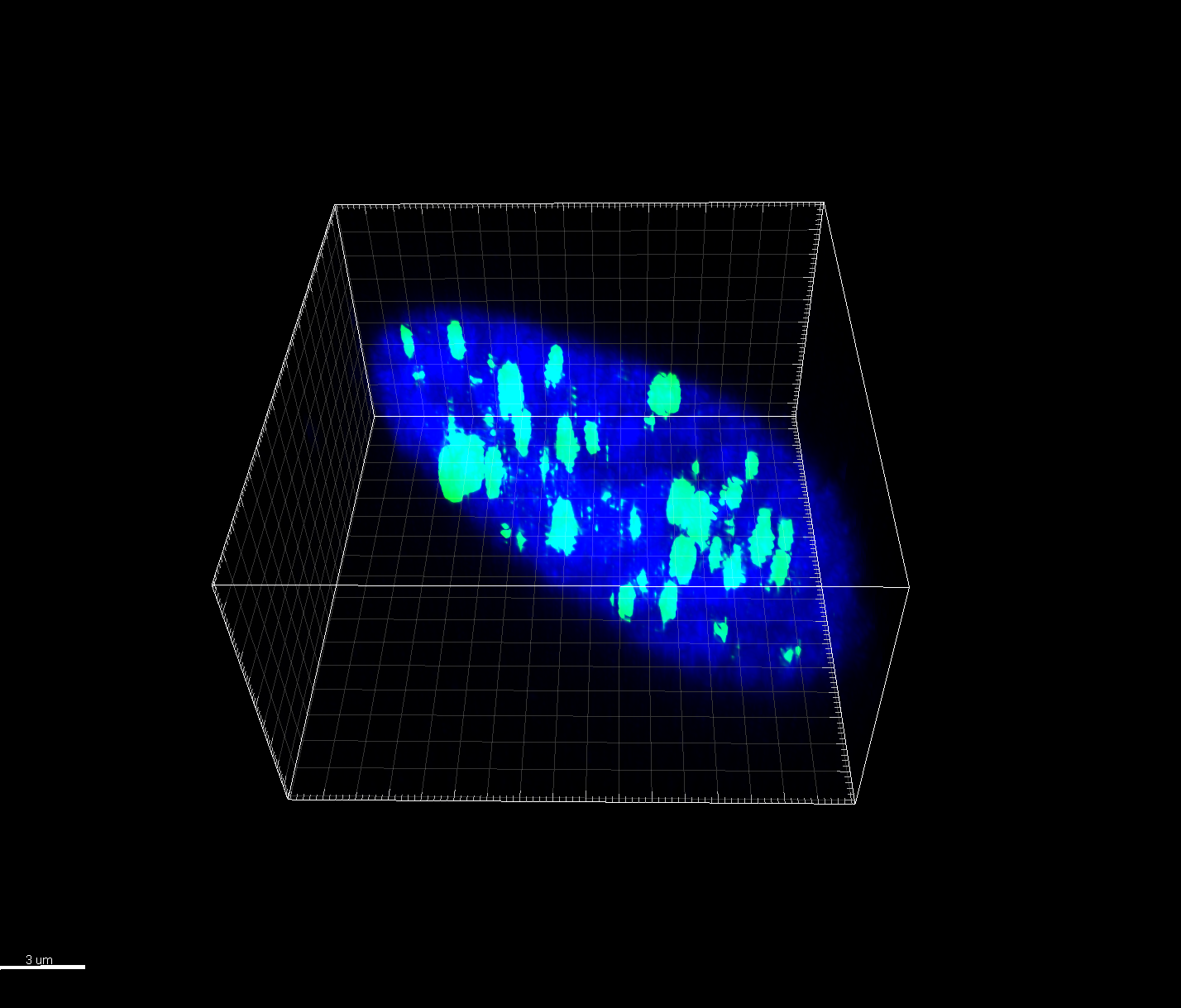

Supplement: Figure 1—source data 7. [file elife-85412-fig1-data7.zip › Fig 1- source data 7 Fig 1 G/24 hr (1).tif]

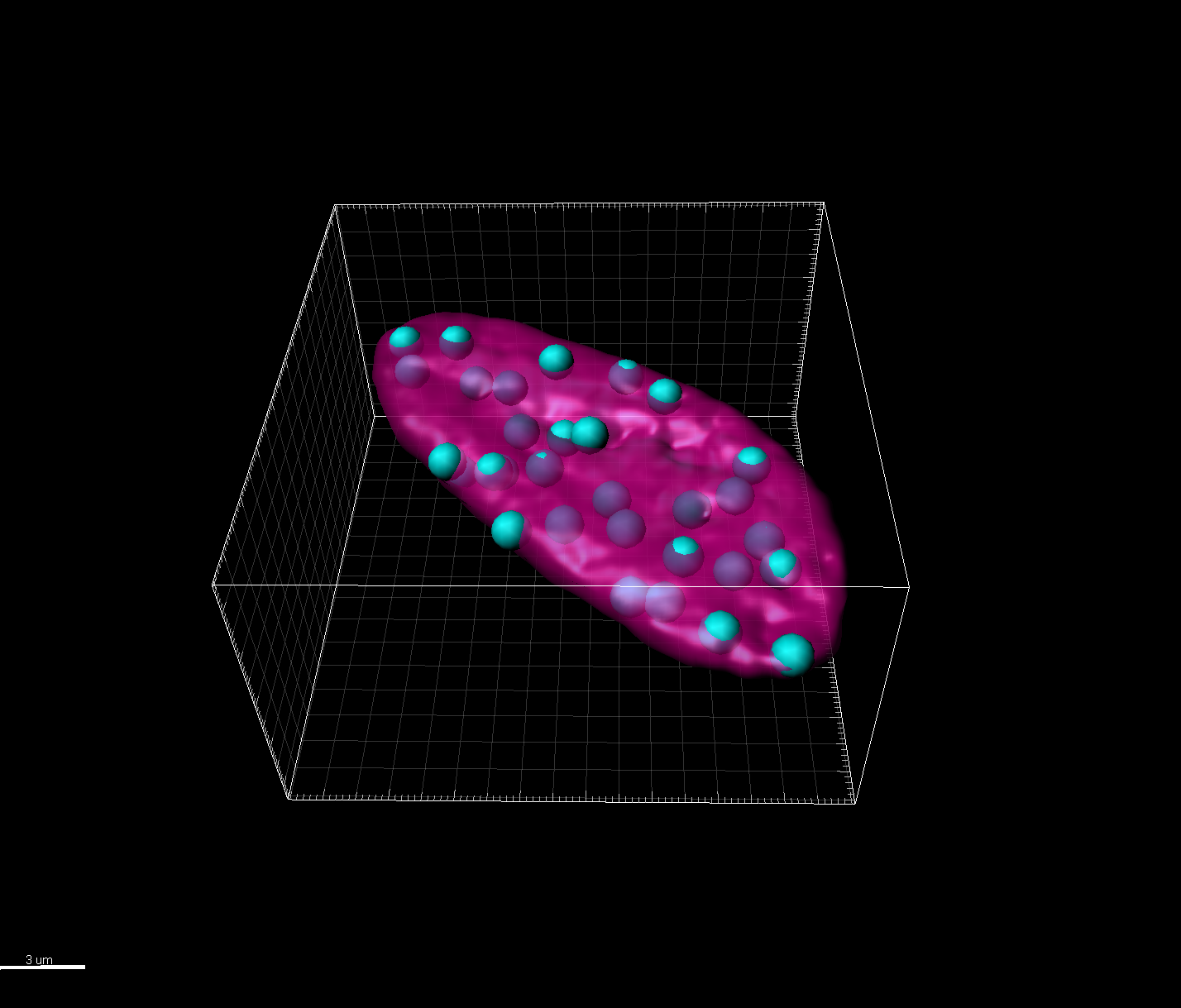

Supplement: Figure 1—source data 7. [file elife-85412-fig1-data7.zip › Fig 1- source data 7 Fig 1 G/24 hr (2).tif]

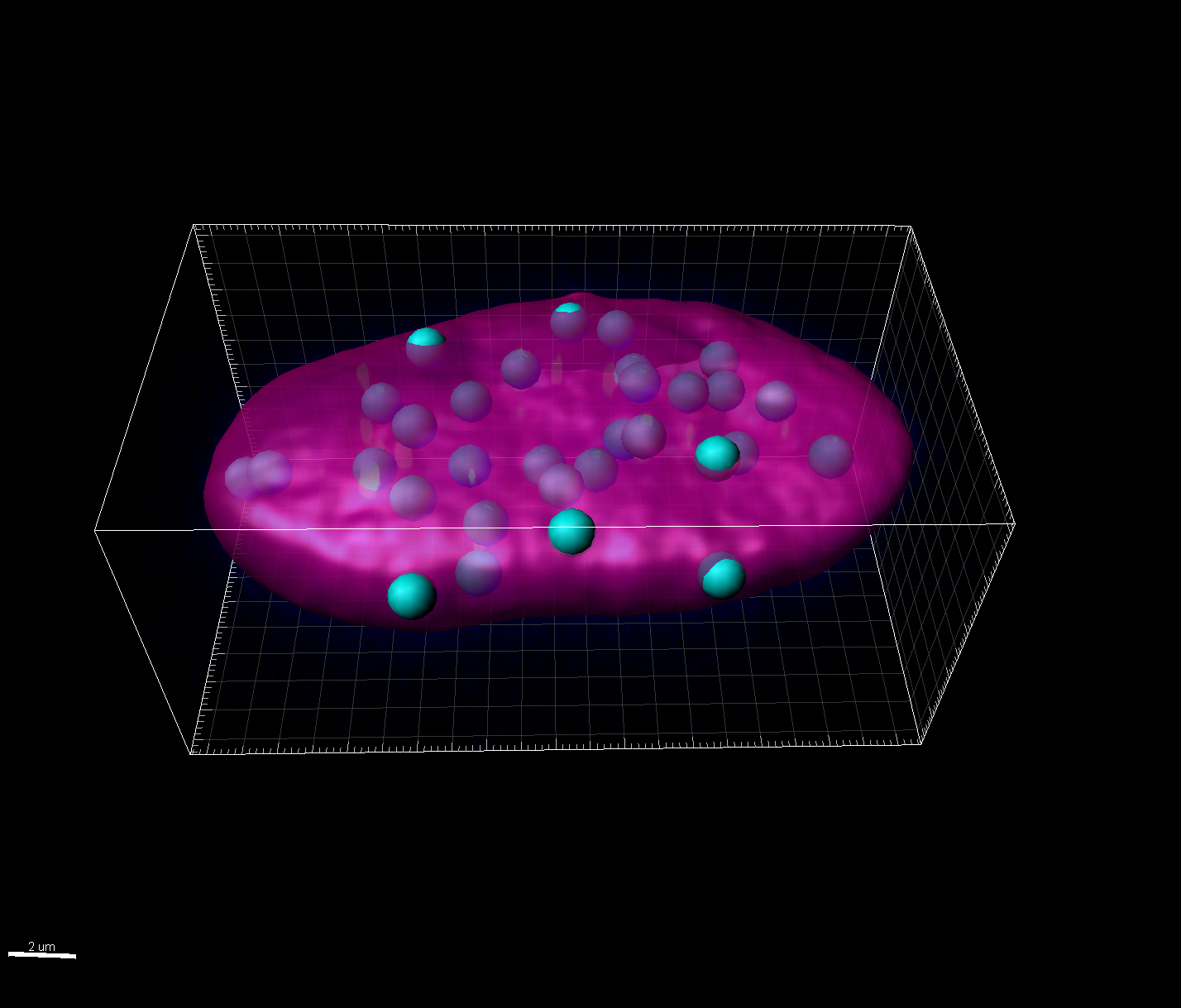

Supplement: Figure 1—source data 7. [file elife-85412-fig1-data7.zip › Fig 1- source data 7 Fig 1 G/24 hr control (1).tif]

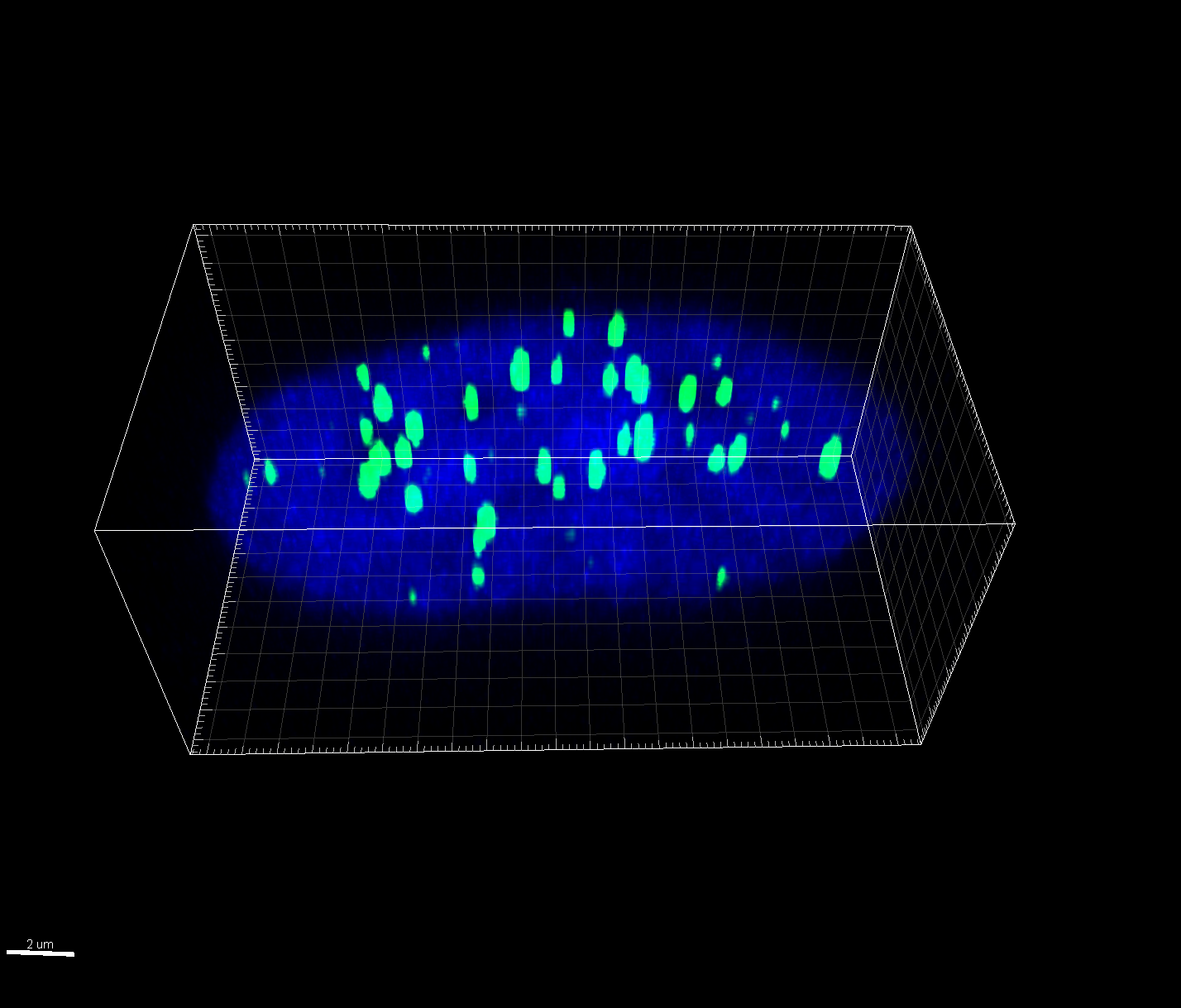

Supplement: Figure 1—source data 7. [file elife-85412-fig1-data7.zip › Fig 1- source data 7 Fig 1 G/24 hr control (2).tif]

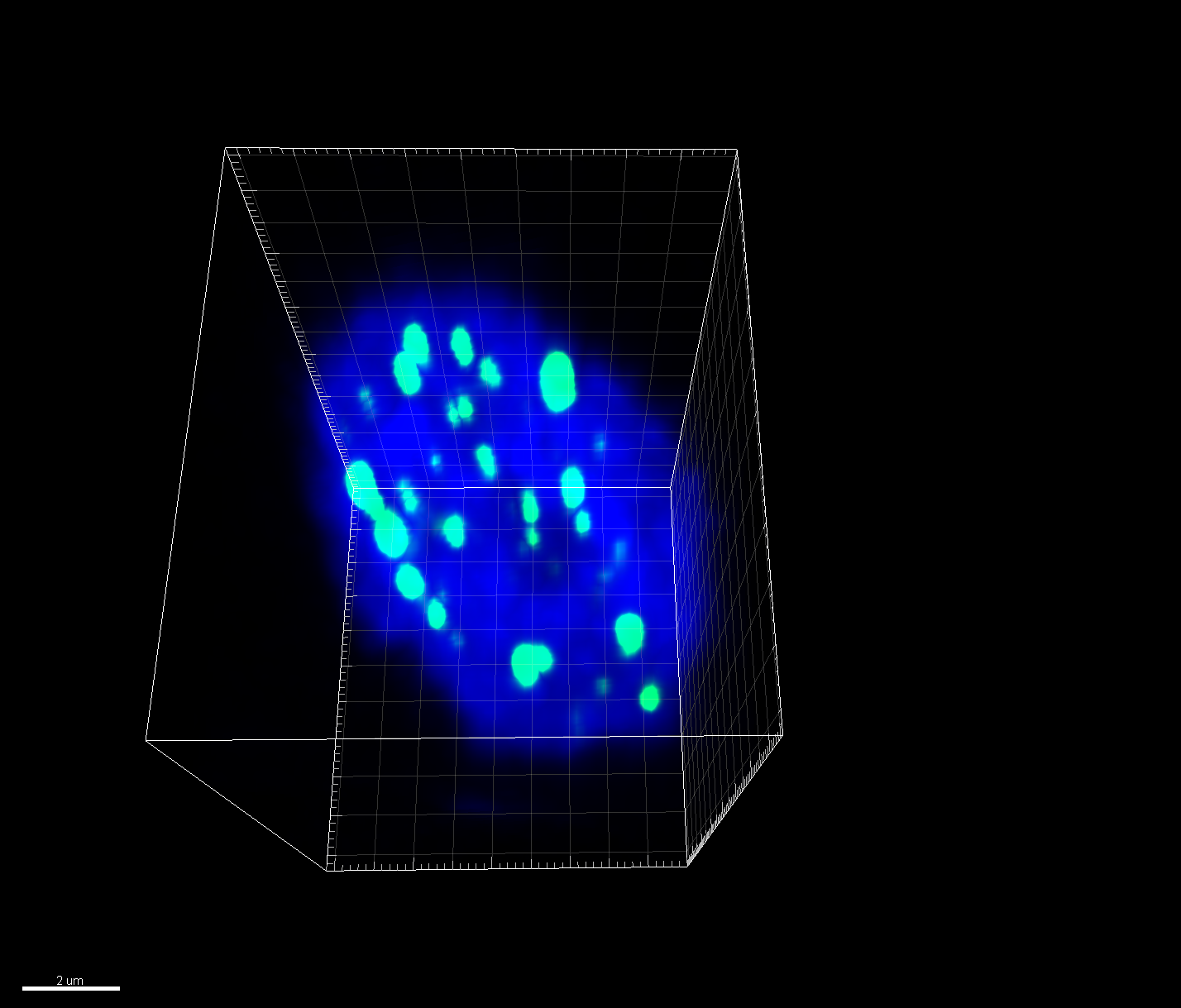

Supplement: Figure 1—source data 7. [file elife-85412-fig1-data7.zip › Fig 1- source data 7 Fig 1 G/3 hr (1).tif]

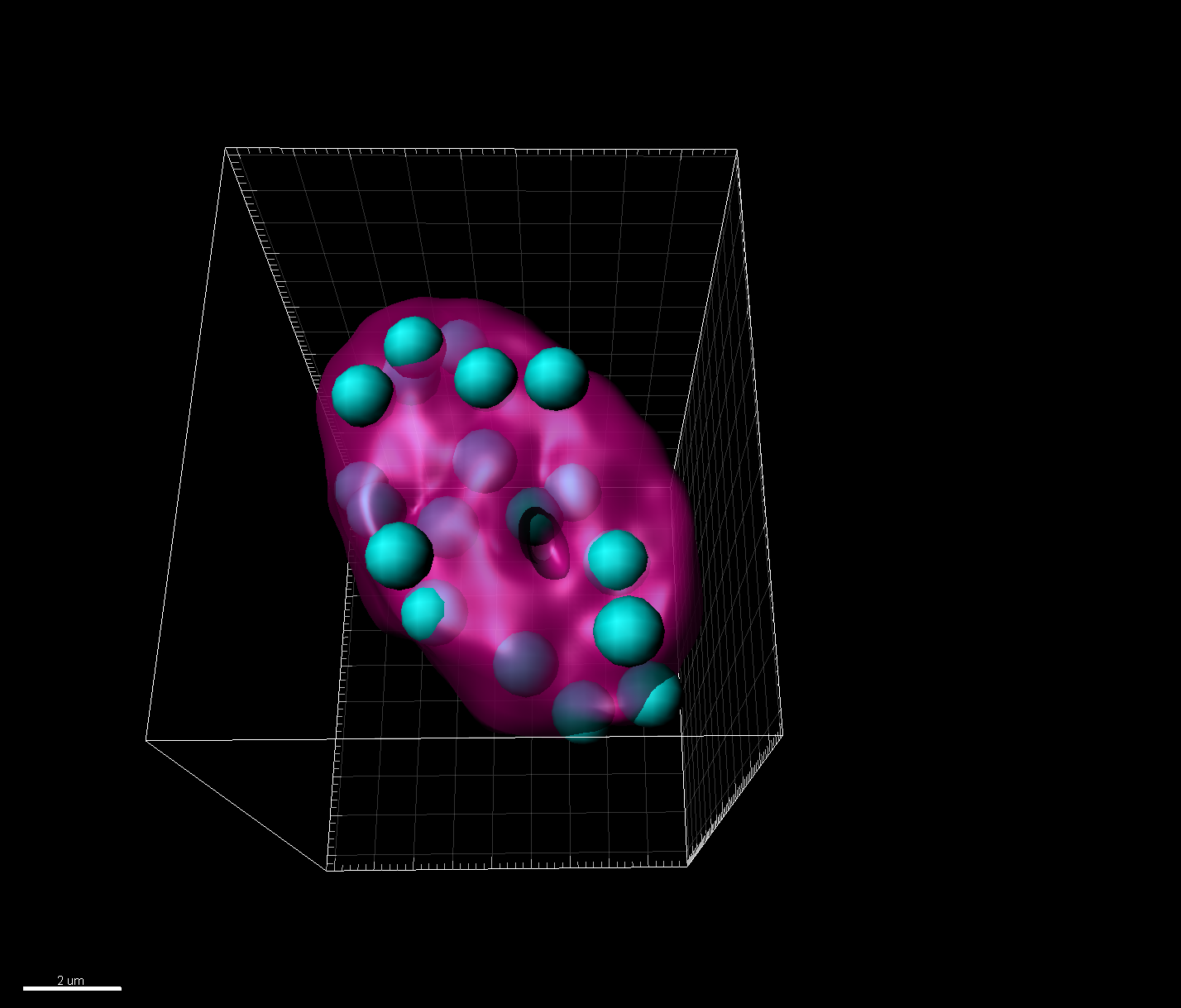

Supplement: Figure 1—source data 7. [file elife-85412-fig1-data7.zip › Fig 1- source data 7 Fig 1 G/3 hr (2).tif]

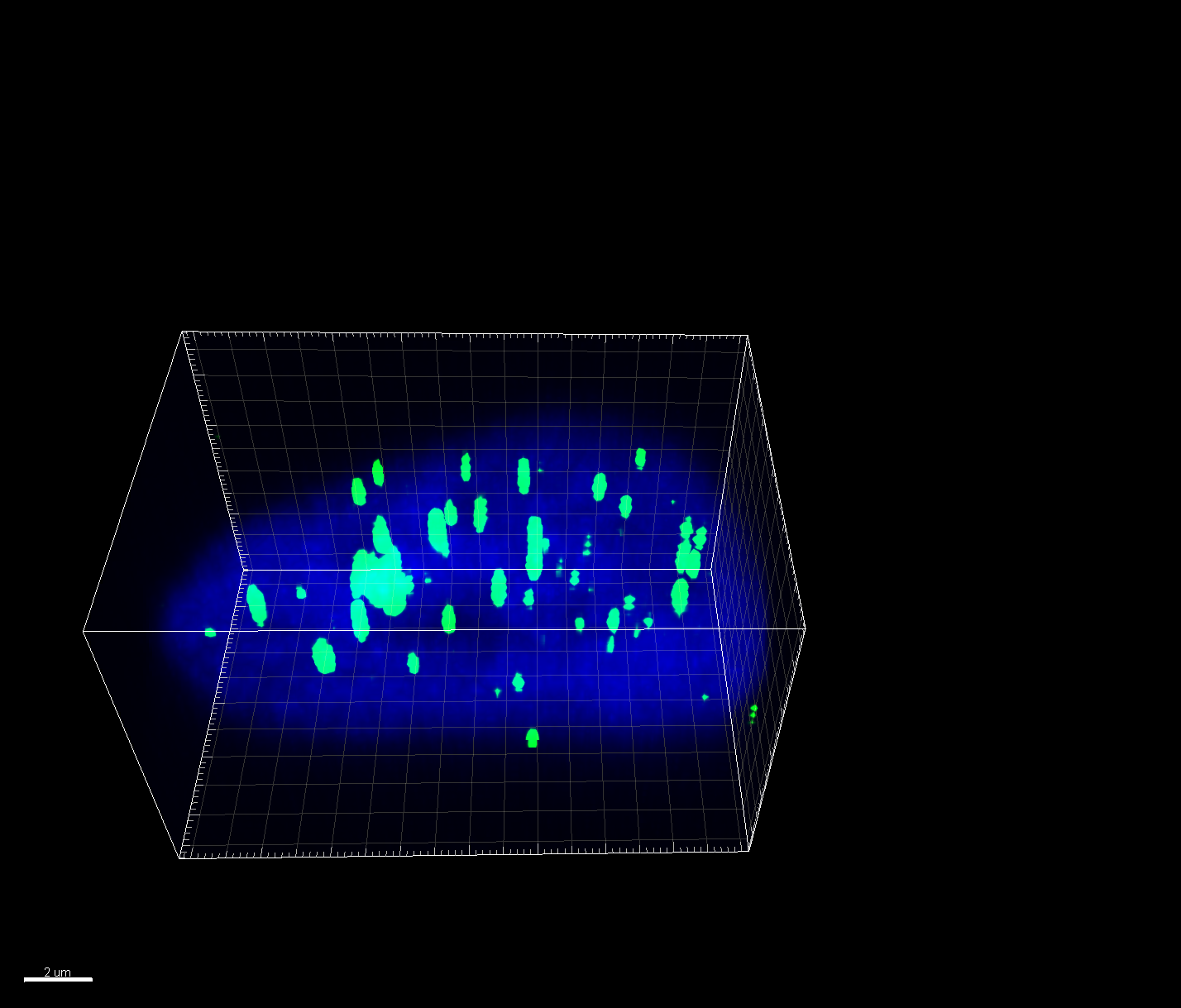

Supplement: Figure 1—source data 7. [file elife-85412-fig1-data7.zip › Fig 1- source data 7 Fig 1 G/6 hr (1).tif]

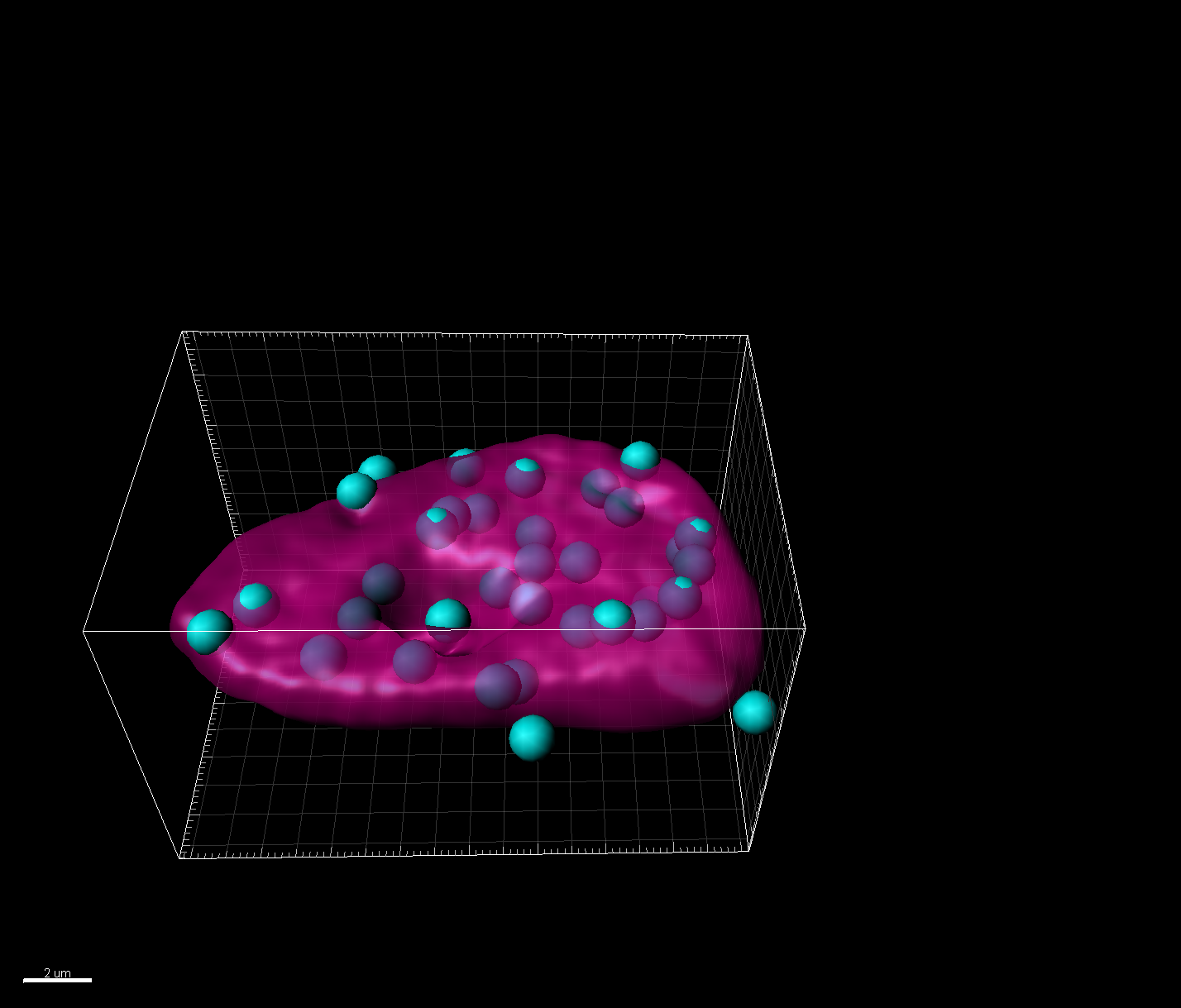

Supplement: Figure 1—source data 7. [file elife-85412-fig1-data7.zip › Fig 1- source data 7 Fig 1 G/6 hr (2).tif]

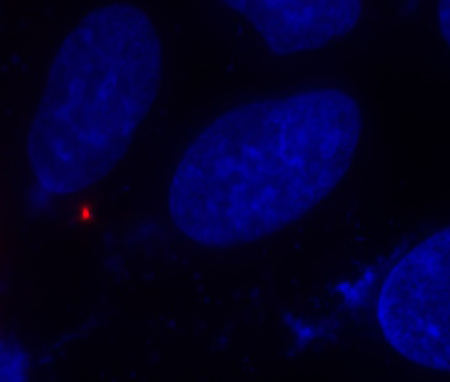

Supplement: Figure 1—figure supplement 1—source data 1. — This zip archive contains source data for western blot in panel B and C, original data collected for statistical analysis in panel G and I, and processed images (TIF files) in panel B, D, E, F, H, J, and K. Original fluorescence images and confocal images are deposited on DRYAD (https://doi.org/10.5061/dryad.vmcvdncxd). [file elife-85412-fig1-figsupp1-data1.zip › Figure 1-figure supplement 1-sourse data 1 Figure 1-figure supplement 1 B IF/Control.jpg]

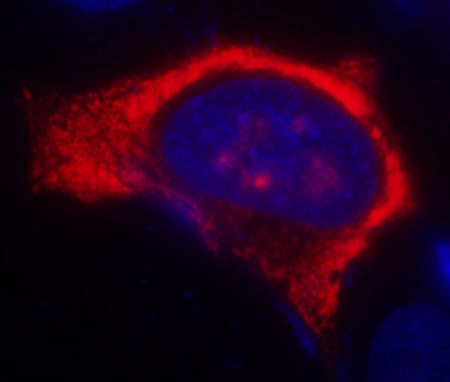

Supplement: Figure 1—figure supplement 1—source data 1. — This zip archive contains source data for western blot in panel B and C, original data collected for statistical analysis in panel G and I, and processed images (TIF files) in panel B, D, E, F, H, J, and K. Original fluorescence images and confocal images are deposited on DRYAD (https://doi.org/10.5061/dryad.vmcvdncxd). [file elife-85412-fig1-figsupp1-data1.zip › Figure 1-figure supplement 1-sourse data 1 Figure 1-figure supplement 1 B IF/Flag-dcas9.jpg]

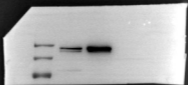

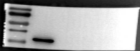

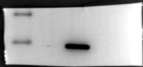

Supplement: Figure 1—figure supplement 1—source data 2. [file elife-85412-fig1-figsupp1-data2.pdf]

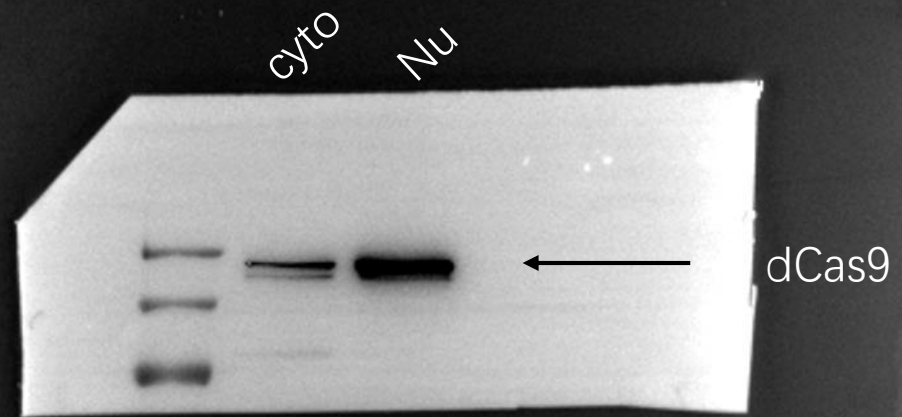

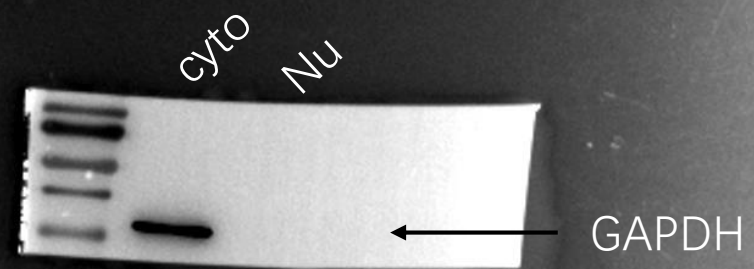

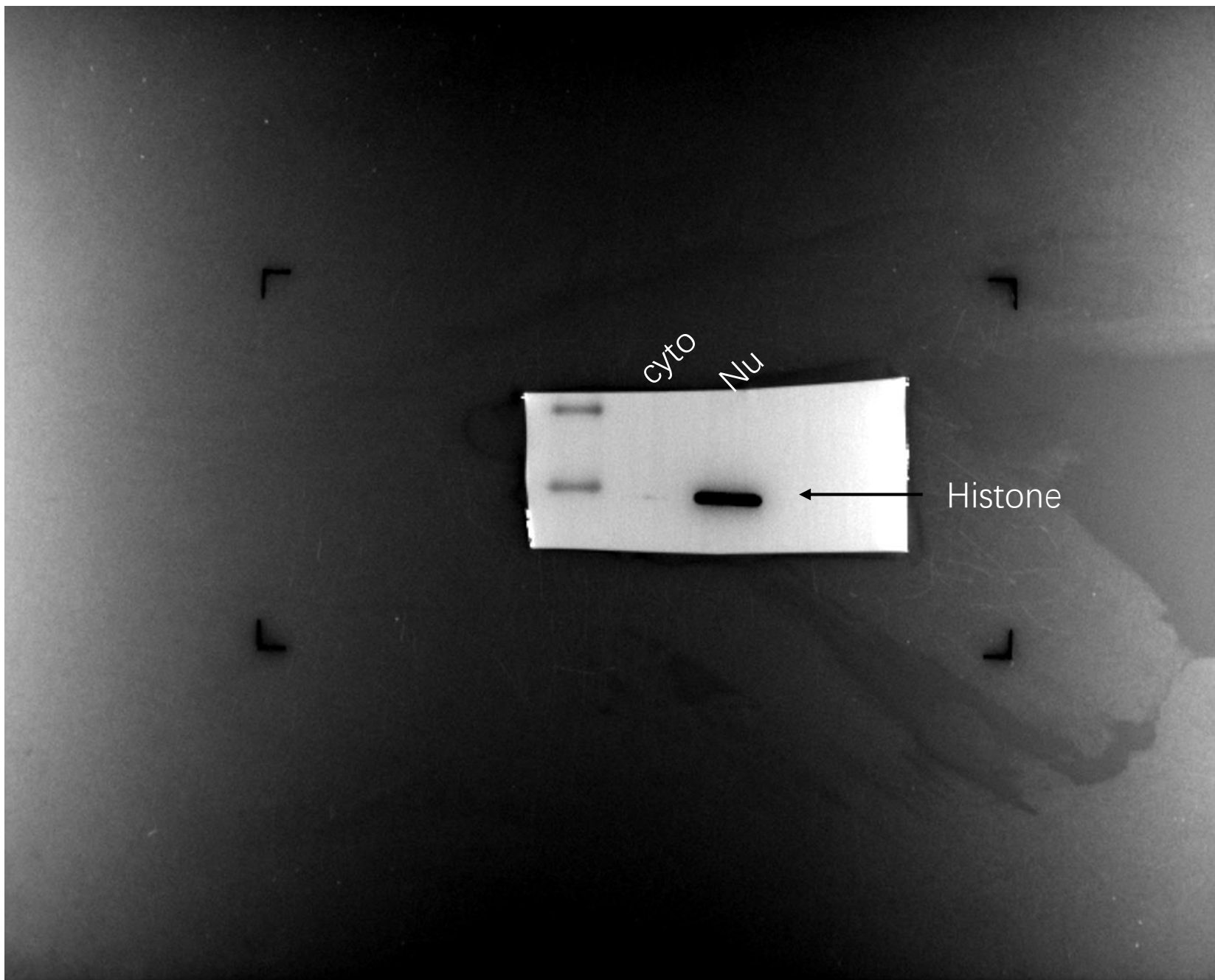

Supplement: Figure 1—figure supplement 1—source data 3. [file elife-85412-fig1-figsupp1-data3.pdf]

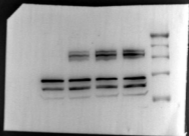

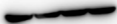

Supplement: Figure 1—figure supplement 1—source data 4. [file elife-85412-fig1-figsupp1-data4.pdf]

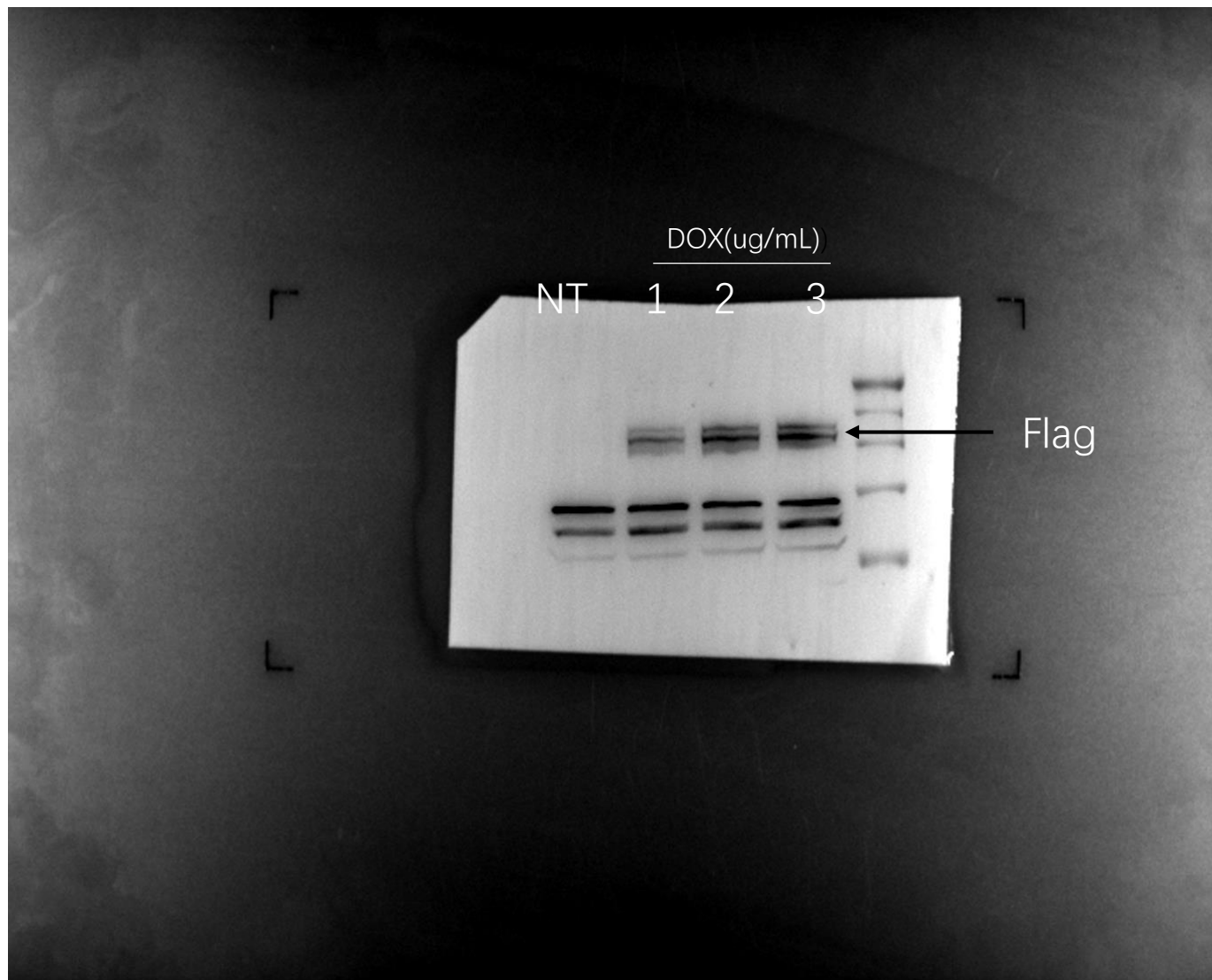

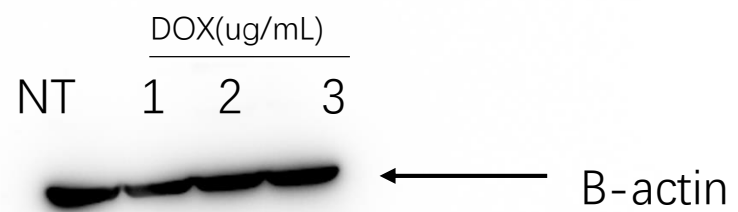

Supplement: Figure 1—figure supplement 1—source data 5. [file elife-85412-fig1-figsupp1-data5.pdf]

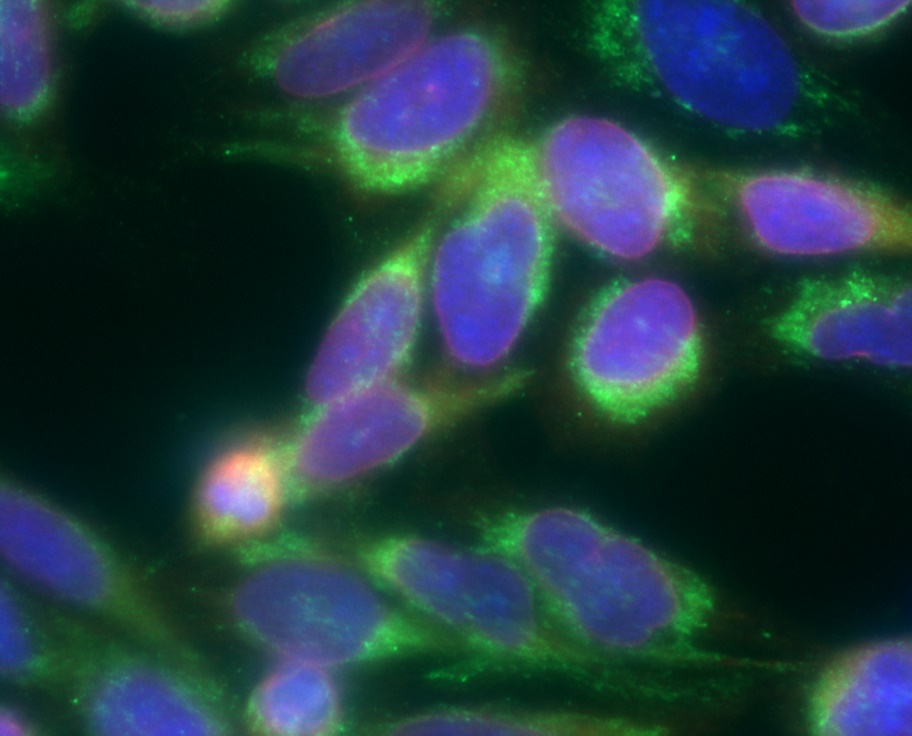

Supplement: Figure 1—figure supplement 1—source data 6. [file elife-85412-fig1-figsupp1-data6.zip › Figure 1-figure supplement 1- source data 6 Figure 1-figure supplement 1 D/calnexin emerin_-2__c1-3.jpg]

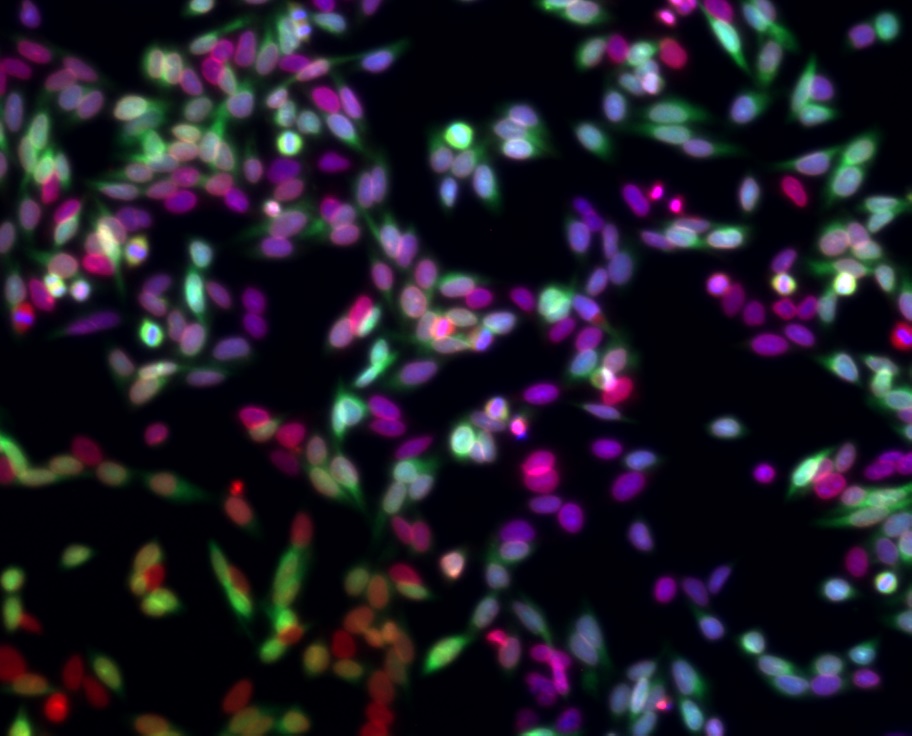

Supplement: Figure 1—figure supplement 1—source data 7. [file elife-85412-fig1-figsupp1-data7.zip › Figure 1-figure supplement 1-source data 7 Figure 1-figure supplement 1 E/+DOX_c1-3.jpg]

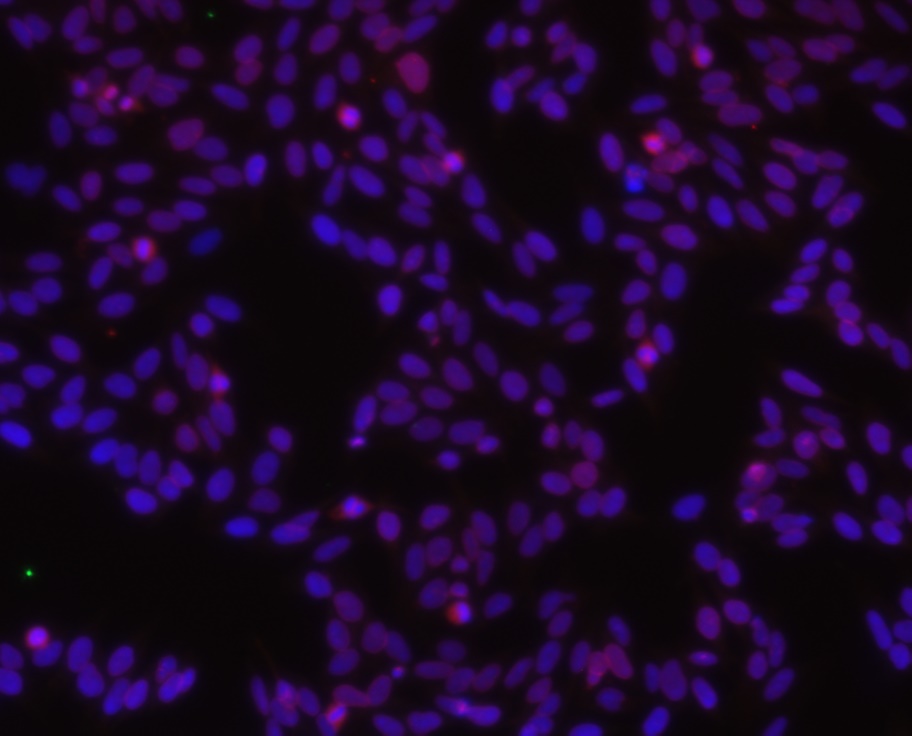

Supplement: Figure 1—figure supplement 1—source data 7. [file elife-85412-fig1-figsupp1-data7.zip › Figure 1-figure supplement 1-source data 7 Figure 1-figure supplement 1 E/-DOX__c1-3.jpg]

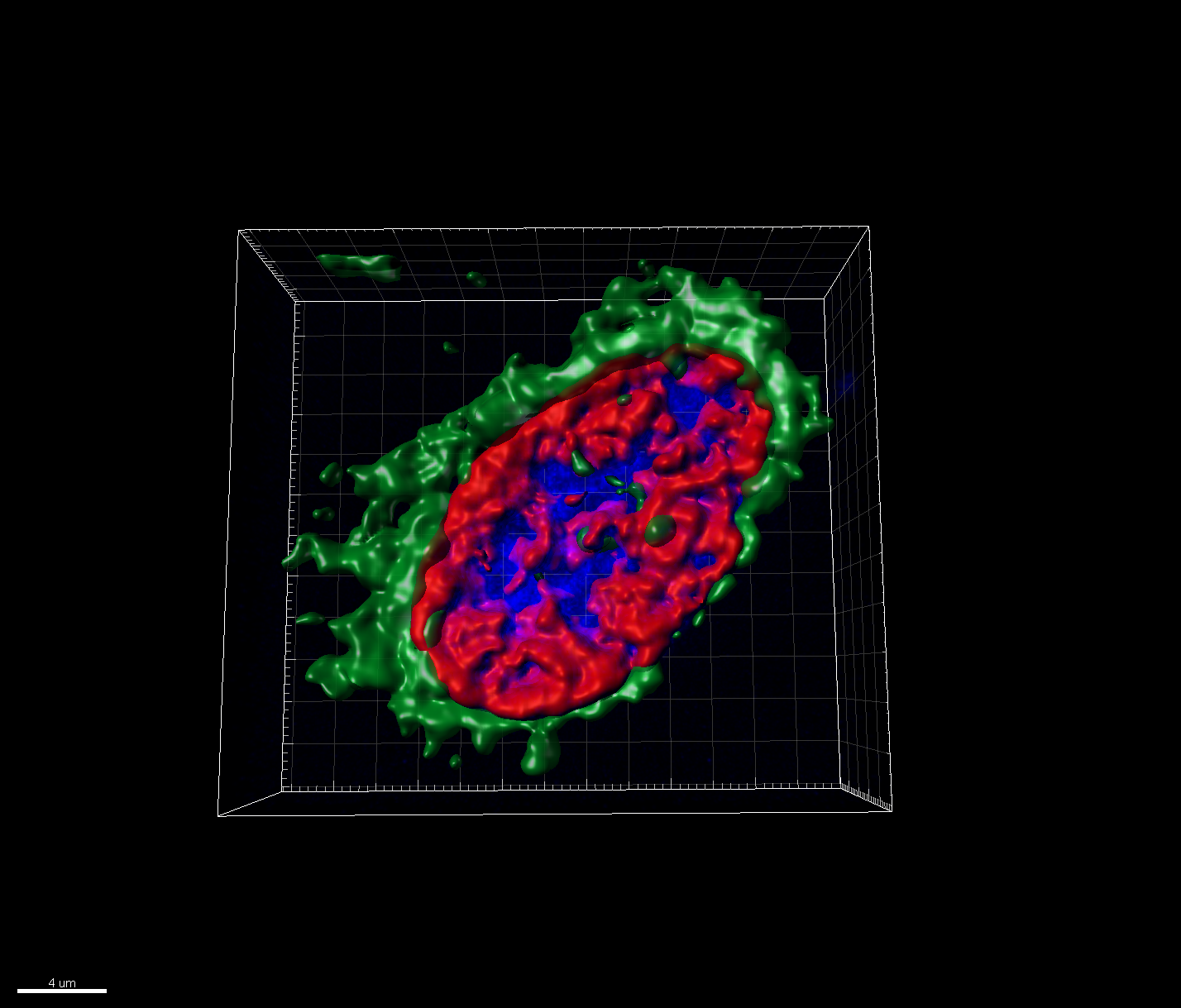

Supplement: Figure 1—figure supplement 1—source data 8. [file elife-85412-fig1-figsupp1-data8.zip › Figure 1-figure supplement 1- source data 8 Figure 1-figure supplement 1 F/Sup Fig 1 F upper panel/3D reconstruction.tif]

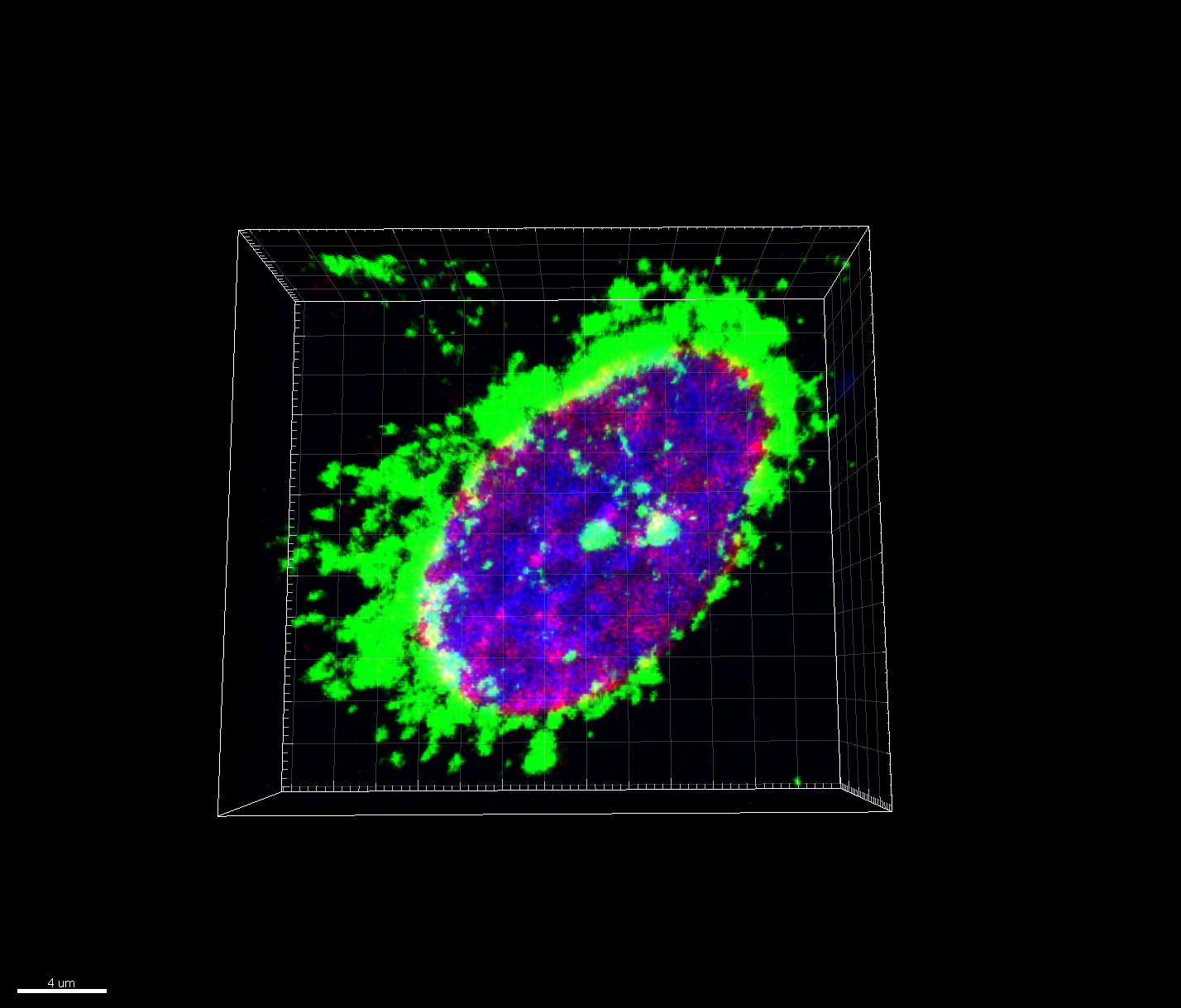

Supplement: Figure 1—figure supplement 1—source data 8. [file elife-85412-fig1-figsupp1-data8.zip › Figure 1-figure supplement 1- source data 8 Figure 1-figure supplement 1 F/Sup Fig 1 F upper panel/confocal .tif]

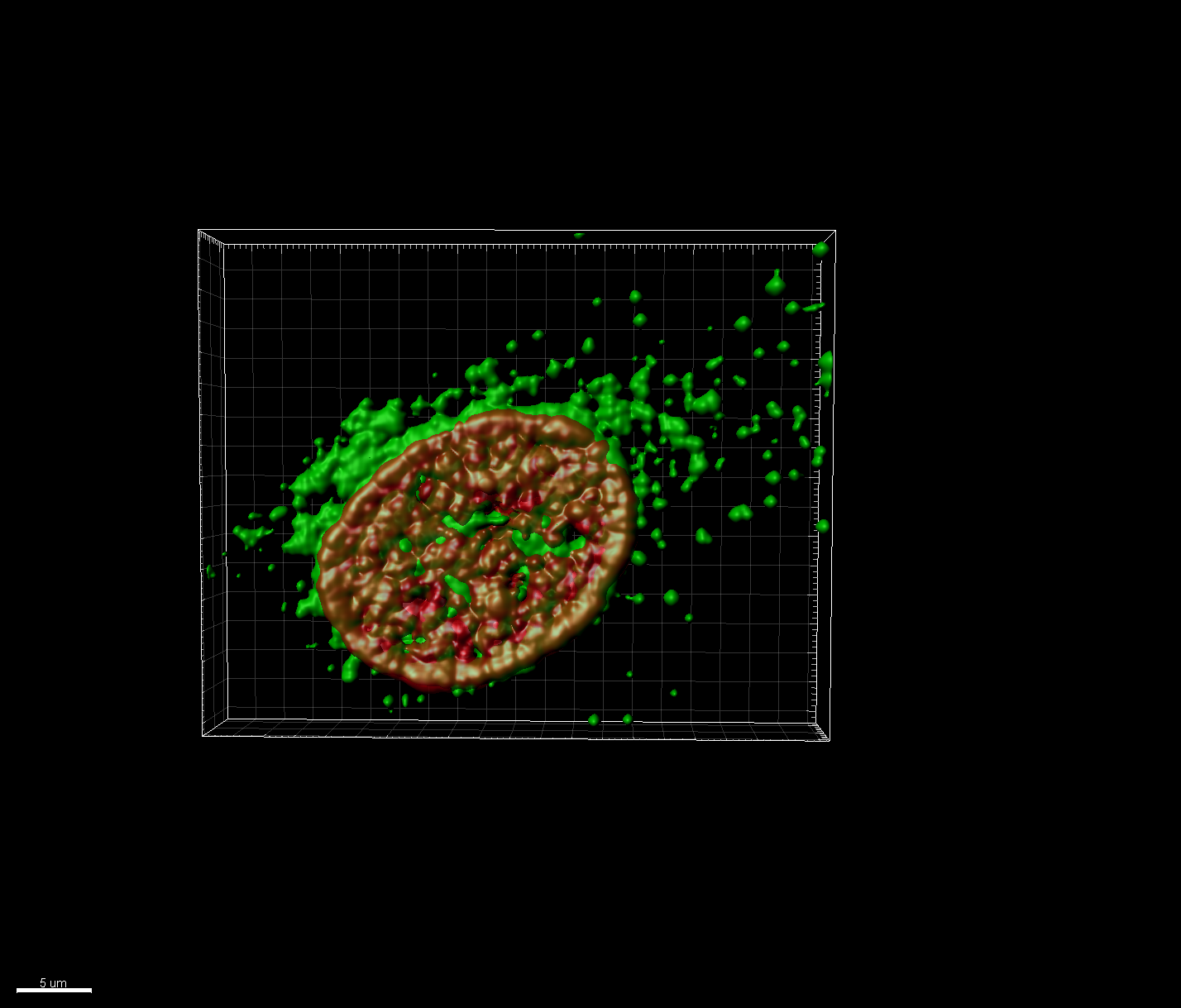

Supplement: Figure 1—figure supplement 1—source data 8. [file elife-85412-fig1-figsupp1-data8.zip › Figure 1-figure supplement 1- source data 8 Figure 1-figure supplement 1 F/Sup Fig 1 lower panel/0028_2022-04-25T16-44-43.693.tif]

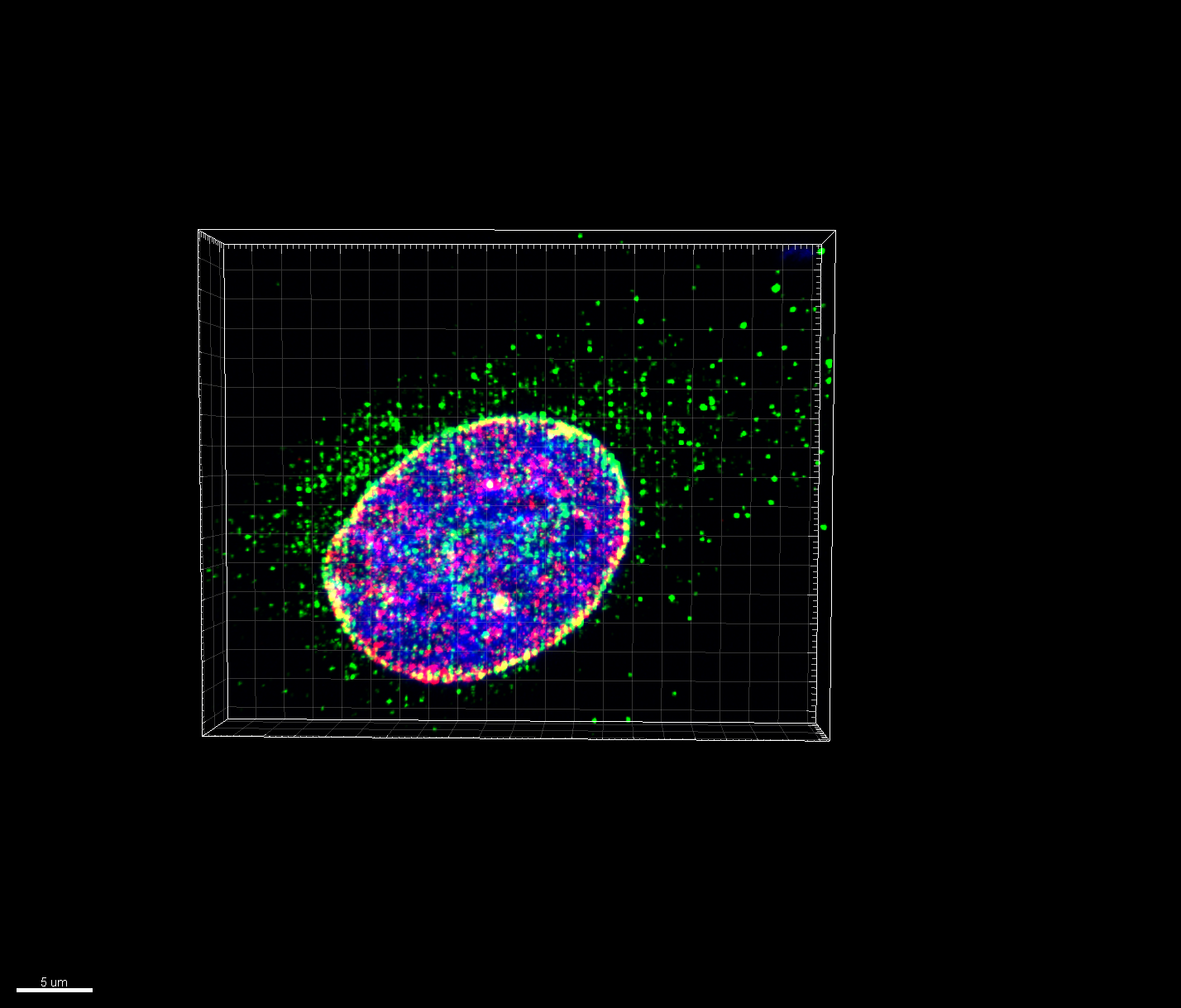

Supplement: Figure 1—figure supplement 1—source data 8. [file elife-85412-fig1-figsupp1-data8.zip › Figure 1-figure supplement 1- source data 8 Figure 1-figure supplement 1 F/Sup Fig 1 lower panel/0028_2022-04-25T16-45-01.152.tif]

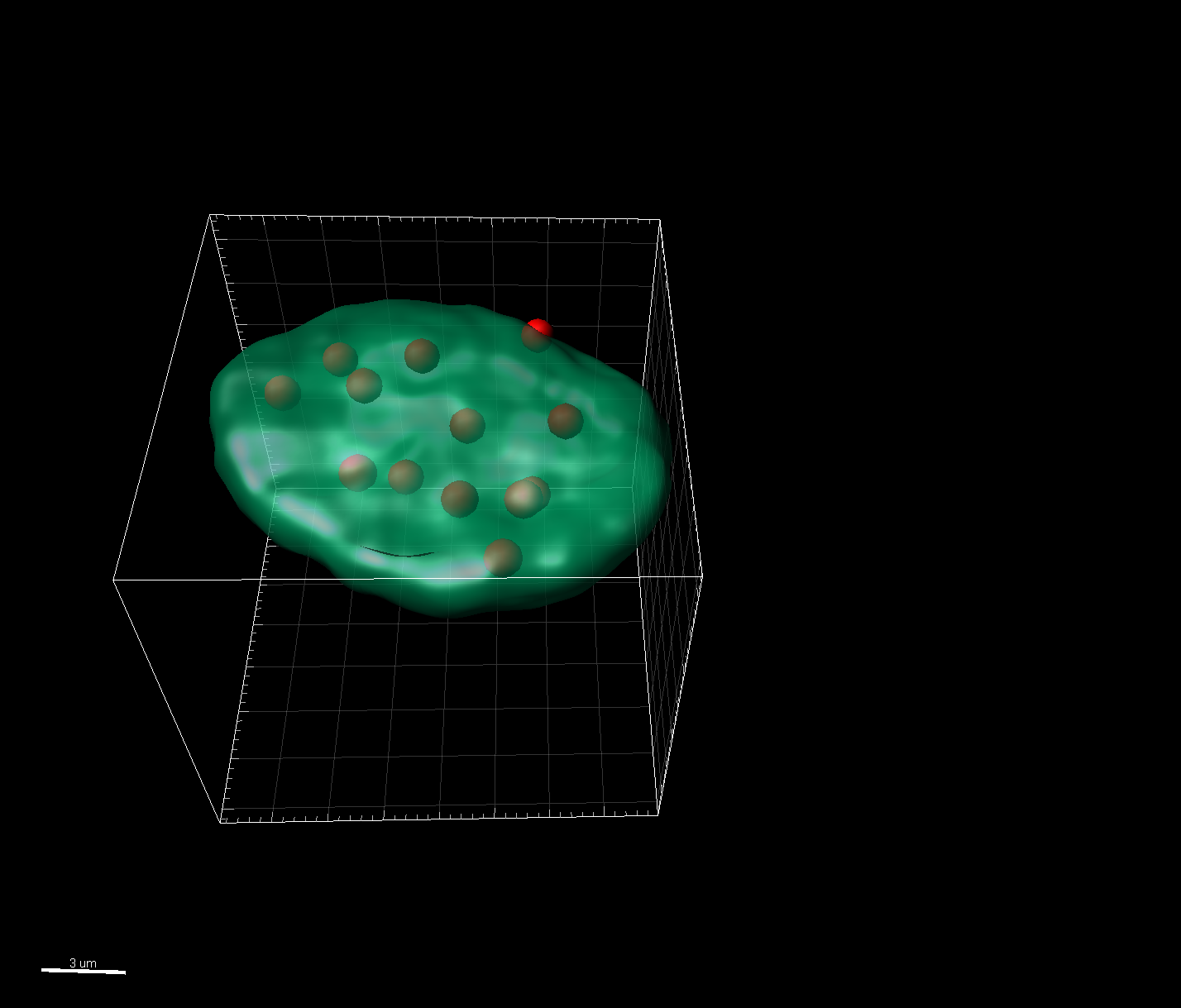

Supplement: Figure 1—figure supplement 1—source data 10. [file elife-85412-fig1-figsupp1-data10.zip › Figure 1-figure supplement 1-source data 10 Figure 1-figure supplement 1 H/plasmid 0s 3d.tif]

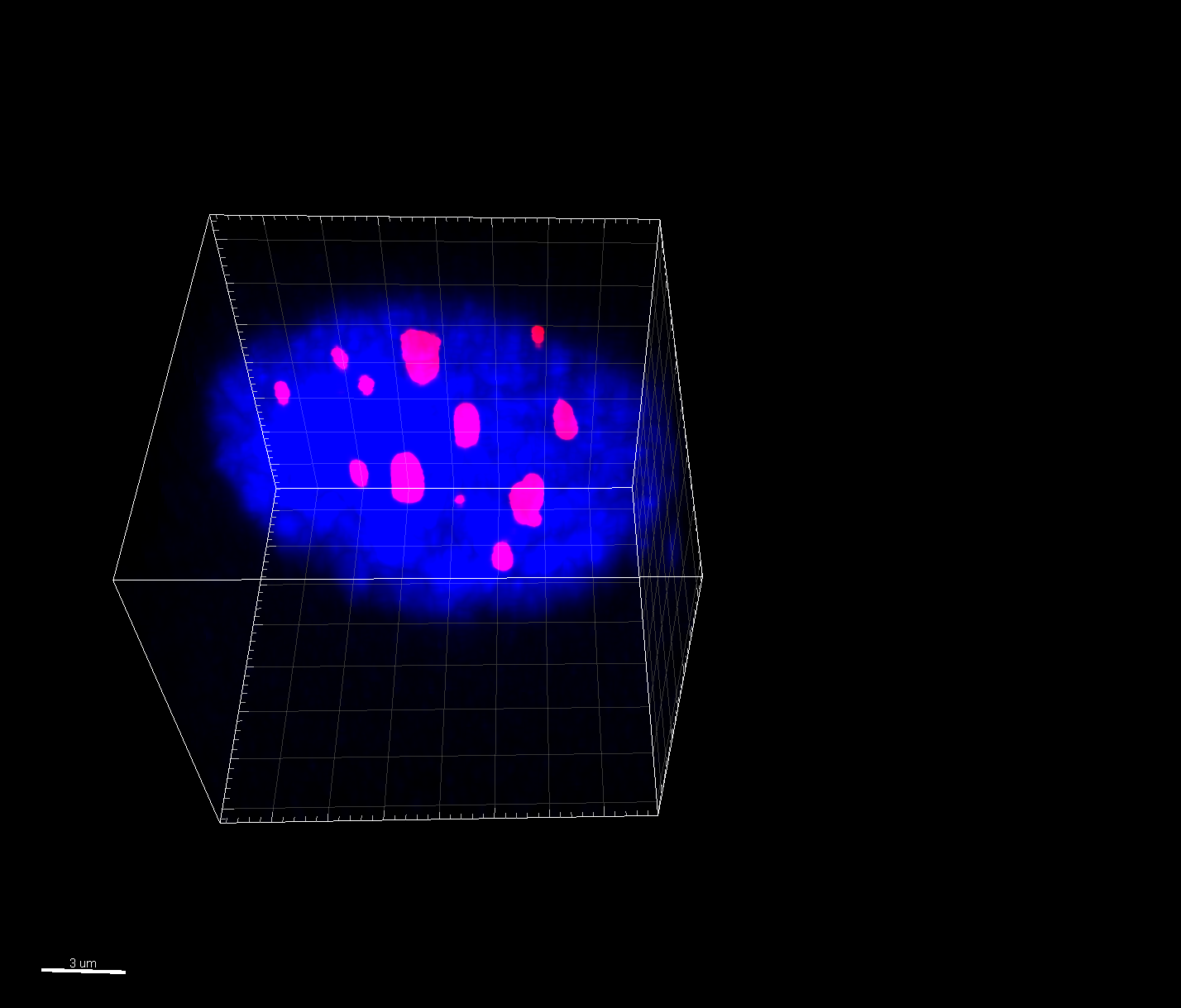

Supplement: Figure 1—figure supplement 1—source data 10. [file elife-85412-fig1-figsupp1-data10.zip › Figure 1-figure supplement 1-source data 10 Figure 1-figure supplement 1 H/plasmid 0s.tif]

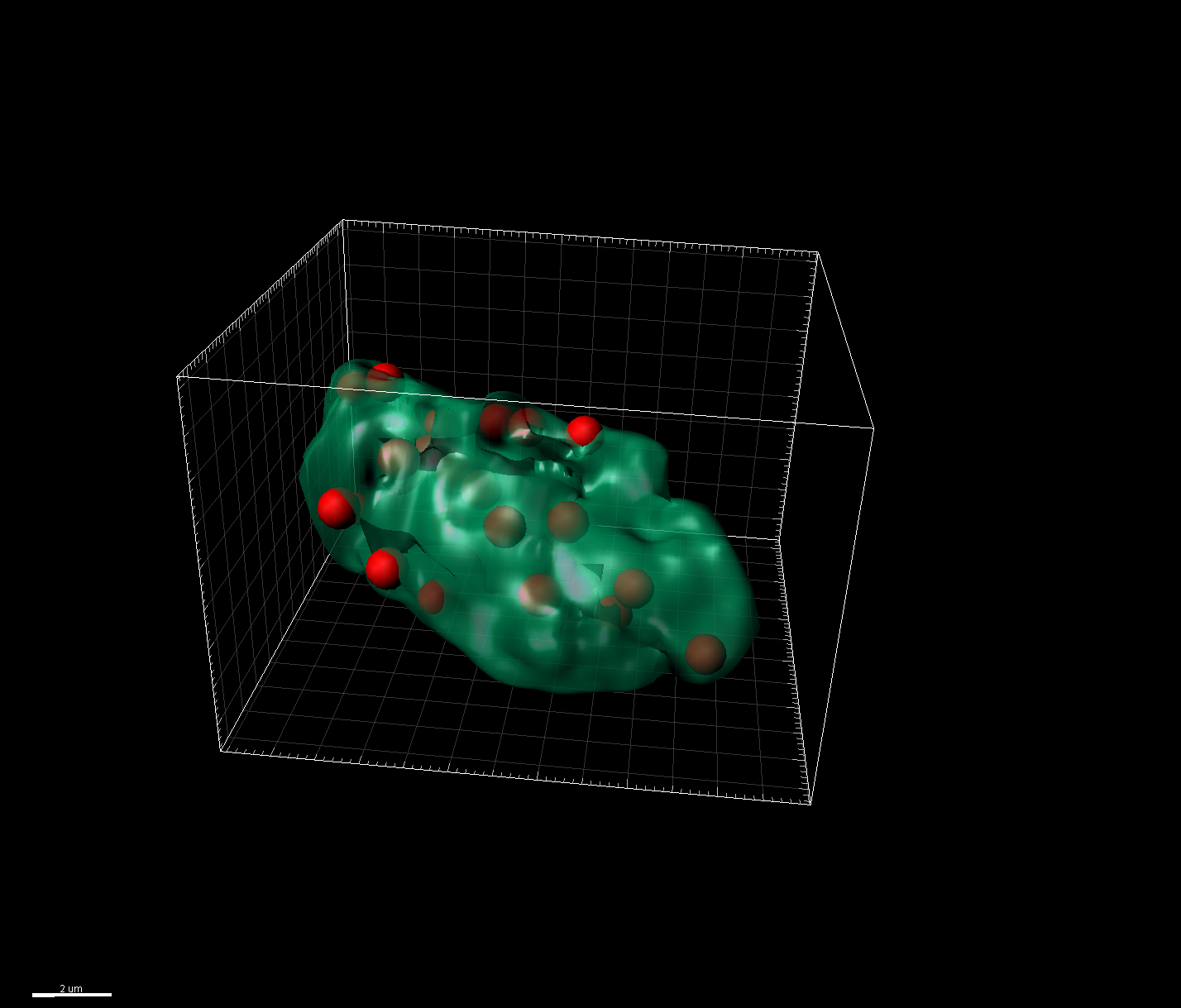

Supplement: Figure 1—figure supplement 1—source data 10. [file elife-85412-fig1-figsupp1-data10.zip › Figure 1-figure supplement 1-source data 10 Figure 1-figure supplement 1 H/plasmid 10s 3D.tif]

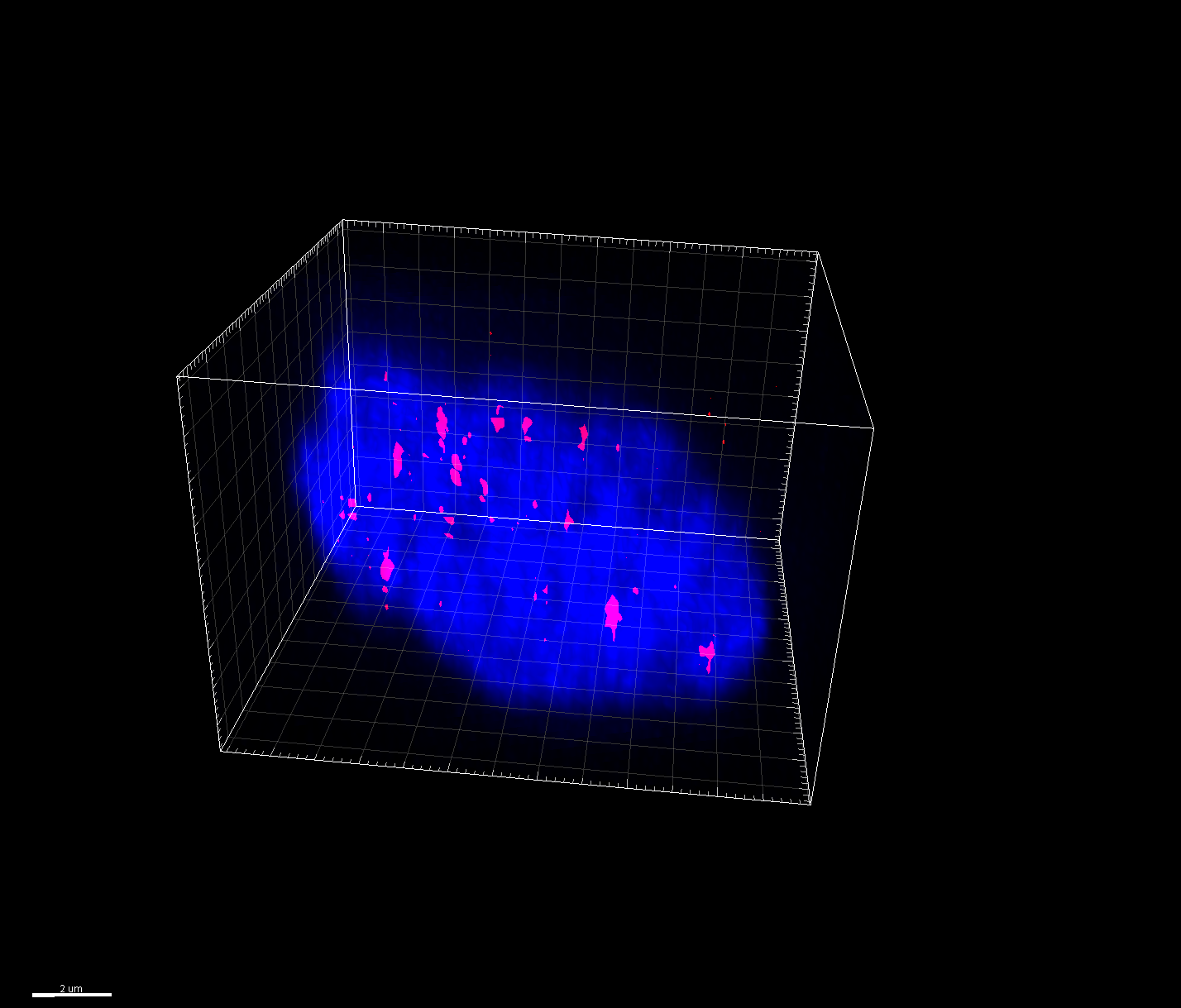

Supplement: Figure 1—figure supplement 1—source data 10. [file elife-85412-fig1-figsupp1-data10.zip › Figure 1-figure supplement 1-source data 10 Figure 1-figure supplement 1 H/plasmid 10s.tif]

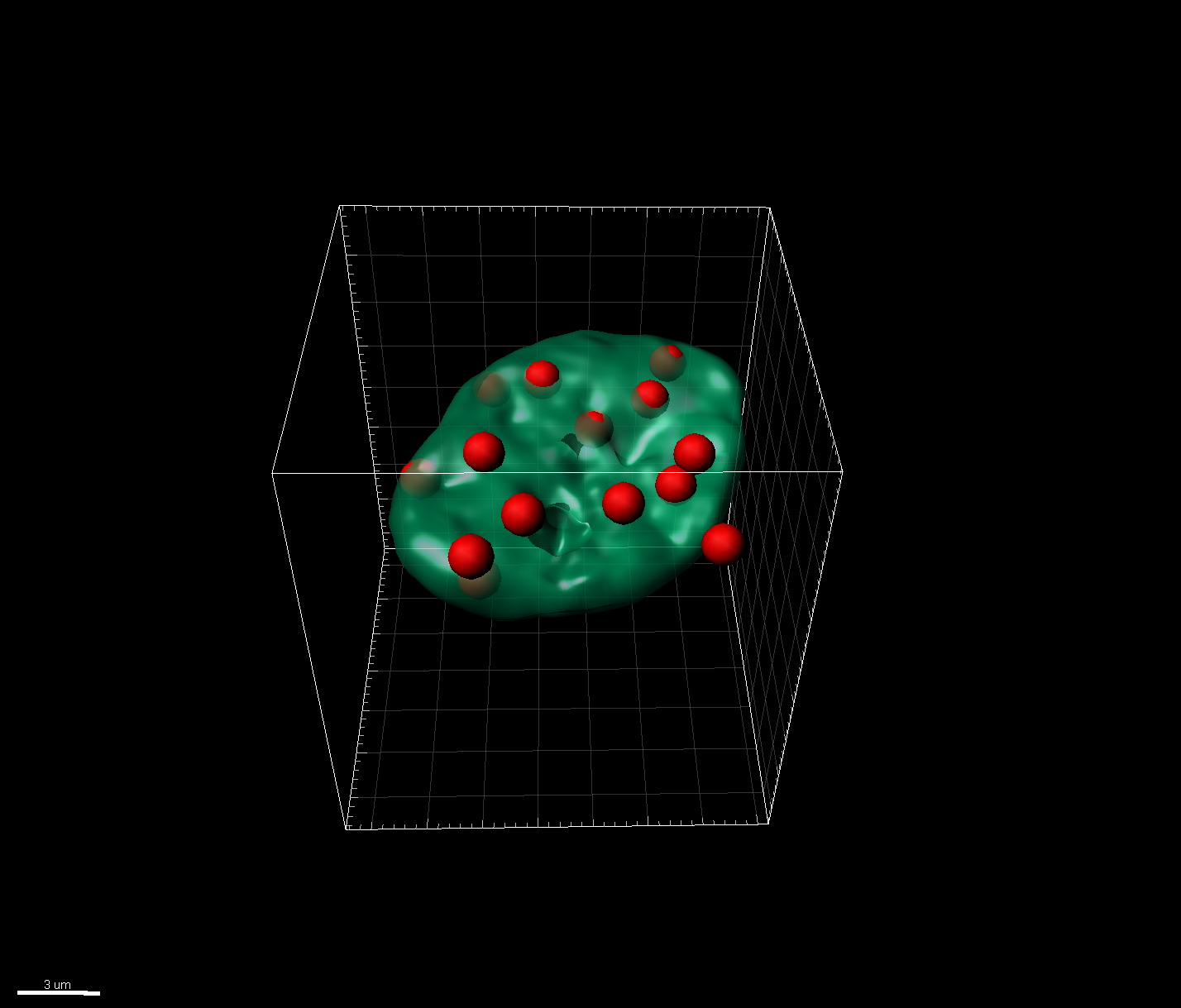

Supplement: Figure 1—figure supplement 1—source data 10. [file elife-85412-fig1-figsupp1-data10.zip › Figure 1-figure supplement 1-source data 10 Figure 1-figure supplement 1 H/plasmid 180s 3D.tif]

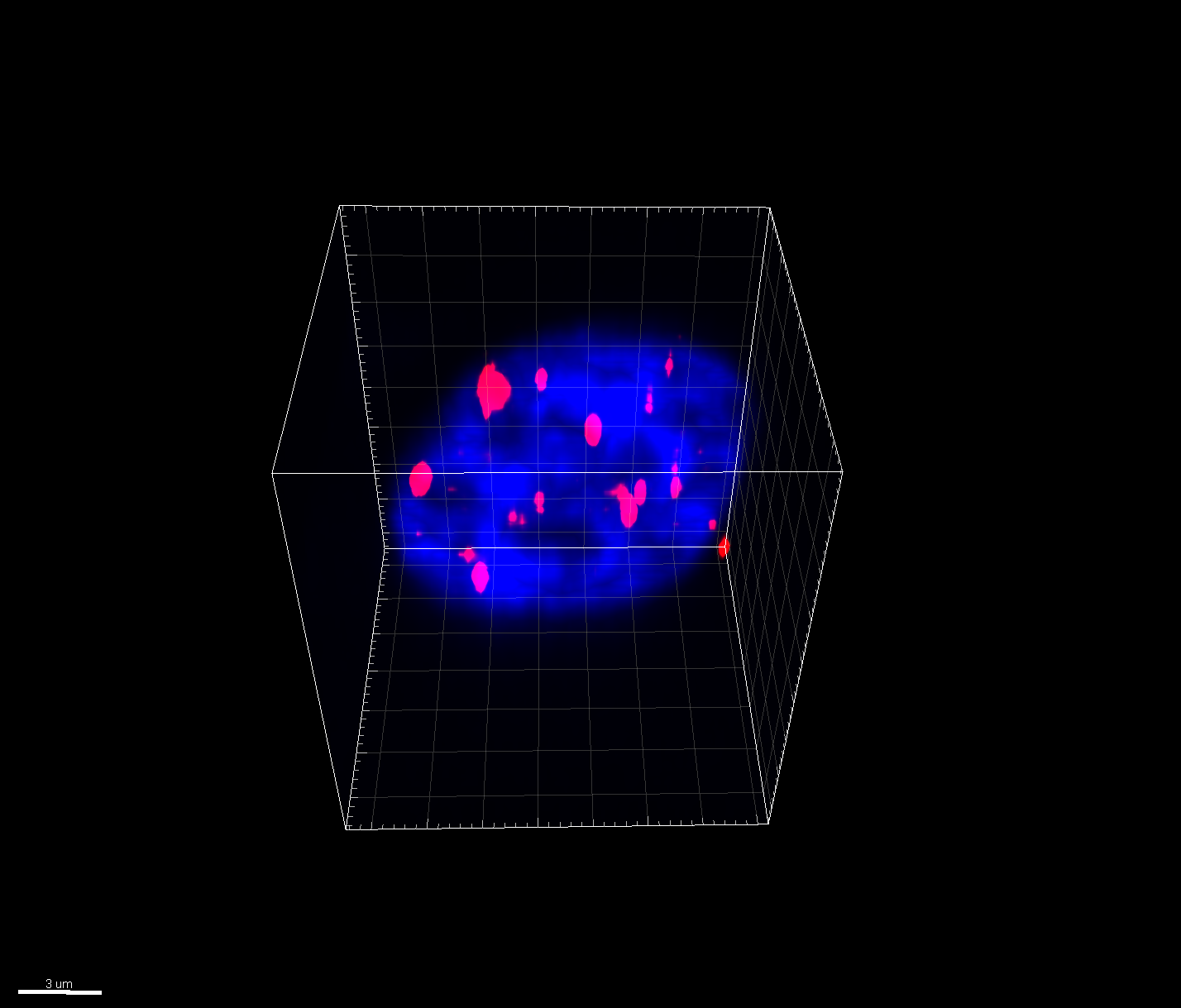

Supplement: Figure 1—figure supplement 1—source data 10. [file elife-85412-fig1-figsupp1-data10.zip › Figure 1-figure supplement 1-source data 10 Figure 1-figure supplement 1 H/plasmid 180s.tif]

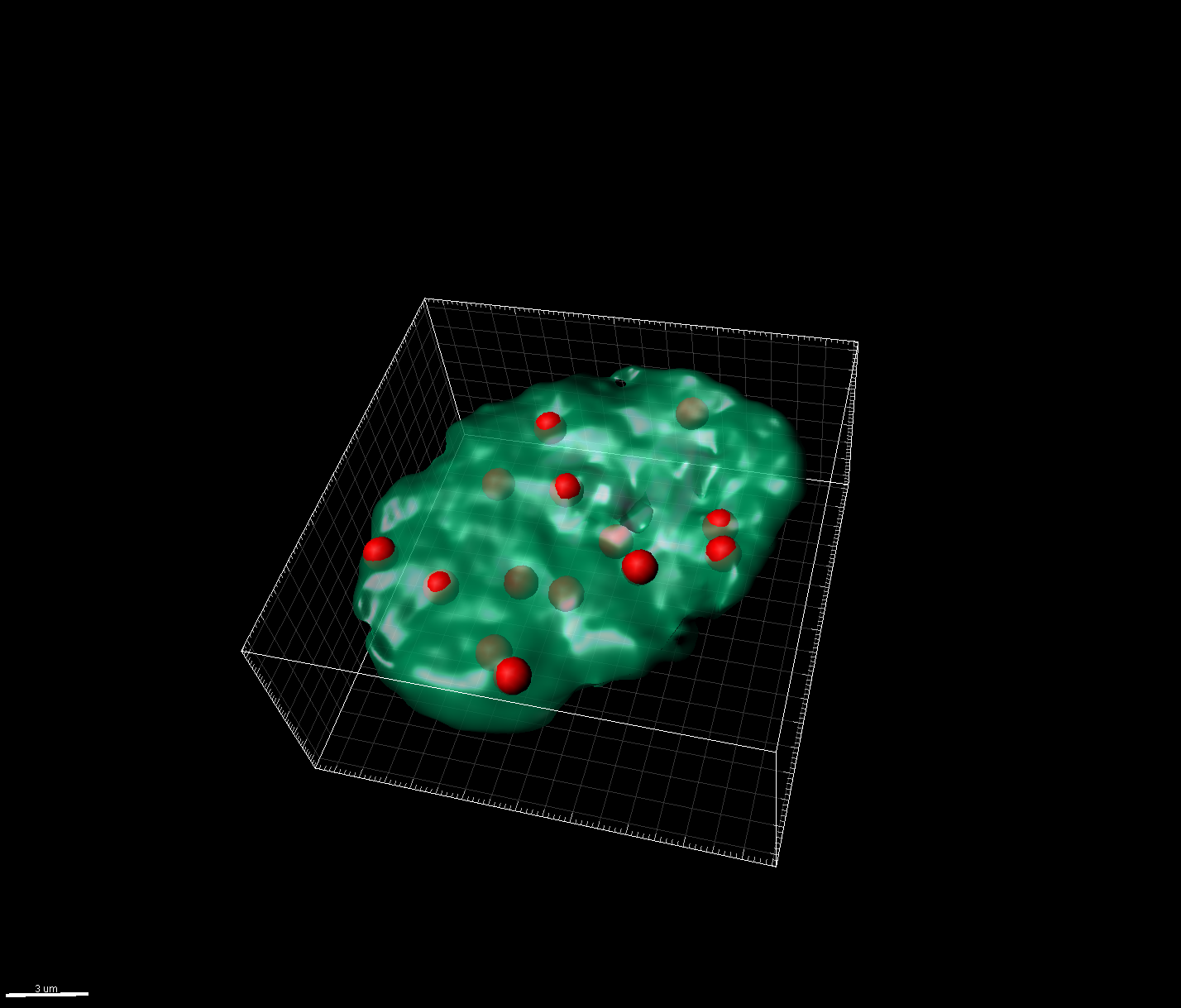

Supplement: Figure 1—figure supplement 1—source data 10. [file elife-85412-fig1-figsupp1-data10.zip › Figure 1-figure supplement 1-source data 10 Figure 1-figure supplement 1 H/plasmid 20 3D.tif]

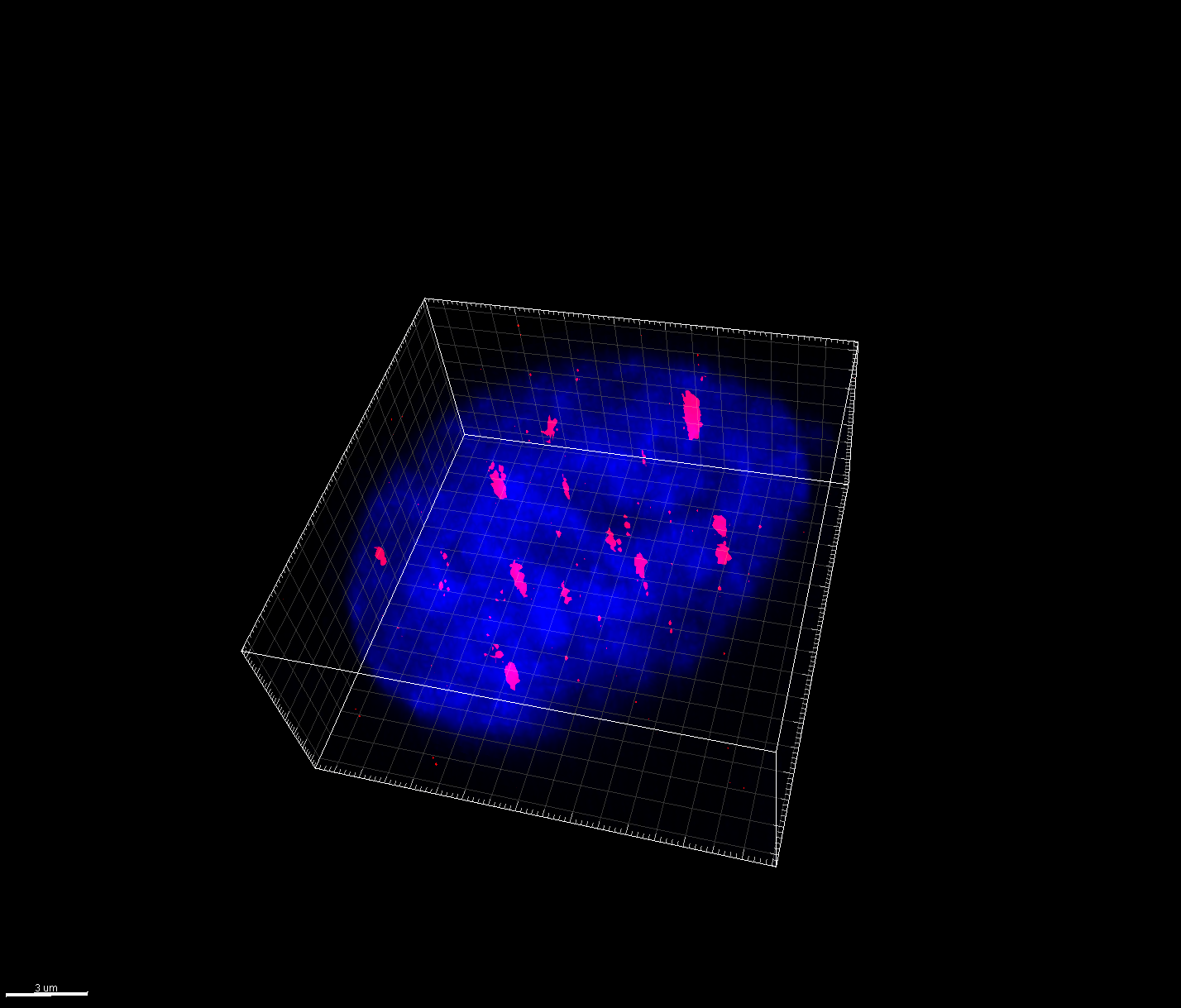

Supplement: Figure 1—figure supplement 1—source data 10. [file elife-85412-fig1-figsupp1-data10.zip › Figure 1-figure supplement 1-source data 10 Figure 1-figure supplement 1 H/plasmid 20s.tif]

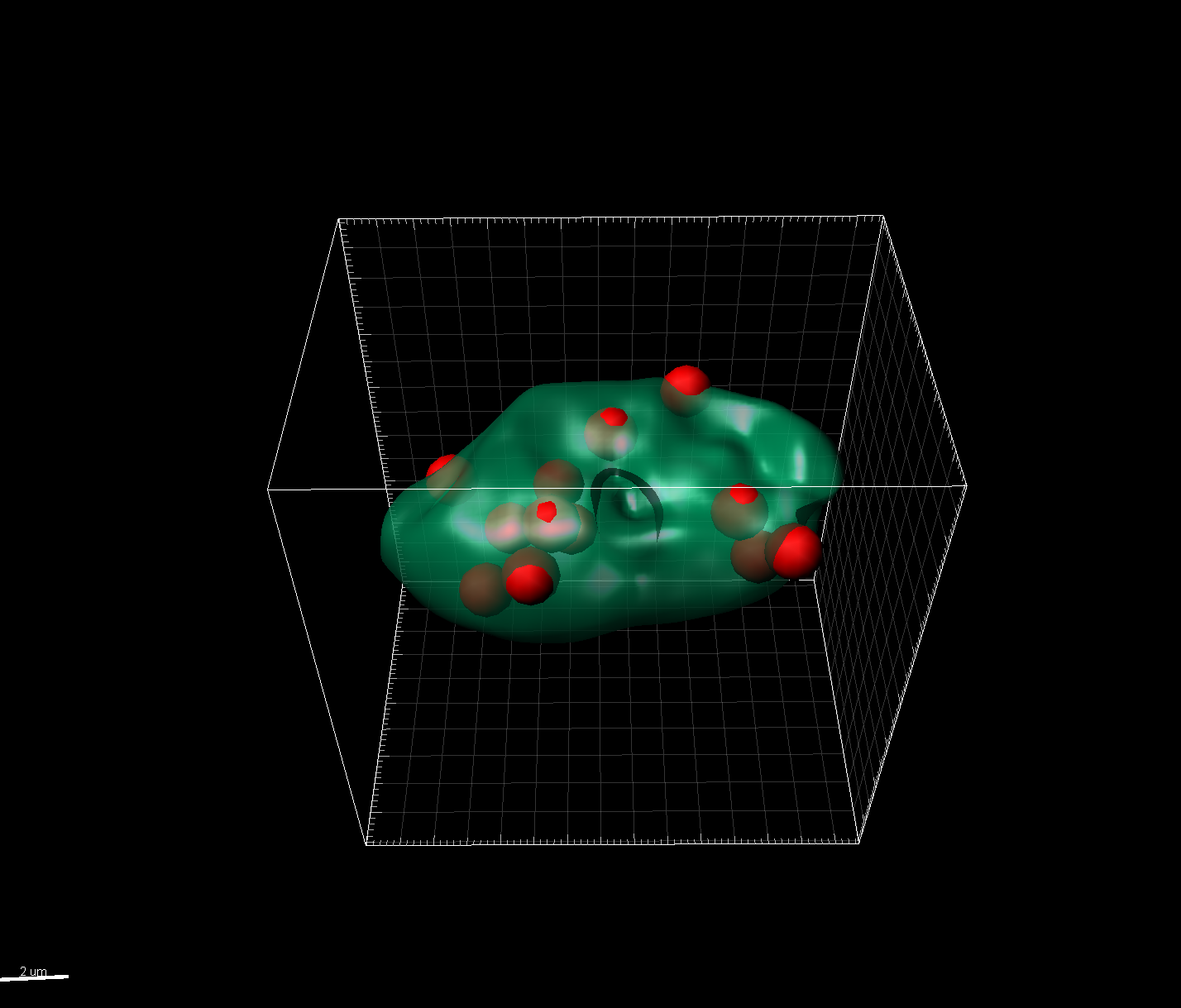

Supplement: Figure 1—figure supplement 1—source data 10. [file elife-85412-fig1-figsupp1-data10.zip › Figure 1-figure supplement 1-source data 10 Figure 1-figure supplement 1 H/plasmid 40s 3D.tif]

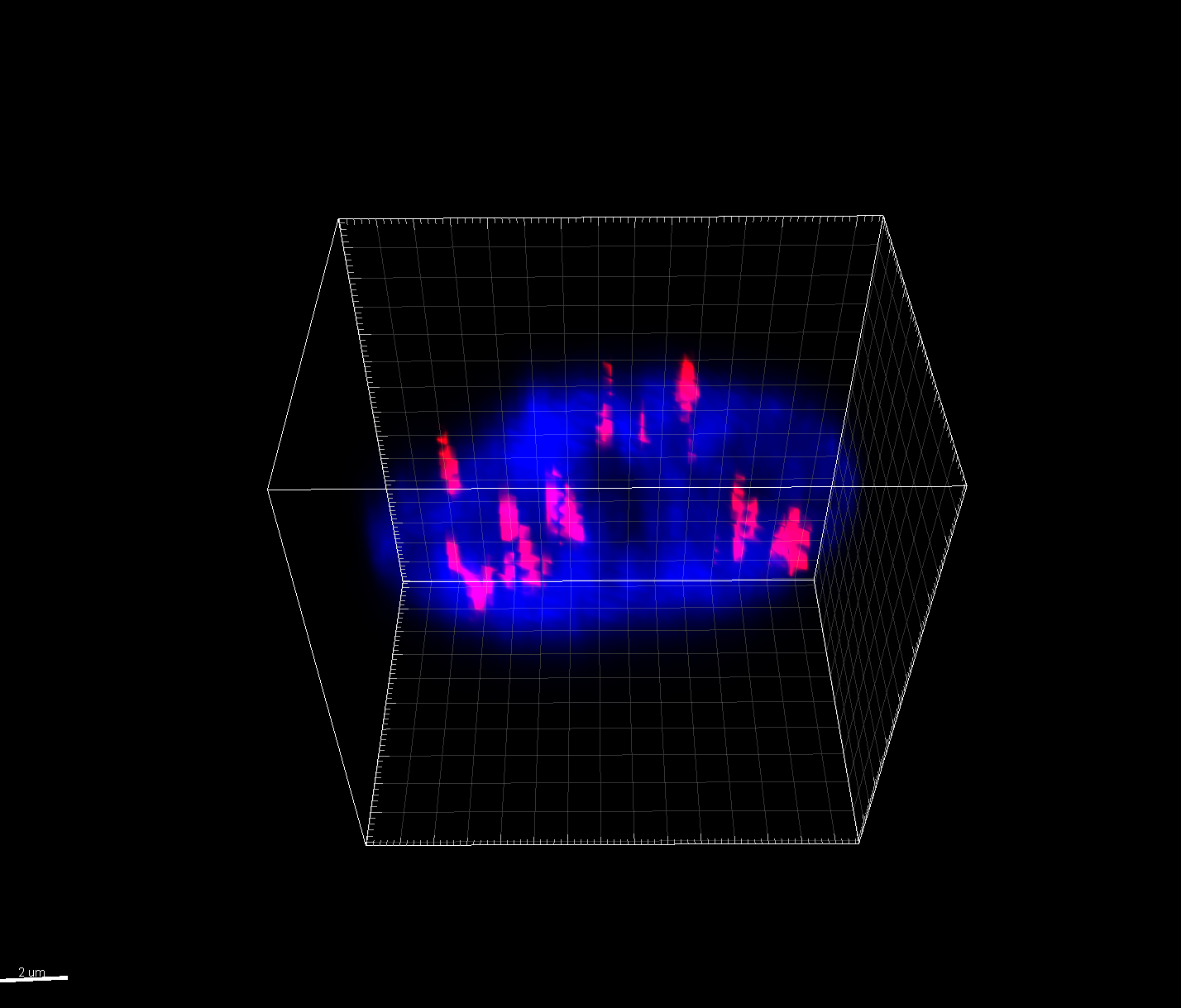

Supplement: Figure 1—figure supplement 1—source data 10. [file elife-85412-fig1-figsupp1-data10.zip › Figure 1-figure supplement 1-source data 10 Figure 1-figure supplement 1 H/plasmid 40s.tif]

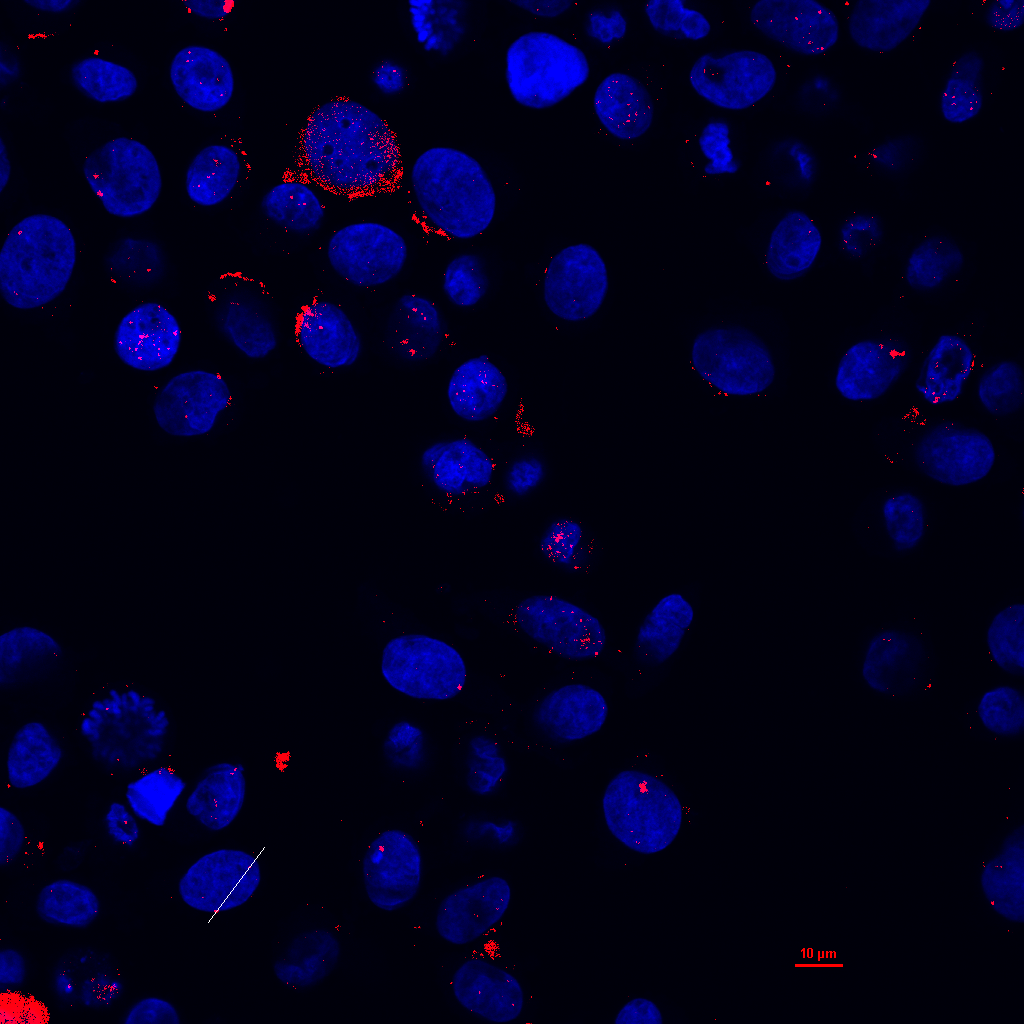

Supplement: Figure 1—figure supplement 1—source data 11. [file elife-85412-fig1-figsupp1-data11.zip › Figure 1-figure supplement 1- source data 11 Figure 1-figure supplement 1 J/bac-hsv-1-10s-1004z06.tif]

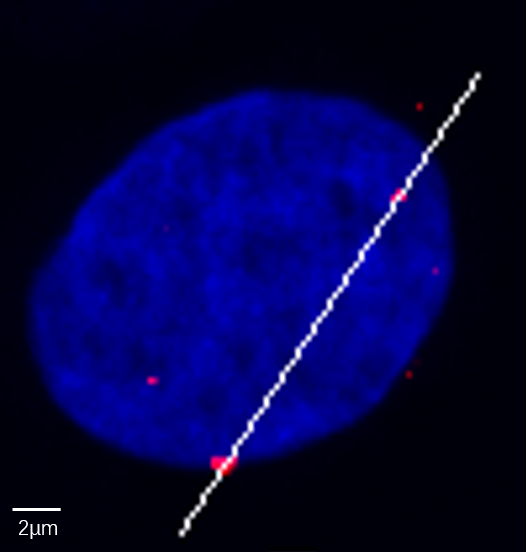

Supplement: Figure 1—figure supplement 1—source data 11. [file elife-85412-fig1-figsupp1-data11.zip › Figure 1-figure supplement 1- source data 11 Figure 1-figure supplement 1 J/used image.tif]

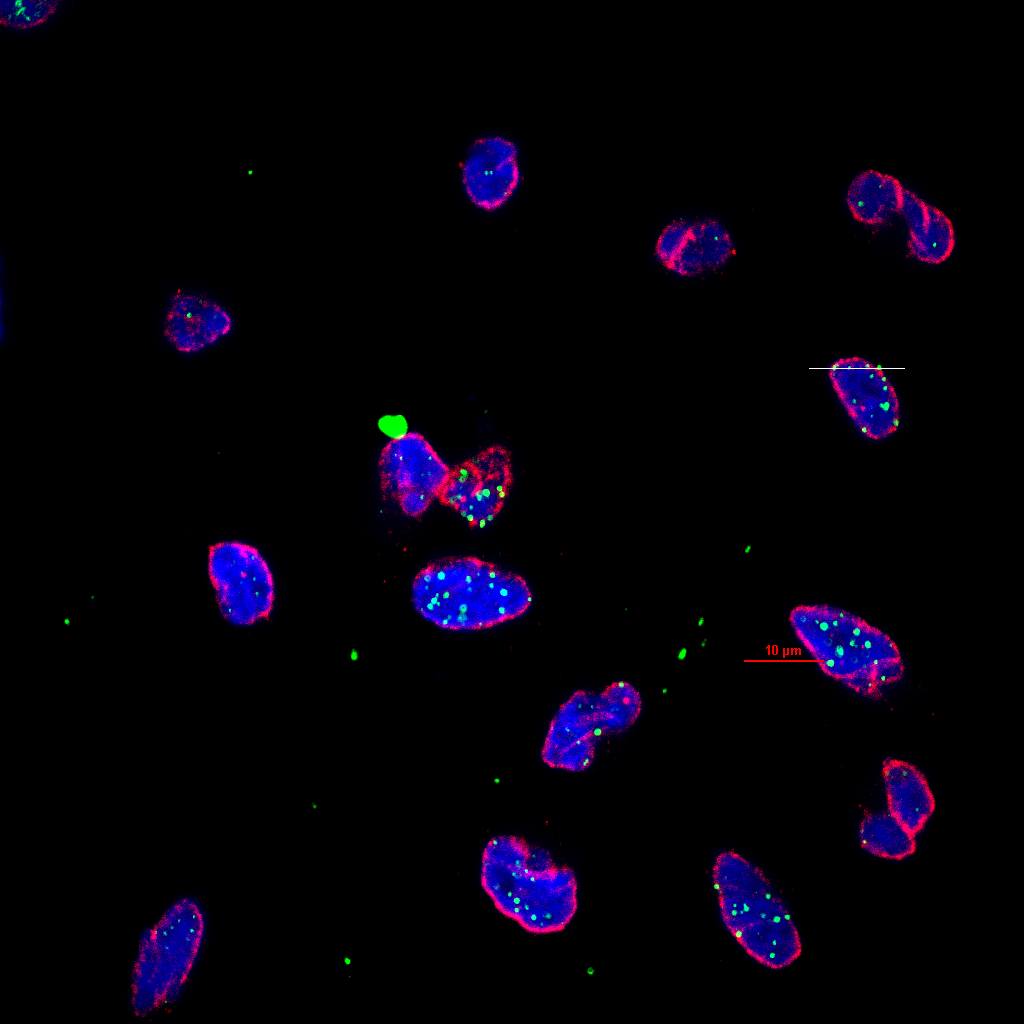

Supplement: Figure 1—figure supplement 1—source data 12. [file elife-85412-fig1-figsupp1-data12.zip › Figure 1-figure supplement 1- source data 12 Figure 1-figure supplement 1 K/Tel.tif]

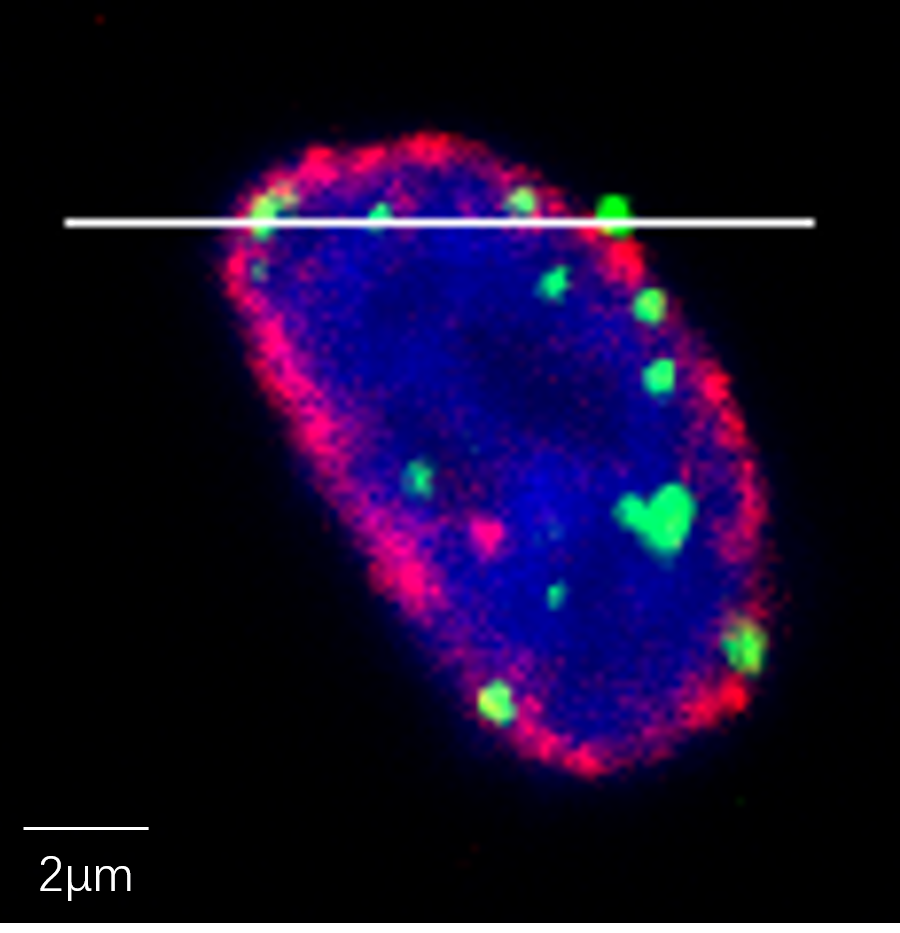

Supplement: Figure 1—figure supplement 1—source data 12. [file elife-85412-fig1-figsupp1-data12.zip › Figure 1-figure supplement 1- source data 12 Figure 1-figure supplement 1 K/used image.tif]

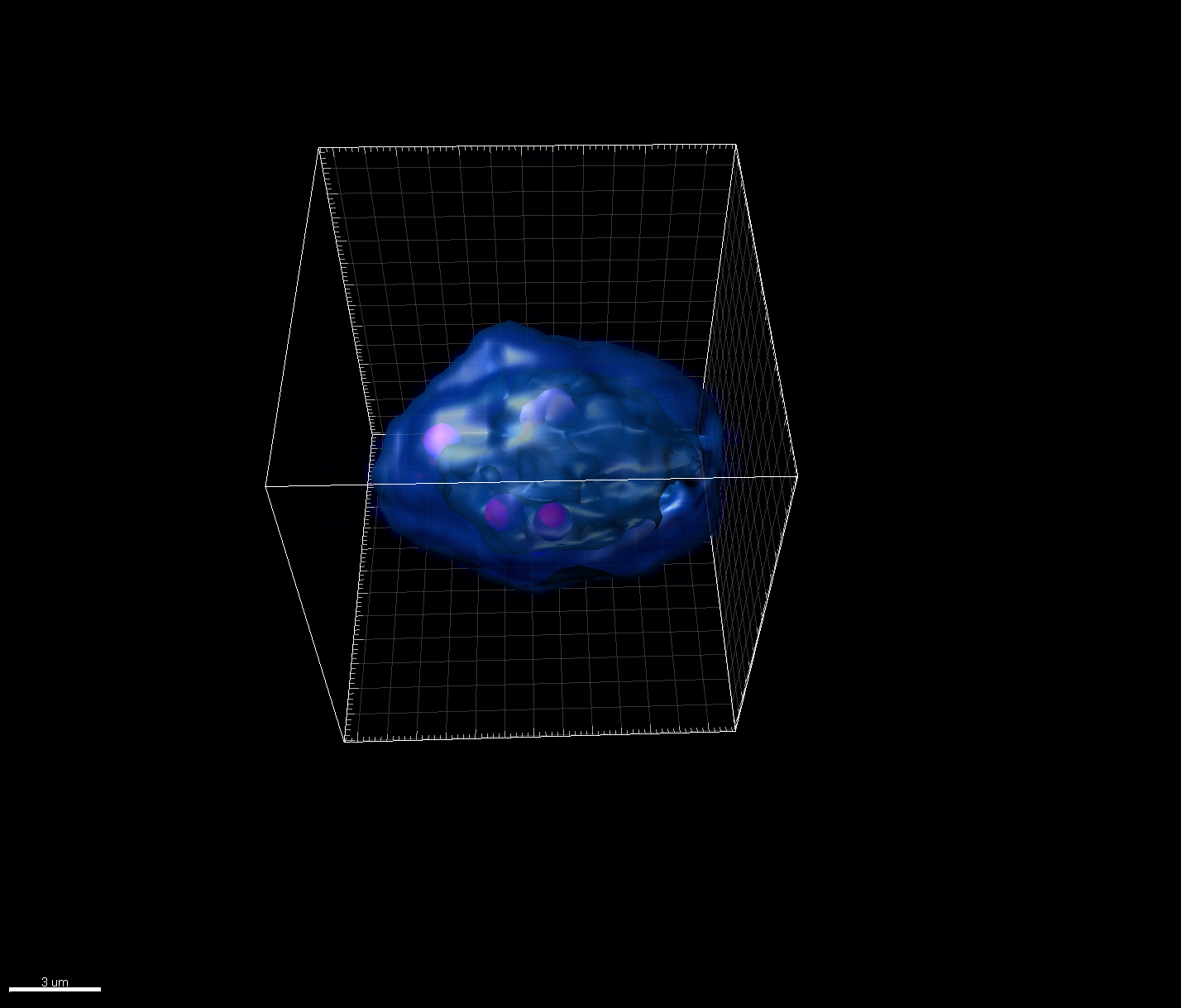

Supplement: Figure 2—source data 2. [file elife-85412-fig2-data2.zip › Figure 2 -source data 2 Figure B/3D ctr sgRNA.tif]

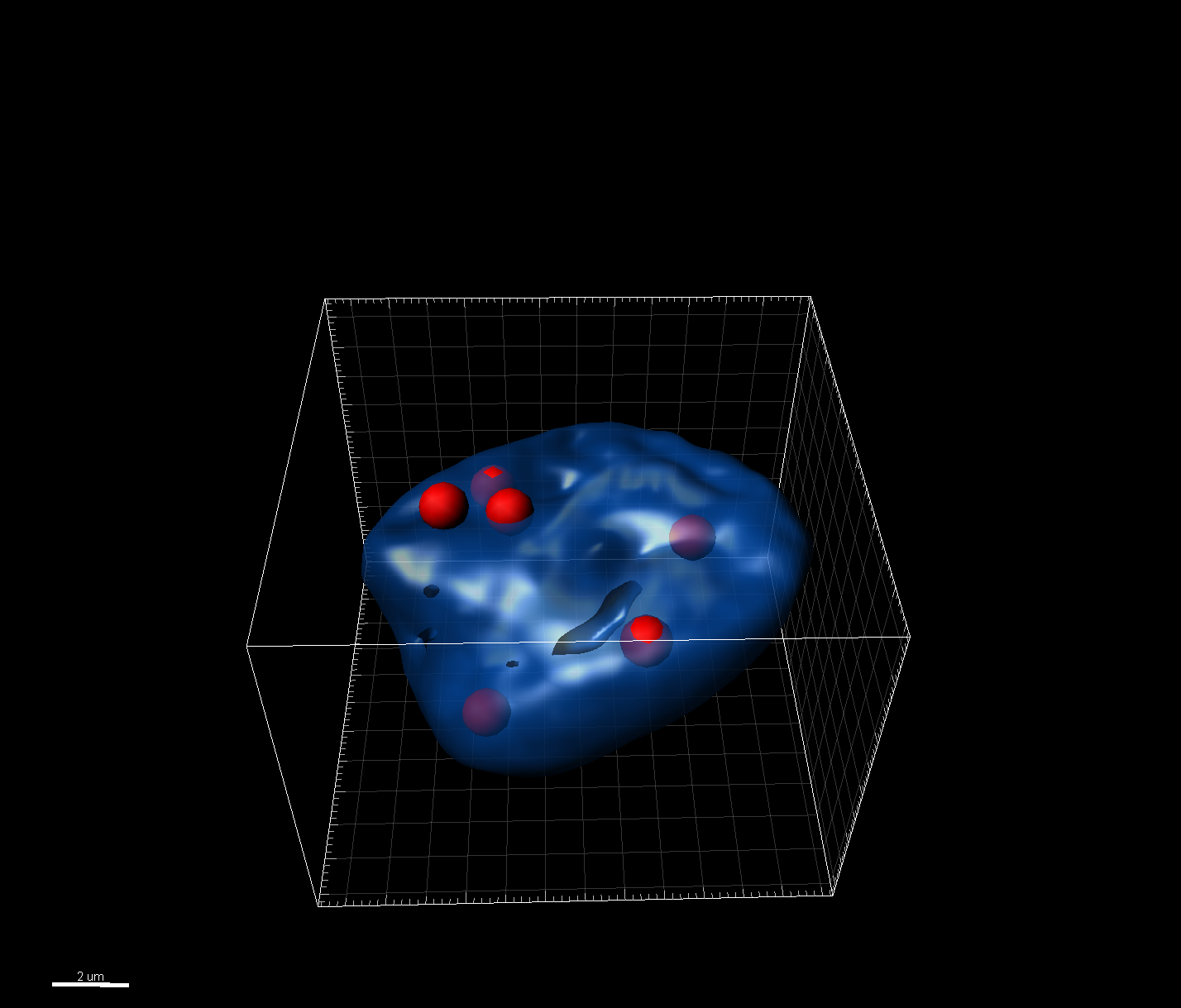

Supplement: Figure 2—source data 2. [file elife-85412-fig2-data2.zip › Figure 2 -source data 2 Figure B/3D HSV-1 sgRNA.tif]

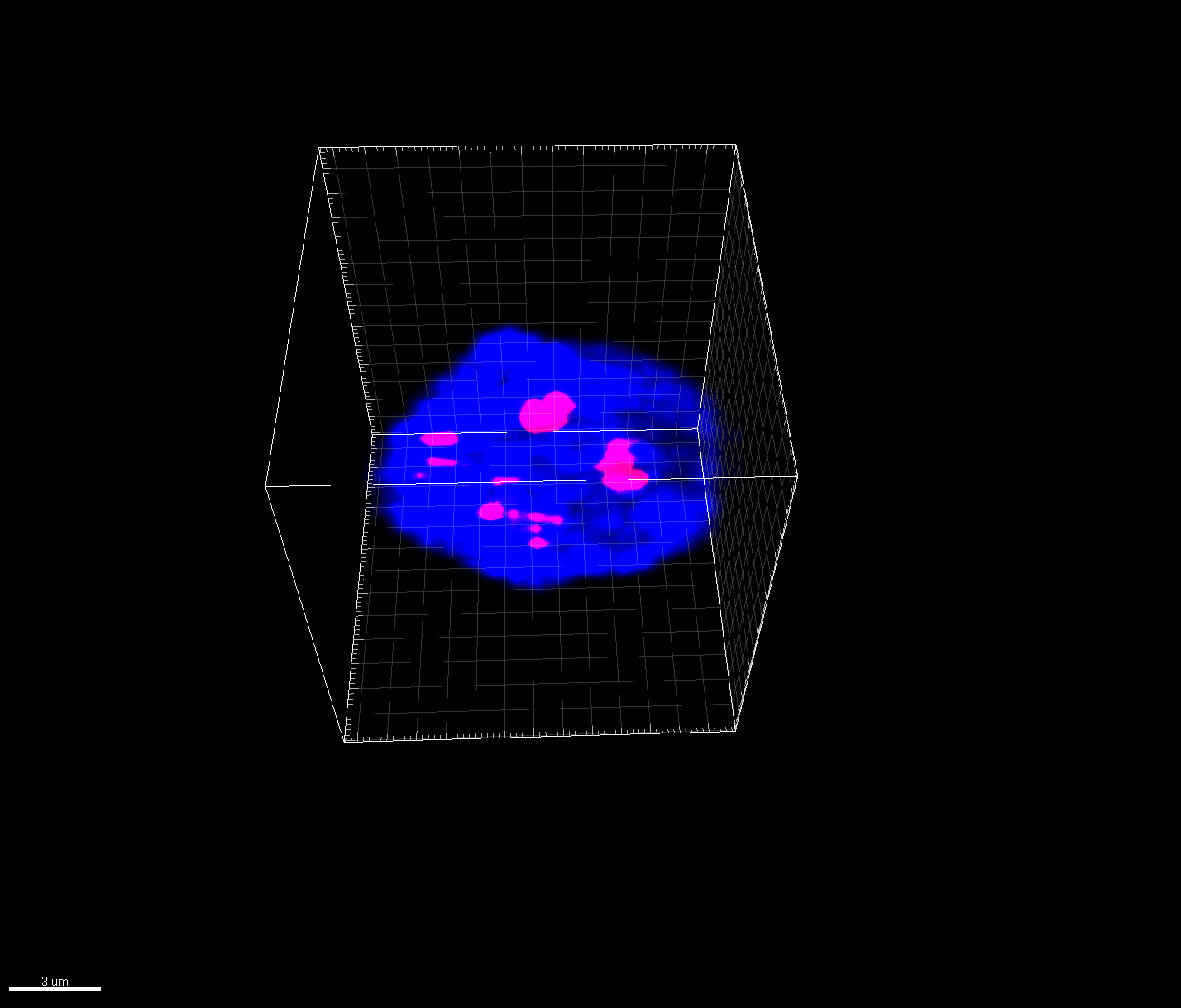

Supplement: Figure 2—source data 2. [file elife-85412-fig2-data2.zip › Figure 2 -source data 2 Figure B/ctr sgRNA.tif]

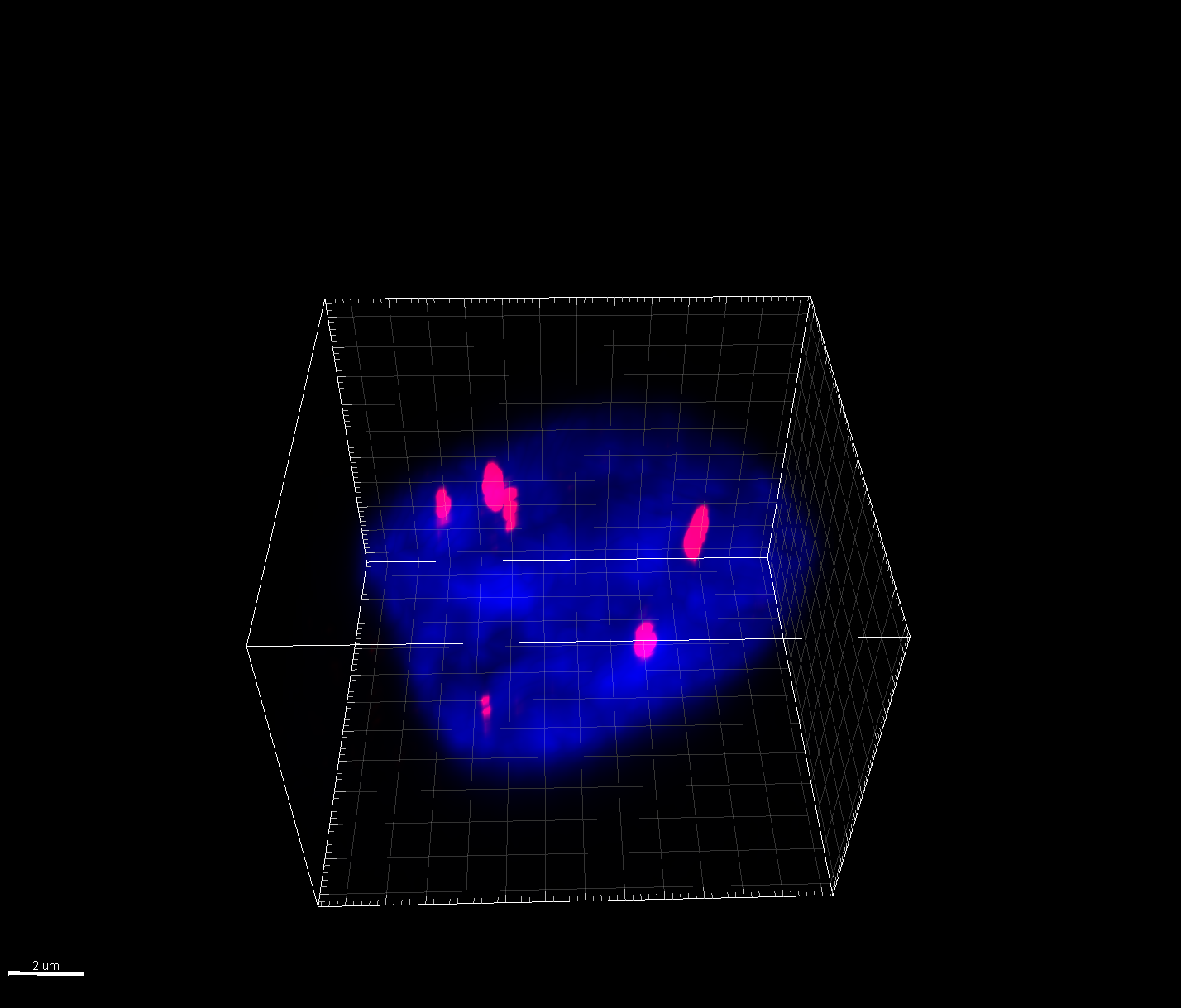

Supplement: Figure 2—source data 2. [file elife-85412-fig2-data2.zip › Figure 2 -source data 2 Figure B/HSV-1 sgRNA.tif]

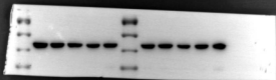

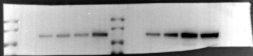

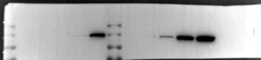

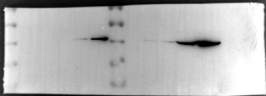

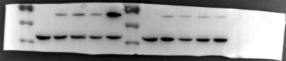

Supplement: Figure 2—source data 3. [file elife-85412-fig2-data3.pdf]

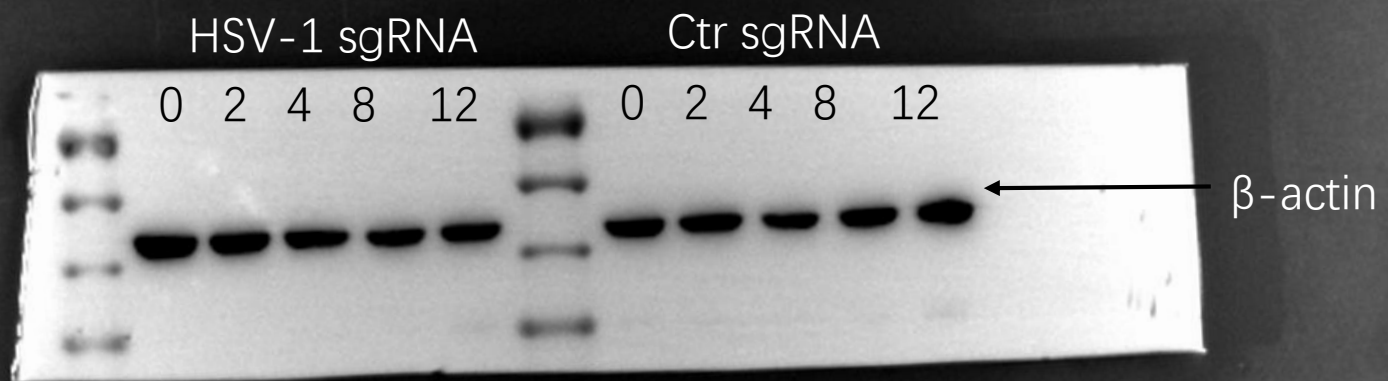

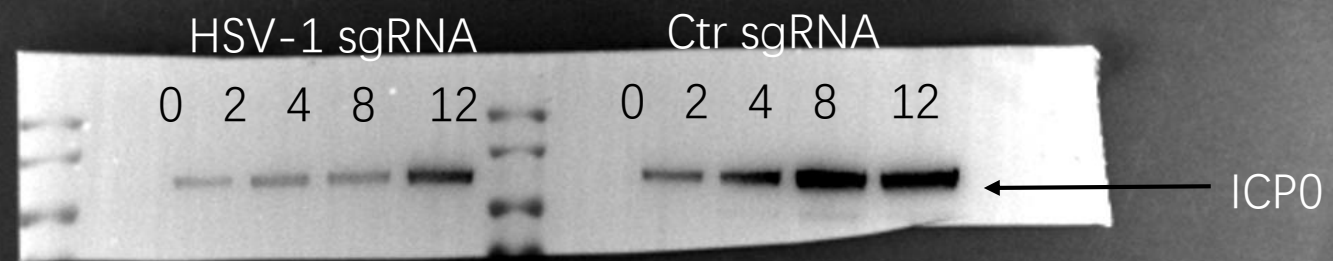

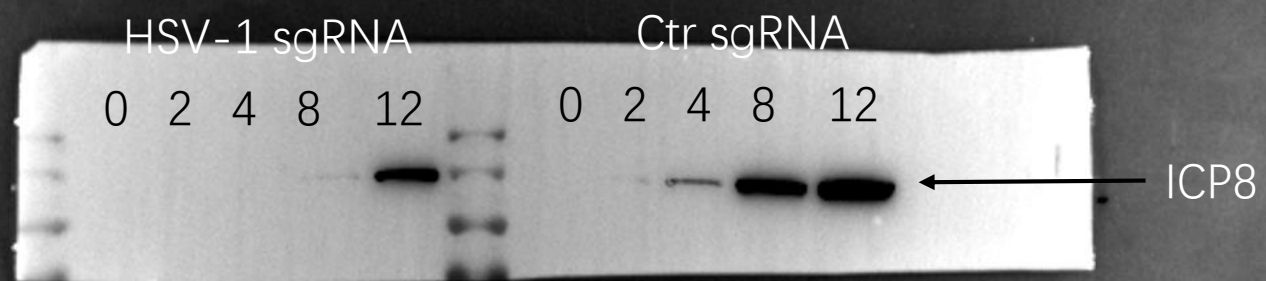

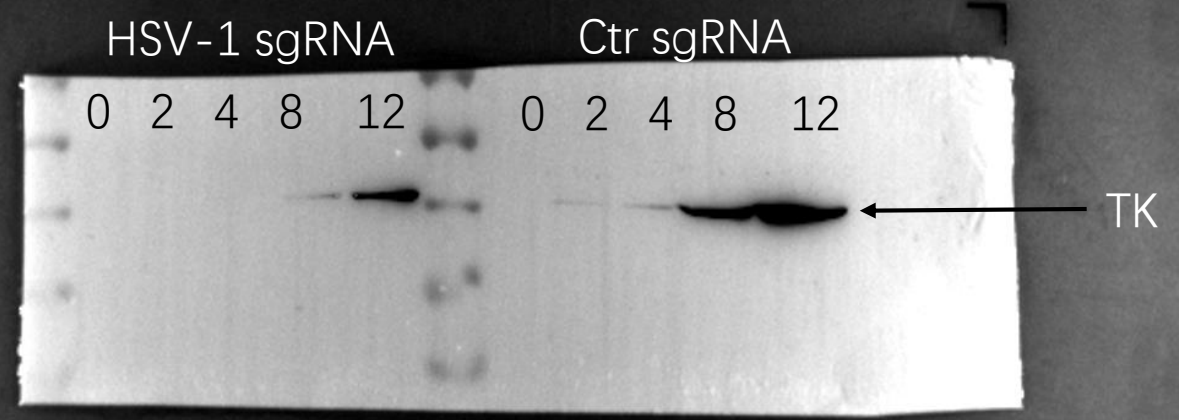

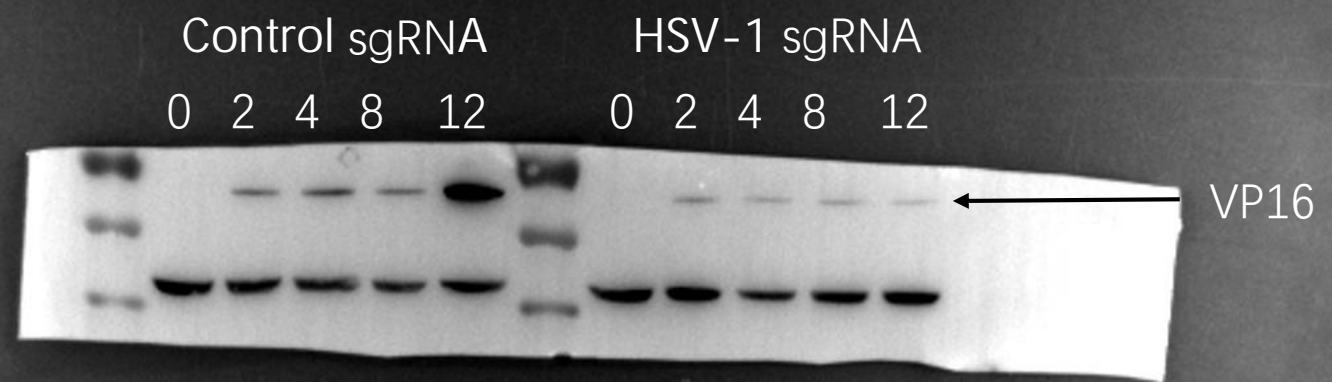

Supplement: Figure 2—source data 4. [file elife-85412-fig2-data4.pdf]

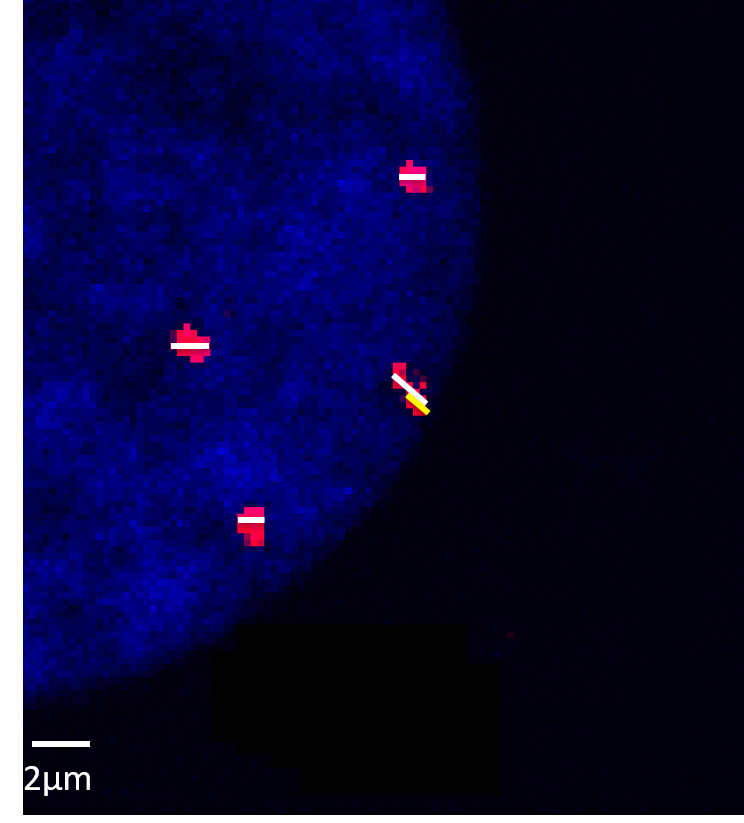

Supplement: Figure 2—figure supplement 1—source data 2. [file elife-85412-fig2-figsupp1-data2.zip › Figure 2-figure supplement 1-source data 2 Figure 2-figure supplement 1 B/Sup Fig 2 B.tif]

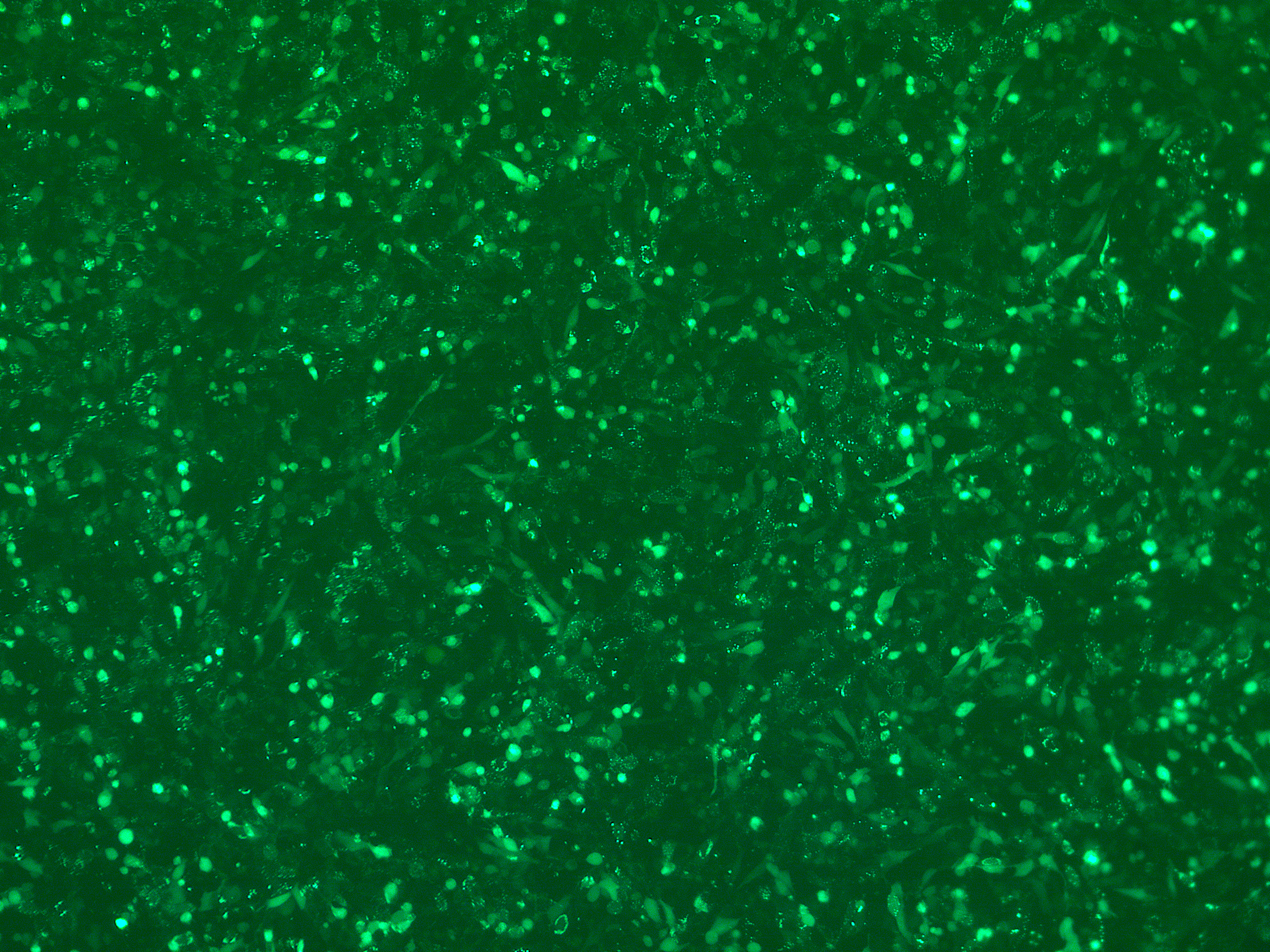

Supplement: Figure 2—figure supplement 1—source data 3. [file elife-85412-fig2-figsupp1-data3.zip › Figure 2-figure supplement 1-source data 3 Figure 2-figure supplement 1 G/Sup Fig 2 G dcas9 2022-01-28.tif]

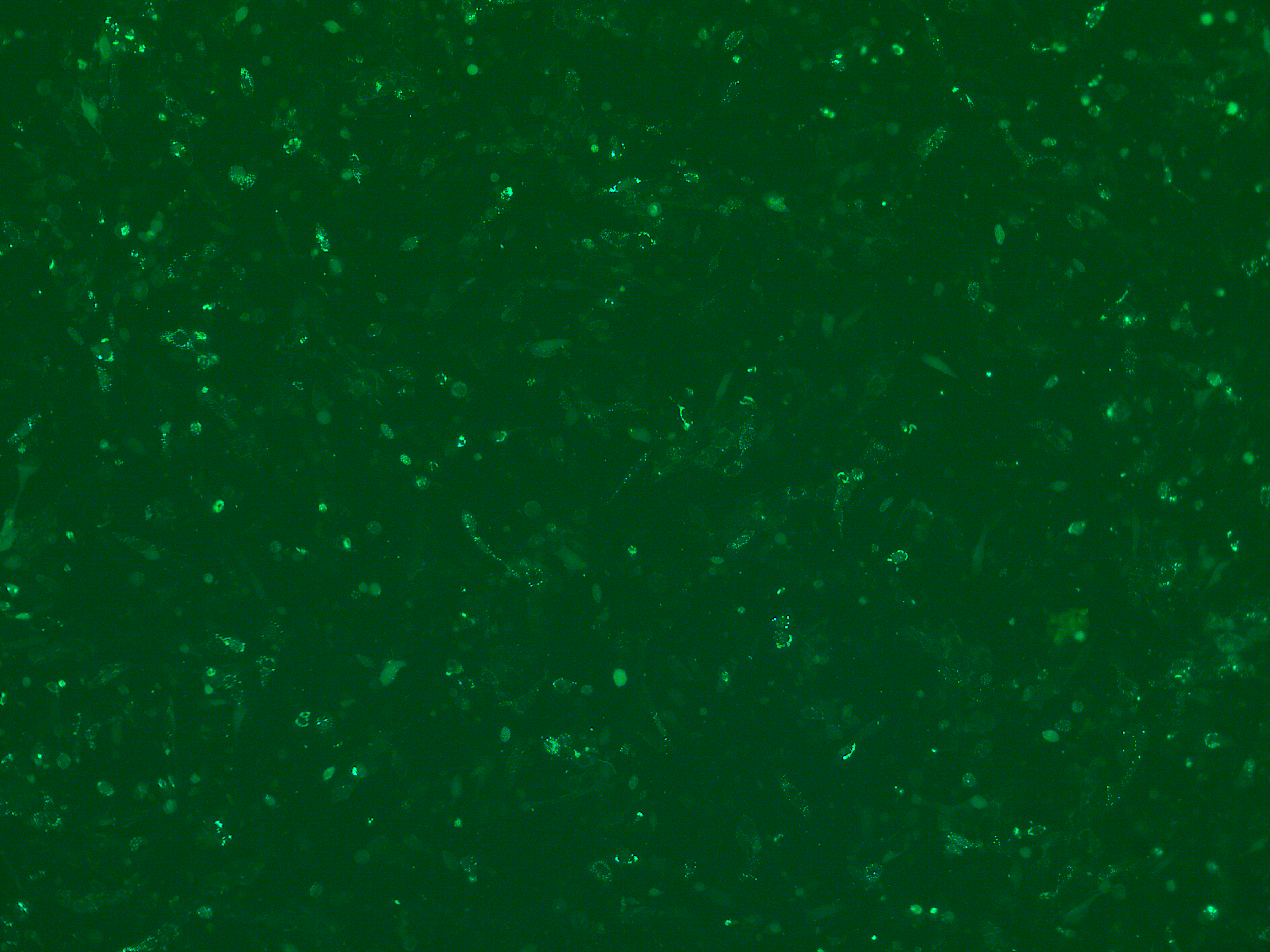

Supplement: Figure 2—figure supplement 1—source data 3. [file elife-85412-fig2-figsupp1-data3.zip › Figure 2-figure supplement 1-source data 3 Figure 2-figure supplement 1 G/Sup Fig 2 G dcas9-emerin 2022-01-28 .tif]

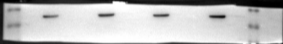

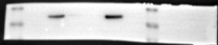

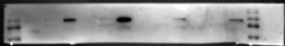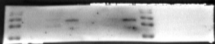

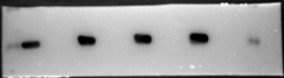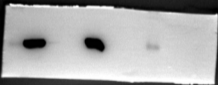

Supplement: Figure 2—figure supplement 1—source data 4. [file elife-85412-fig2-figsupp1-data4.pdf]

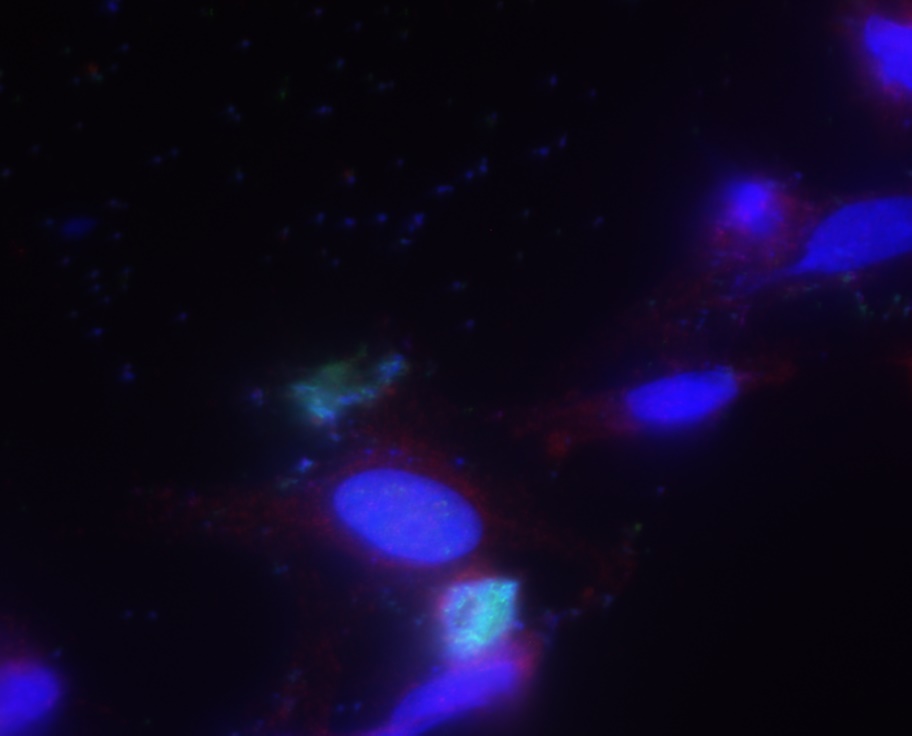

Supplement: Figure 2—figure supplement 1—source data 6. [file elife-85412-fig2-figsupp1-data6.zip › Figure 2-figure supplement 1-source data 6 Figure 2-figure supplement 1 J/All TIFF FILES/+Dox/4hr-DOX-5 (1).jpg]

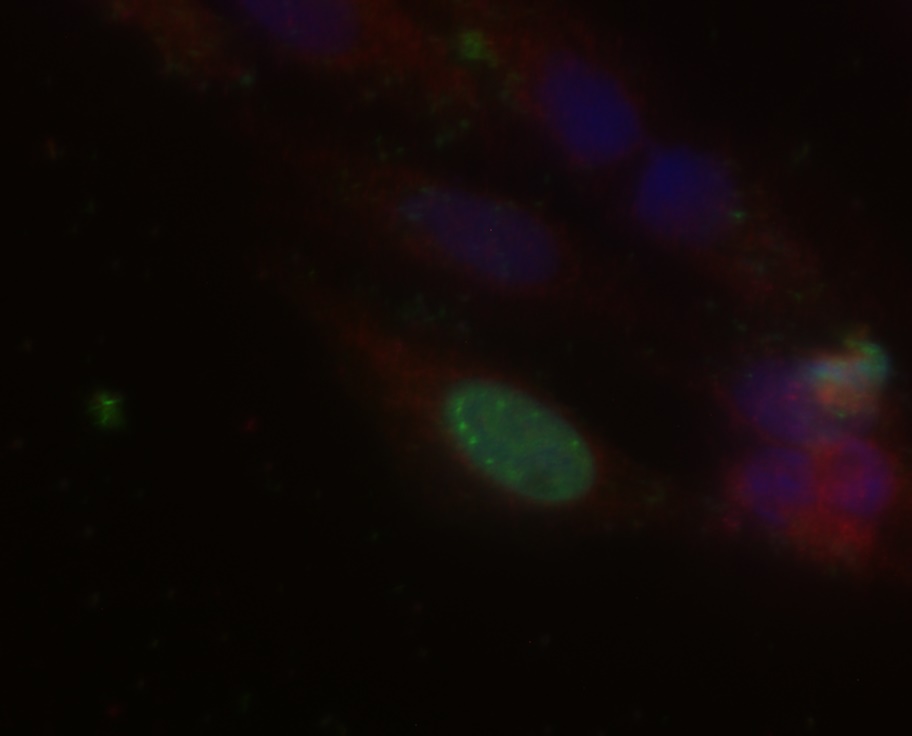

Supplement: Figure 2—figure supplement 1—source data 6. [file elife-85412-fig2-figsupp1-data6.zip › Figure 2-figure supplement 1-source data 6 Figure 2-figure supplement 1 J/All TIFF FILES/+Dox/4hr-DOX-5 (2).jpg]

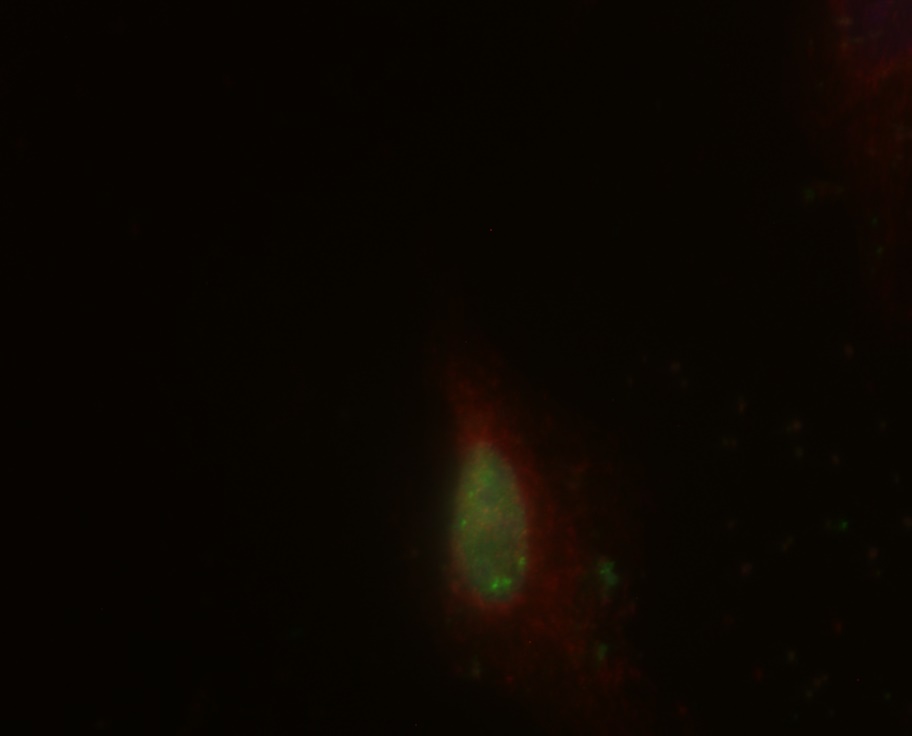

Supplement: Figure 2—figure supplement 1—source data 6. [file elife-85412-fig2-figsupp1-data6.zip › Figure 2-figure supplement 1-source data 6 Figure 2-figure supplement 1 J/All TIFF FILES/+Dox/4hr-DOX-5 (3).jpg]

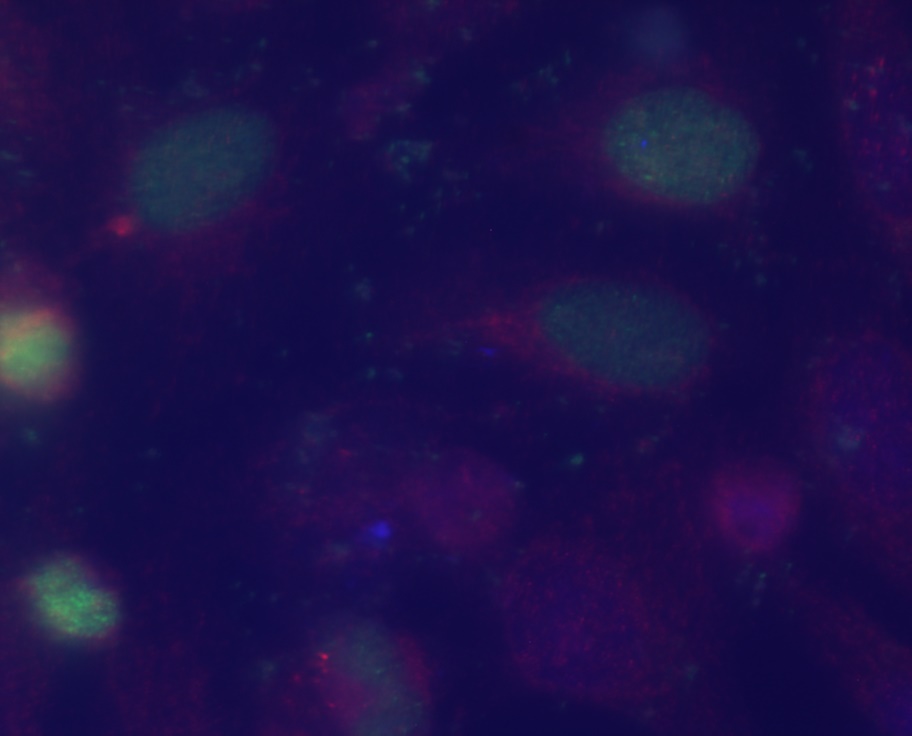

Supplement: Figure 2—figure supplement 1—source data 6. [file elife-85412-fig2-figsupp1-data6.zip › Figure 2-figure supplement 1-source data 6 Figure 2-figure supplement 1 J/All TIFF FILES/+Dox/4hr-DOX-5 (4).jpg]

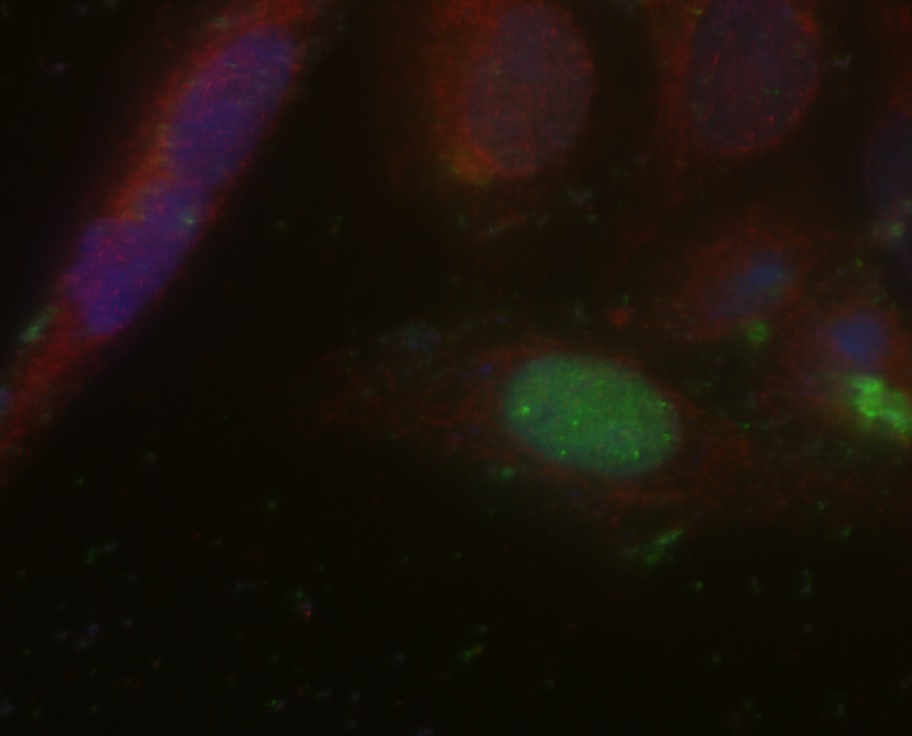

Supplement: Figure 2—figure supplement 1—source data 6. [file elife-85412-fig2-figsupp1-data6.zip › Figure 2-figure supplement 1-source data 6 Figure 2-figure supplement 1 J/All TIFF FILES/+Dox/4hr-DOX-5 (5).jpg]

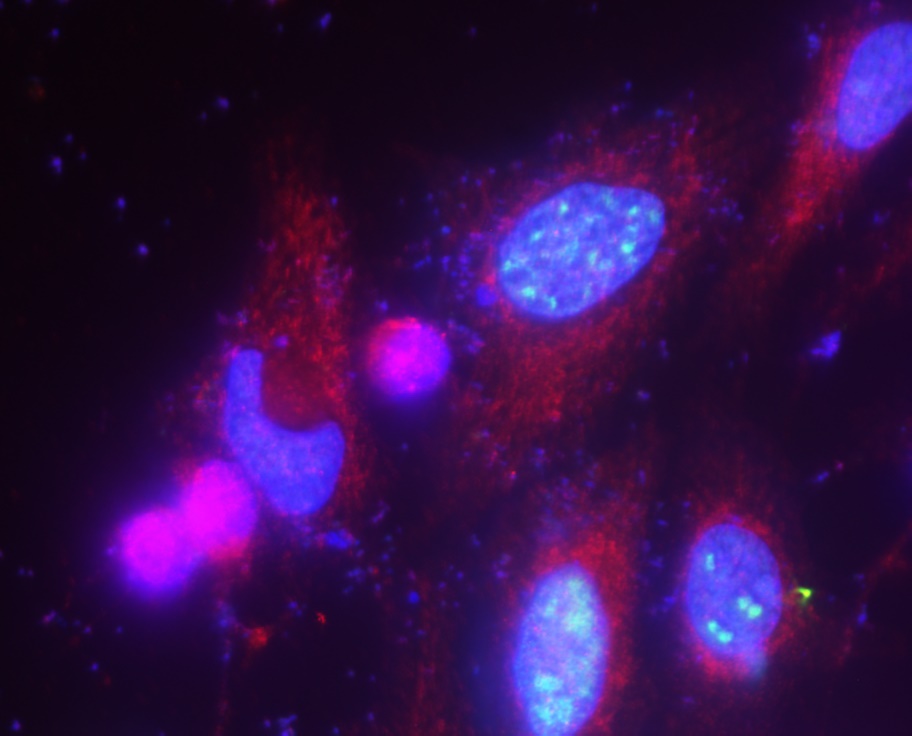

Supplement: Figure 2—figure supplement 1—source data 6. [file elife-85412-fig2-figsupp1-data6.zip › Figure 2-figure supplement 1-source data 6 Figure 2-figure supplement 1 J/All TIFF FILES/+Dox/6hr-DOX-6 (10).jpg]

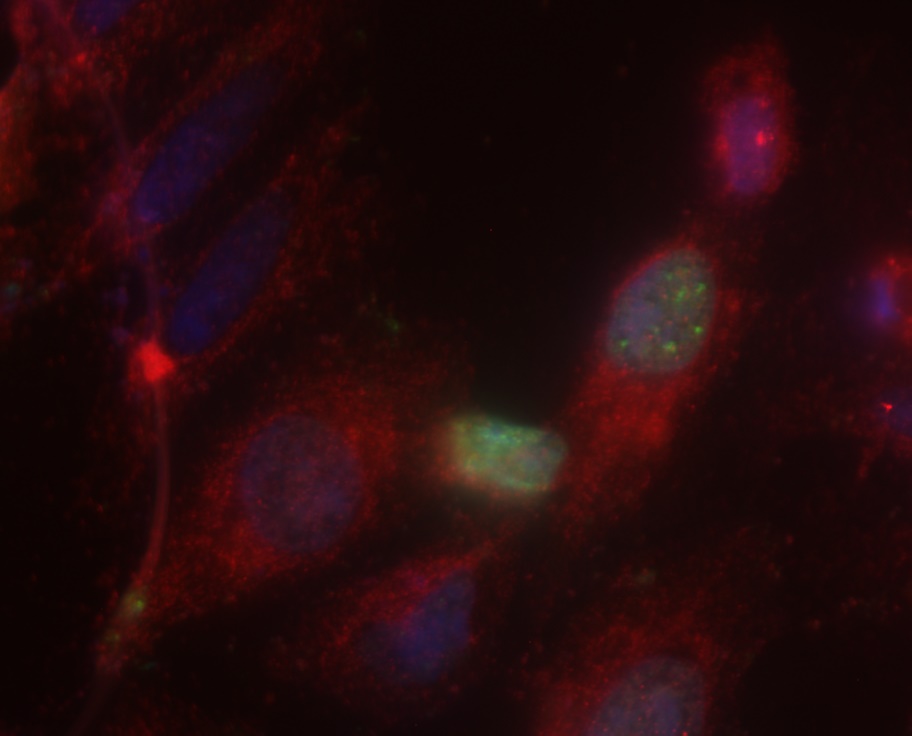

Supplement: Figure 2—figure supplement 1—source data 6. [file elife-85412-fig2-figsupp1-data6.zip › Figure 2-figure supplement 1-source data 6 Figure 2-figure supplement 1 J/All TIFF FILES/+Dox/6hr-DOX-6 (11).jpg]

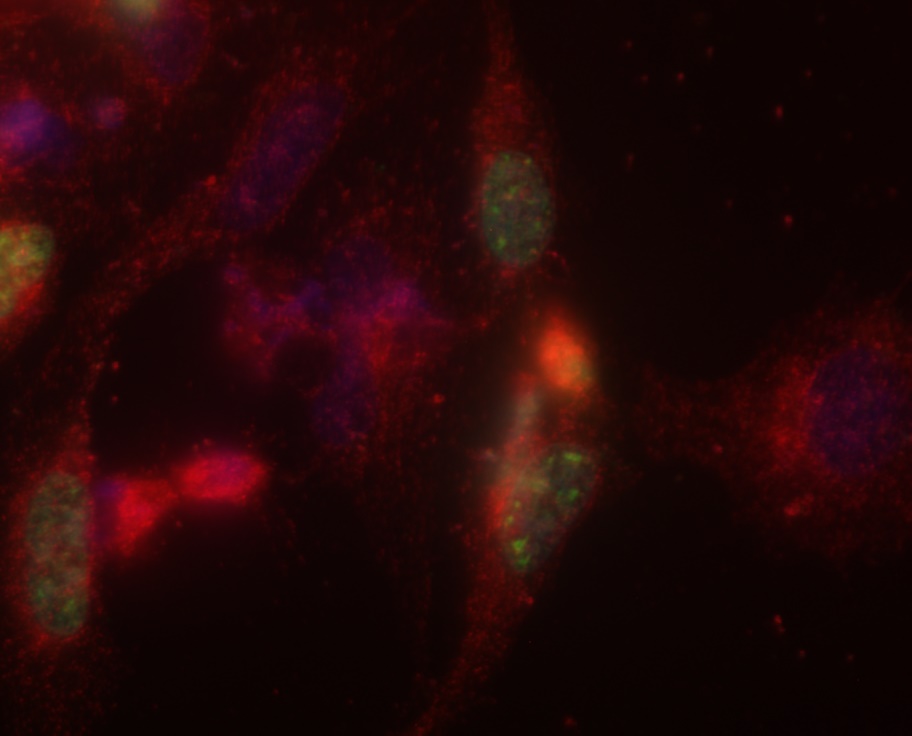

Supplement: Figure 2—figure supplement 1—source data 6. [file elife-85412-fig2-figsupp1-data6.zip › Figure 2-figure supplement 1-source data 6 Figure 2-figure supplement 1 J/All TIFF FILES/+Dox/6hr-DOX-6 (6).jpg]

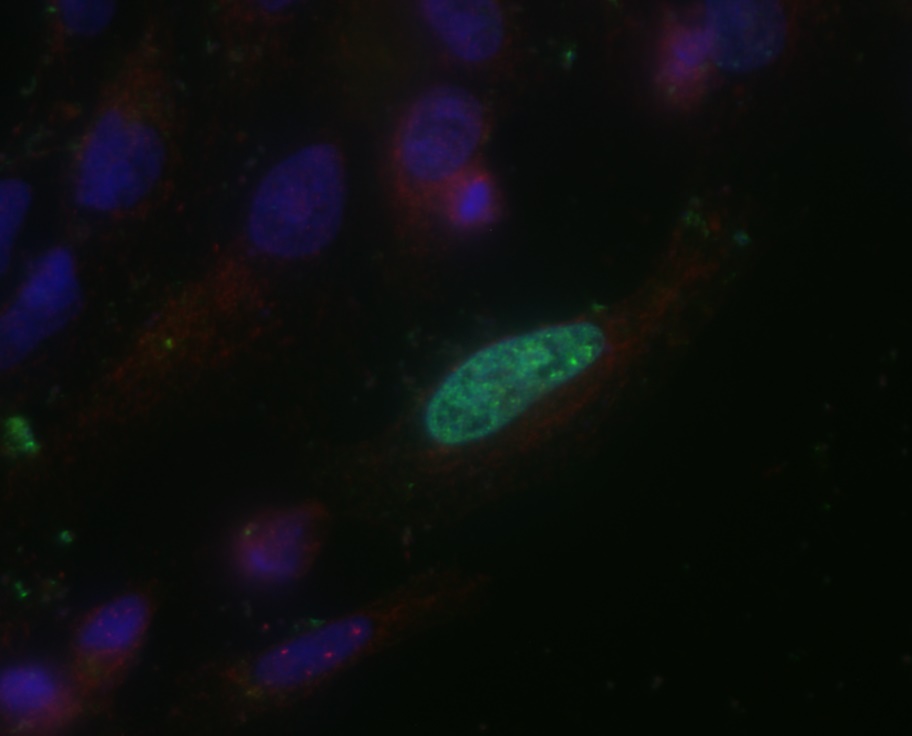

Supplement: Figure 2—figure supplement 1—source data 6. [file elife-85412-fig2-figsupp1-data6.zip › Figure 2-figure supplement 1-source data 6 Figure 2-figure supplement 1 J/All TIFF FILES/+Dox/6hr-DOX-6 (7).jpg]

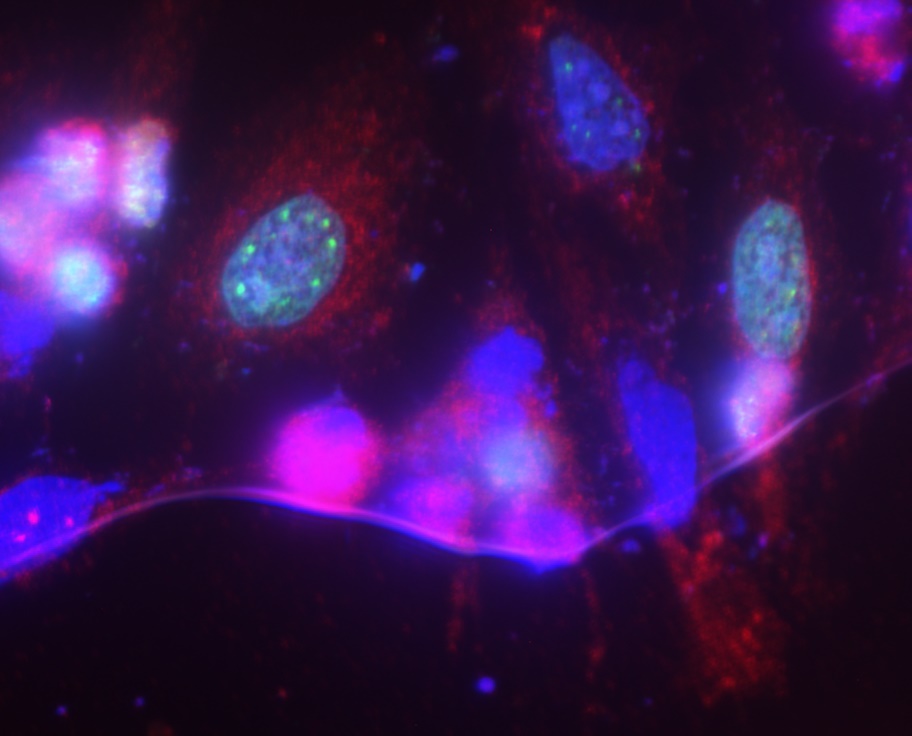

Supplement: Figure 2—figure supplement 1—source data 6. [file elife-85412-fig2-figsupp1-data6.zip › Figure 2-figure supplement 1-source data 6 Figure 2-figure supplement 1 J/All TIFF FILES/+Dox/6hr-DOX-6 (8).jpg]

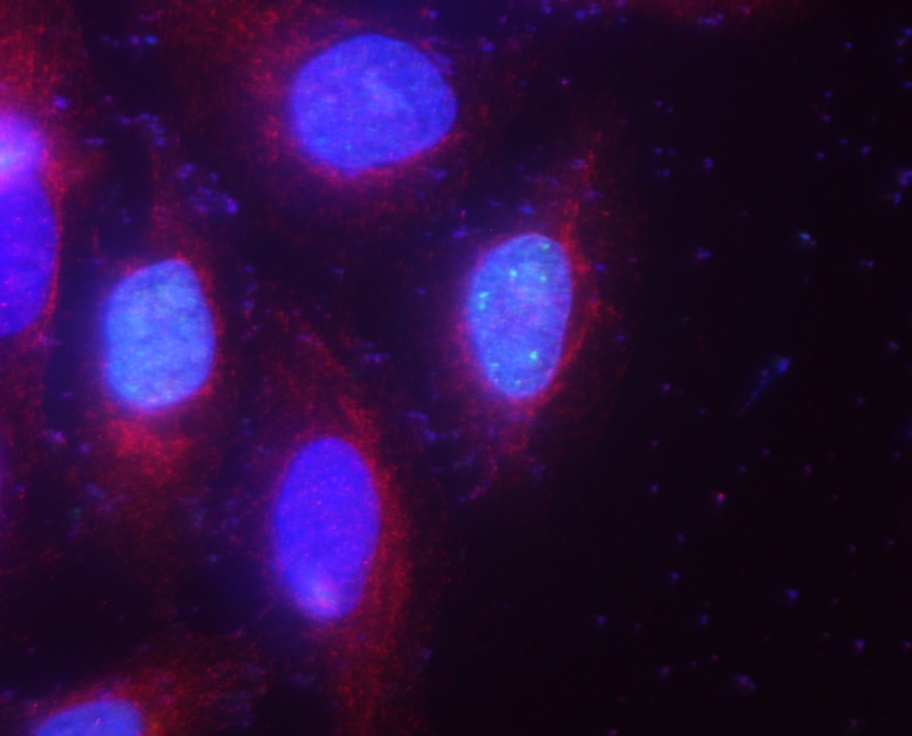

Supplement: Figure 2—figure supplement 1—source data 6. [file elife-85412-fig2-figsupp1-data6.zip › Figure 2-figure supplement 1-source data 6 Figure 2-figure supplement 1 J/All TIFF FILES/+Dox/6hr-DOX-6 (9).jpg]

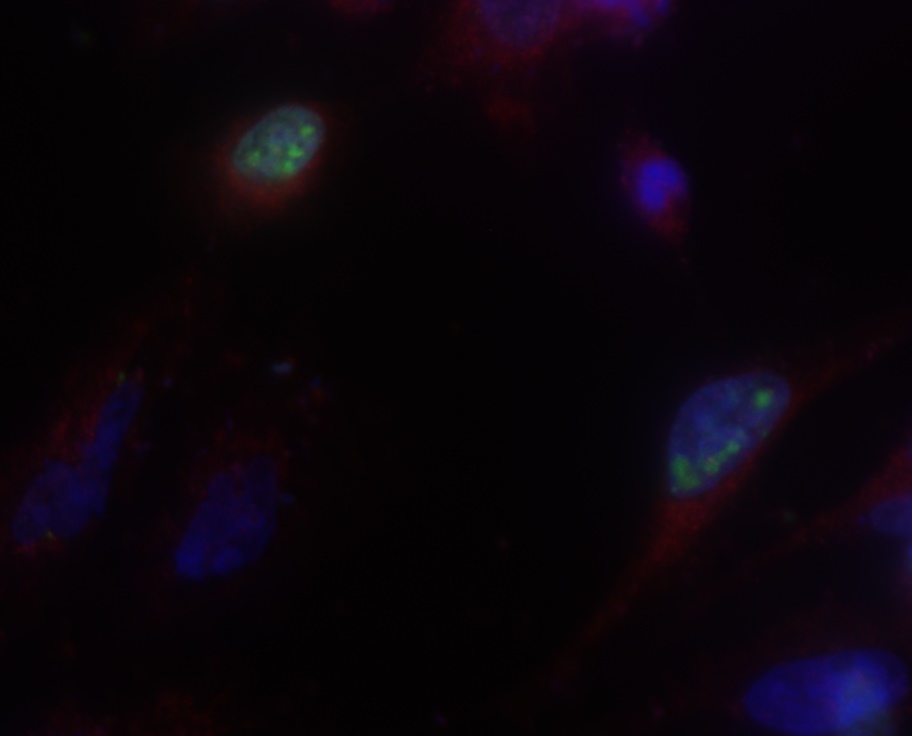

Supplement: Figure 2—figure supplement 1—source data 6. [file elife-85412-fig2-figsupp1-data6.zip › Figure 2-figure supplement 1-source data 6 Figure 2-figure supplement 1 J/All TIFF FILES/+Dox/8hr-DOX1 (1).jpg]

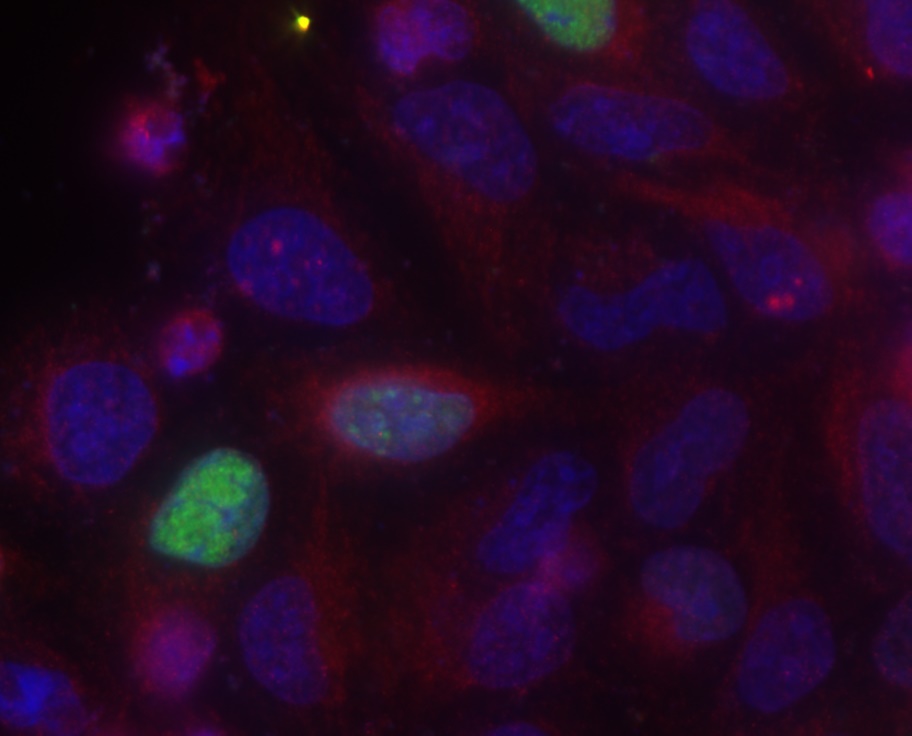

Supplement: Figure 2—figure supplement 1—source data 6. [file elife-85412-fig2-figsupp1-data6.zip › Figure 2-figure supplement 1-source data 6 Figure 2-figure supplement 1 J/All TIFF FILES/+Dox/8hr-DOX1 (2).jpg]

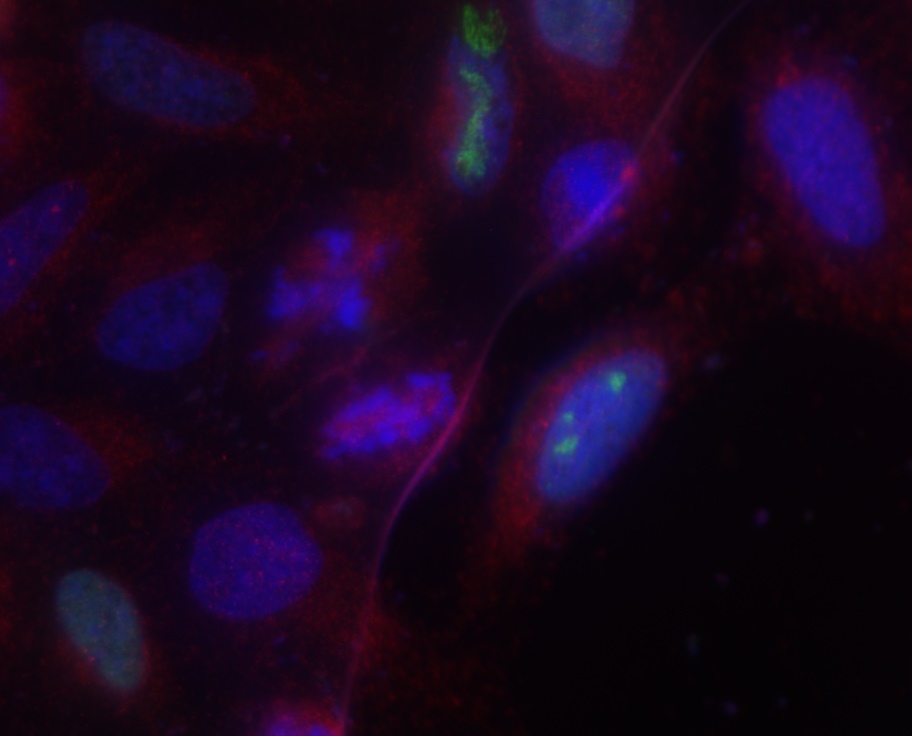

Supplement: Figure 2—figure supplement 1—source data 6. [file elife-85412-fig2-figsupp1-data6.zip › Figure 2-figure supplement 1-source data 6 Figure 2-figure supplement 1 J/All TIFF FILES/+Dox/8hr-DOX1 (3).jpg]

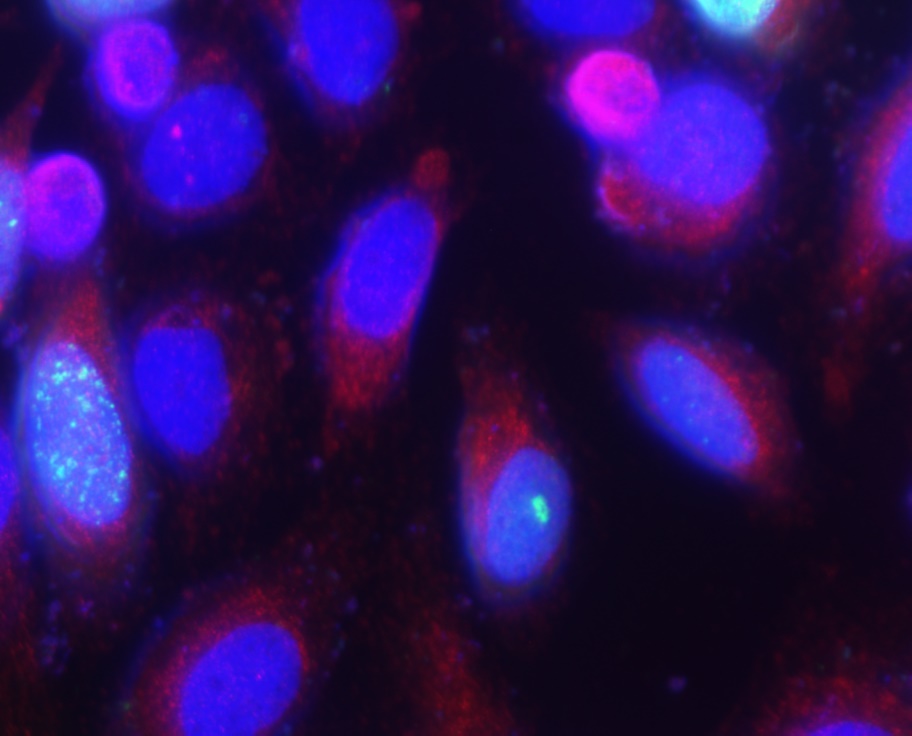

Supplement: Figure 2—figure supplement 1—source data 6. [file elife-85412-fig2-figsupp1-data6.zip › Figure 2-figure supplement 1-source data 6 Figure 2-figure supplement 1 J/All TIFF FILES/+Dox/8hr-DOX1 (4).jpg]

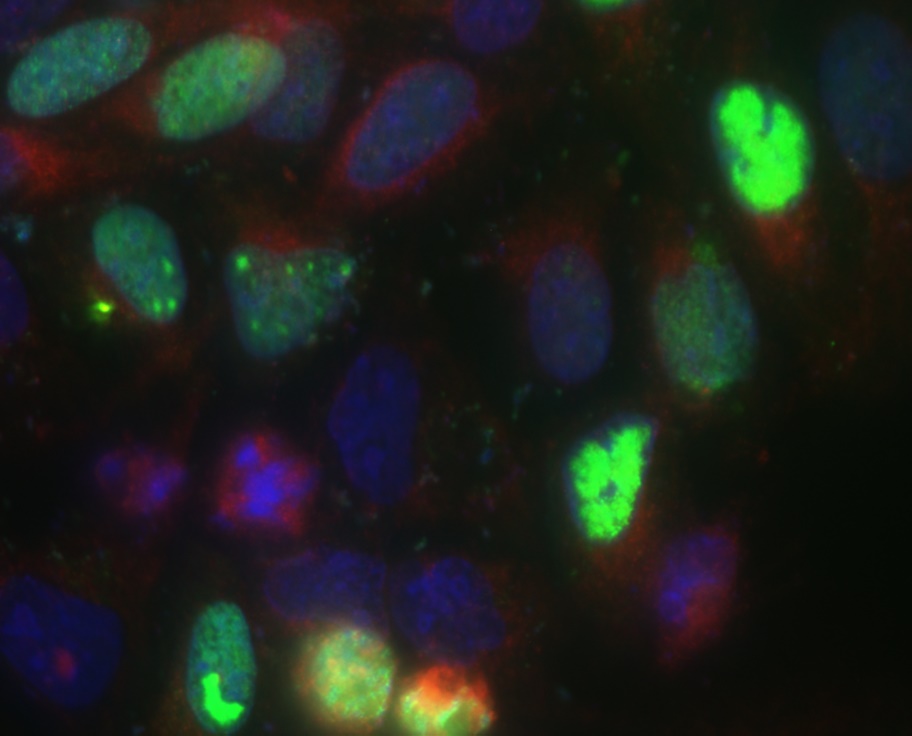

Supplement: Figure 2—figure supplement 1—source data 6. [file elife-85412-fig2-figsupp1-data6.zip › Figure 2-figure supplement 1-source data 6 Figure 2-figure supplement 1 J/All TIFF FILES/+Dox/8hr-DOX1 (5).jpg]

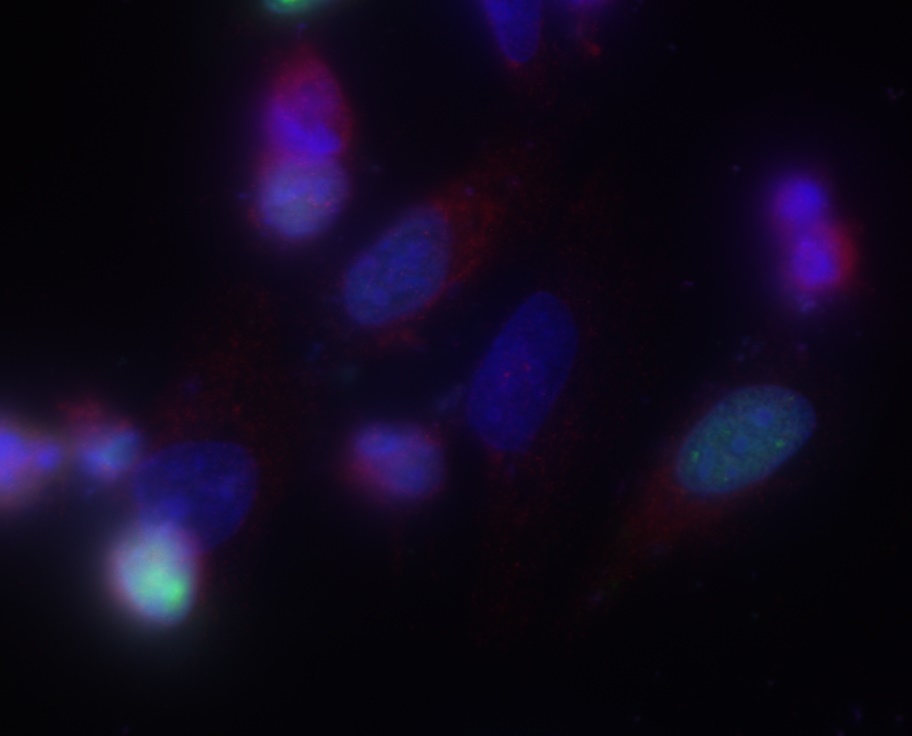

Supplement: Figure 2—figure supplement 1—source data 6. [file elife-85412-fig2-figsupp1-data6.zip › Figure 2-figure supplement 1-source data 6 Figure 2-figure supplement 1 J/All TIFF FILES/+Dox/8hr-DOX1 (6).jpg]

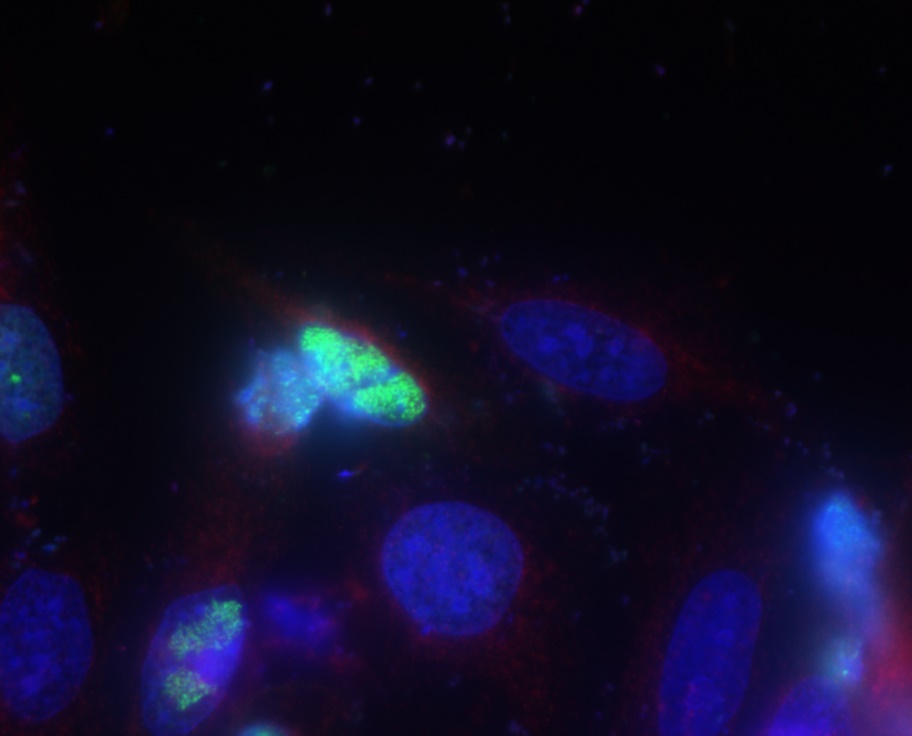

Supplement: Figure 2—figure supplement 1—source data 6. [file elife-85412-fig2-figsupp1-data6.zip › Figure 2-figure supplement 1-source data 6 Figure 2-figure supplement 1 J/All TIFF FILES/+Dox/8hr-DOX1 (7).jpg]

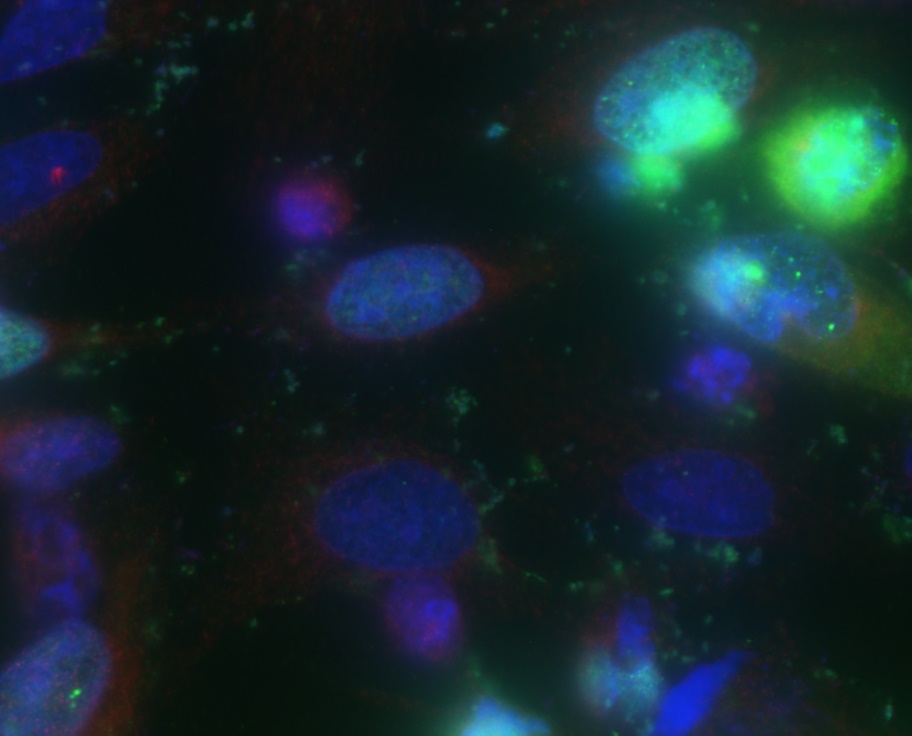

Supplement: Figure 2—figure supplement 1—source data 6. [file elife-85412-fig2-figsupp1-data6.zip › Figure 2-figure supplement 1-source data 6 Figure 2-figure supplement 1 J/All TIFF FILES/+Dox/8hr-DOX1 (8).jpg]

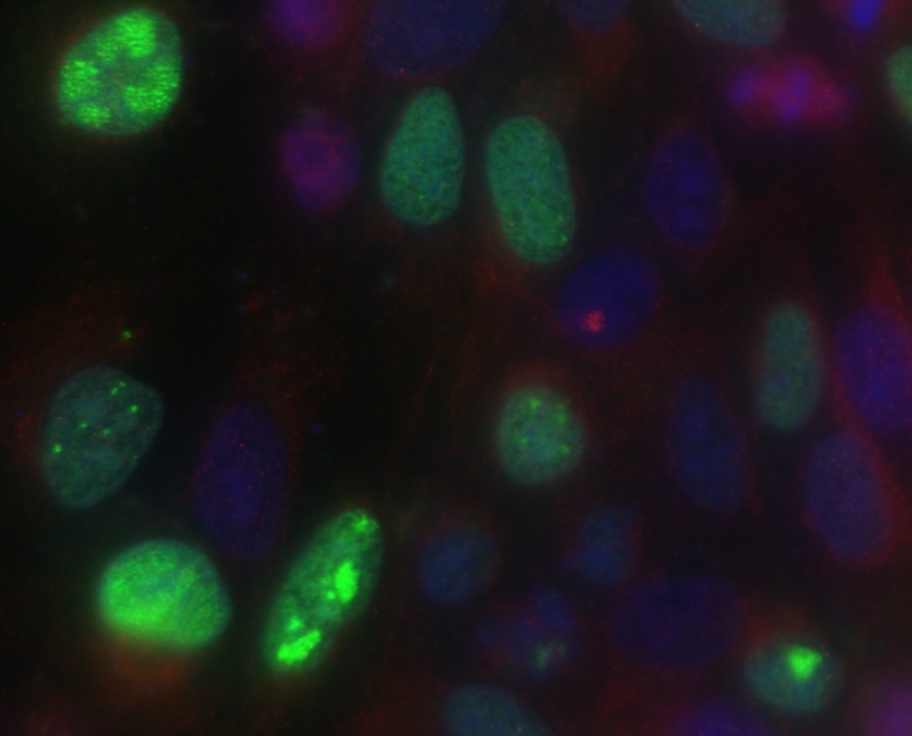

Supplement: Figure 2—figure supplement 1—source data 6. [file elife-85412-fig2-figsupp1-data6.zip › Figure 2-figure supplement 1-source data 6 Figure 2-figure supplement 1 J/All TIFF FILES/+Dox/8hr-DOX1 (9).jpg]

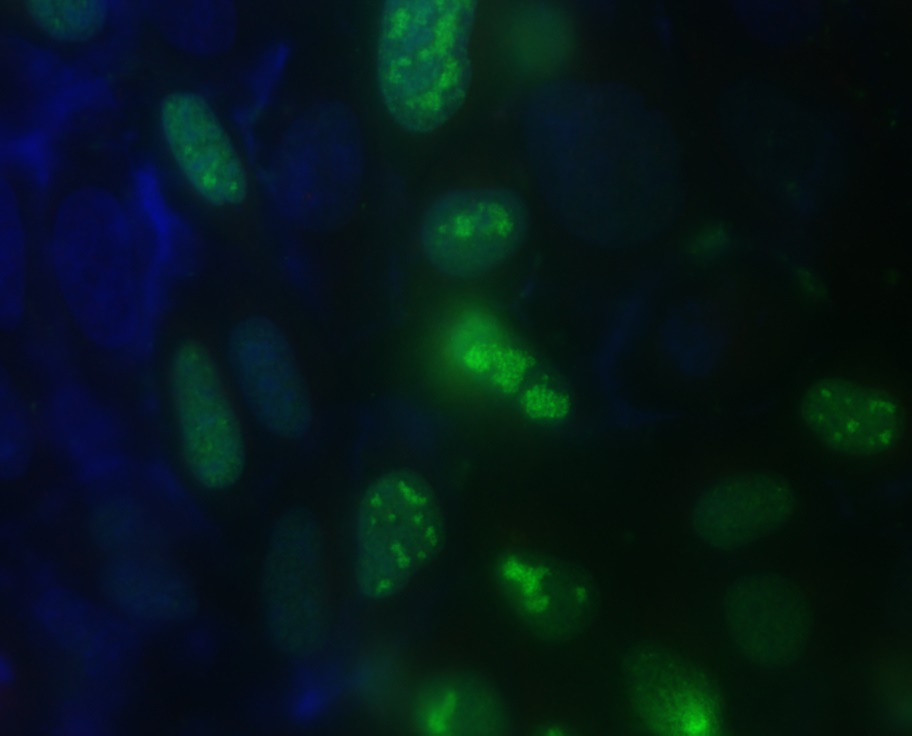

Supplement: Figure 2—figure supplement 1—source data 6. [file elife-85412-fig2-figsupp1-data6.zip › Figure 2-figure supplement 1-source data 6 Figure 2-figure supplement 1 J/All TIFF FILES/-Dox/4hr-1.jpg]

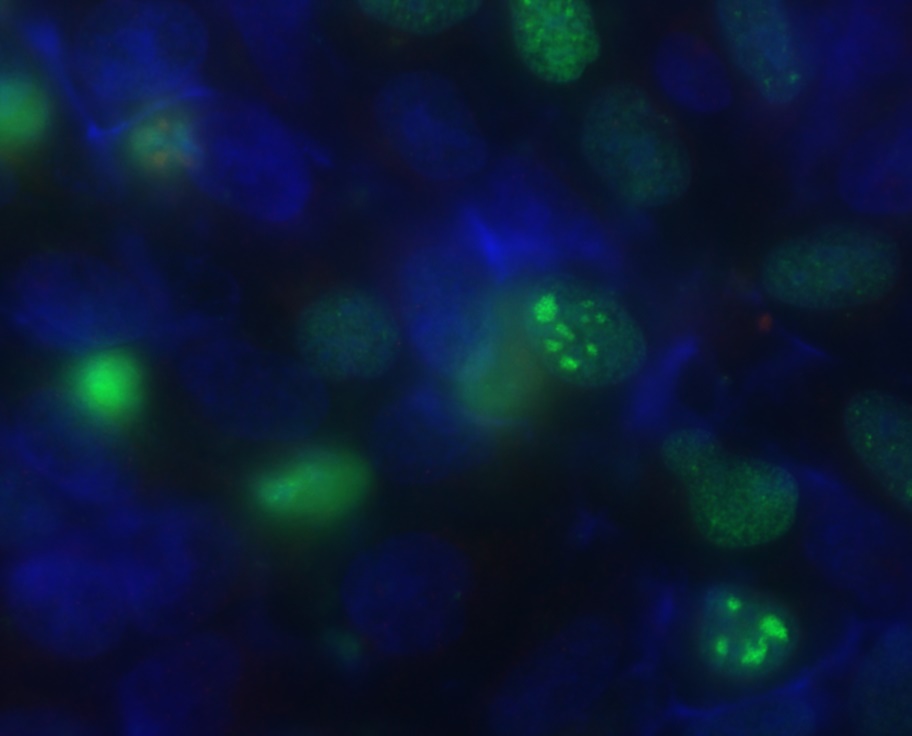

Supplement: Figure 2—figure supplement 1—source data 6. [file elife-85412-fig2-figsupp1-data6.zip › Figure 2-figure supplement 1-source data 6 Figure 2-figure supplement 1 J/All TIFF FILES/-Dox/4hr-2.jpg]

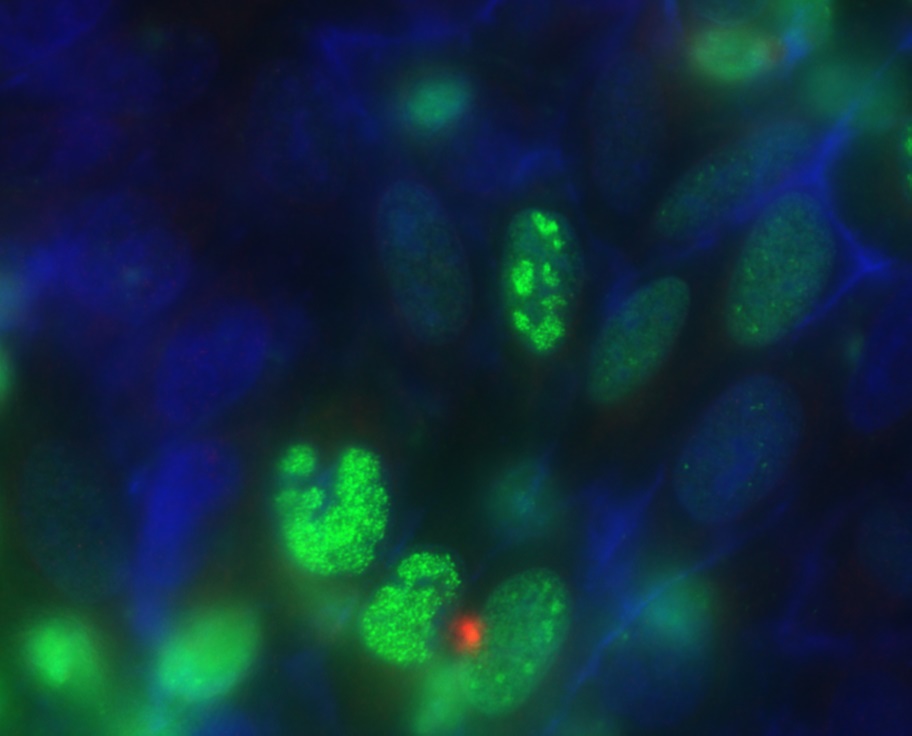

Supplement: Figure 2—figure supplement 1—source data 6. [file elife-85412-fig2-figsupp1-data6.zip › Figure 2-figure supplement 1-source data 6 Figure 2-figure supplement 1 J/All TIFF FILES/-Dox/4hr-3.jpg]

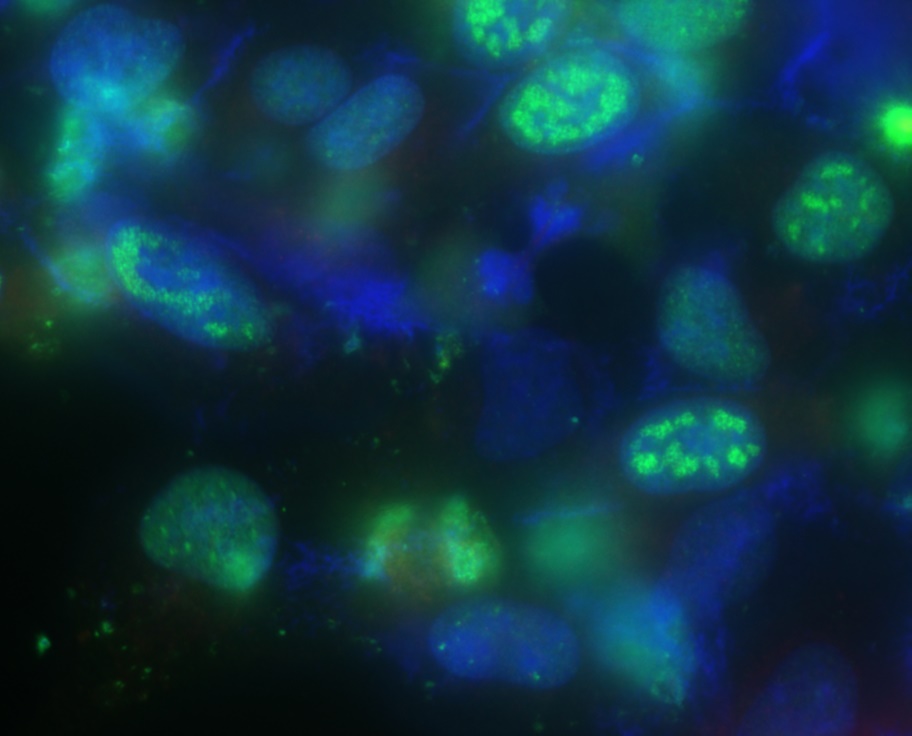

Supplement: Figure 2—figure supplement 1—source data 6. [file elife-85412-fig2-figsupp1-data6.zip › Figure 2-figure supplement 1-source data 6 Figure 2-figure supplement 1 J/All TIFF FILES/-Dox/4hr-4.jpg]

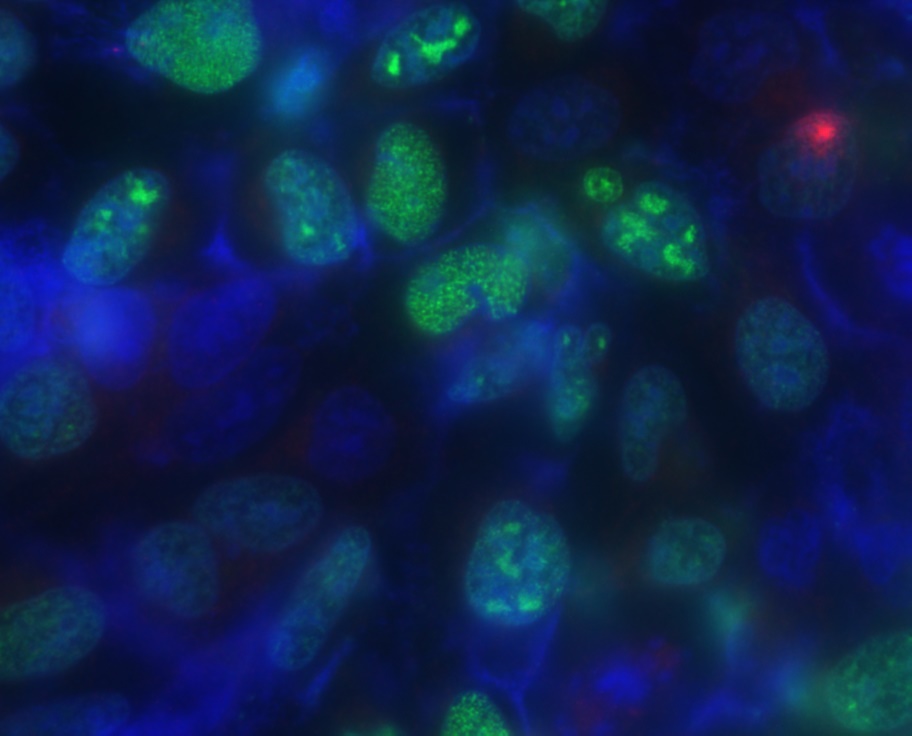

Supplement: Figure 2—figure supplement 1—source data 6. [file elife-85412-fig2-figsupp1-data6.zip › Figure 2-figure supplement 1-source data 6 Figure 2-figure supplement 1 J/All TIFF FILES/-Dox/6hr-1.jpg]

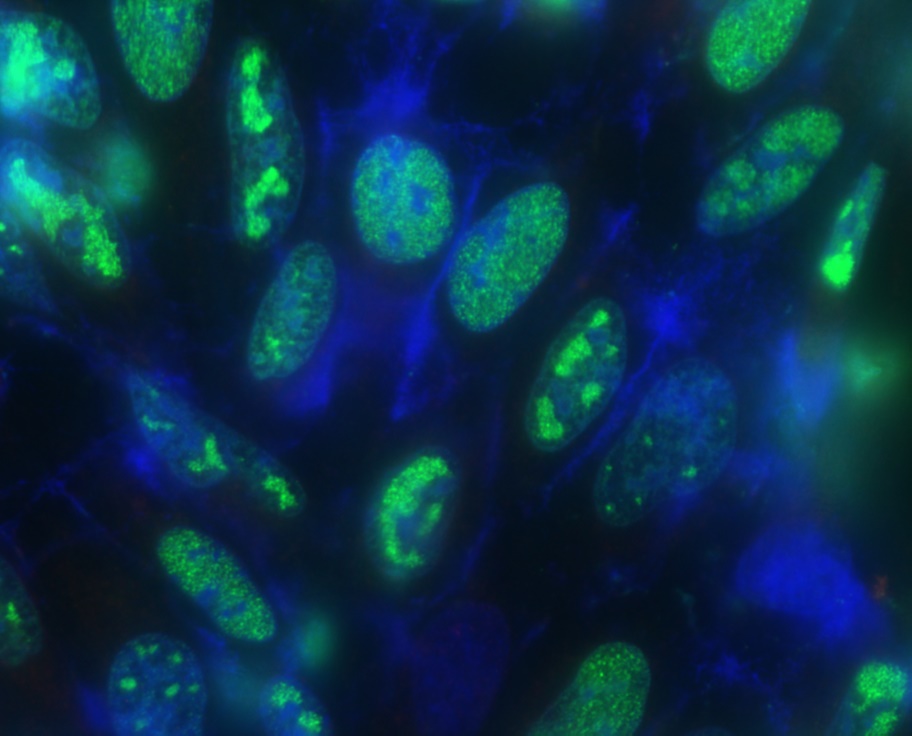

Supplement: Figure 2—figure supplement 1—source data 6. [file elife-85412-fig2-figsupp1-data6.zip › Figure 2-figure supplement 1-source data 6 Figure 2-figure supplement 1 J/All TIFF FILES/-Dox/6hr-2.jpg]

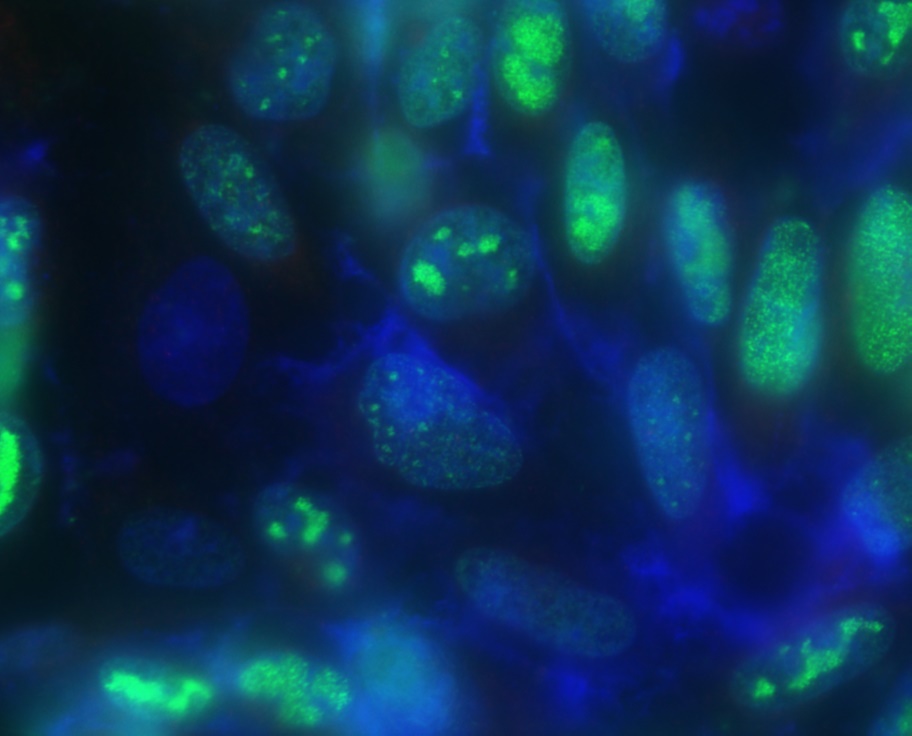

Supplement: Figure 2—figure supplement 1—source data 6. [file elife-85412-fig2-figsupp1-data6.zip › Figure 2-figure supplement 1-source data 6 Figure 2-figure supplement 1 J/All TIFF FILES/-Dox/6hr-3.jpg]

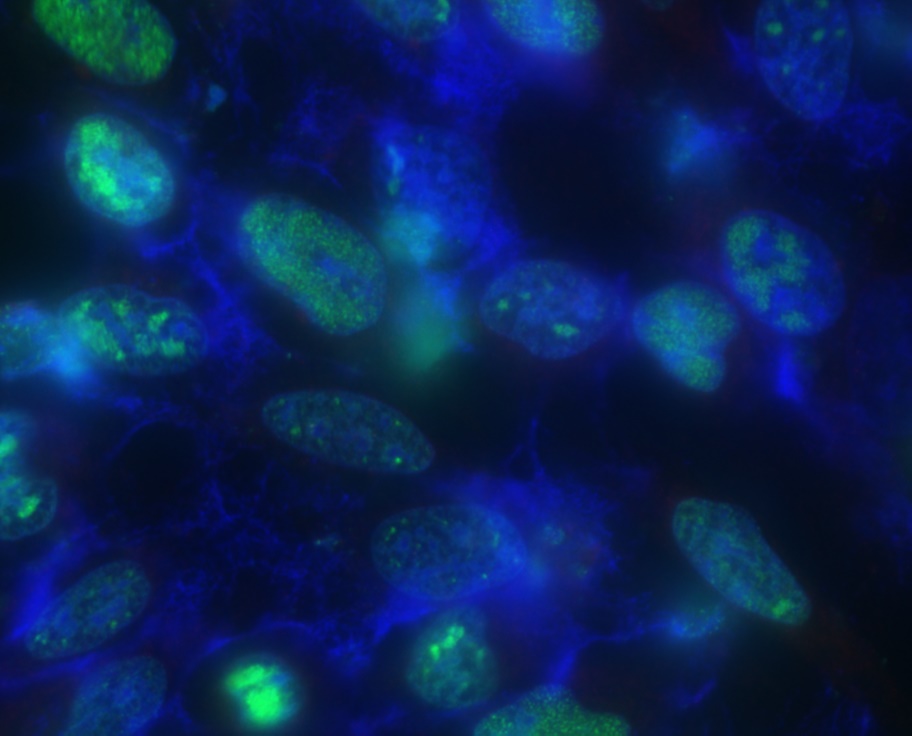

Supplement: Figure 2—figure supplement 1—source data 6. [file elife-85412-fig2-figsupp1-data6.zip › Figure 2-figure supplement 1-source data 6 Figure 2-figure supplement 1 J/All TIFF FILES/-Dox/6hr-4.jpg]

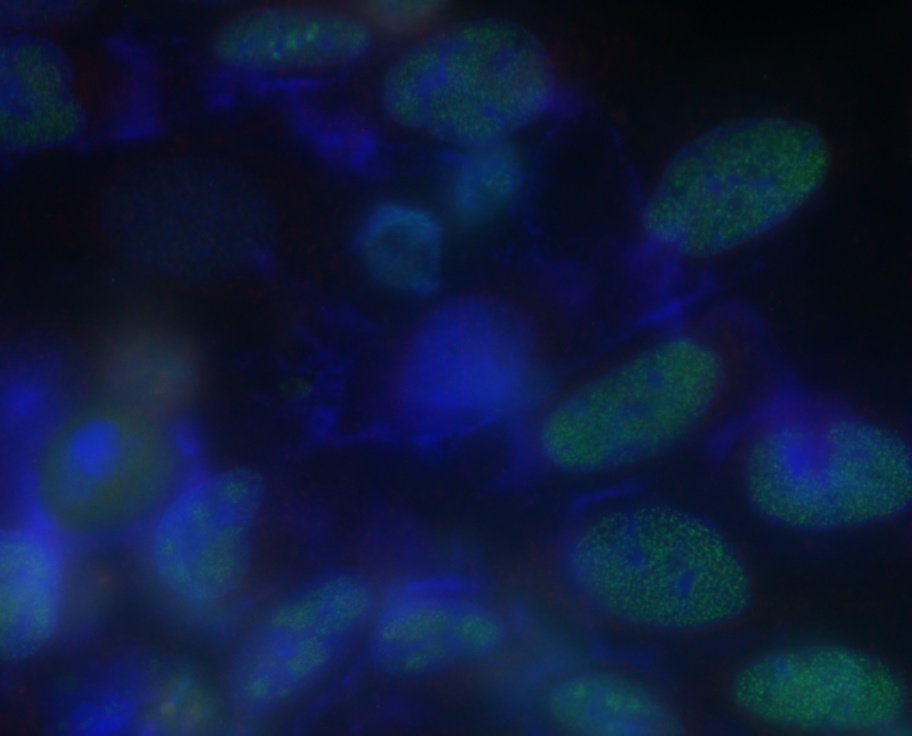

Supplement: Figure 2—figure supplement 1—source data 6. [file elife-85412-fig2-figsupp1-data6.zip › Figure 2-figure supplement 1-source data 6 Figure 2-figure supplement 1 J/All TIFF FILES/-Dox/8hr-1.jpg]

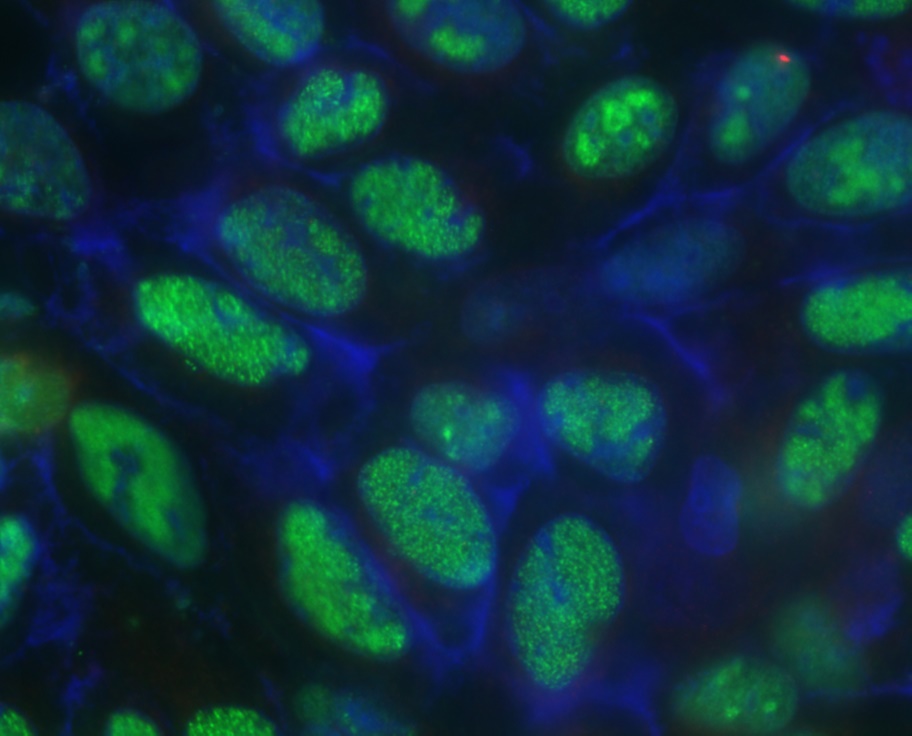

Supplement: Figure 2—figure supplement 1—source data 6. [file elife-85412-fig2-figsupp1-data6.zip › Figure 2-figure supplement 1-source data 6 Figure 2-figure supplement 1 J/All TIFF FILES/-Dox/8hr-2.jpg]

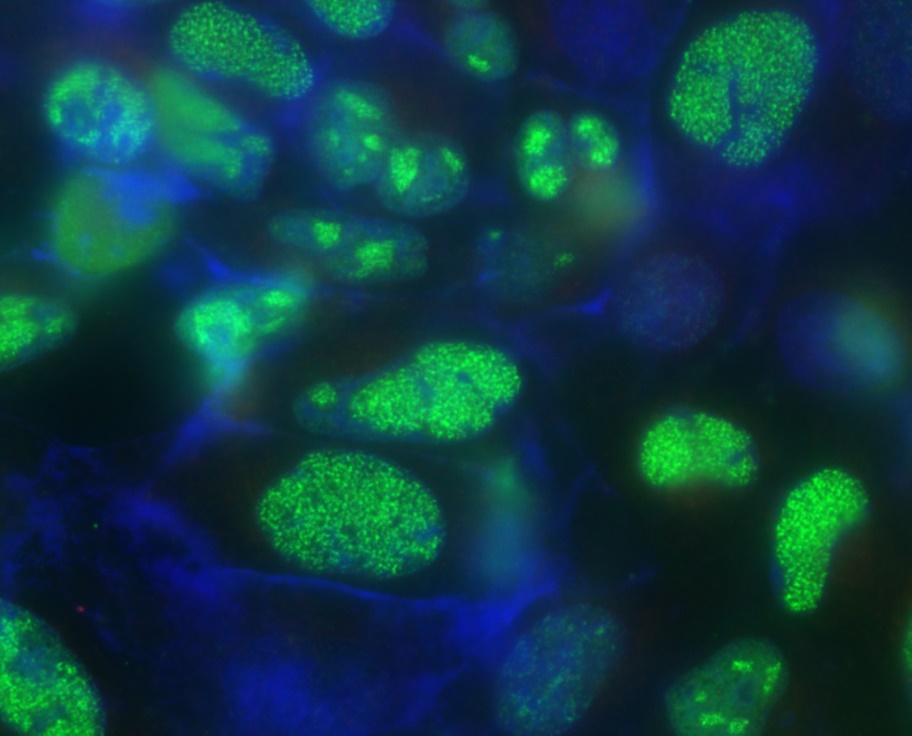

Supplement: Figure 2—figure supplement 1—source data 6. [file elife-85412-fig2-figsupp1-data6.zip › Figure 2-figure supplement 1-source data 6 Figure 2-figure supplement 1 J/All TIFF FILES/-Dox/8hr-3_.jpg]

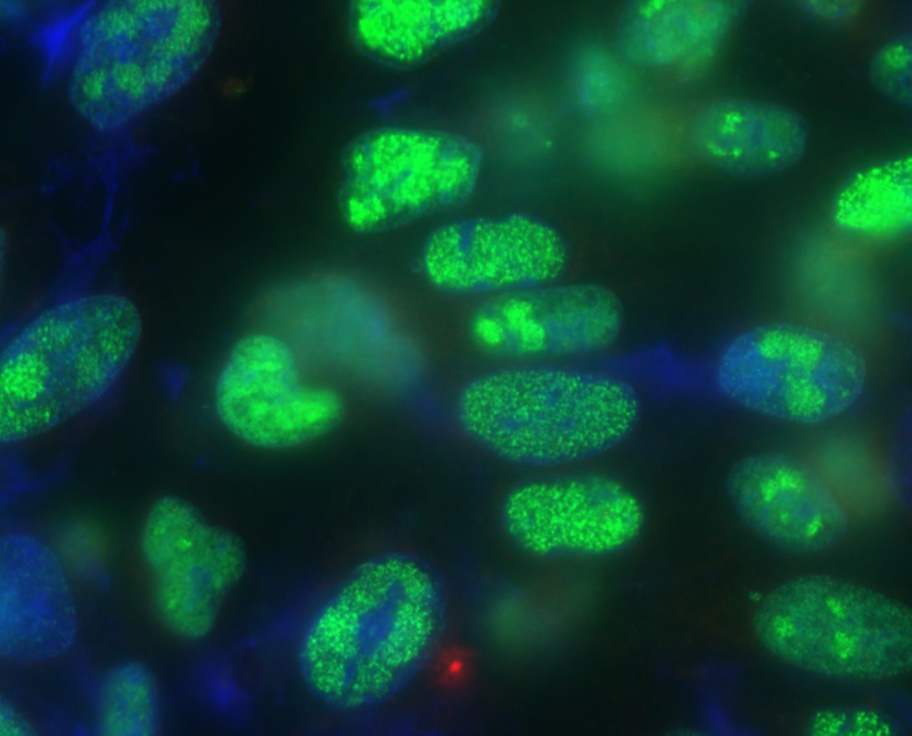

Supplement: Figure 2—figure supplement 1—source data 6. [file elife-85412-fig2-figsupp1-data6.zip › Figure 2-figure supplement 1-source data 6 Figure 2-figure supplement 1 J/All TIFF FILES/-Dox/8hr-4.jpg]

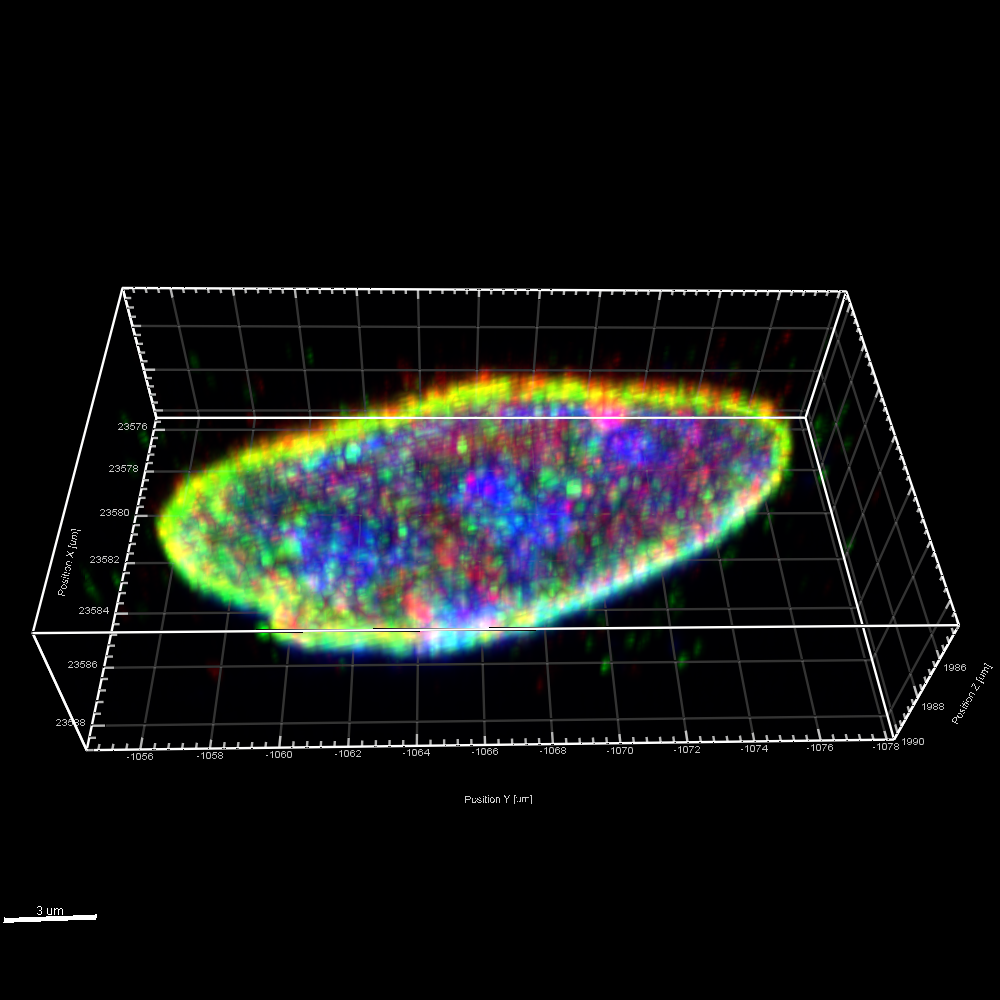

Supplement: Figure 3—source data 1. — This zip archive contains source data for original data collected for statistical analysis in panel D, virus titers in panels E and F, and processed images (TIF files) in panels A, C, and G. Original fluorescence images and confocal images in panels A, C, and G are deposited on DRYAD (https://doi.org/10.5061/dryad.vmcvdncxd). [file elife-85412-fig3-data1.zip › Figure 3 - source data 1 Figure 3 A/FLAG+NUP Mock .tif]

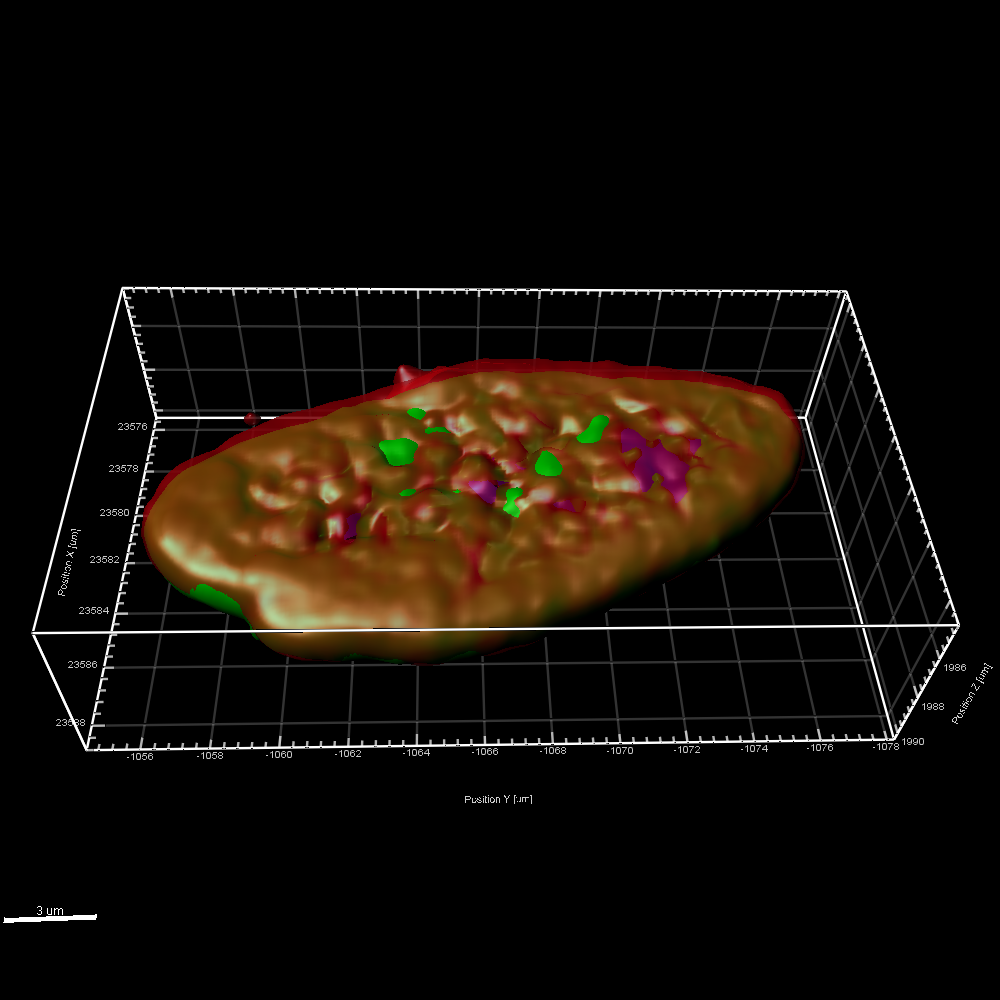

Supplement: Figure 3—source data 1. — This zip archive contains source data for original data collected for statistical analysis in panel D, virus titers in panels E and F, and processed images (TIF files) in panels A, C, and G. Original fluorescence images and confocal images in panels A, C, and G are deposited on DRYAD (https://doi.org/10.5061/dryad.vmcvdncxd). [file elife-85412-fig3-data1.zip › Figure 3 - source data 1 Figure 3 A/FLAG+NUP Mock 3D.tif]

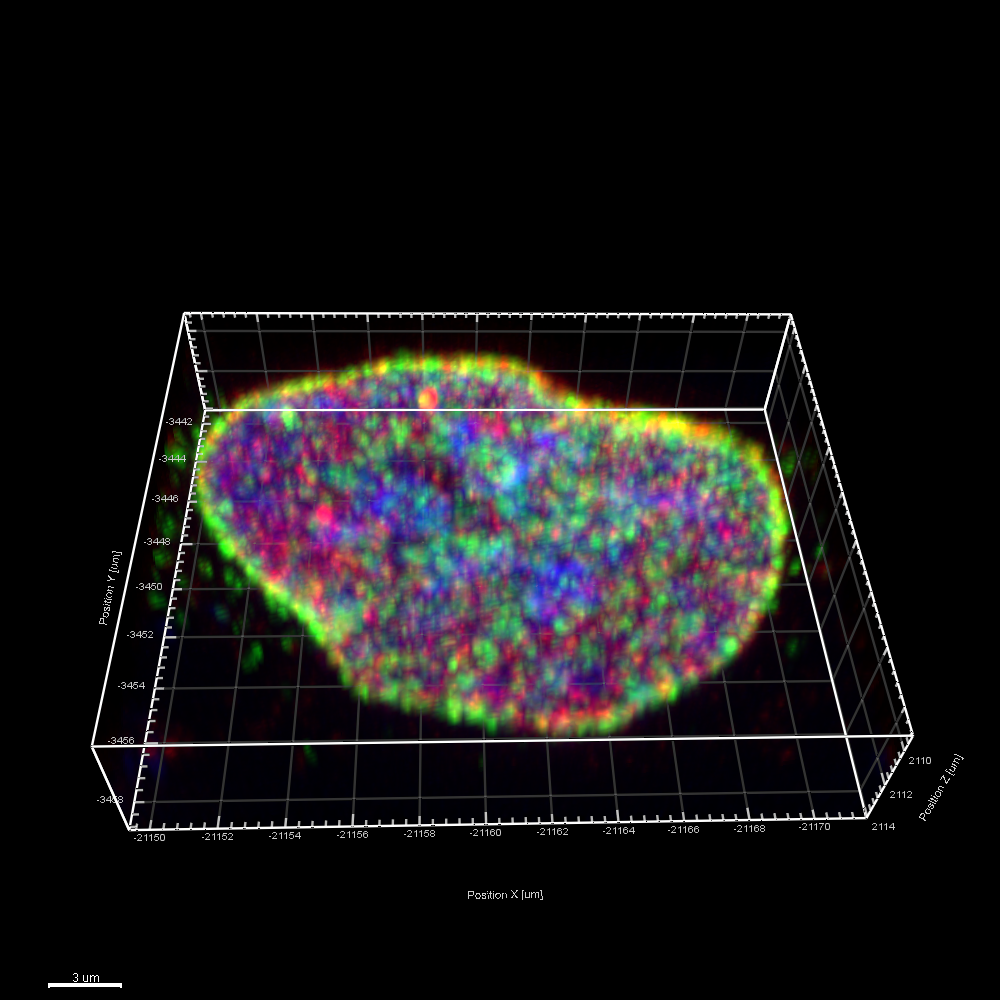

Supplement: Figure 3—source data 1. — This zip archive contains source data for original data collected for statistical analysis in panel D, virus titers in panels E and F, and processed images (TIF files) in panels A, C, and G. Original fluorescence images and confocal images in panels A, C, and G are deposited on DRYAD (https://doi.org/10.5061/dryad.vmcvdncxd). [file elife-85412-fig3-data1.zip › Figure 3 - source data 1 Figure 3 A/FLAG+NUP-MOi =5 3 hpi.tif]
