# Supplementary material for: A CRISPR-based rapid DNA repositioning strategy and the early intranuclear life of HSV-1
Source: eLife. 2023 Sep 13;12:e85412. doi: 10.7554/eLife.85412 (PMC10522339; doi:10.7554/eLife.85412)
Supplement: Figure 1—source data 2. [file elife-85412-fig1-data2.pdf]

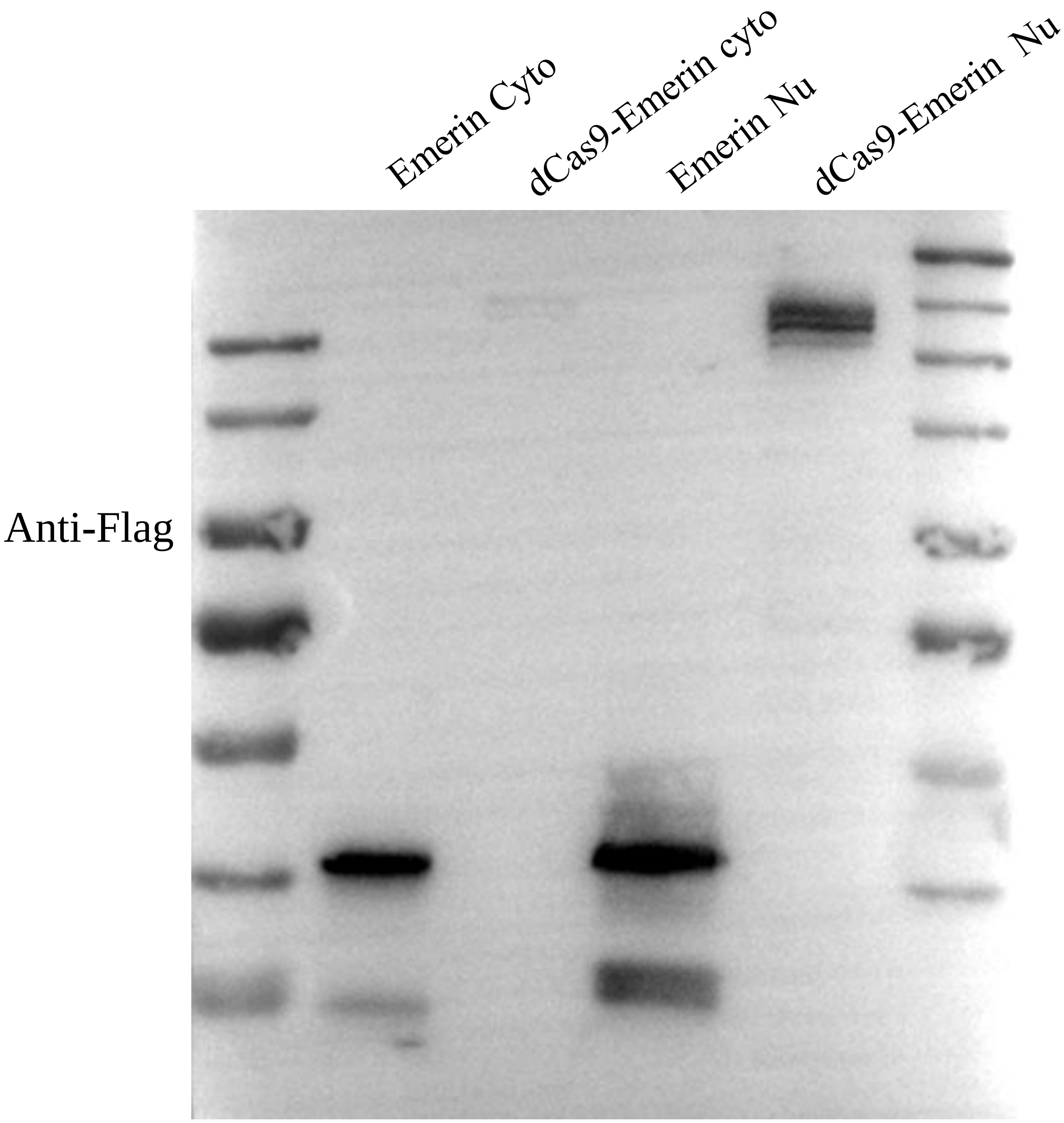
Emerin Cyto
dCas9-Emerin cyto
Emerin Nu
dCas9-Emerin Nu
Anti-Flag

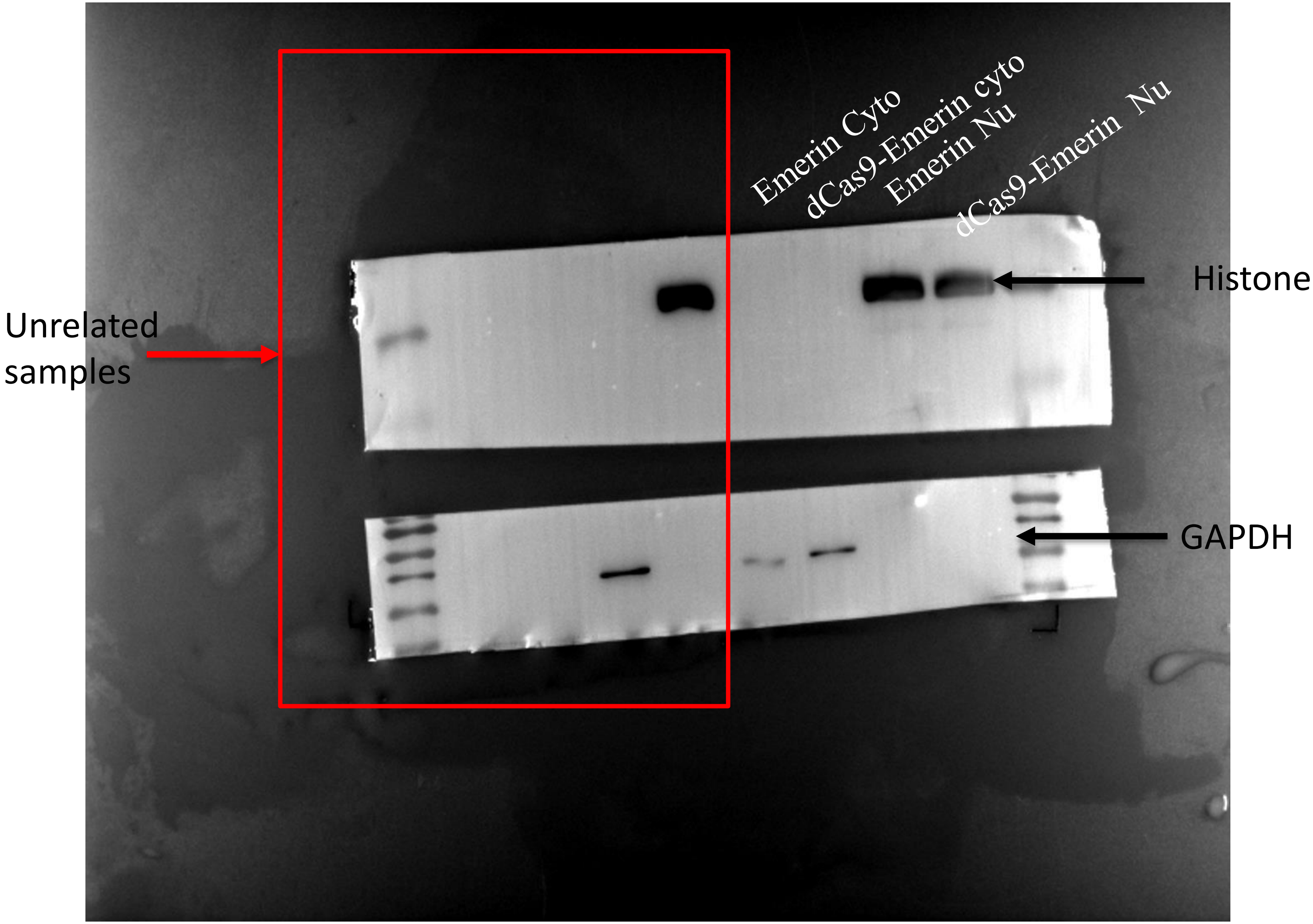

Emerin Cyto
dCas9-Emerin cyto
Emerin Nu
dCas9-Emerin Nu
Histone
Unrelated samples
GAPDH
