# Supplementary material for: A CRISPR-based rapid DNA repositioning strategy and the early intranuclear life of HSV-1
Source: eLife. 2023 Sep 13;12:e85412. doi: 10.7554/eLife.85412 (PMC10522339; doi:10.7554/eLife.85412)
Supplement: Figure 2—figure supplement 1—source data 5. [file elife-85412-fig2-figsupp1-data5.pdf]

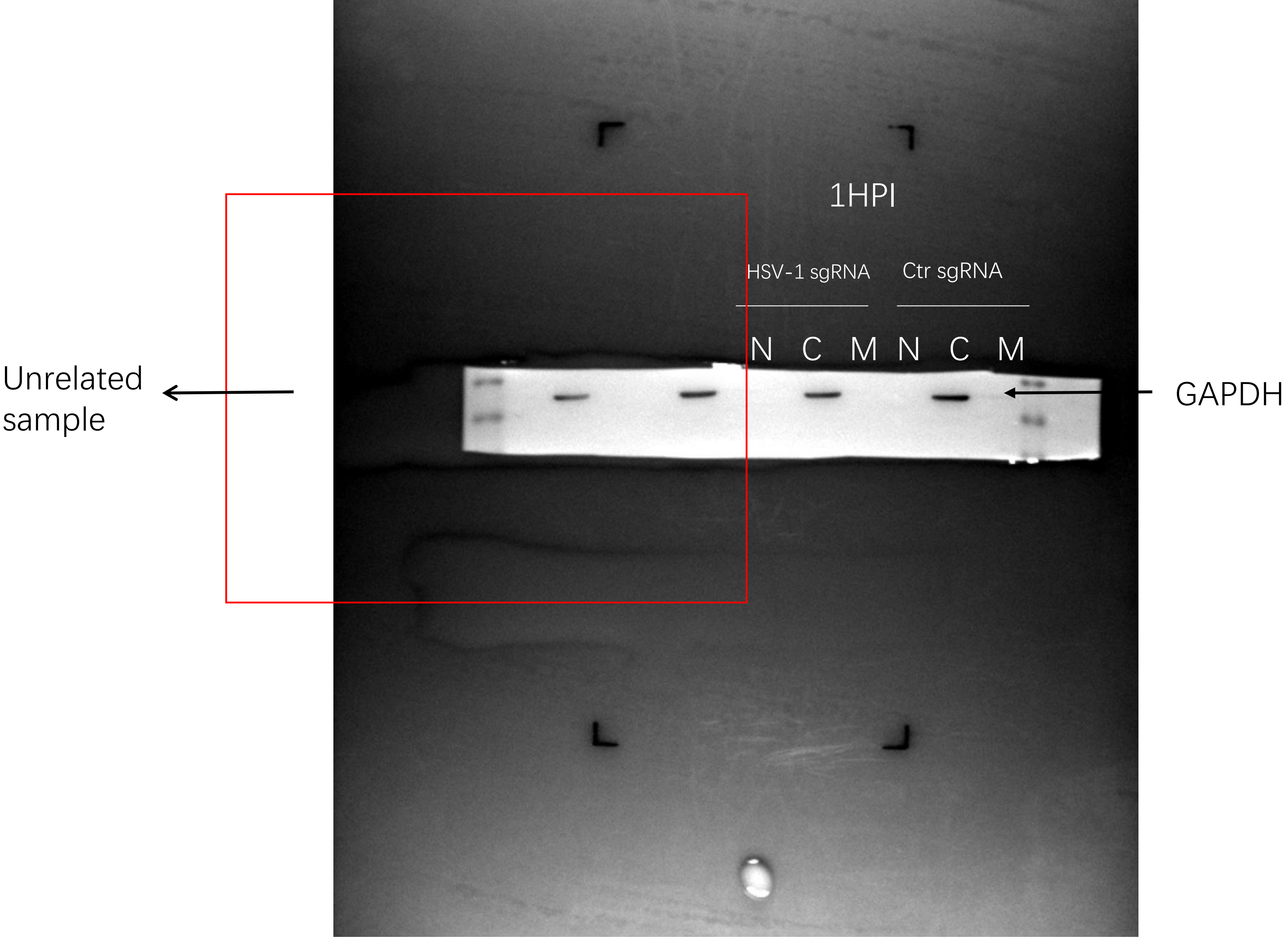
1HPI
HSV-1 sgRNA
Ctr sgRNA
N C M N C M
Unrelated sample
GAPDH

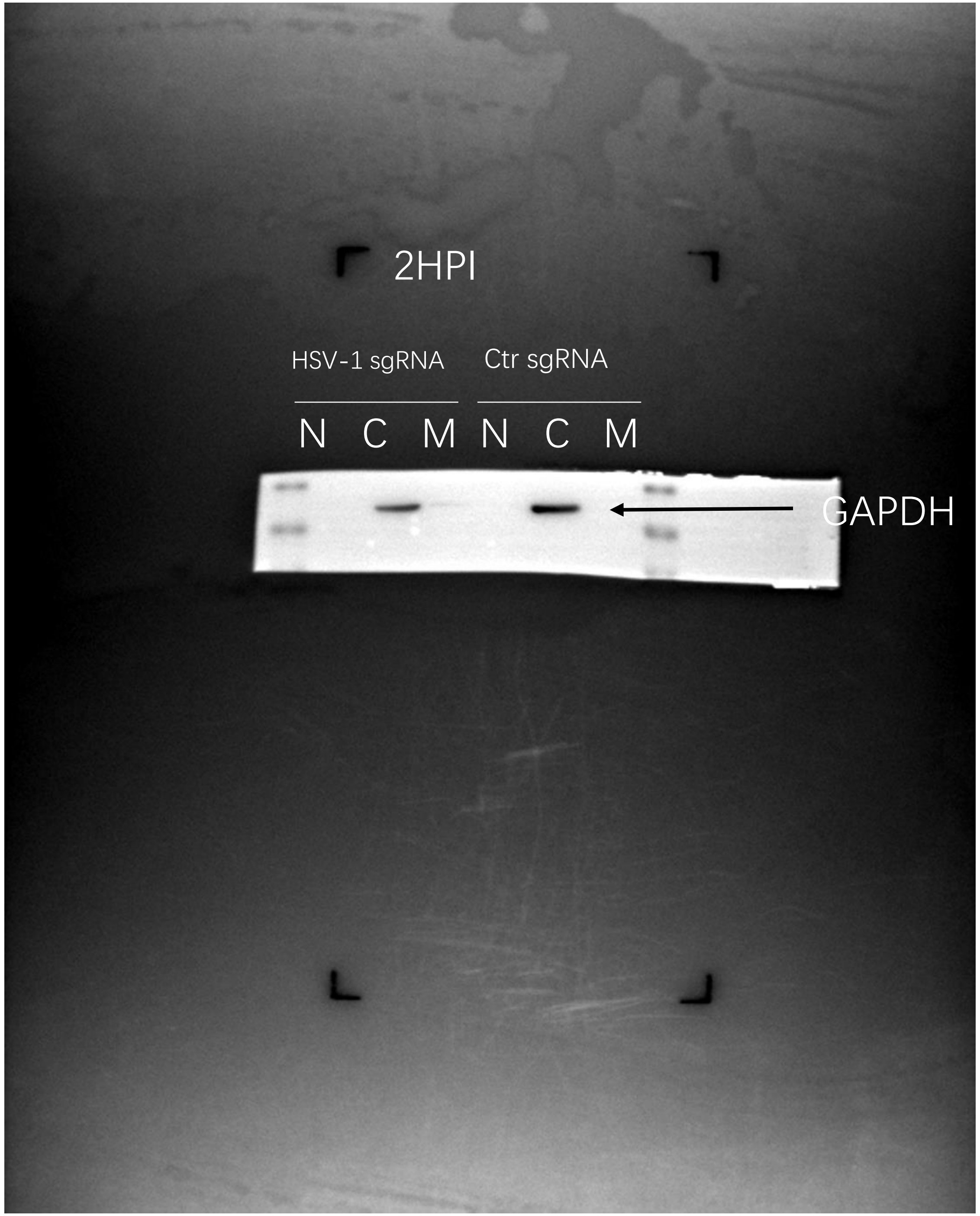

2HPI
HSV-1 sgRNA
Ctr sgRNA
N C M N C M
GAPDH

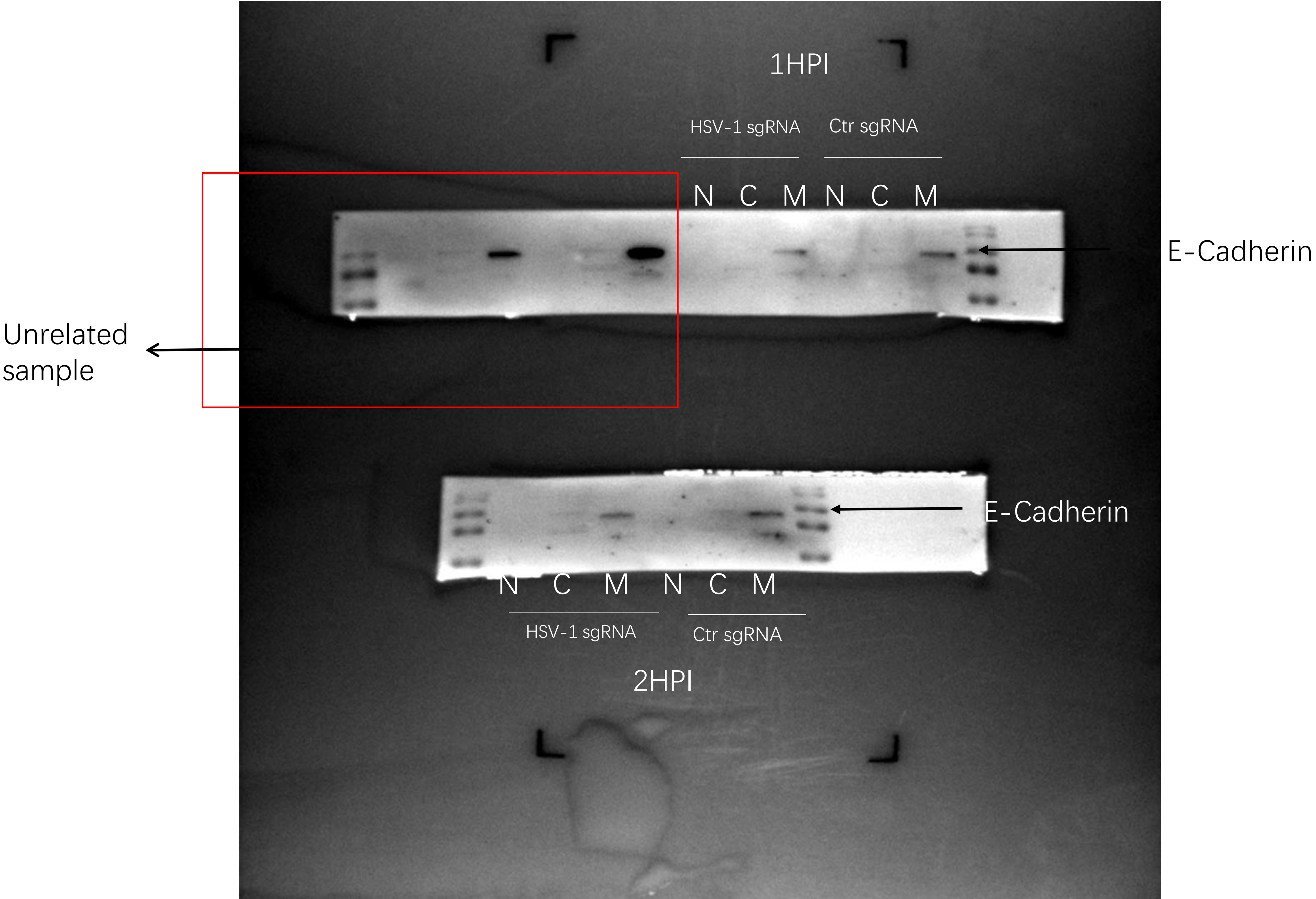
1HPI
HSV-1 sgRNA
Ctr sgRNA
N C M N C M
E-Cadherin
Unrelated sample
E-Cadherin
N C M N C M
HSV-1 sgRNA
Ctr sgRNA
2HPI

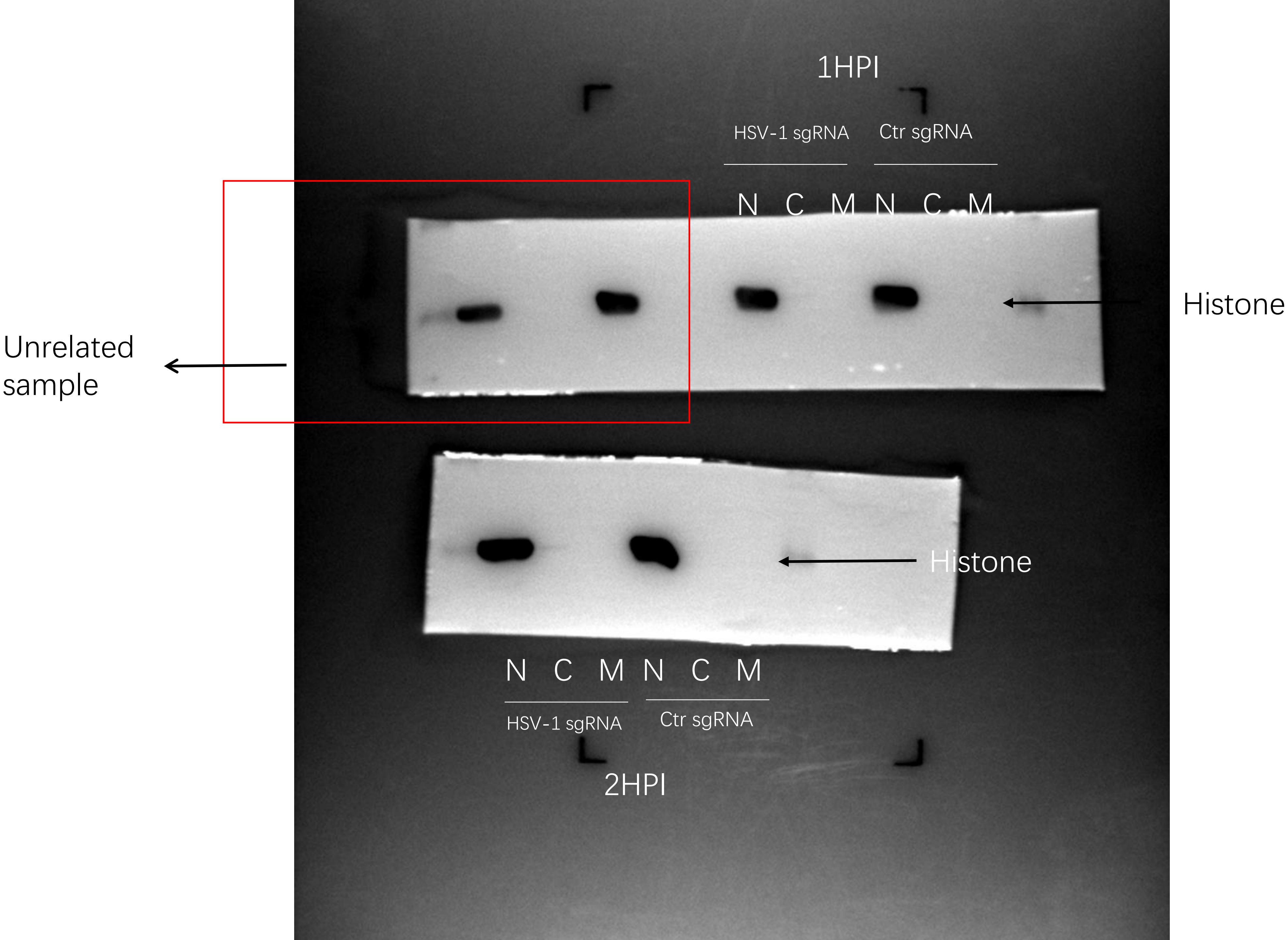
1HPI
HSV-1 sgRNA
Ctr sgRNA
N C M N C M
Histone
Unrelated sample
Histone
N C M N C M
HSV-1 sgRNA
Ctr sgRNA
2HPI
