# Supplementary figures and images for: A CRISPR-based rapid DNA repositioning strategy and the early intranuclear life of HSV-1 (part 2 of 2)
Source: eLife. 2023 Sep 13;12:e85412. doi: 10.7554/eLife.85412 (PMC10522339; doi:10.7554/eLife.85412)

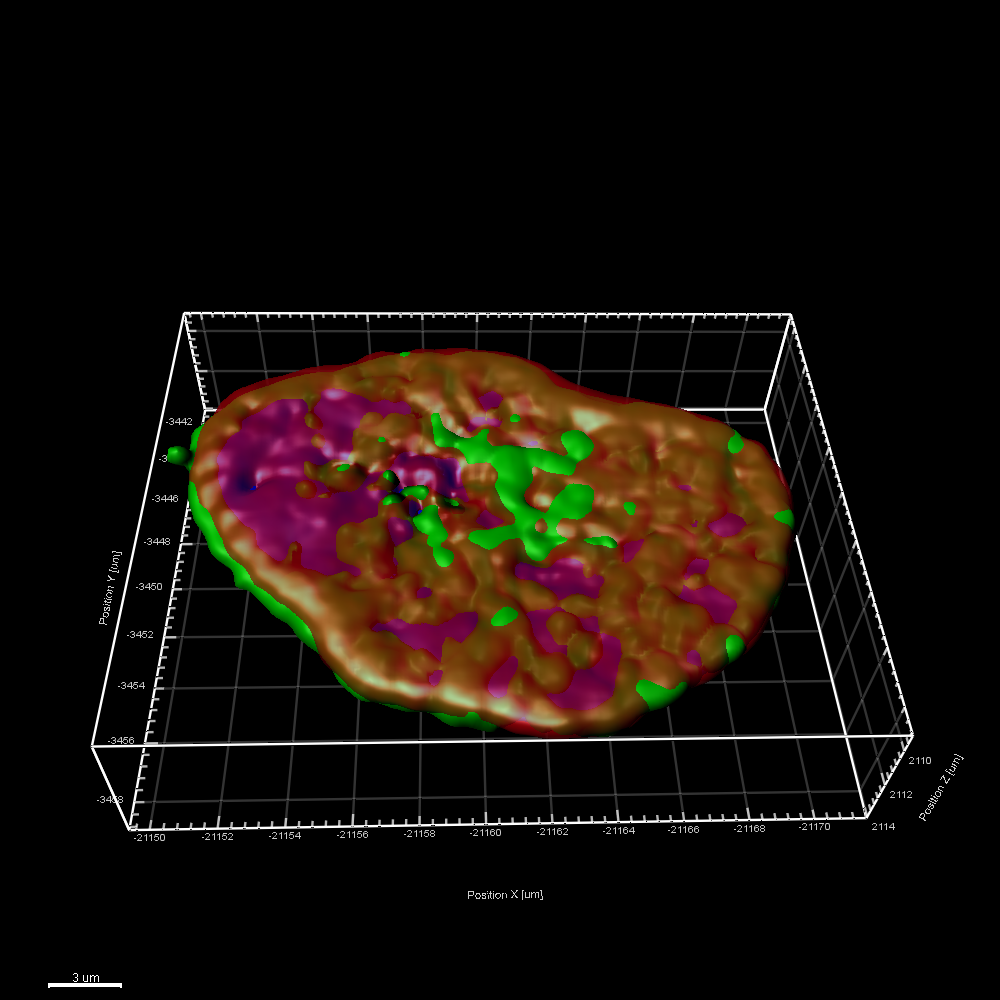

Supplement: Figure 3—source data 1. — This zip archive contains source data for original data collected for statistical analysis in panel D, virus titers in panels E and F, and processed images (TIF files) in panels A, C, and G. Original fluorescence images and confocal images in panels A, C, and G are deposited on DRYAD (https://doi.org/10.5061/dryad.vmcvdncxd). [file elife-85412-fig3-data1.zip › Figure 3 - source data 1 Figure 3 A/FLAG+NUP-MOi =5 3hpi 3D.tif]

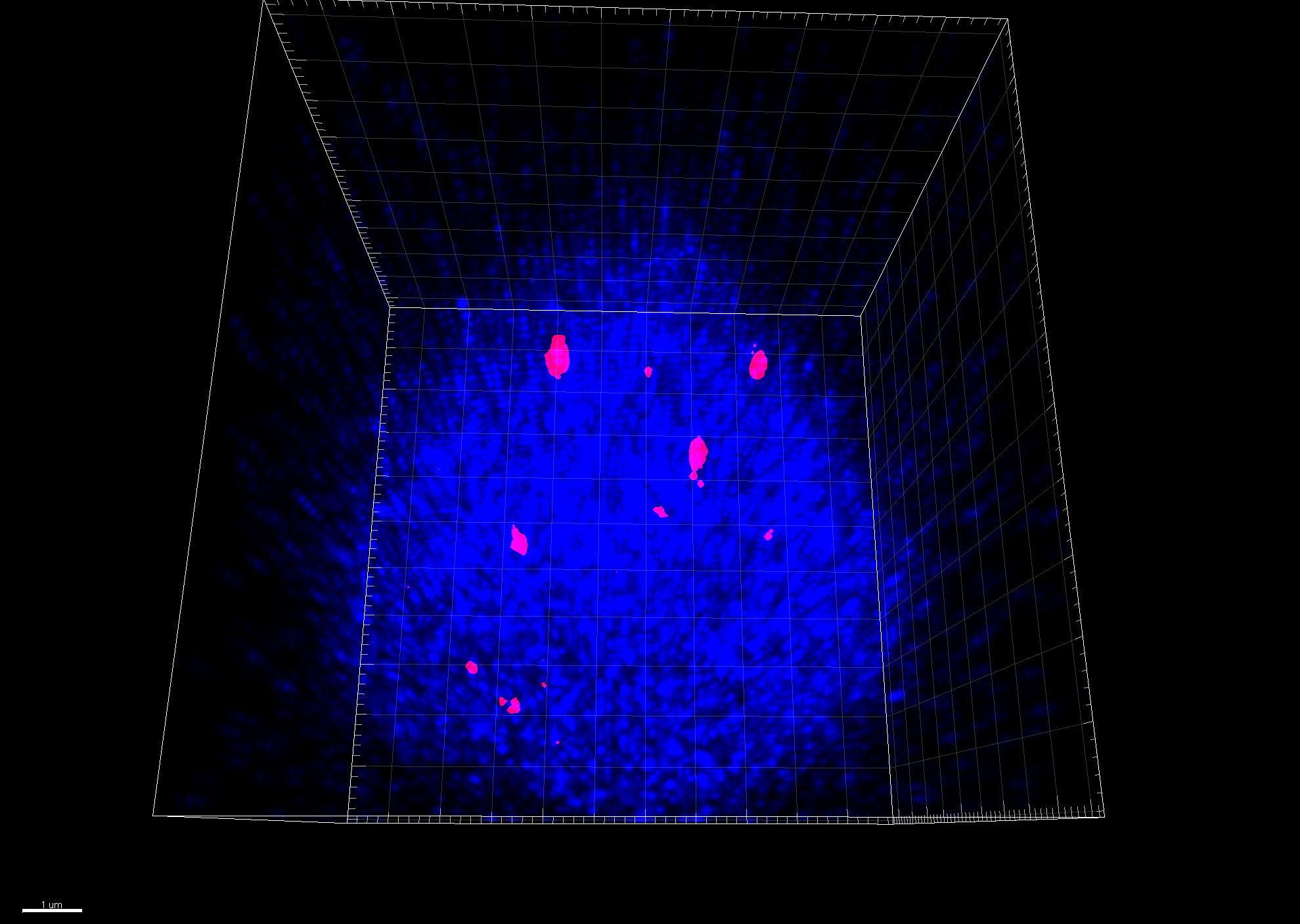

Supplement: Figure 3—source data 2. [file elife-85412-fig3-data2.zip › Figure 3 - source data 2 Figure 3 C/figre 3c-2-ctr sgRNA.tif]

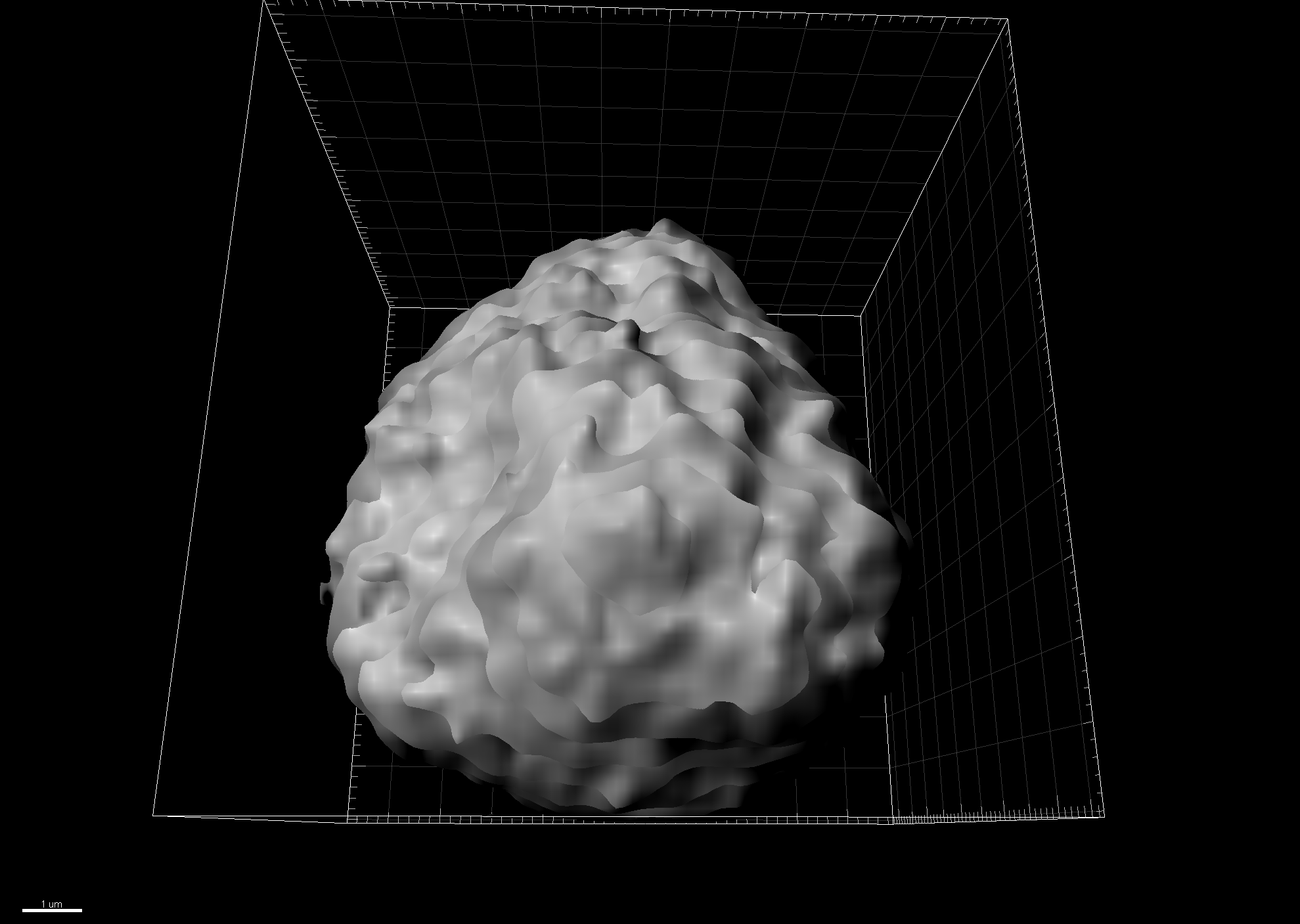

Supplement: Figure 3—source data 2. [file elife-85412-fig3-data2.zip › Figure 3 - source data 2 Figure 3 C/figre 3c-2-ctr sgRNA-3D.tif]

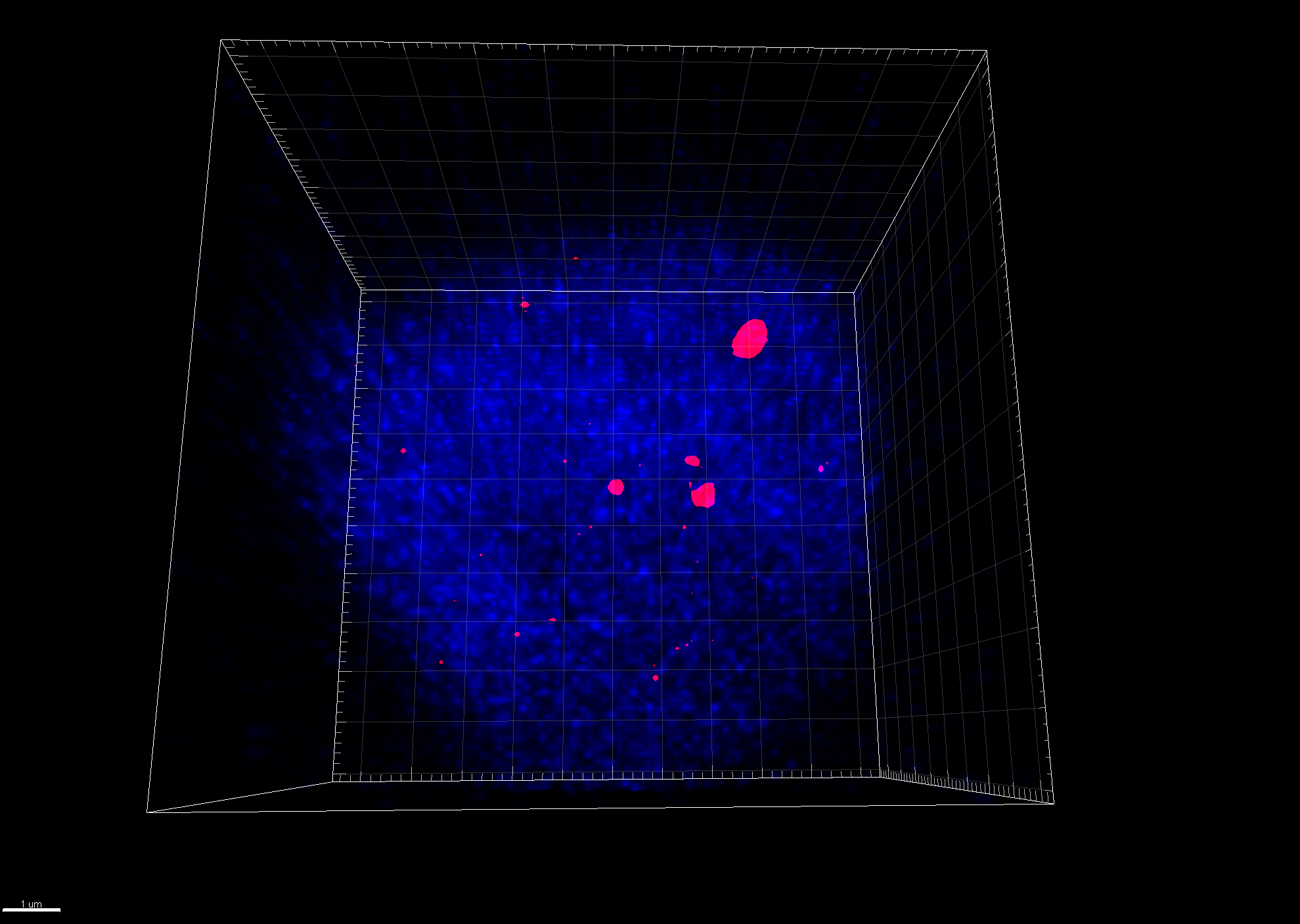

Supplement: Figure 3—source data 2. [file elife-85412-fig3-data2.zip › Figure 3 - source data 2 Figure 3 C/figure 3c-0.5-ctr sgRNA.tif]

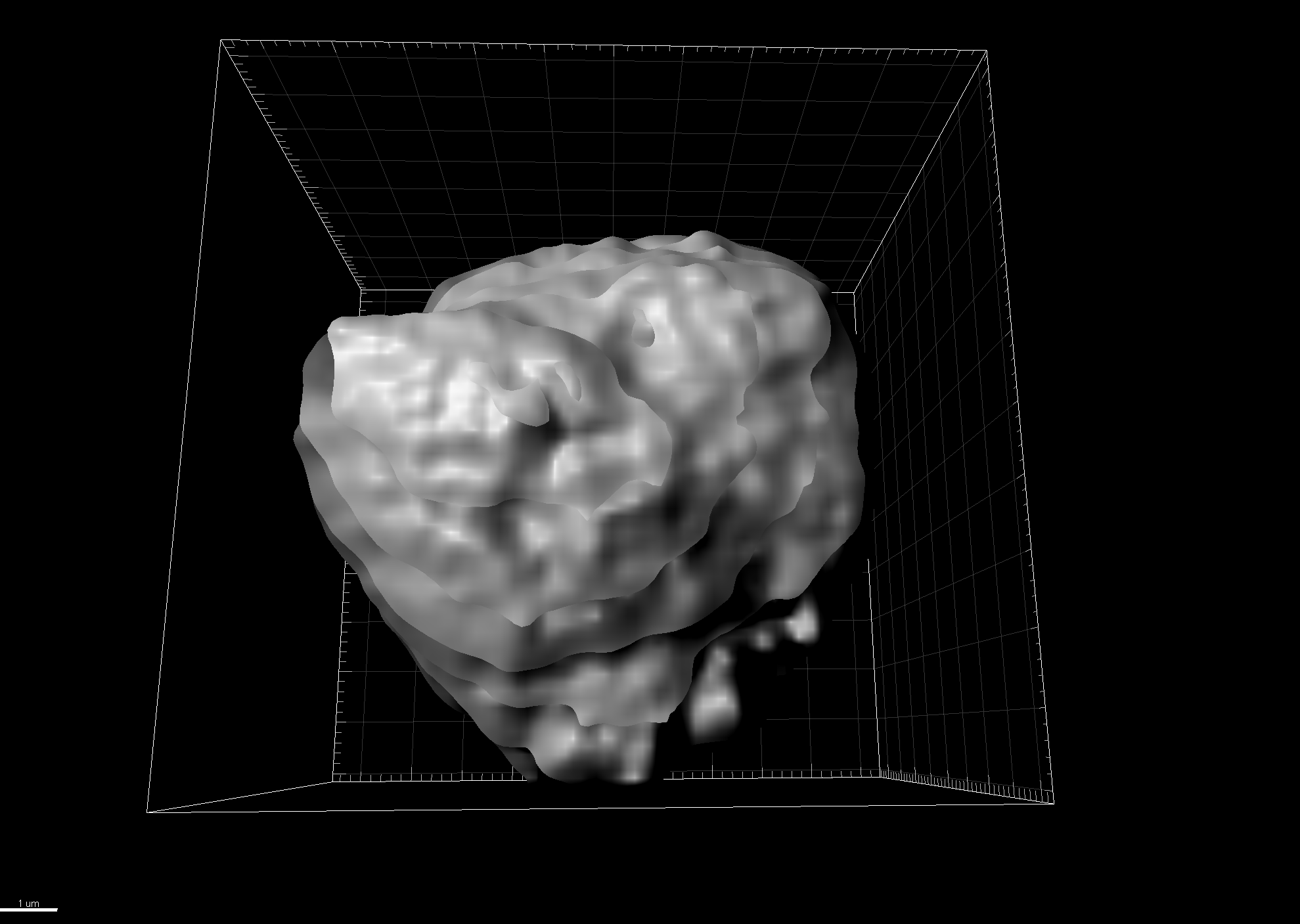

Supplement: Figure 3—source data 2. [file elife-85412-fig3-data2.zip › Figure 3 - source data 2 Figure 3 C/figure 3c-0.5-ctr sgRNA-3D.tif]

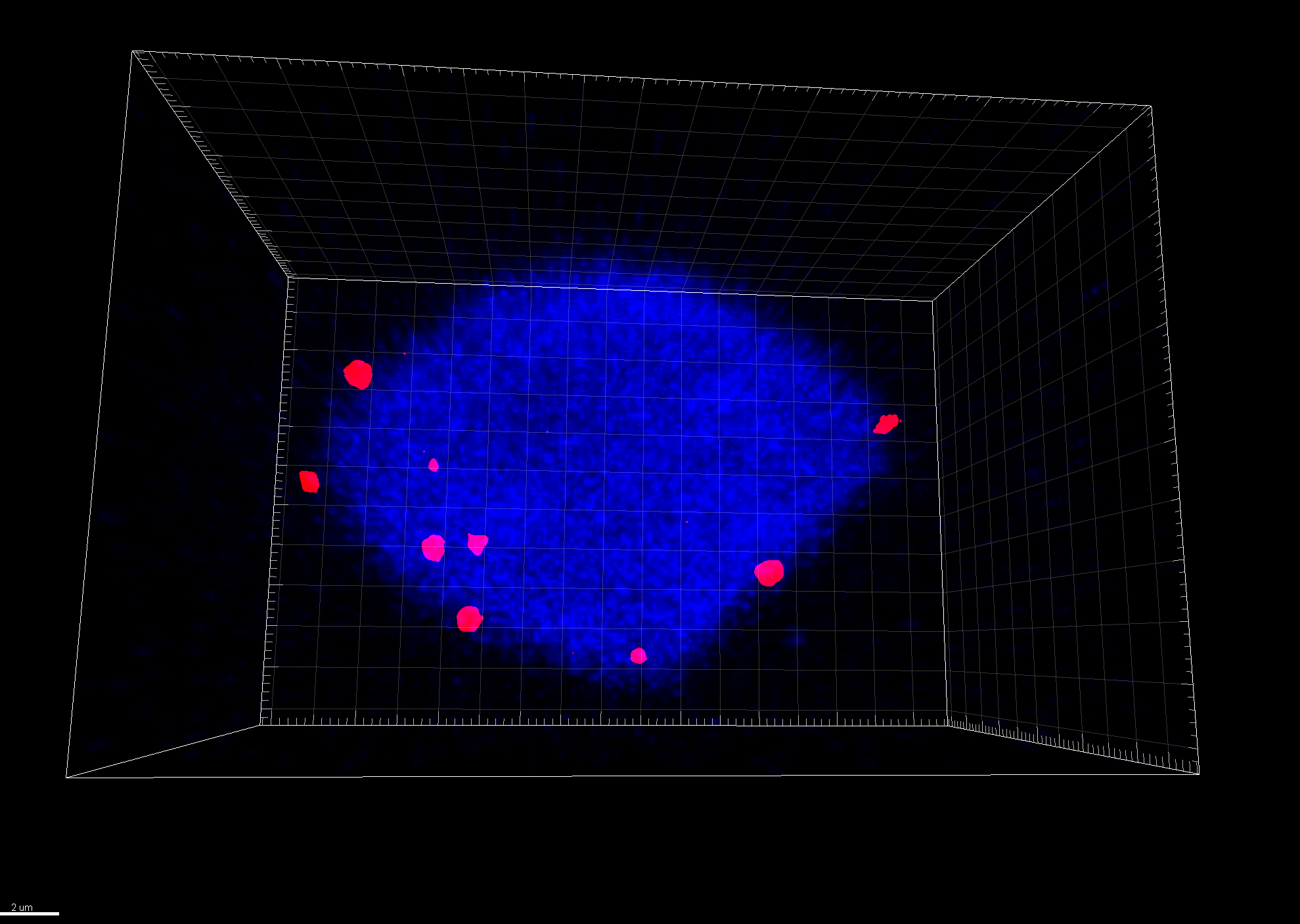

Supplement: Figure 3—source data 2. [file elife-85412-fig3-data2.zip › Figure 3 - source data 2 Figure 3 C/figure 3c-0.5-HSV-1-sgRNA.tif]

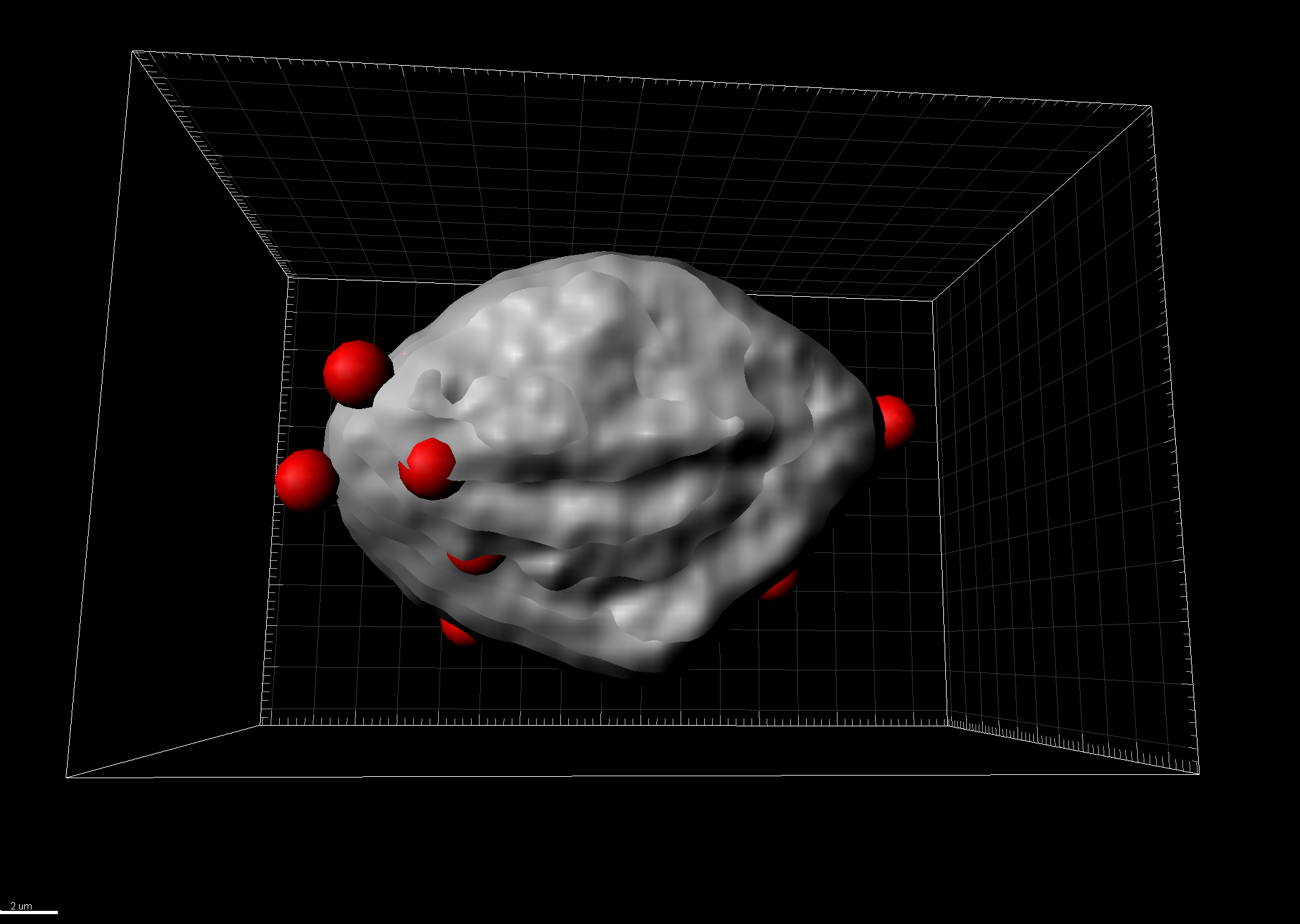

Supplement: Figure 3—source data 2. [file elife-85412-fig3-data2.zip › Figure 3 - source data 2 Figure 3 C/figure 3c-0.5-HSV-1-sgRNA-3D.tif]

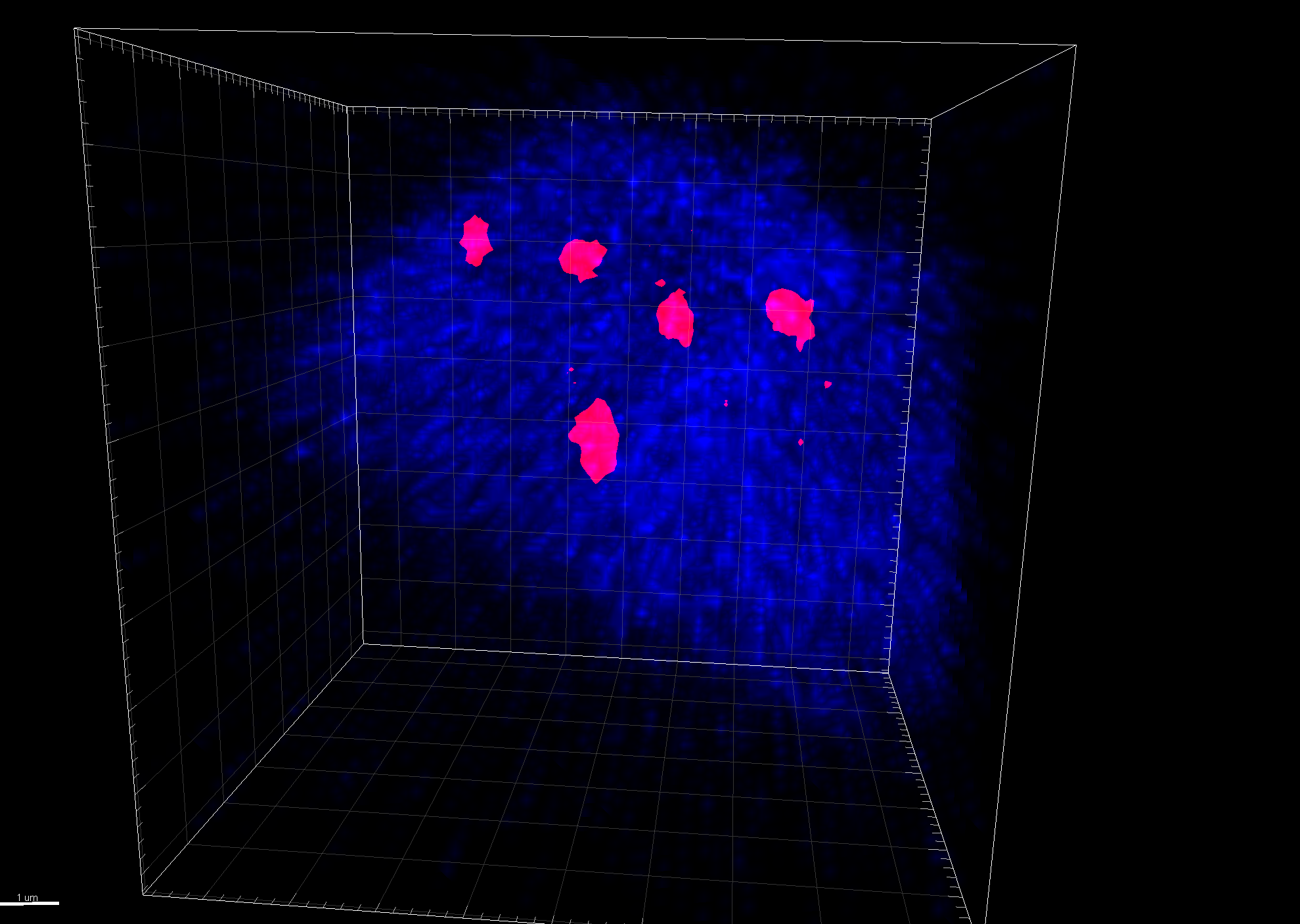

Supplement: Figure 3—source data 2. [file elife-85412-fig3-data2.zip › Figure 3 - source data 2 Figure 3 C/figure 3c-1-ctr sgRNA.tif]

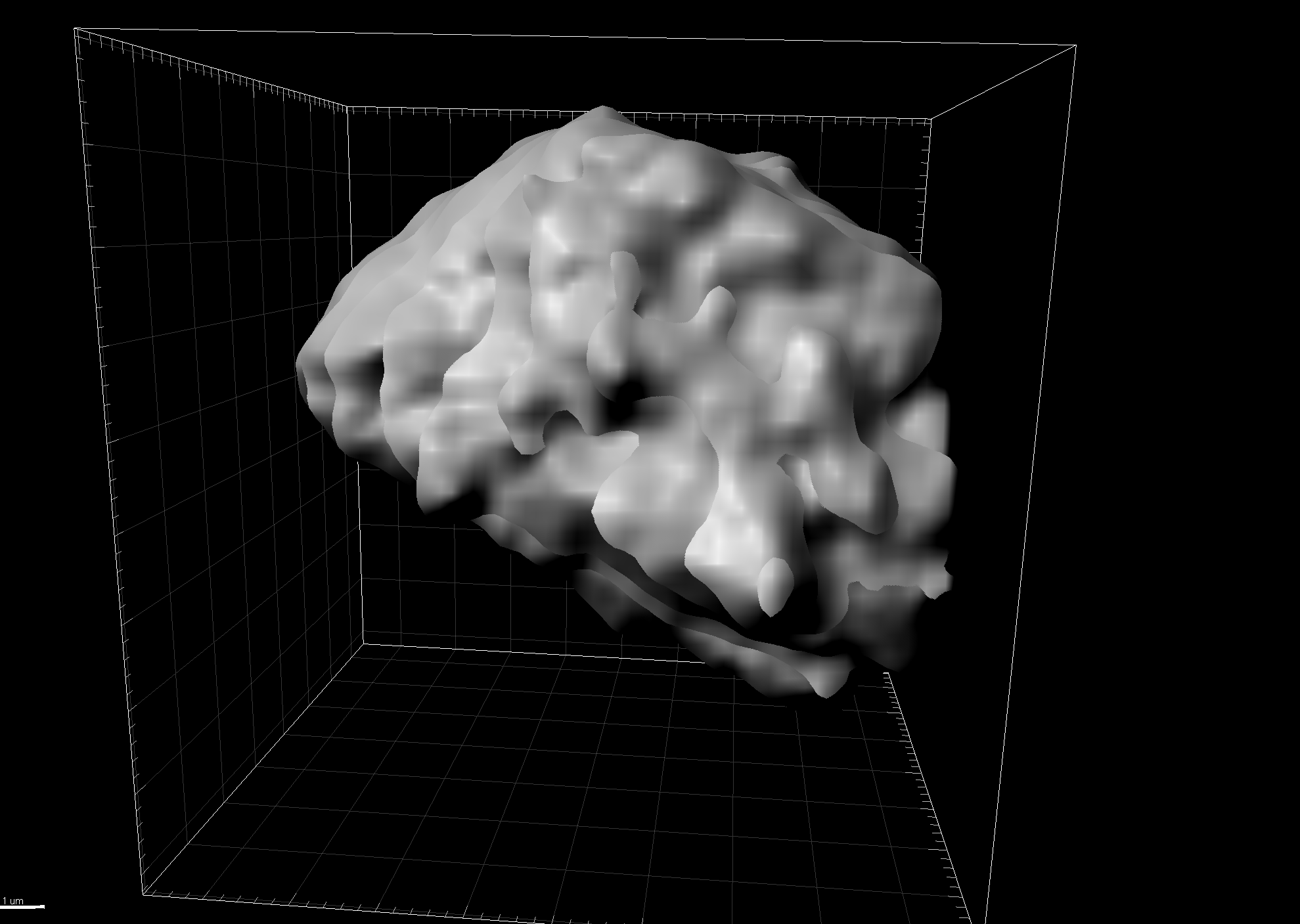

Supplement: Figure 3—source data 2. [file elife-85412-fig3-data2.zip › Figure 3 - source data 2 Figure 3 C/figure 3c-1-ctr sgRNA-3D.tif]

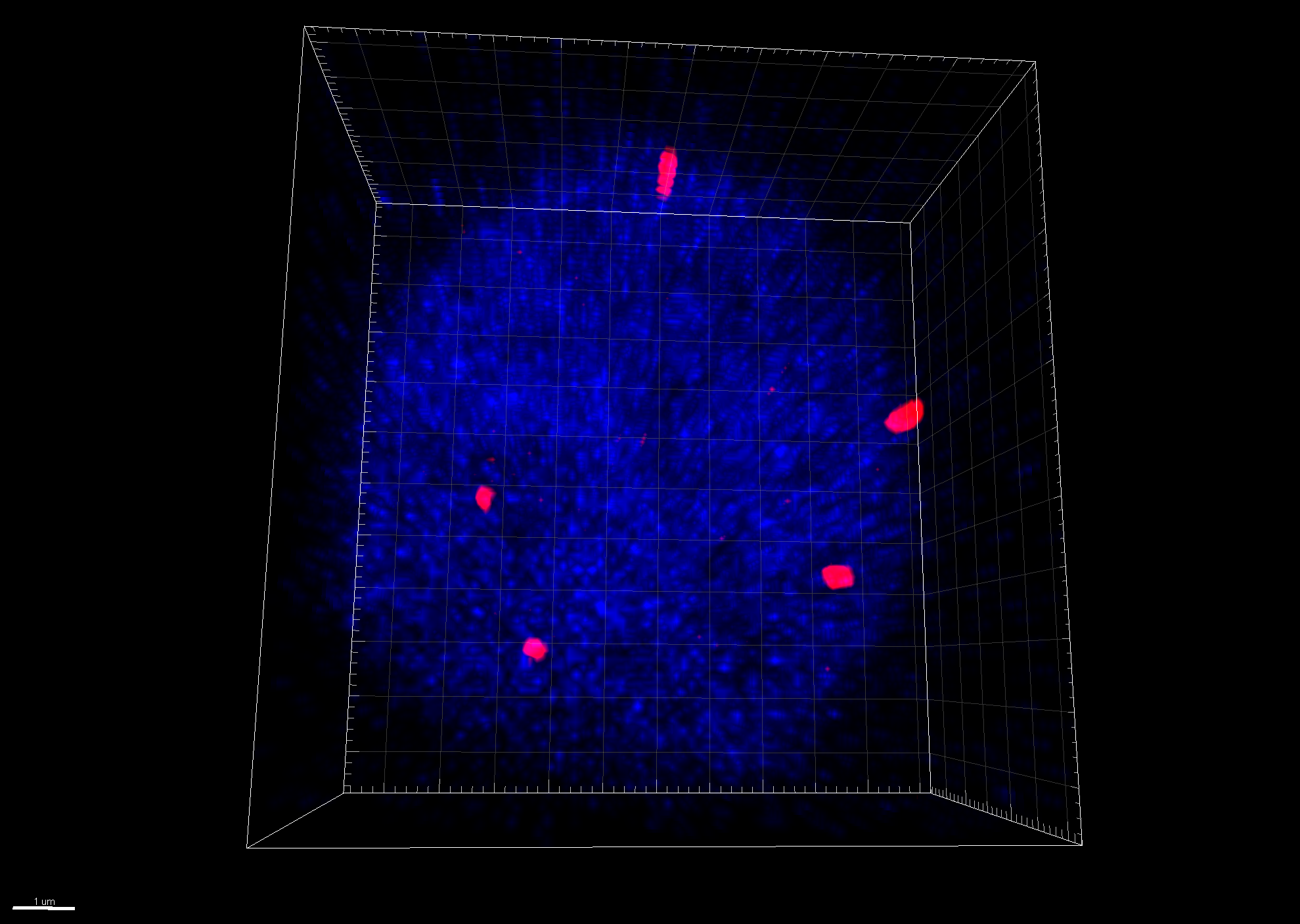

Supplement: Figure 3—source data 2. [file elife-85412-fig3-data2.zip › Figure 3 - source data 2 Figure 3 C/figure 3c-1-HSV-1 sgRNA.tif]

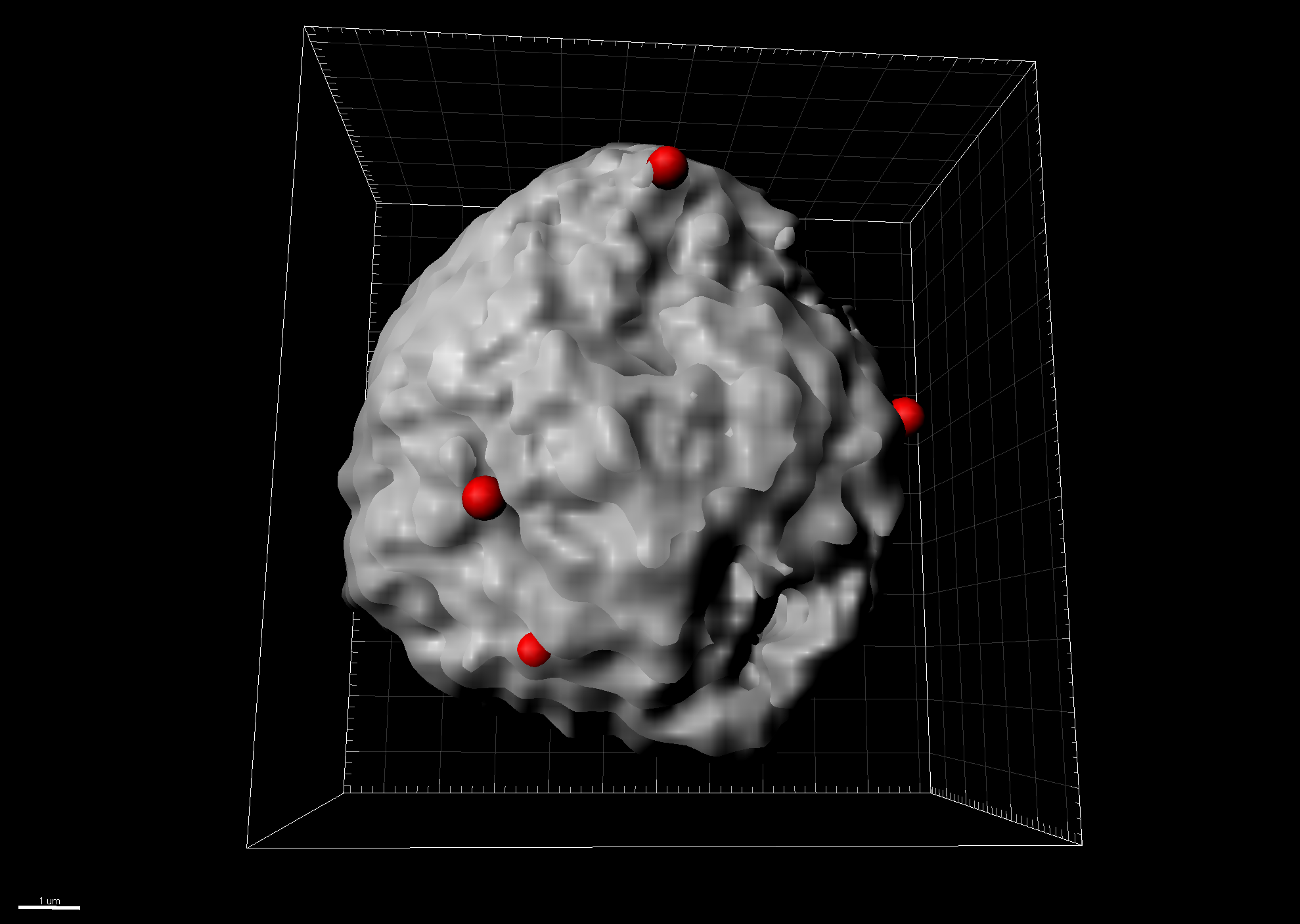

Supplement: Figure 3—source data 2. [file elife-85412-fig3-data2.zip › Figure 3 - source data 2 Figure 3 C/figure 3c-1-HSV-1 sgRNA-3D.tif]

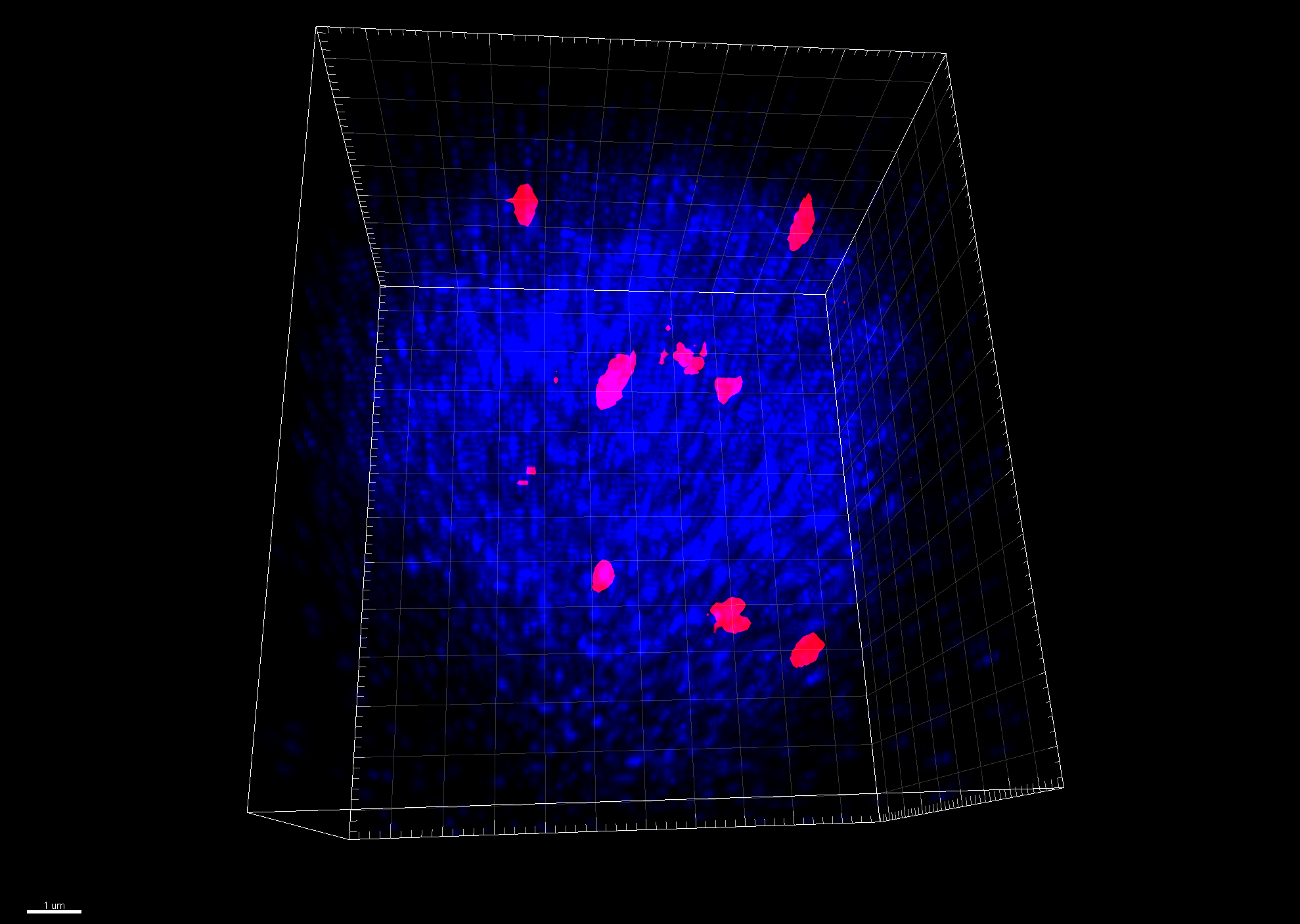

Supplement: Figure 3—source data 2. [file elife-85412-fig3-data2.zip › Figure 3 - source data 2 Figure 3 C/figure 3c-2-HSV-1 sgRNA.tif]

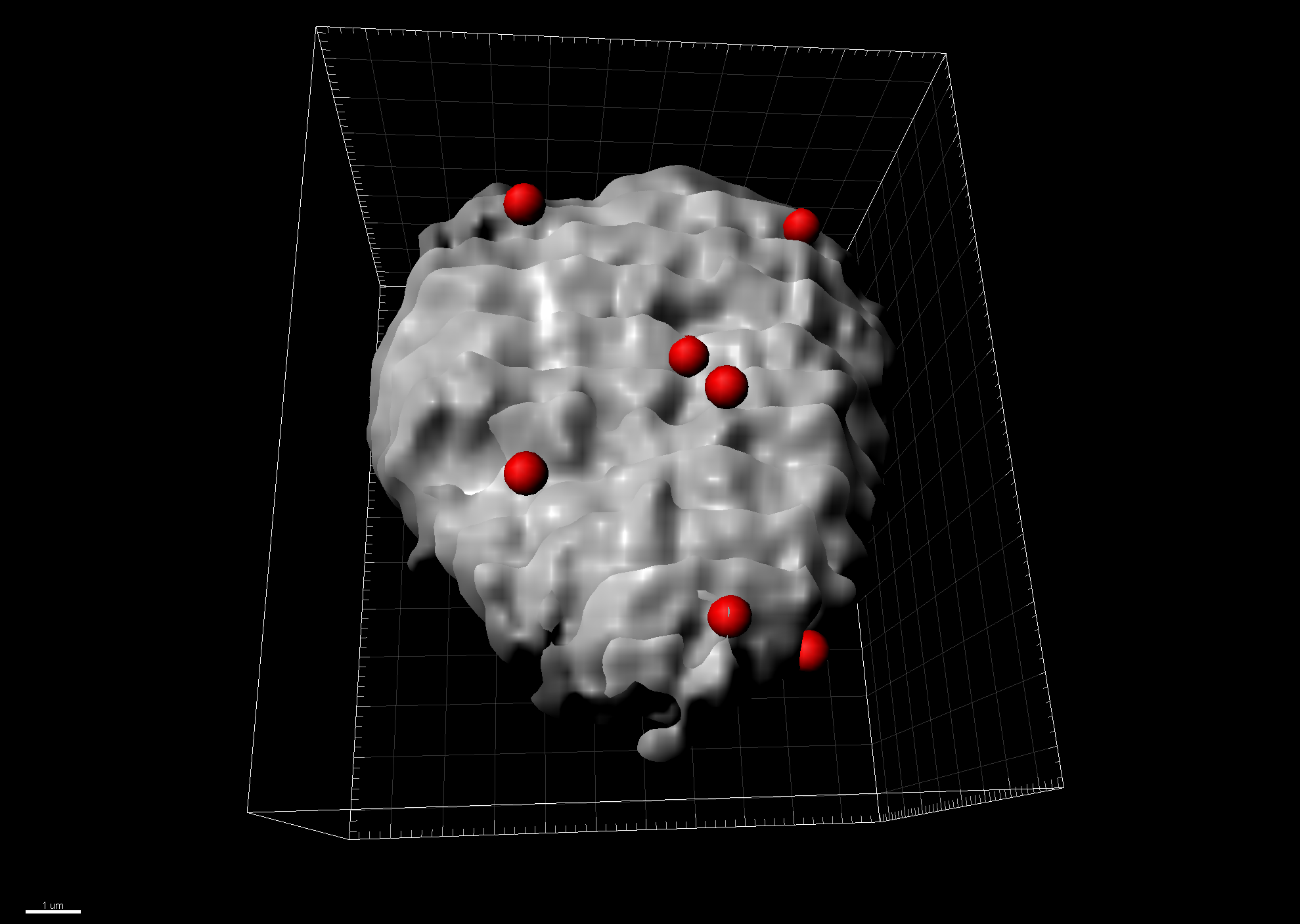

Supplement: Figure 3—source data 2. [file elife-85412-fig3-data2.zip › Figure 3 - source data 2 Figure 3 C/figure 3c-2-HSV-1 sgRNA-3D.tif]

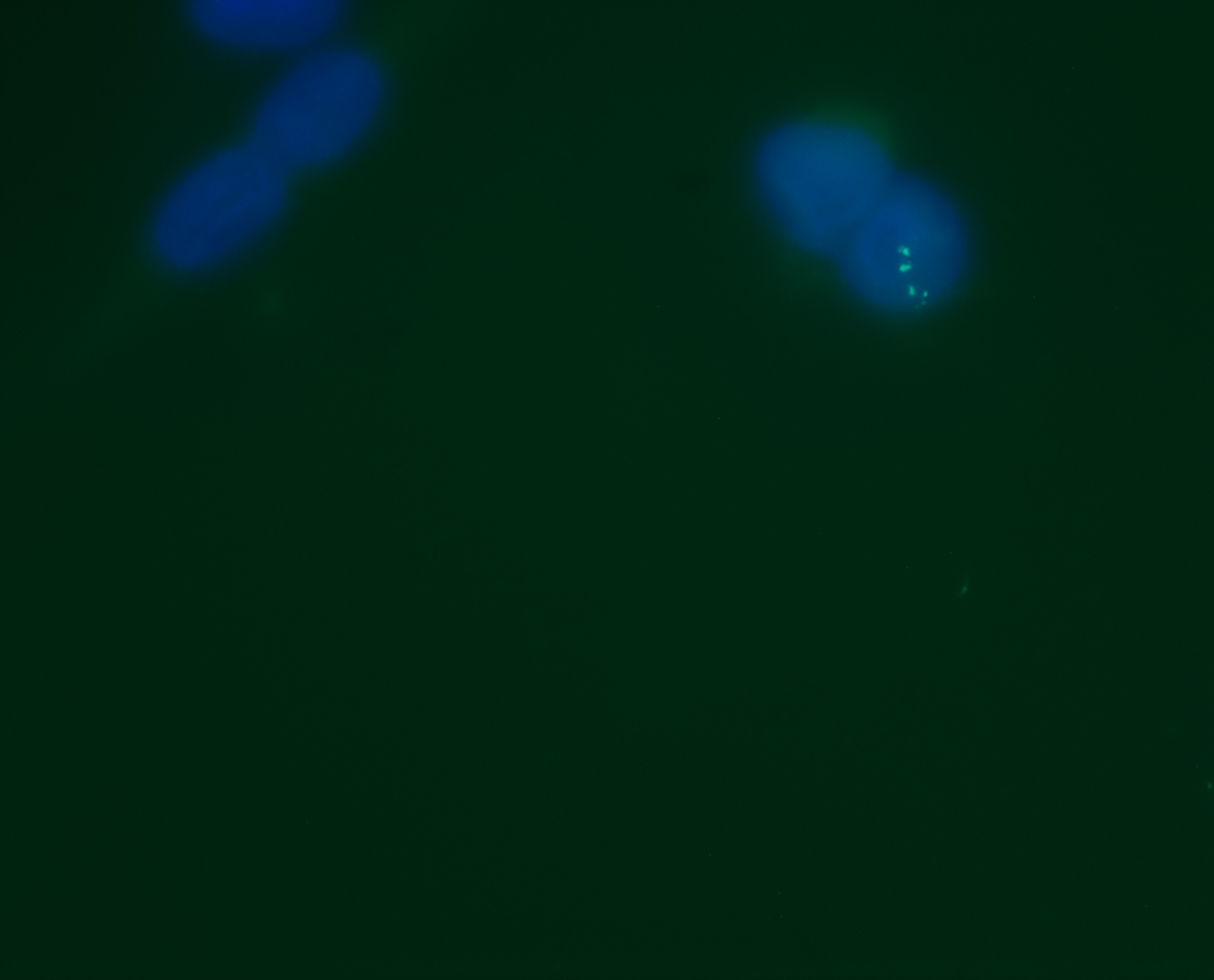

Supplement: Figure 3—source data 4. [file elife-85412-fig3-data4.zip › Figure 3 - source data 4 Figure 3 G/ctr sgRNA-1.jpg]

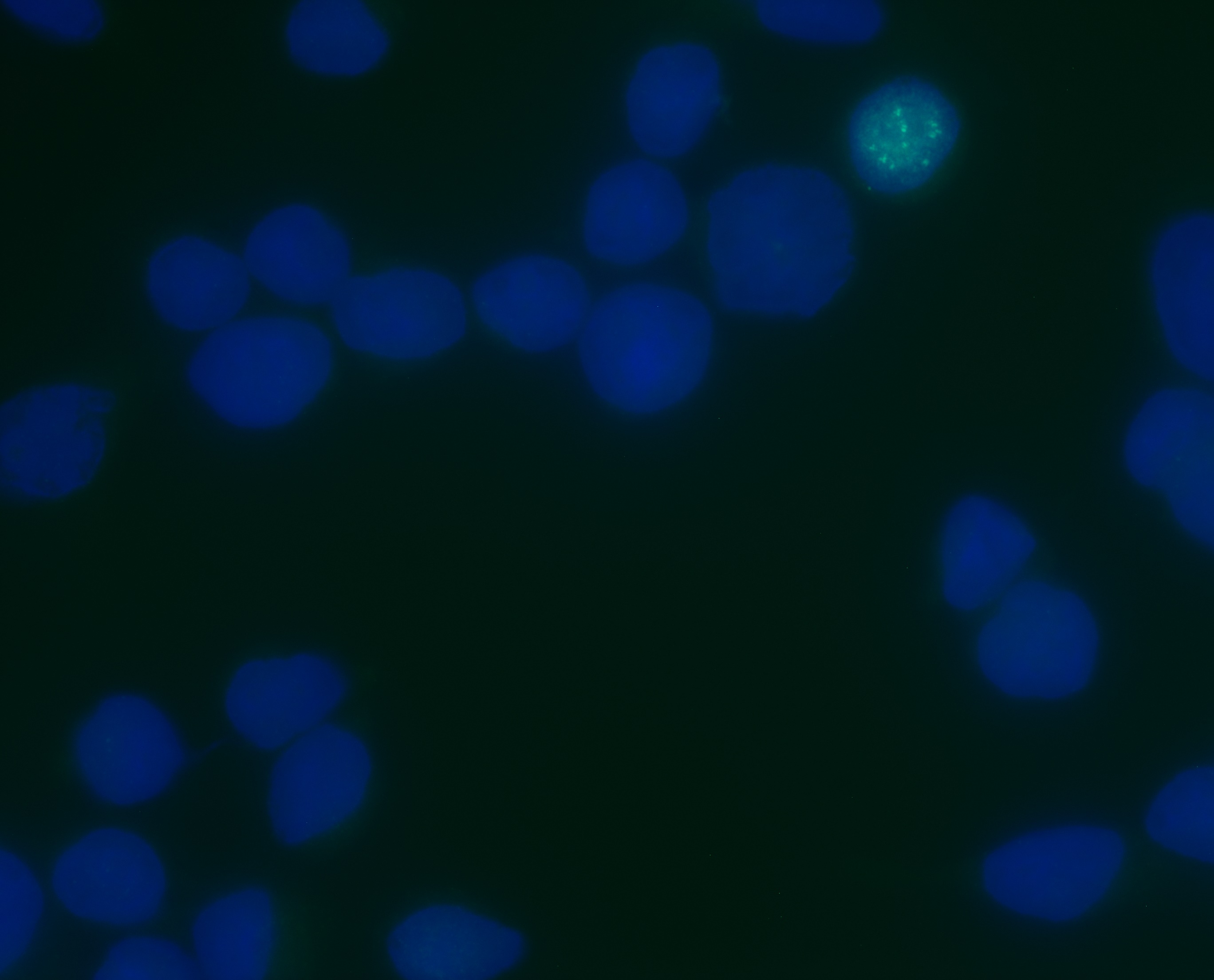

Supplement: Figure 3—source data 4. [file elife-85412-fig3-data4.zip › Figure 3 - source data 4 Figure 3 G/ctr- sgRNA-2.jpg]

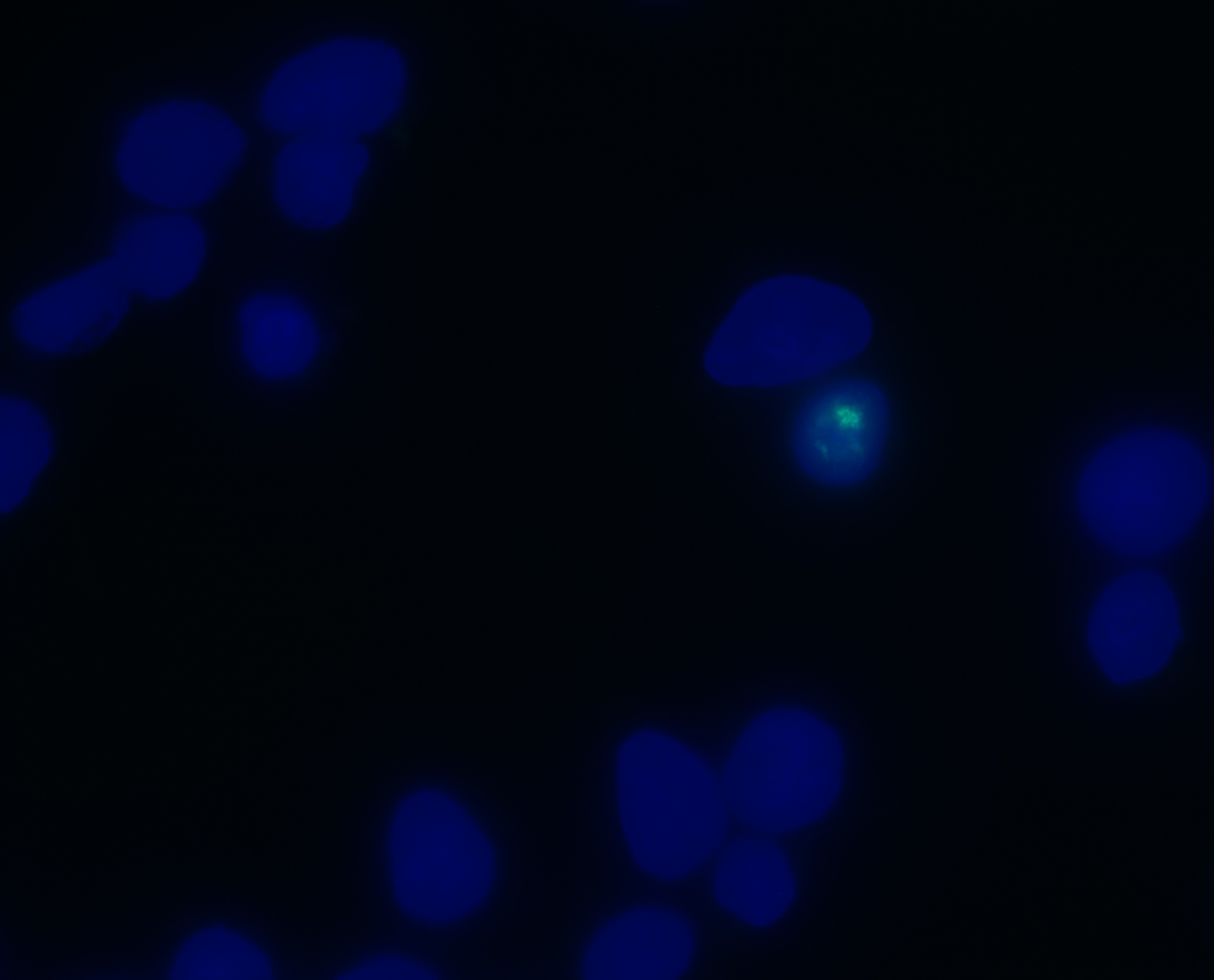

Supplement: Figure 3—source data 4. [file elife-85412-fig3-data4.zip › Figure 3 - source data 4 Figure 3 G/ctr sgRNA-3.jpg]

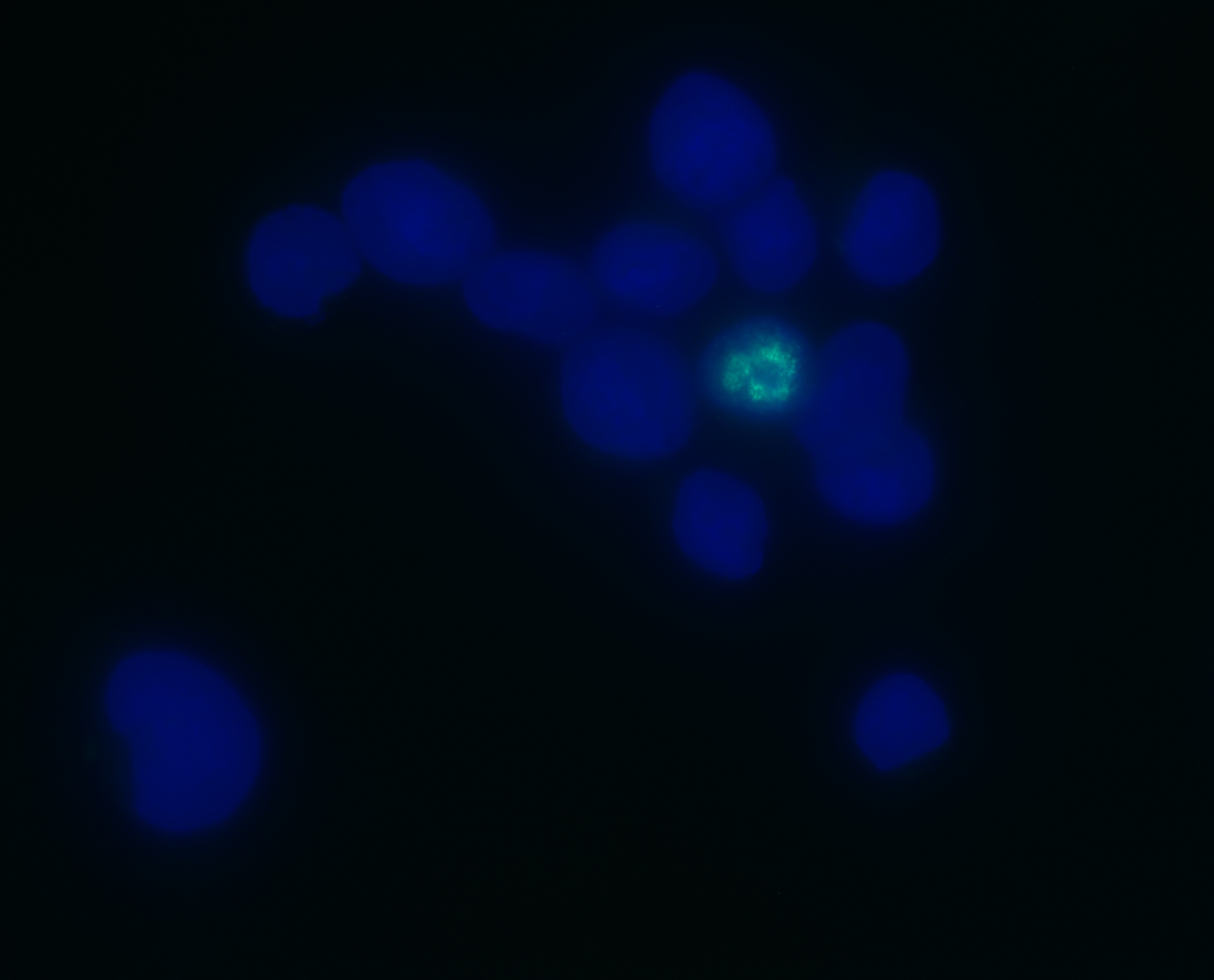

Supplement: Figure 3—source data 4. [file elife-85412-fig3-data4.zip › Figure 3 - source data 4 Figure 3 G/ctr sgRNA-4.jpg]

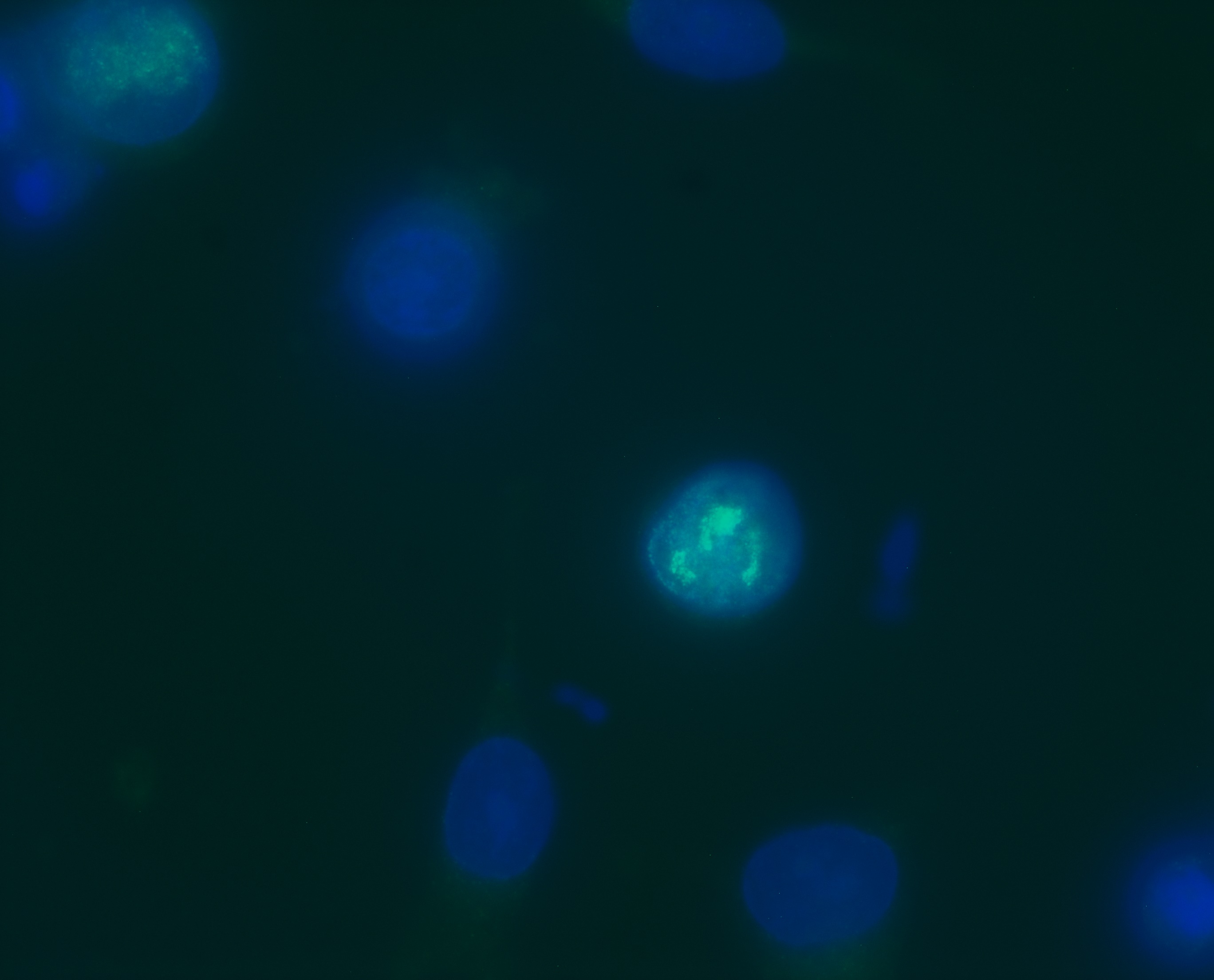

Supplement: Figure 3—source data 4. [file elife-85412-fig3-data4.zip › Figure 3 - source data 4 Figure 3 G/ctr sgRNA-5.jpg]

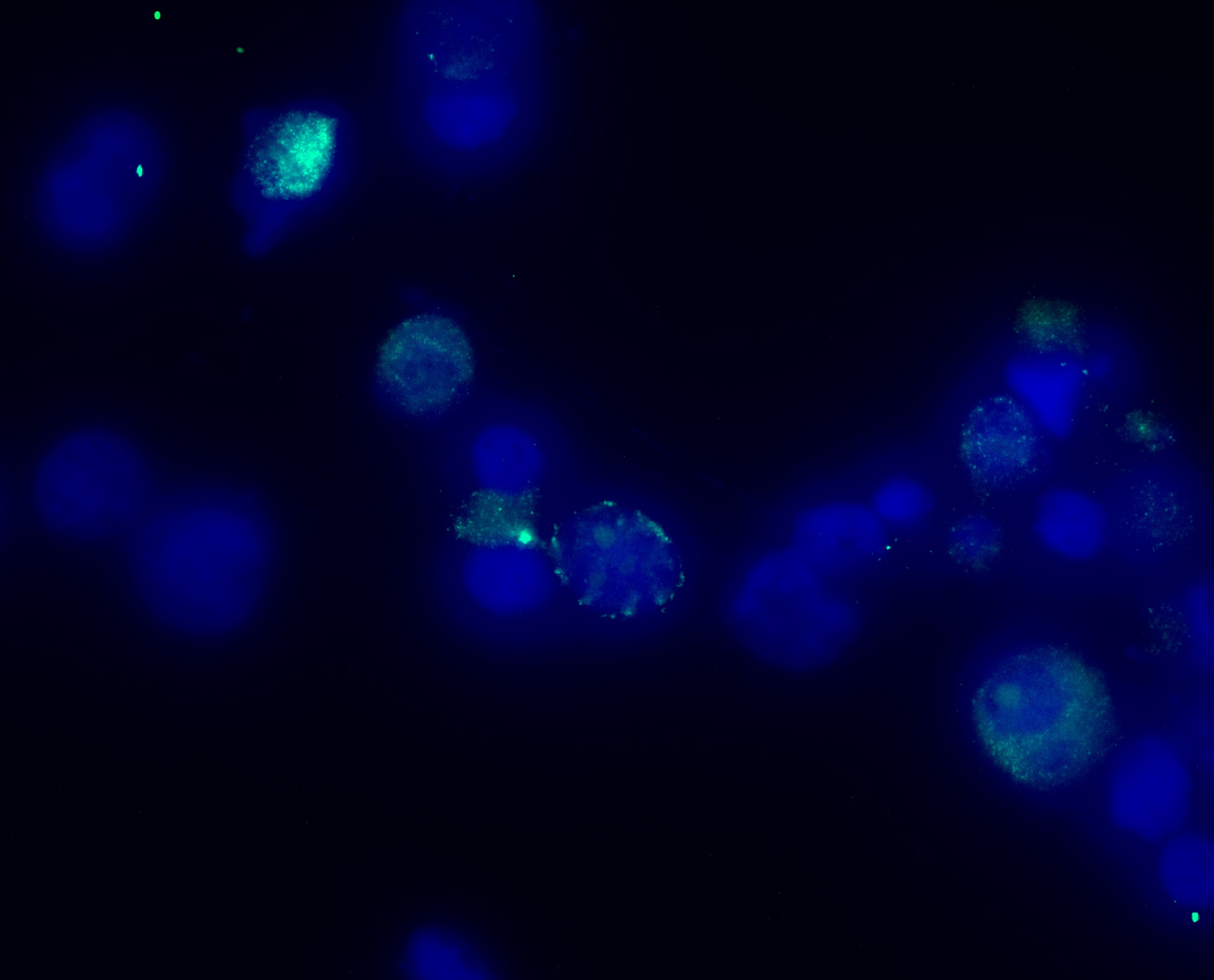

Supplement: Figure 3—source data 4. [file elife-85412-fig3-data4.zip › Figure 3 - source data 4 Figure 3 G/HSV-1 sgRAN-2_c1-2.jpg]

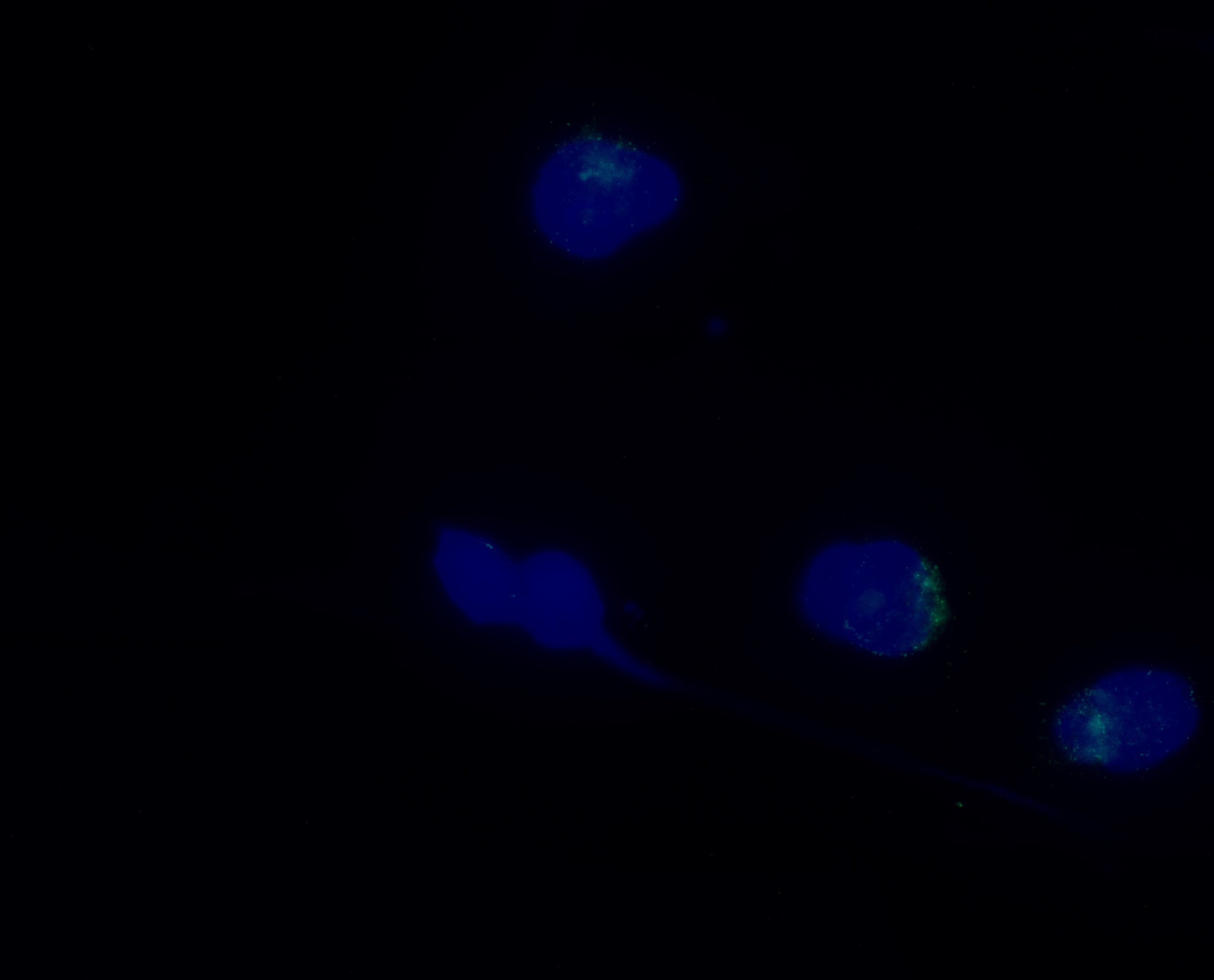

Supplement: Figure 3—source data 4. [file elife-85412-fig3-data4.zip › Figure 3 - source data 4 Figure 3 G/HSV-1 sgRNA-1.jpg]

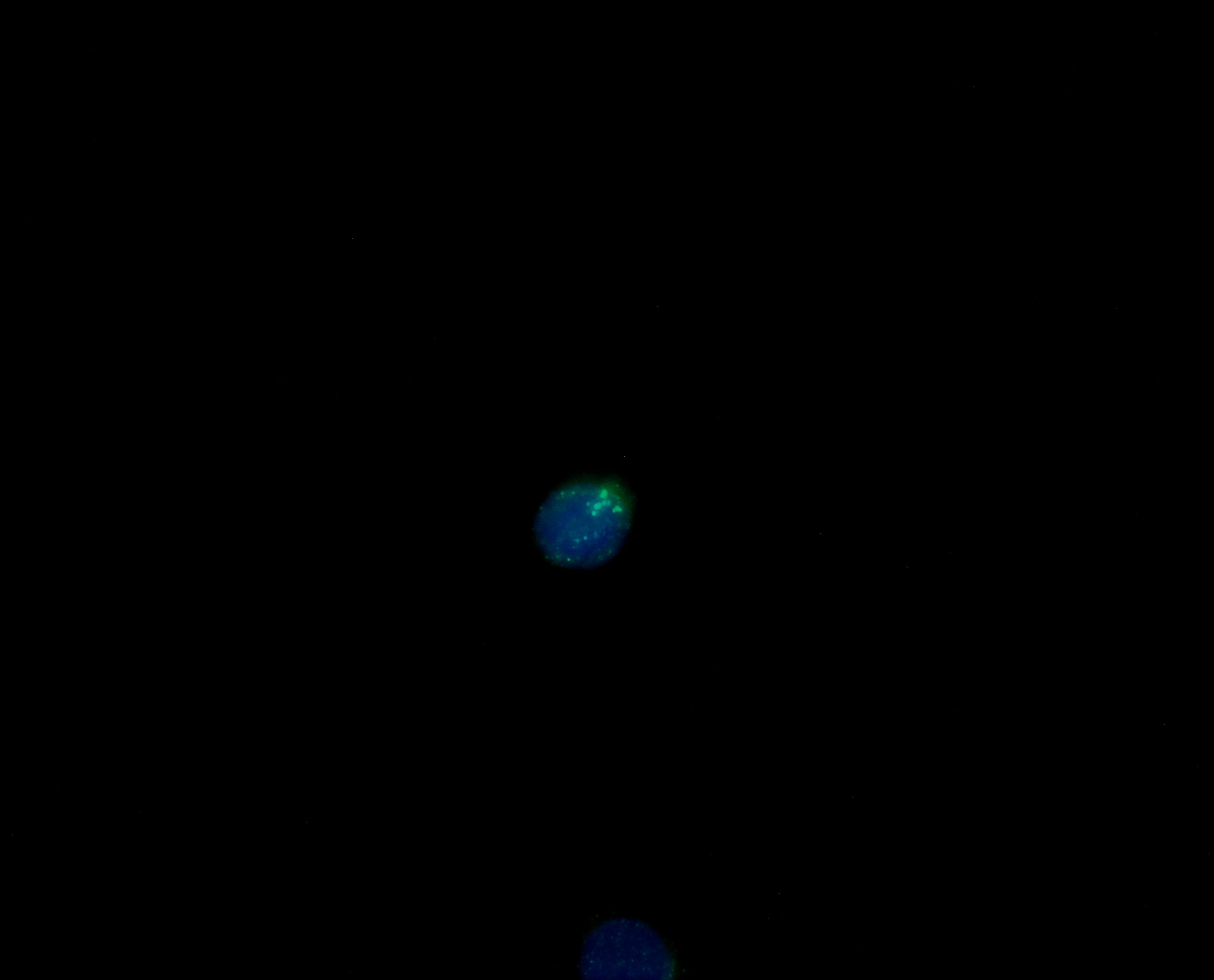

Supplement: Figure 3—source data 4. [file elife-85412-fig3-data4.zip › Figure 3 - source data 4 Figure 3 G/HSV-1 sgRNA-3_c1-2.jpg]

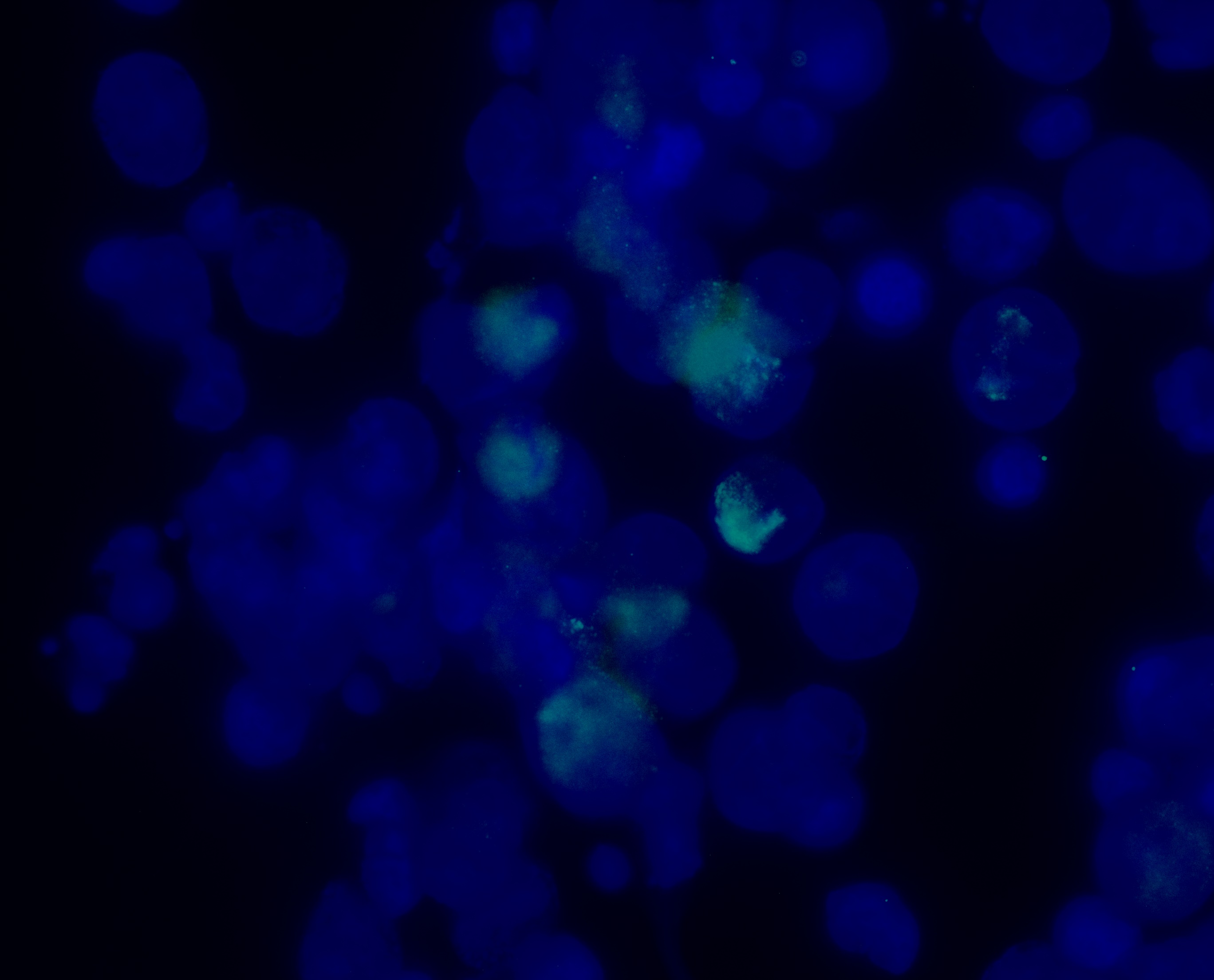

Supplement: Figure 3—source data 4. [file elife-85412-fig3-data4.zip › Figure 3 - source data 4 Figure 3 G/HSV-1 sgRNA-4.jpg]

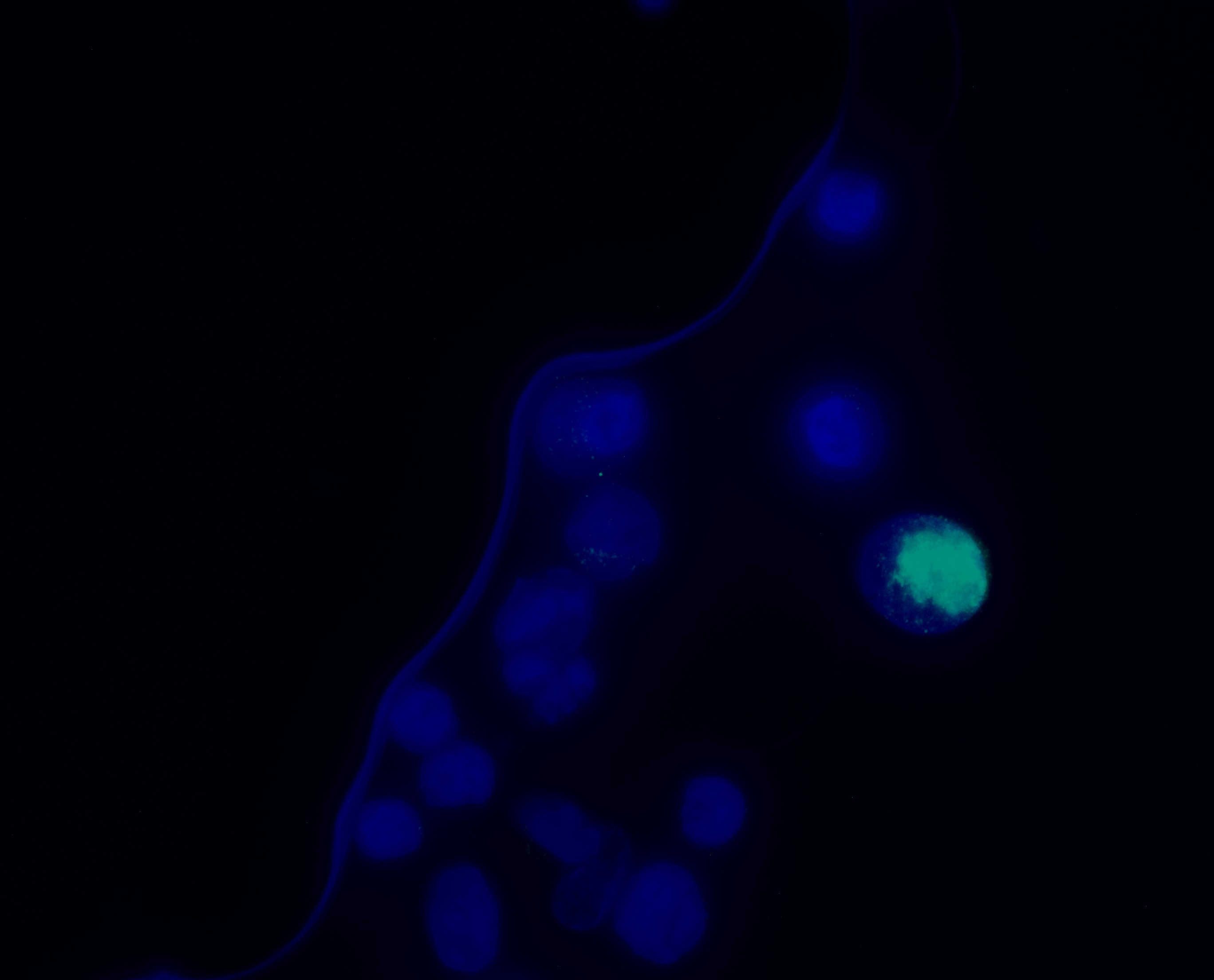

Supplement: Figure 3—source data 4. [file elife-85412-fig3-data4.zip › Figure 3 - source data 4 Figure 3 G/HSV-1 sgRNA-5.jpg]

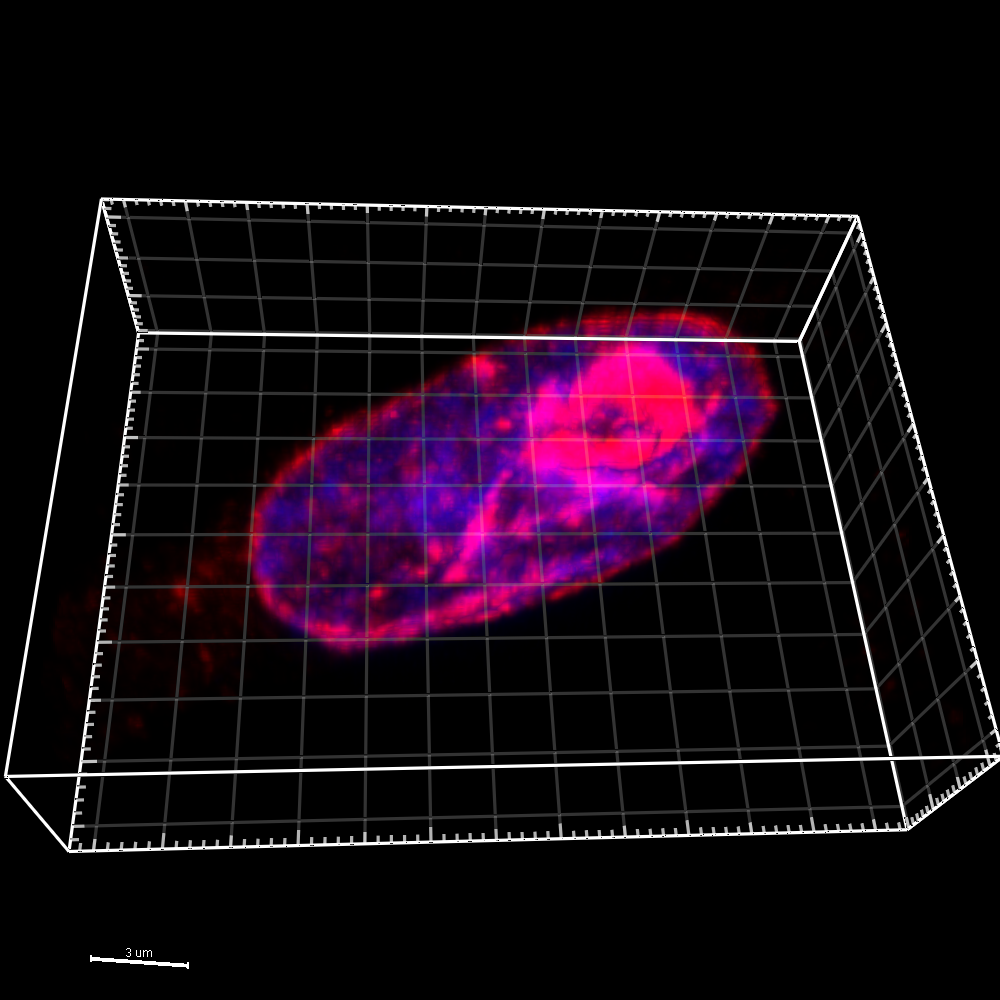

Supplement: Figure 3—figure supplement 1—source data 1. — This zip archive contains source data for original virus titers in panel D, and processed images (TIF files) in panels A, B, and C. Original confocal images are deposited on DRYAD (https://doi.org/10.5061/dryad.vmcvdncxd). [file elife-85412-fig3-figsupp1-data1.zip › Figure 3-figure supplement 1-source data 1 Figure 3-figure supplement 1 A/ICP8+EMERIN-0H-MOi =5.tif]

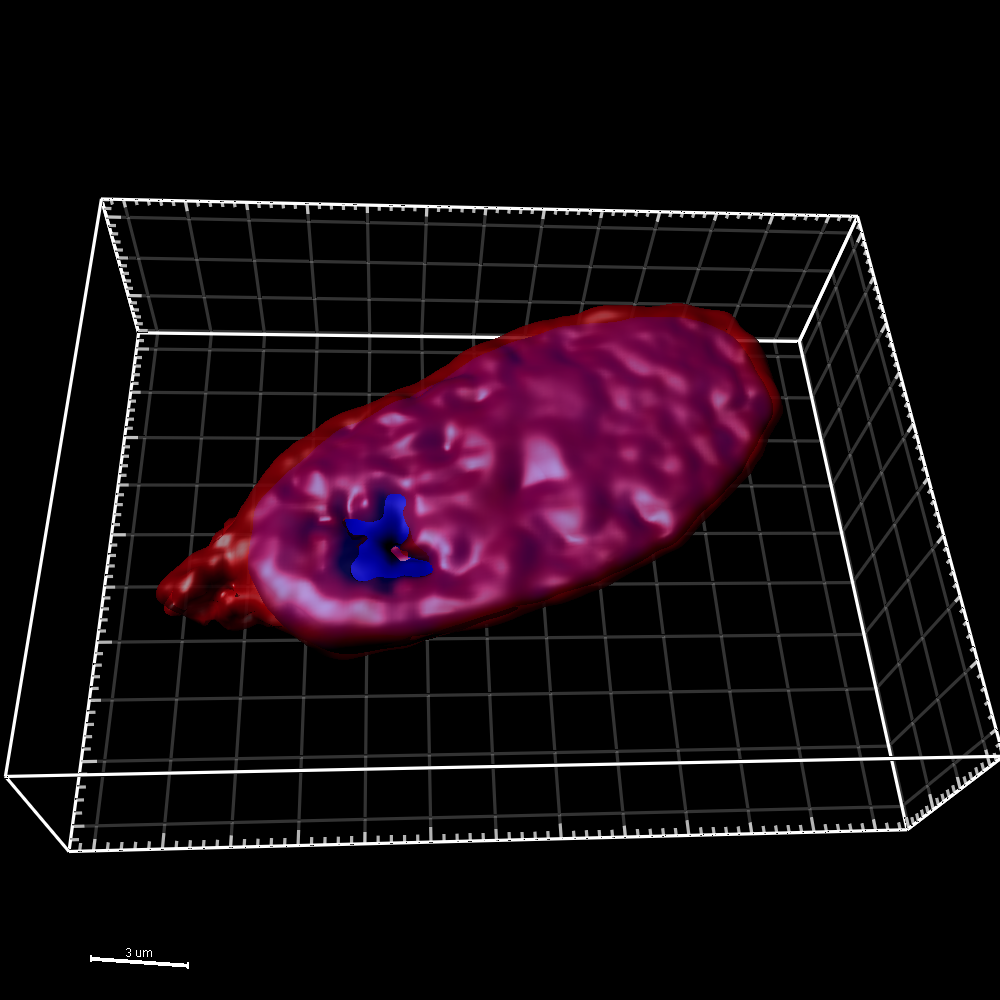

Supplement: Figure 3—figure supplement 1—source data 1. — This zip archive contains source data for original virus titers in panel D, and processed images (TIF files) in panels A, B, and C. Original confocal images are deposited on DRYAD (https://doi.org/10.5061/dryad.vmcvdncxd). [file elife-85412-fig3-figsupp1-data1.zip › Figure 3-figure supplement 1-source data 1 Figure 3-figure supplement 1 A/ICP8+EMERIN-0H-MOi =5-3D.tif]

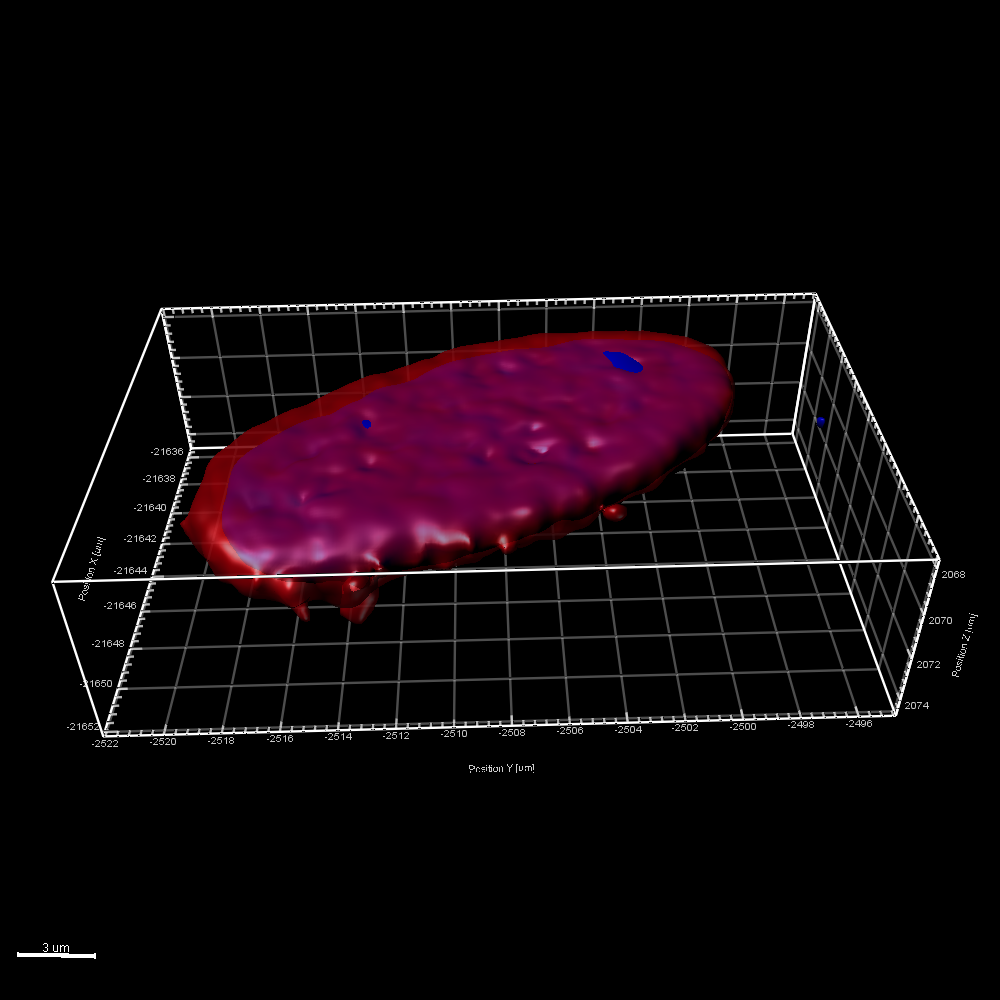

Supplement: Figure 3—figure supplement 1—source data 1. — This zip archive contains source data for original virus titers in panel D, and processed images (TIF files) in panels A, B, and C. Original confocal images are deposited on DRYAD (https://doi.org/10.5061/dryad.vmcvdncxd). [file elife-85412-fig3-figsupp1-data1.zip › Figure 3-figure supplement 1-source data 1 Figure 3-figure supplement 1 A/ICP8+EMERIN-1H-MOI=5 -3D.tif]

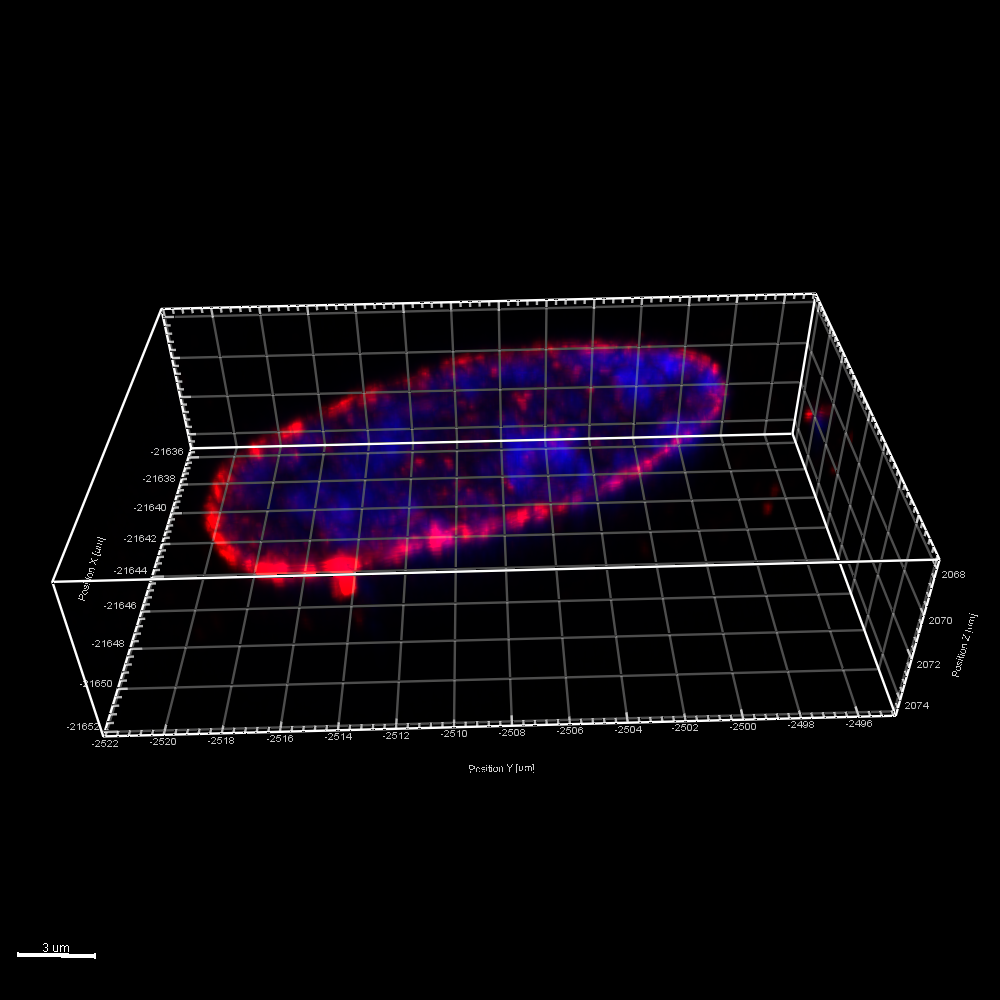

Supplement: Figure 3—figure supplement 1—source data 1. — This zip archive contains source data for original virus titers in panel D, and processed images (TIF files) in panels A, B, and C. Original confocal images are deposited on DRYAD (https://doi.org/10.5061/dryad.vmcvdncxd). [file elife-85412-fig3-figsupp1-data1.zip › Figure 3-figure supplement 1-source data 1 Figure 3-figure supplement 1 A/ICP8+EMERIN-1H-MOI=5.tif]

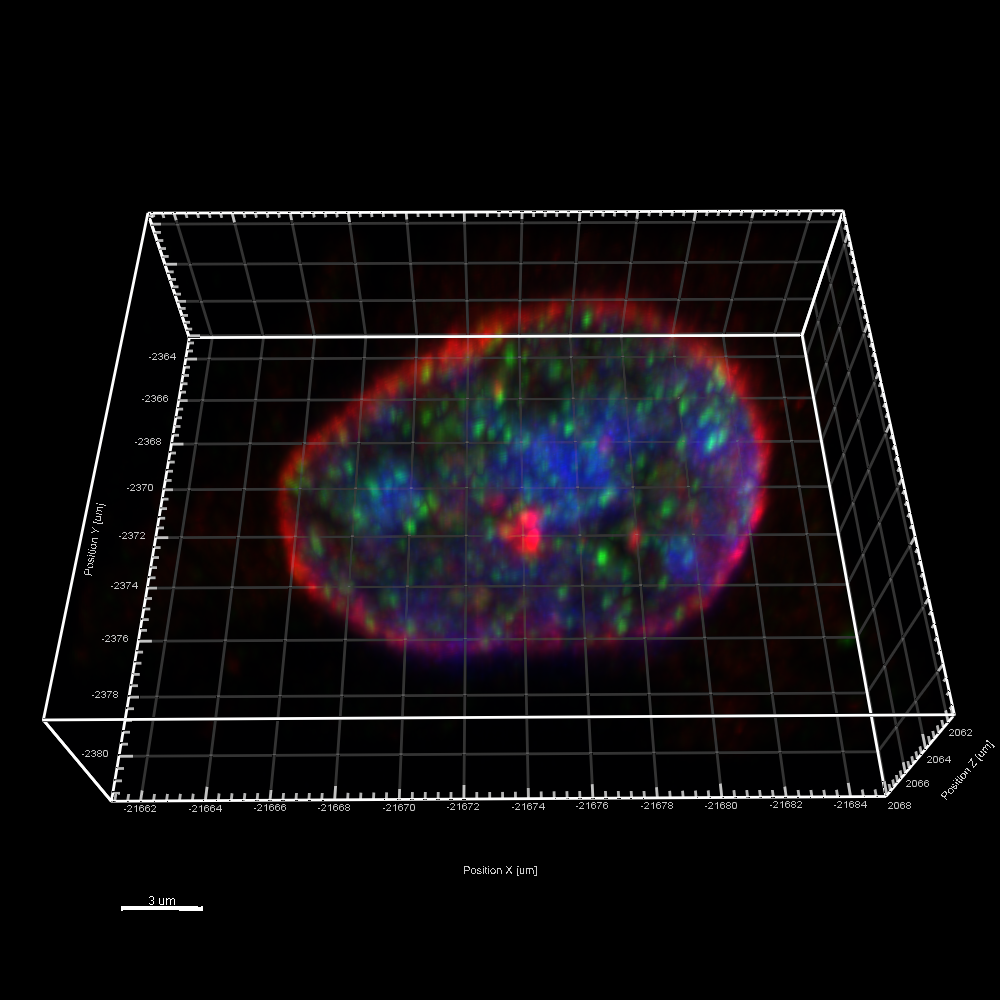

Supplement: Figure 3—figure supplement 1—source data 1. — This zip archive contains source data for original virus titers in panel D, and processed images (TIF files) in panels A, B, and C. Original confocal images are deposited on DRYAD (https://doi.org/10.5061/dryad.vmcvdncxd). [file elife-85412-fig3-figsupp1-data1.zip › Figure 3-figure supplement 1-source data 1 Figure 3-figure supplement 1 A/ICP8+EMERIN-3H-MOI=5.tif]

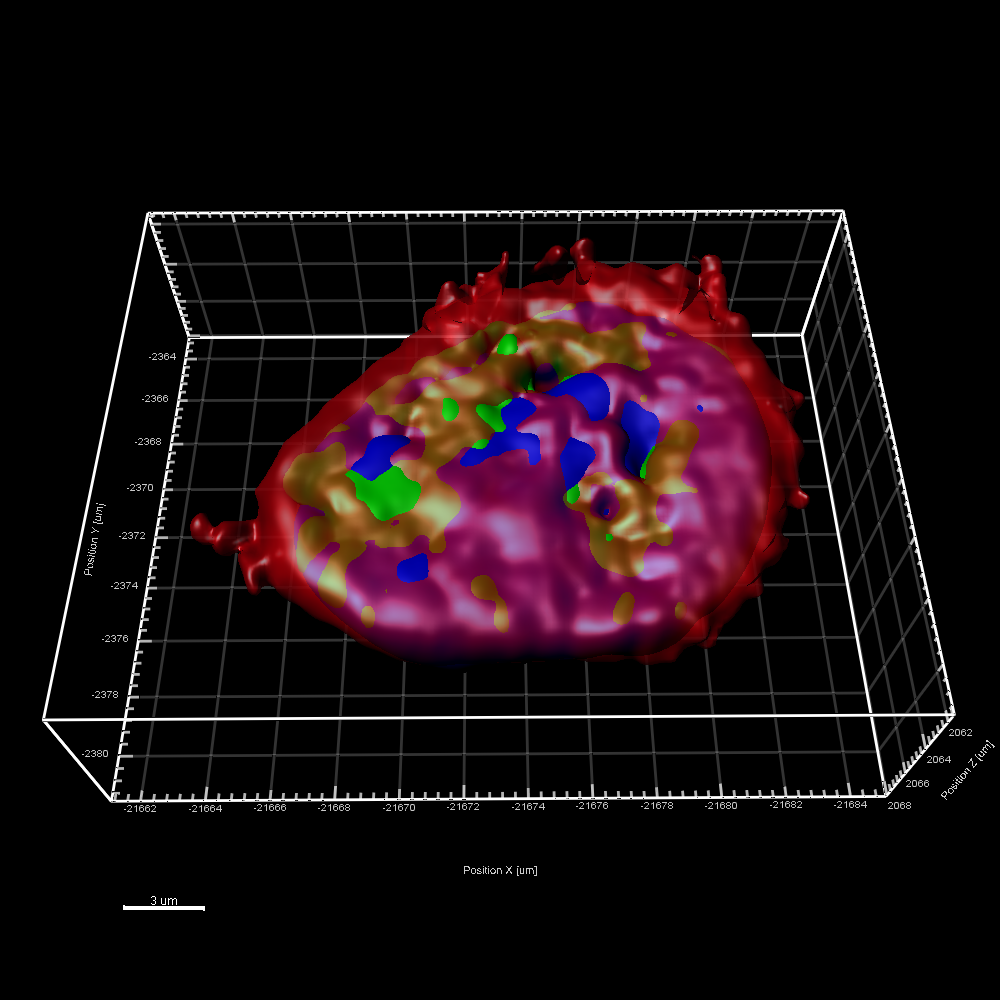

Supplement: Figure 3—figure supplement 1—source data 1. — This zip archive contains source data for original virus titers in panel D, and processed images (TIF files) in panels A, B, and C. Original confocal images are deposited on DRYAD (https://doi.org/10.5061/dryad.vmcvdncxd). [file elife-85412-fig3-figsupp1-data1.zip › Figure 3-figure supplement 1-source data 1 Figure 3-figure supplement 1 A/ICP8+EMERIN-3H-MOI=5-3D.tif]

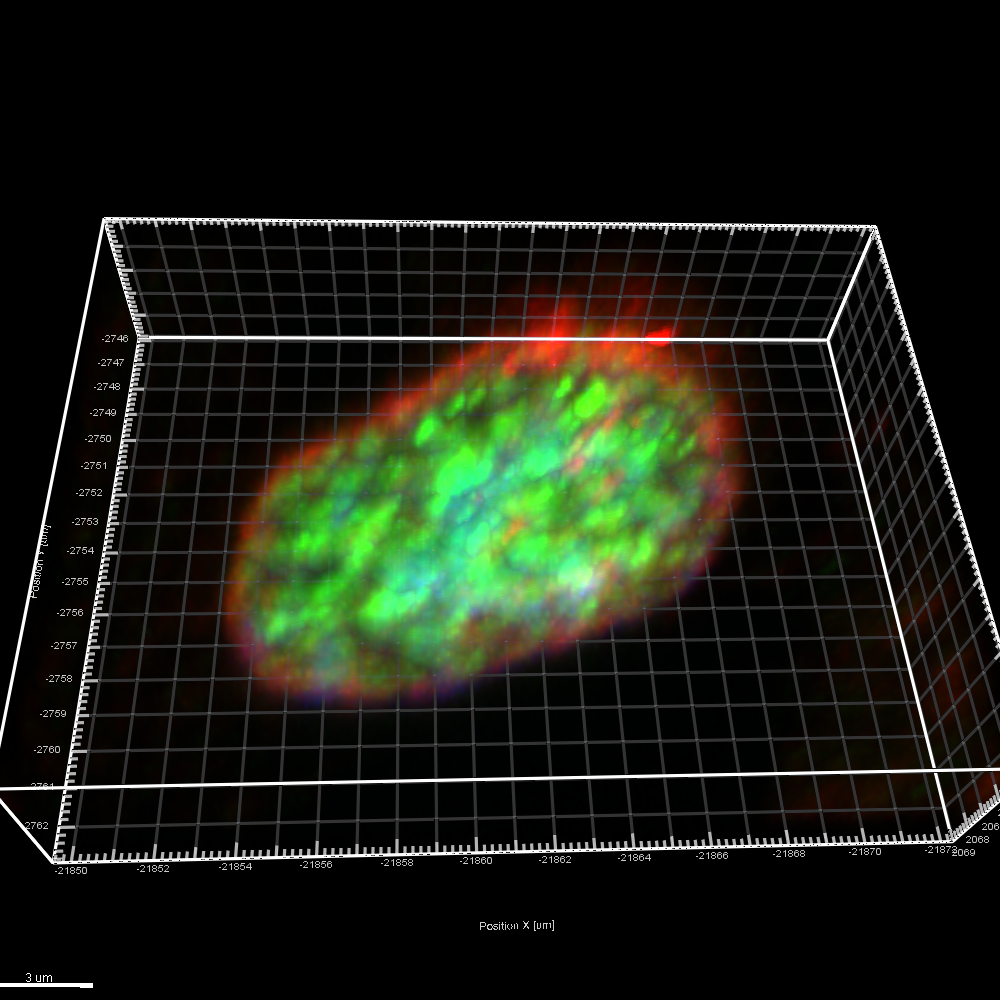

Supplement: Figure 3—figure supplement 1—source data 1. — This zip archive contains source data for original virus titers in panel D, and processed images (TIF files) in panels A, B, and C. Original confocal images are deposited on DRYAD (https://doi.org/10.5061/dryad.vmcvdncxd). [file elife-85412-fig3-figsupp1-data1.zip › Figure 3-figure supplement 1-source data 1 Figure 3-figure supplement 1 A/ICP8+EMERIN-5H-MOI=5.tif]

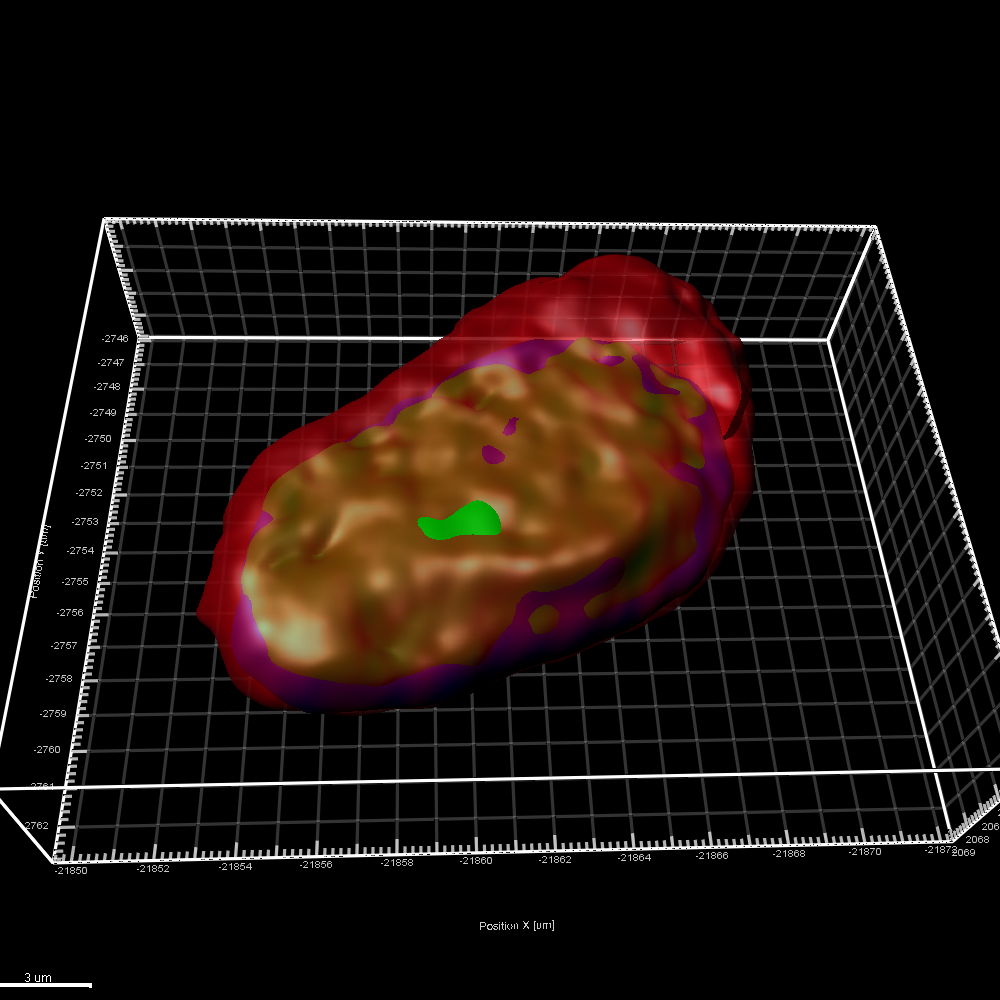

Supplement: Figure 3—figure supplement 1—source data 1. — This zip archive contains source data for original virus titers in panel D, and processed images (TIF files) in panels A, B, and C. Original confocal images are deposited on DRYAD (https://doi.org/10.5061/dryad.vmcvdncxd). [file elife-85412-fig3-figsupp1-data1.zip › Figure 3-figure supplement 1-source data 1 Figure 3-figure supplement 1 A/ICP8+EMERIN-5H-MOI=5-3D.tif]

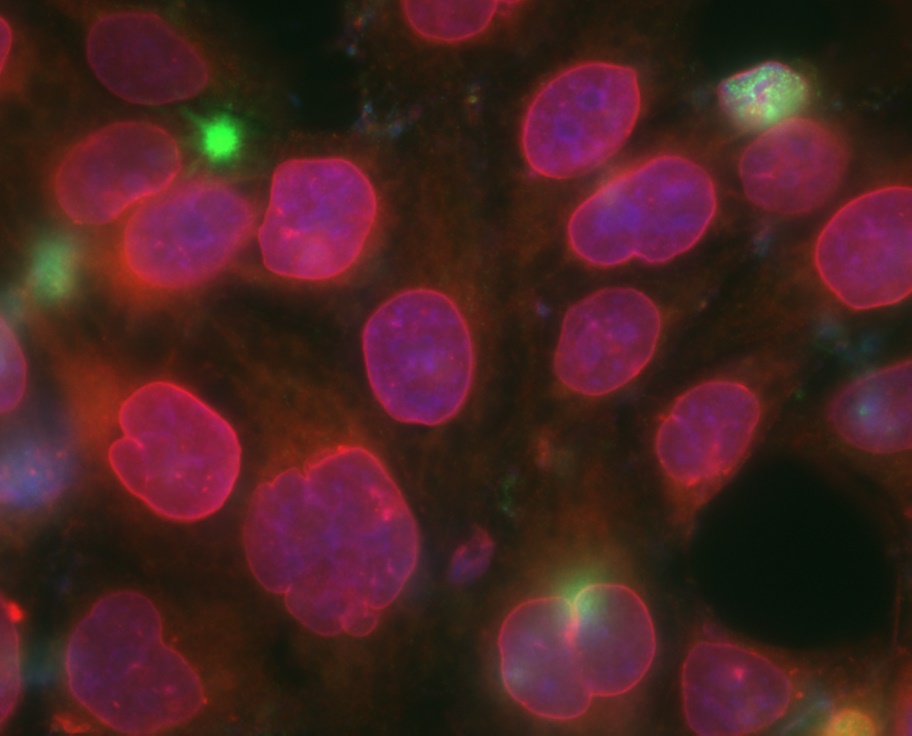

Supplement: Figure 3—figure supplement 1—source data 2. [file elife-85412-fig3-figsupp1-data2.zip › Figure 3-figure supplement 1-source data 2 Figure 3-figure supplement 1 B/sup fig3-b-0hpi-Snap-793_c1-3.jpg]

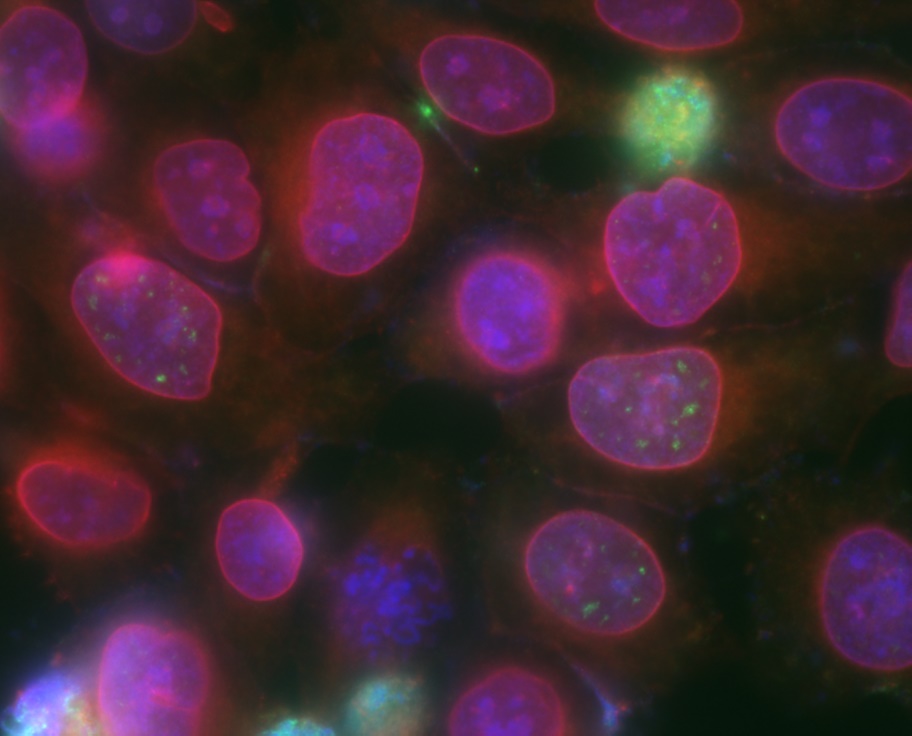

Supplement: Figure 3—figure supplement 1—source data 2. [file elife-85412-fig3-figsupp1-data2.zip › Figure 3-figure supplement 1-source data 2 Figure 3-figure supplement 1 B/sup fig3-b-1hpi-Snap-790_c1-3.jpg]

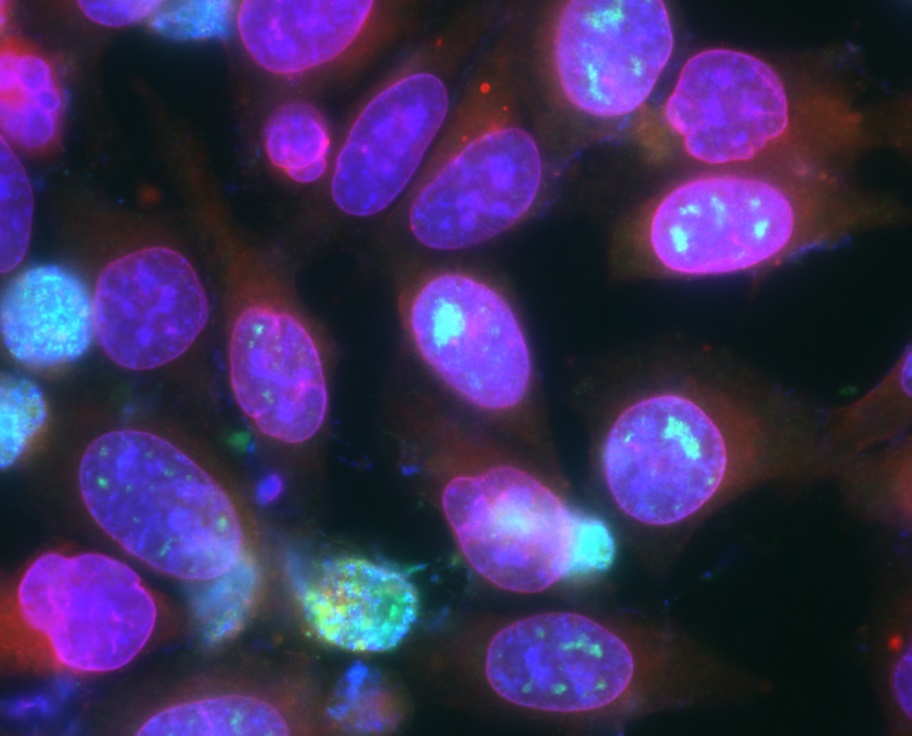

Supplement: Figure 3—figure supplement 1—source data 2. [file elife-85412-fig3-figsupp1-data2.zip › Figure 3-figure supplement 1-source data 2 Figure 3-figure supplement 1 B/sup fig3-b-2hpi-Snap-789_c1-3.jpg]

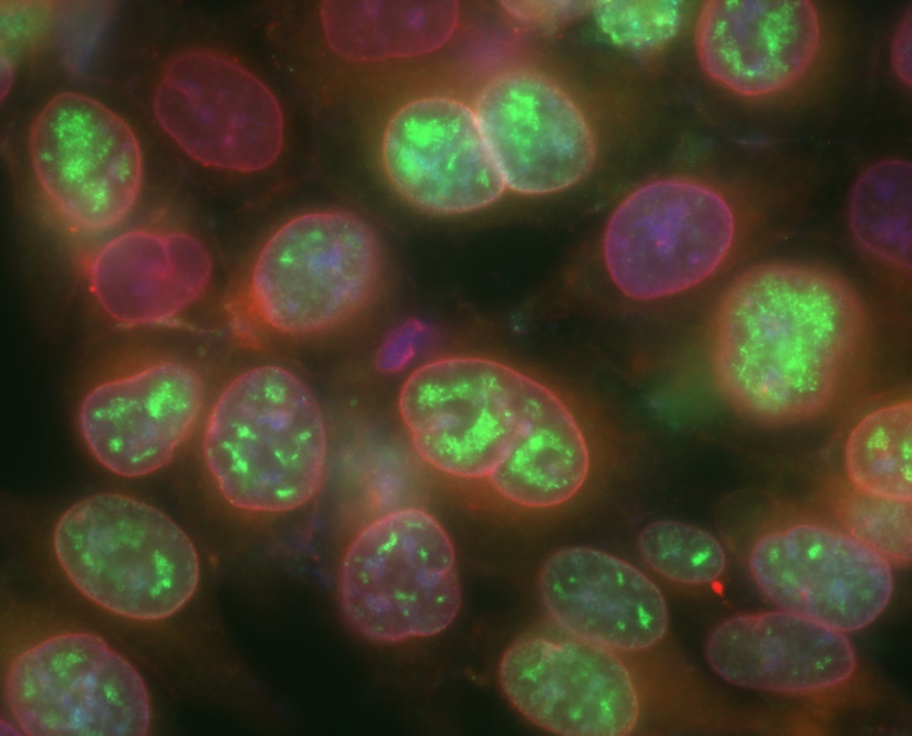

Supplement: Figure 3—figure supplement 1—source data 2. [file elife-85412-fig3-figsupp1-data2.zip › Figure 3-figure supplement 1-source data 2 Figure 3-figure supplement 1 B/sup fig3-b-3hpi-Snap-785_c1-3.jpg]

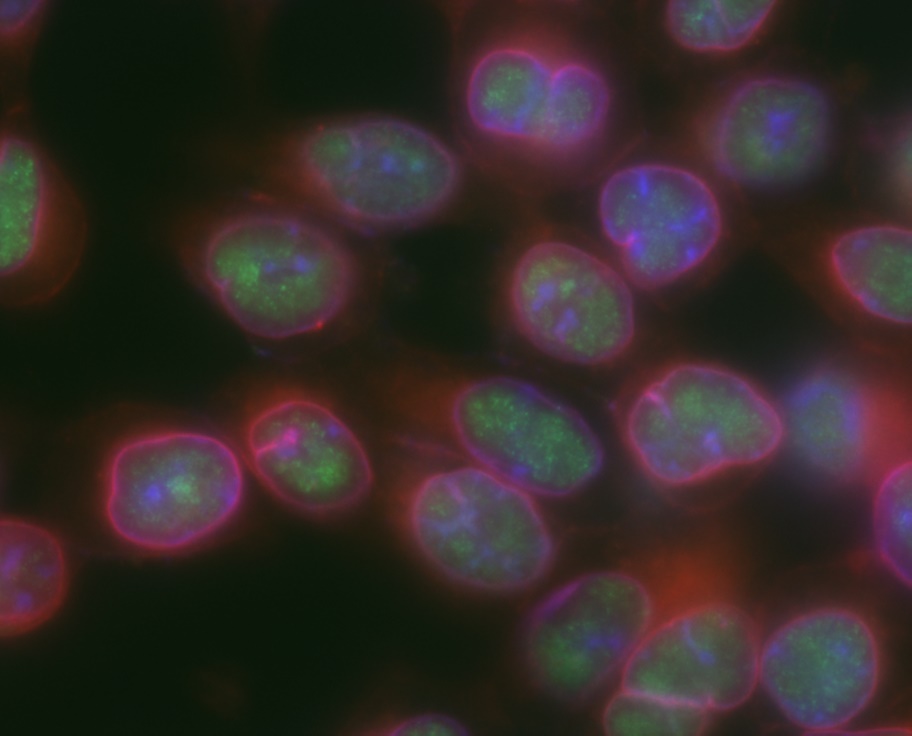

Supplement: Figure 3—figure supplement 1—source data 2. [file elife-85412-fig3-figsupp1-data2.zip › Figure 3-figure supplement 1-source data 2 Figure 3-figure supplement 1 B/sup fig3-b-6hpi-Snap-780_c1-3.jpg]

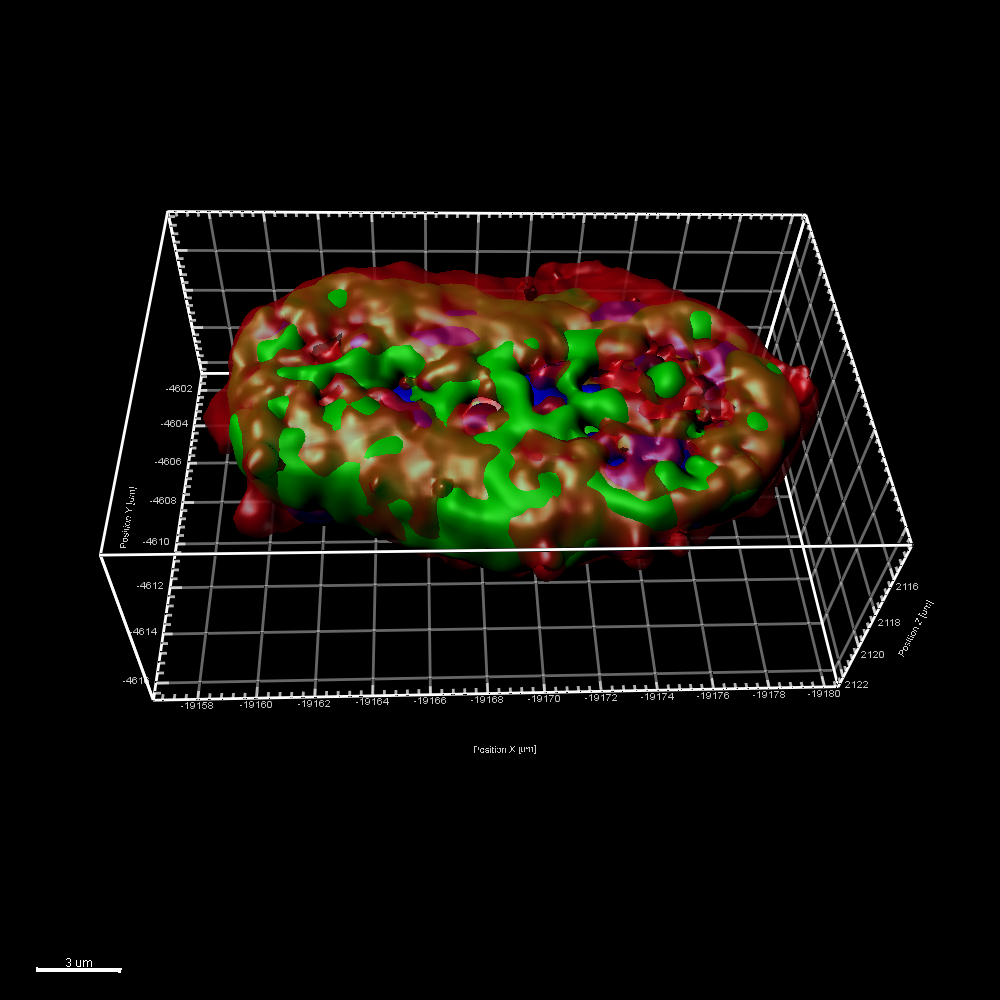

Supplement: Figure 3—figure supplement 1—source data 3. [file elife-85412-fig3-figsupp1-data3.zip › Figure 3-figure supplement 1-source data 3 Figure 3-figure supplement 1 C/FLAG+NUP-MOi =5 16H -3D.tif]

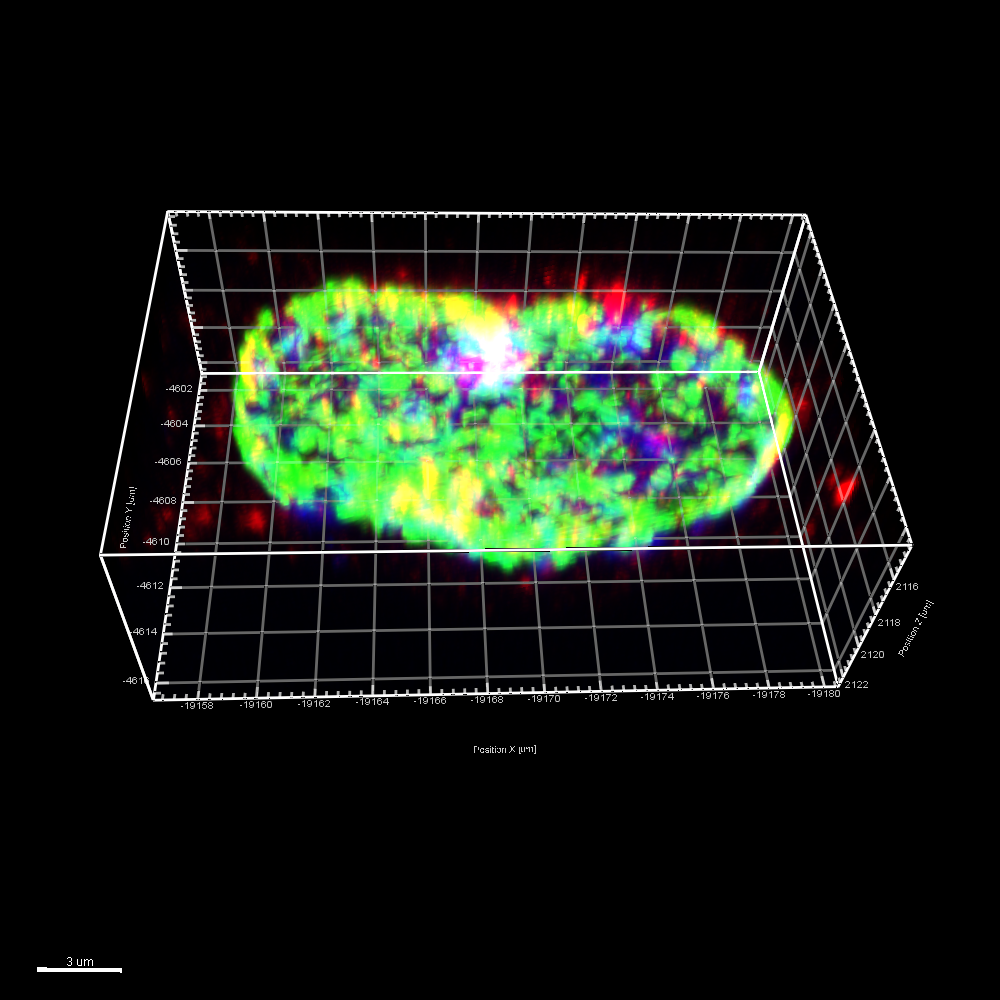

Supplement: Figure 3—figure supplement 1—source data 3. [file elife-85412-fig3-figsupp1-data3.zip › Figure 3-figure supplement 1-source data 3 Figure 3-figure supplement 1 C/FLAG+NUP-MOi =5 16H.tif]

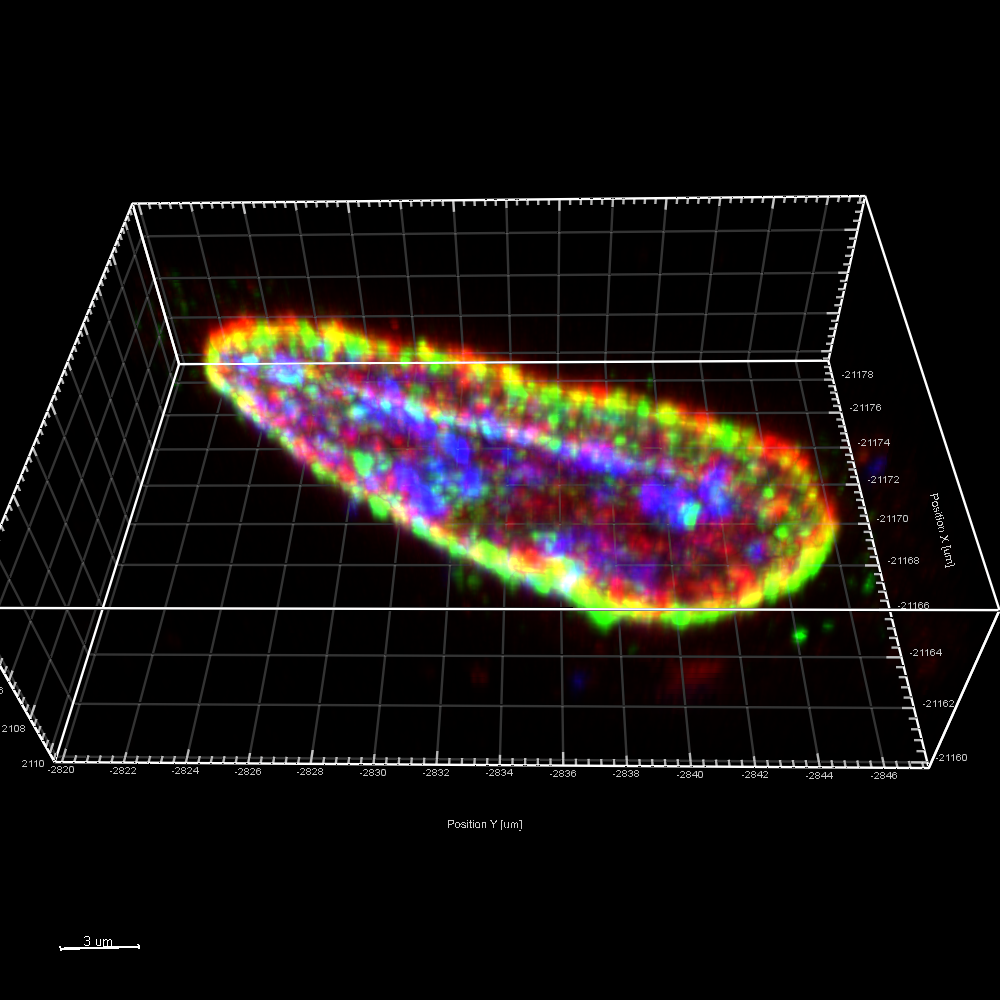

Supplement: Figure 3—figure supplement 1—source data 3. [file elife-85412-fig3-figsupp1-data3.zip › Figure 3-figure supplement 1-source data 3 Figure 3-figure supplement 1 C/FLAG+NUP-MOi =5 1H.tif]

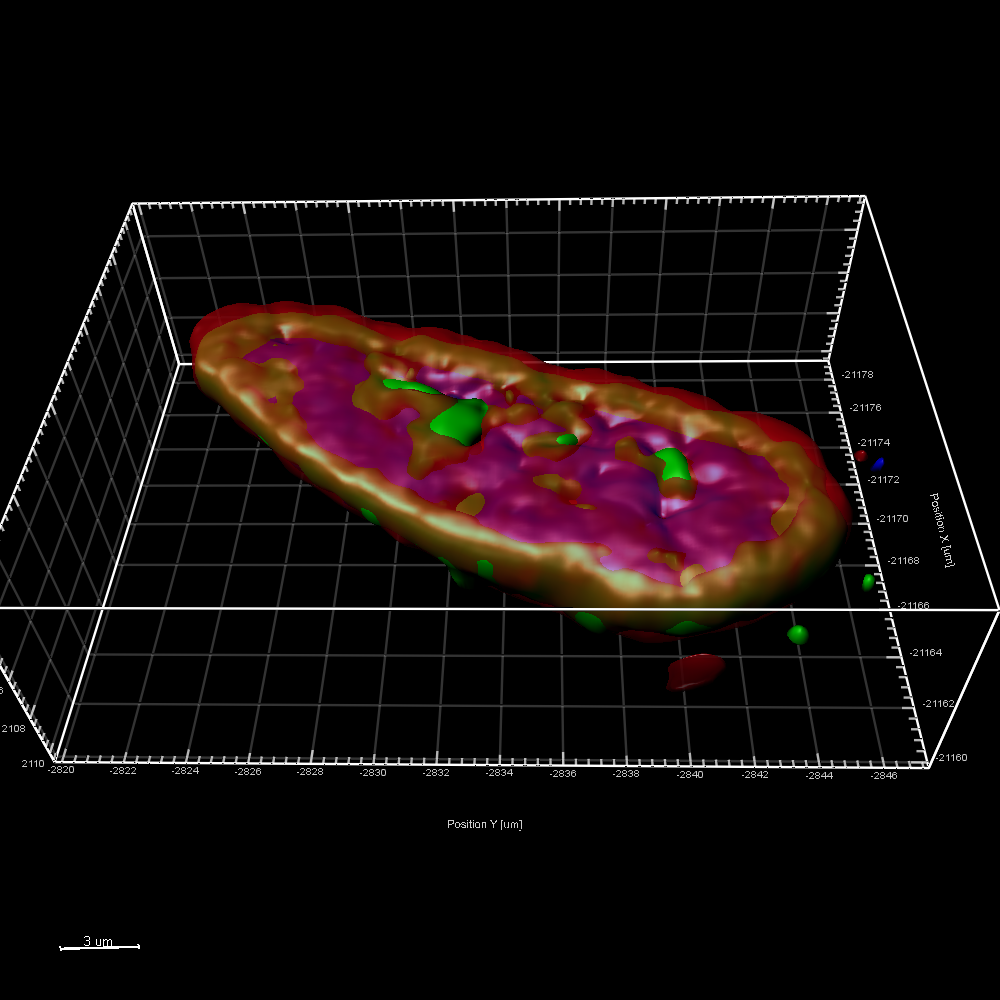

Supplement: Figure 3—figure supplement 1—source data 3. [file elife-85412-fig3-figsupp1-data3.zip › Figure 3-figure supplement 1-source data 3 Figure 3-figure supplement 1 C/FLAG+NUP-MOi =5 1H-3D.tif]

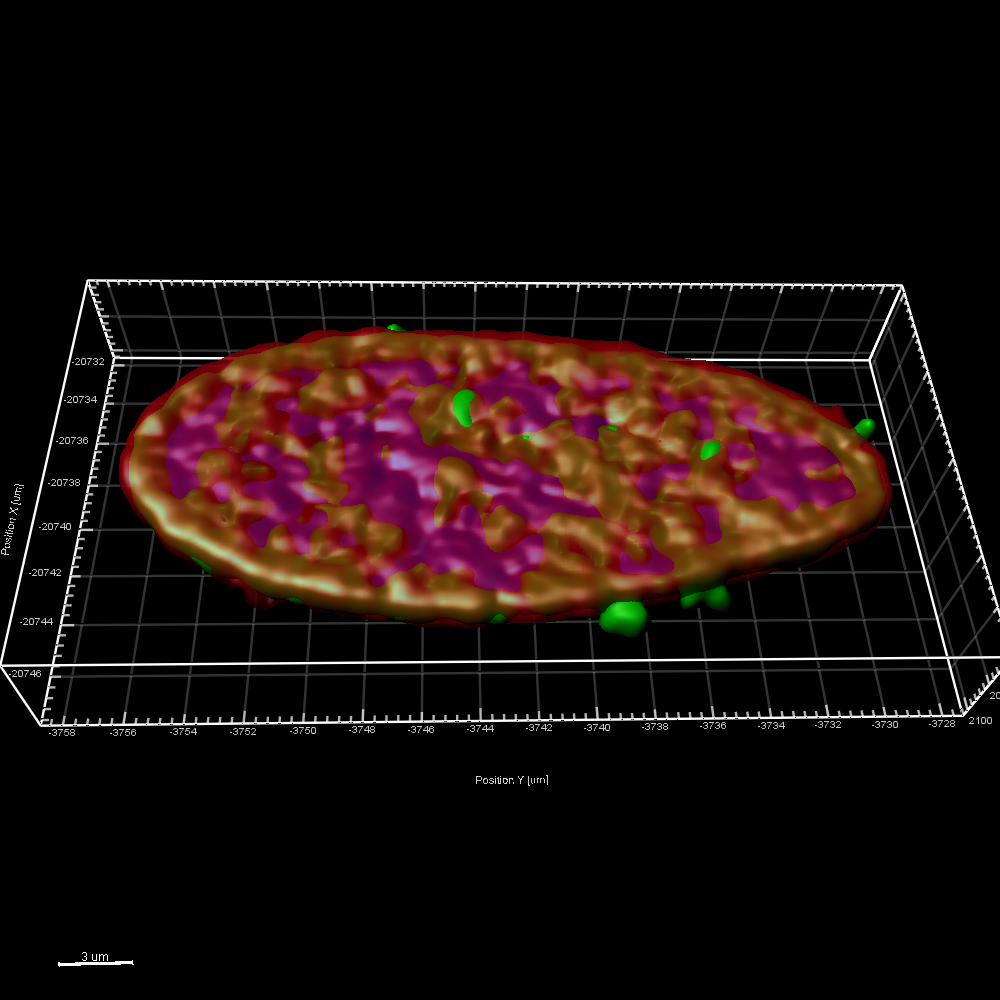

Supplement: Figure 3—figure supplement 1—source data 3. [file elife-85412-fig3-figsupp1-data3.zip › Figure 3-figure supplement 1-source data 3 Figure 3-figure supplement 1 C/FLAG+NUP-MOi =5 5H -3D.tif]

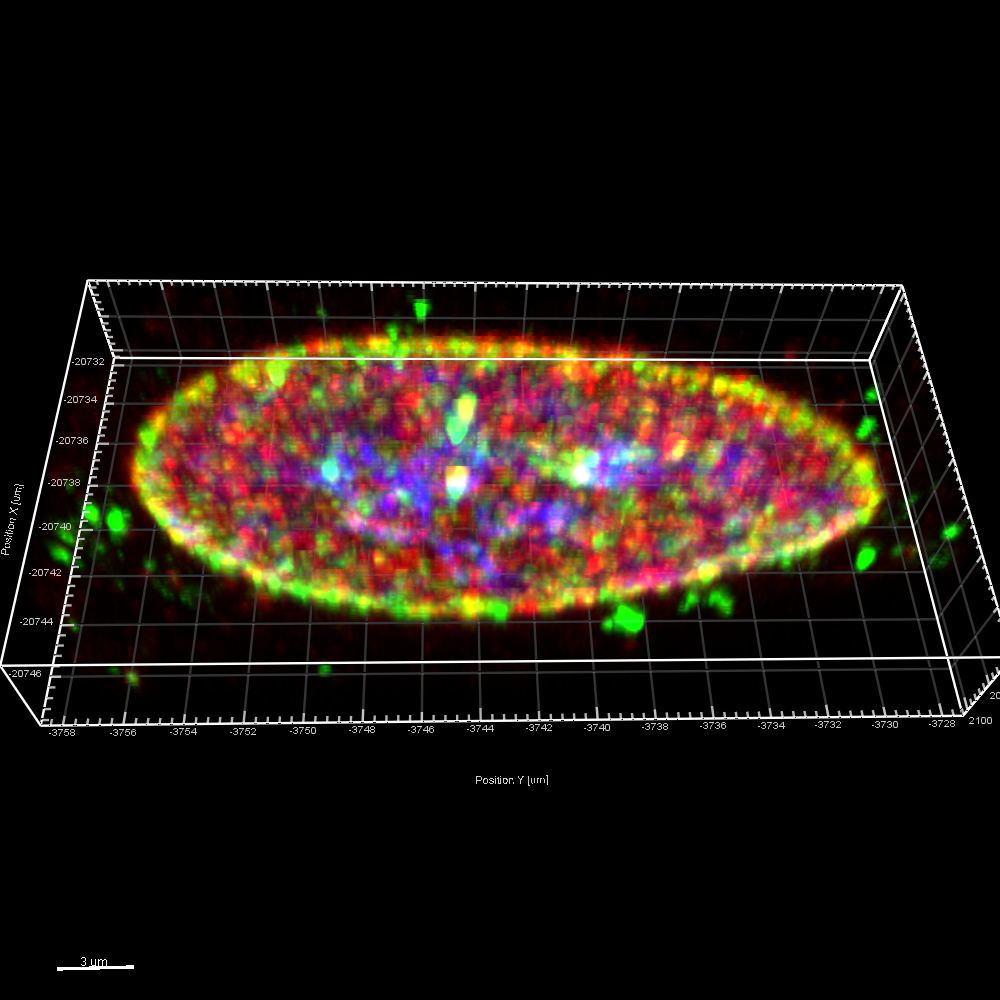

Supplement: Figure 3—figure supplement 1—source data 3. [file elife-85412-fig3-figsupp1-data3.zip › Figure 3-figure supplement 1-source data 3 Figure 3-figure supplement 1 C/FLAG+NUP-MOi =5 5H.tif]

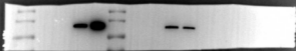

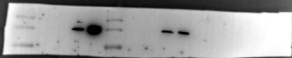

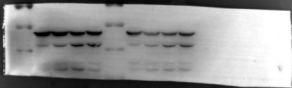

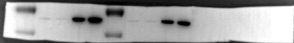

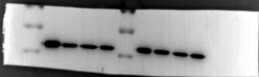

Supplement: Figure 4—source data 2. [file elife-85412-fig4-data2.pdf]

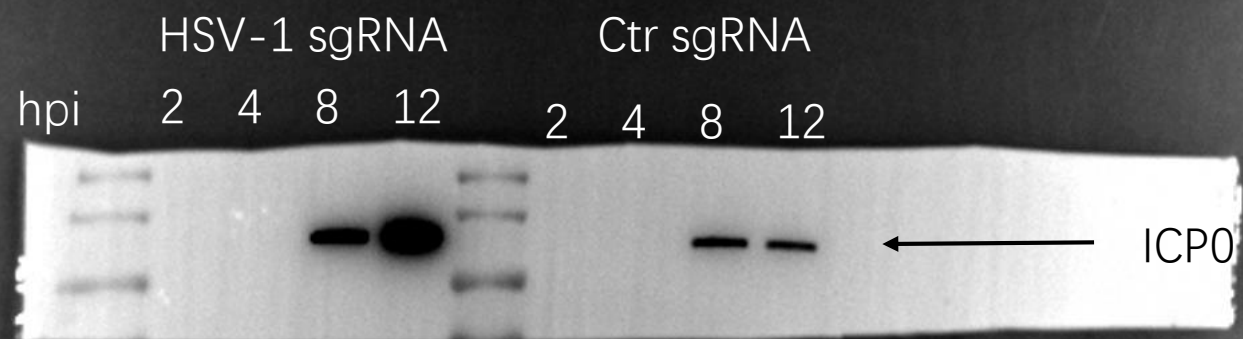

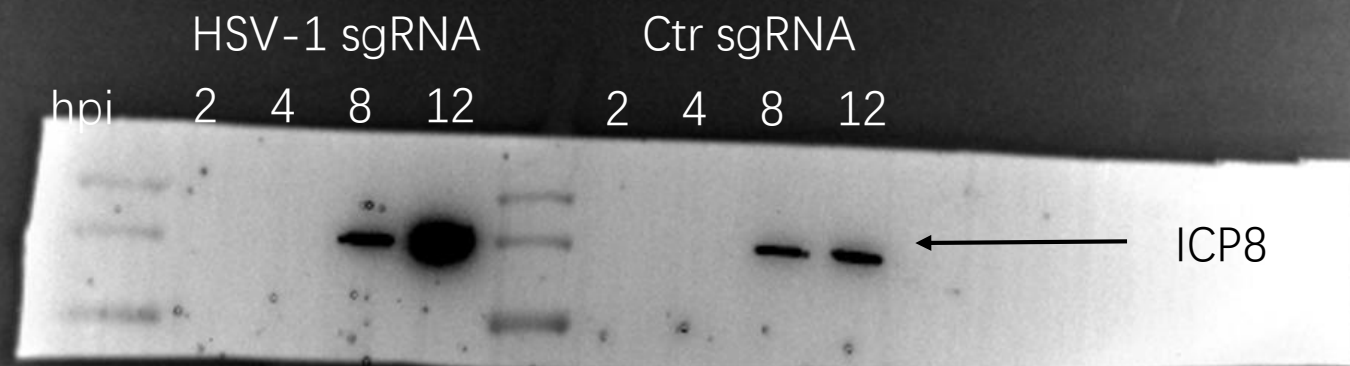

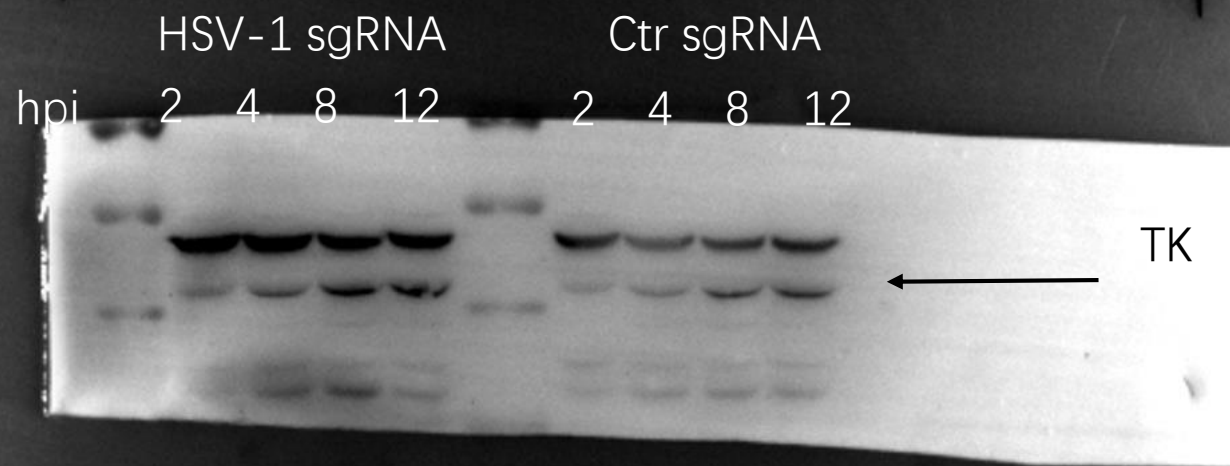

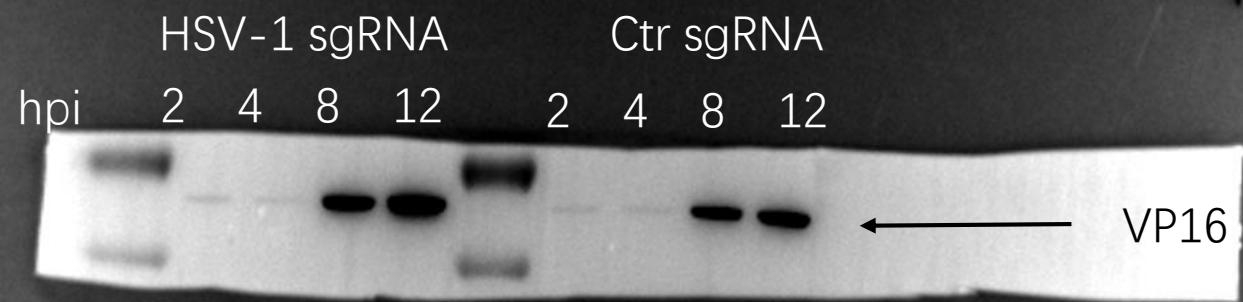

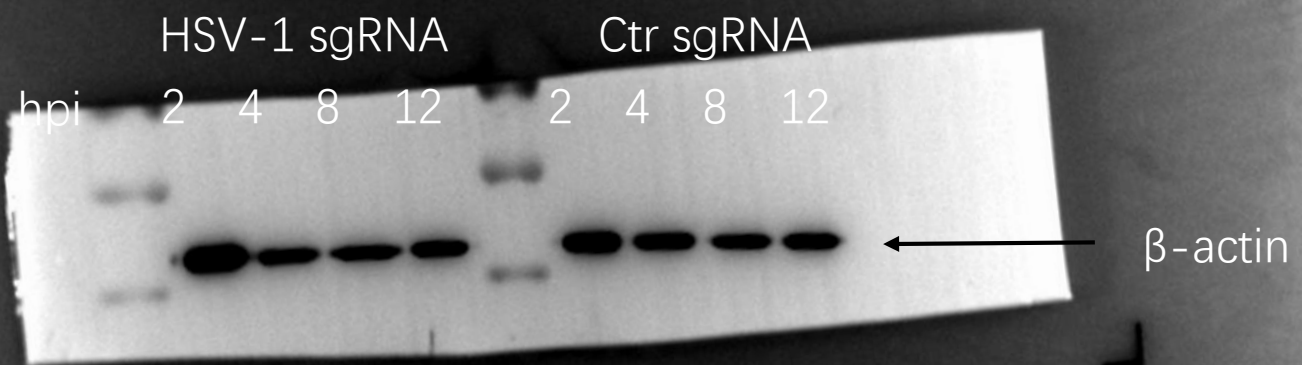

Supplement: Figure 4—source data 3. [file elife-85412-fig4-data3.pdf]

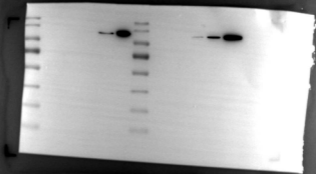

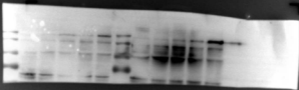

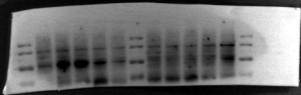

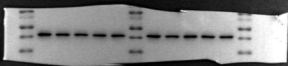

Supplement: Figure 4—source data 4. [file elife-85412-fig4-data4.pdf]

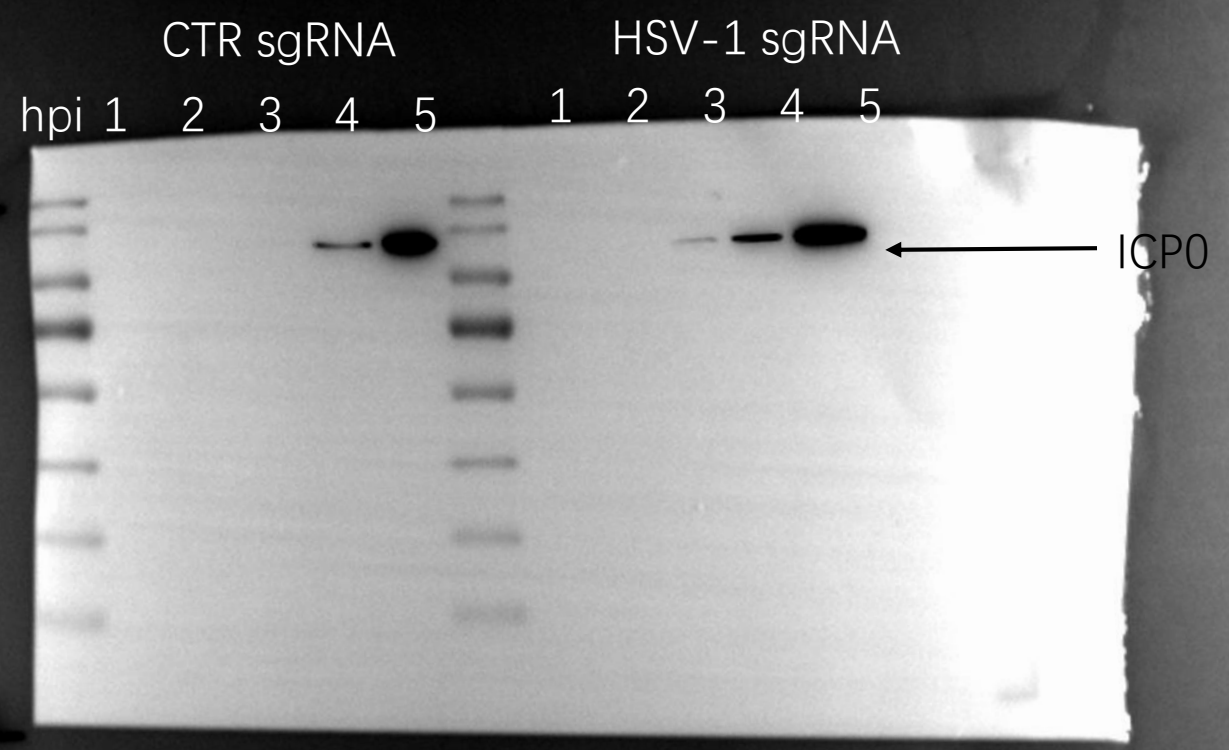

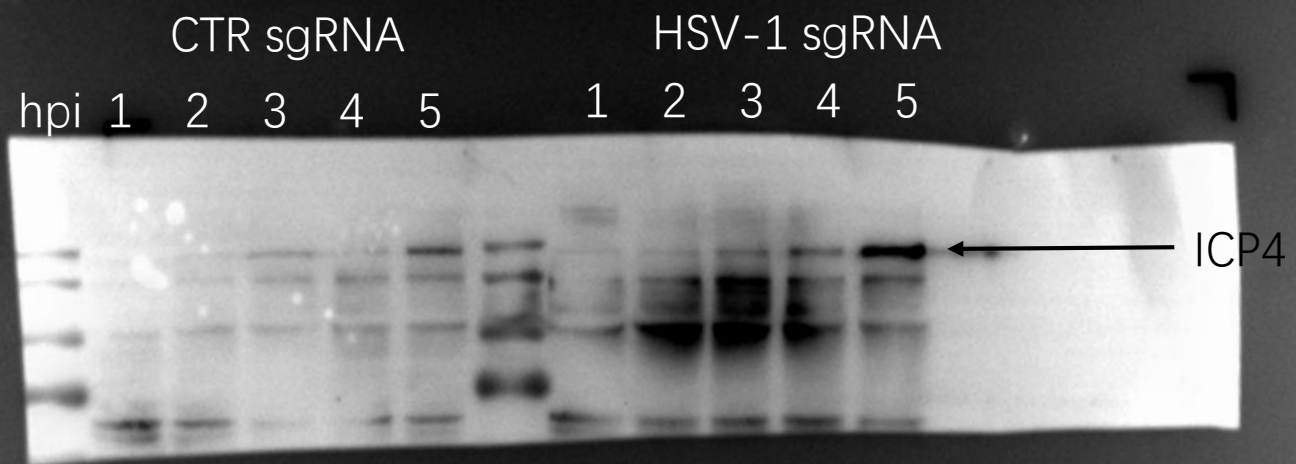

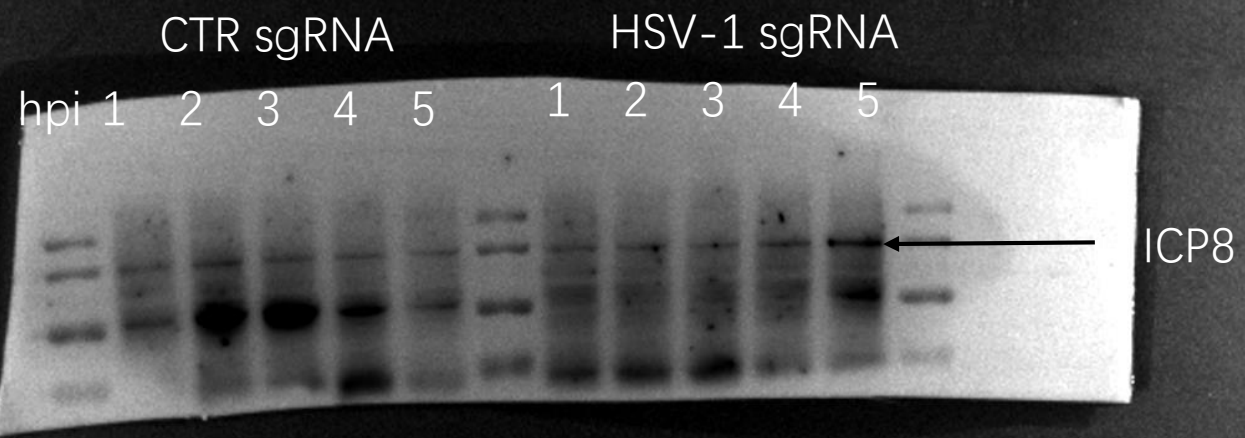

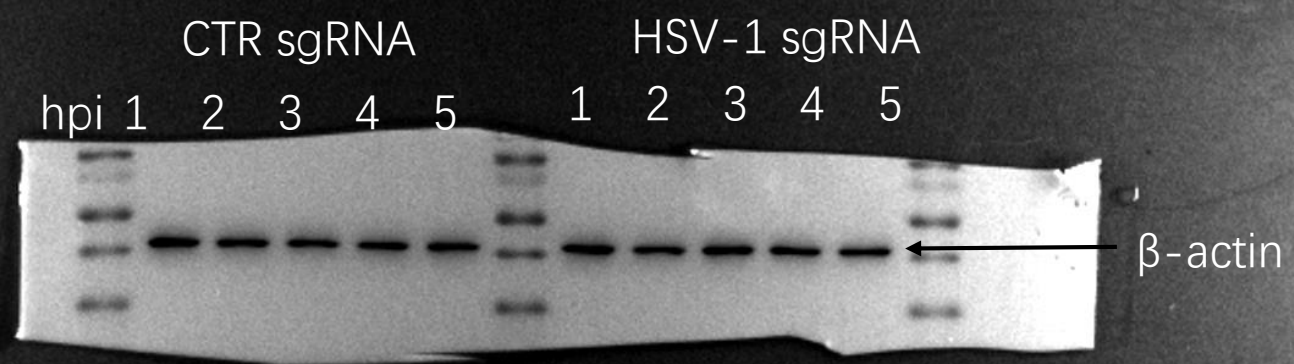

Supplement: Figure 4—source data 5. [file elife-85412-fig4-data5.pdf]

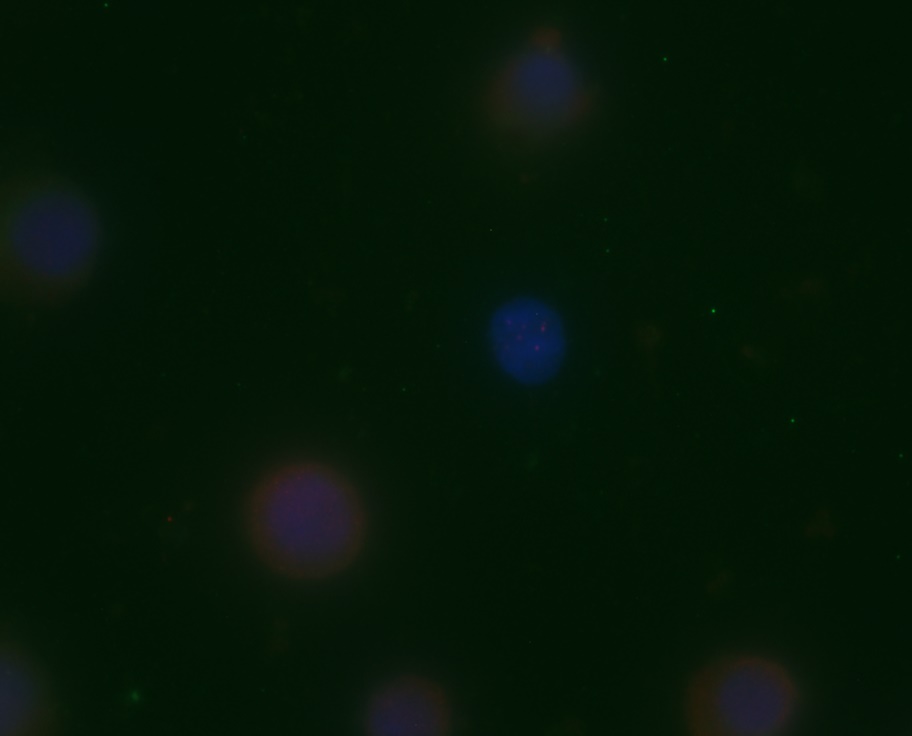

Supplement: Figure 4—source data 6. [file elife-85412-fig4-data6.zip › Figure 4 -source data 6 Fig 4 E/Fig 4 E control sgRNA_c1-3.jpg]

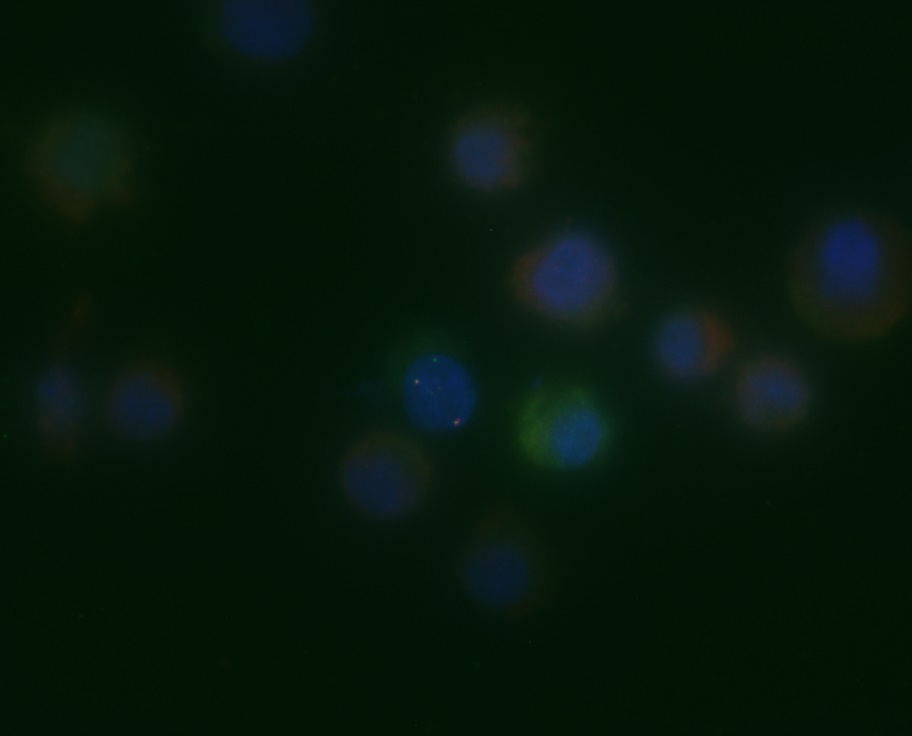

Supplement: Figure 4—source data 6. [file elife-85412-fig4-data6.zip › Figure 4 -source data 6 Fig 4 E/Fig 4 E HSV-1 sgRNA.jpg]

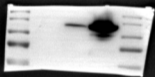

Supplement: Figure 4—source data 7. [file elife-85412-fig4-data7.pdf]

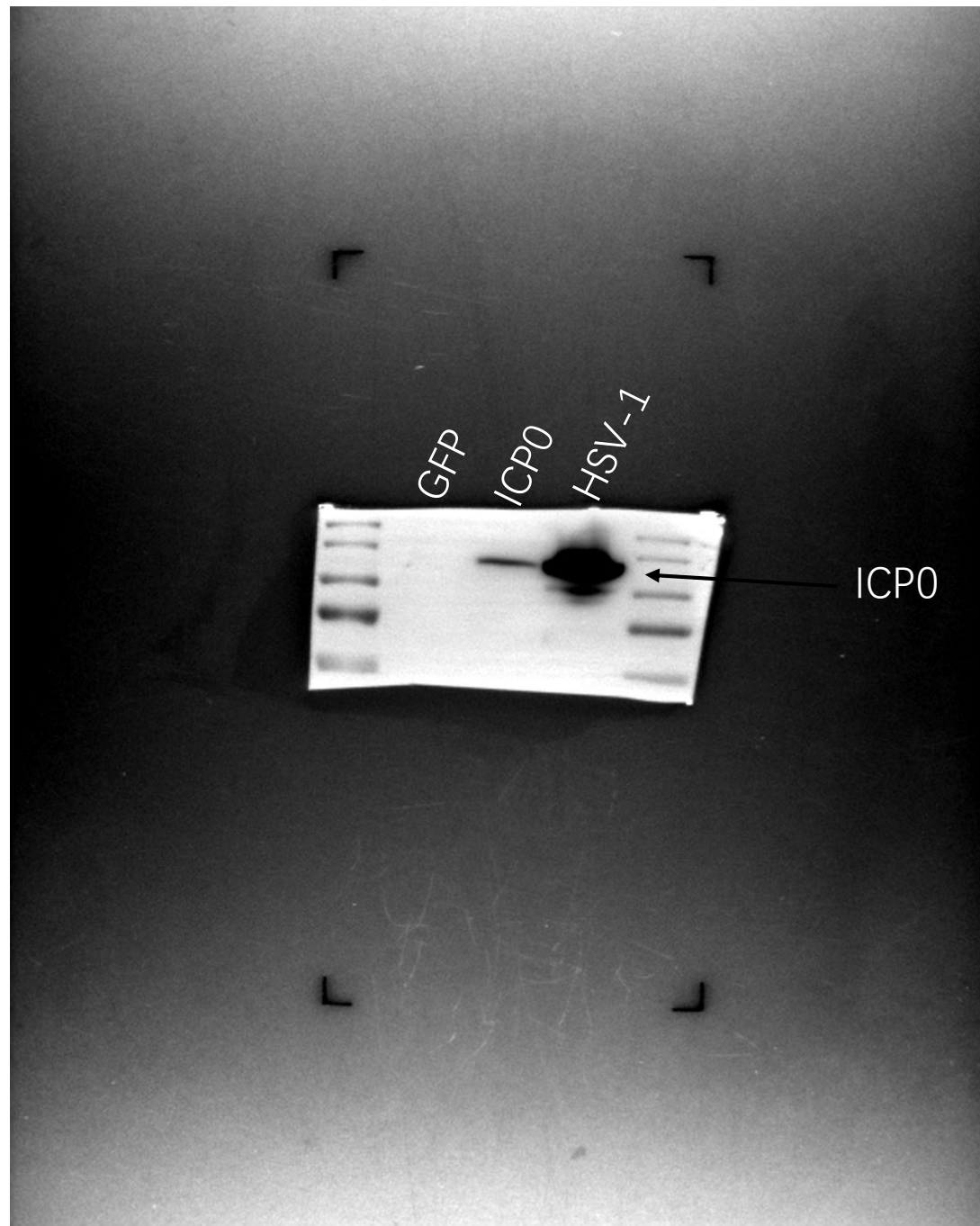

Supplement: Figure 4—source data 8. [file elife-85412-fig4-data8.pdf]

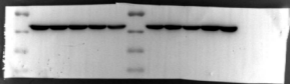

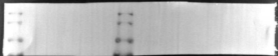

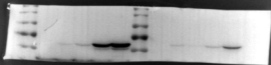

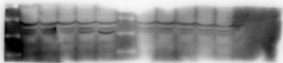

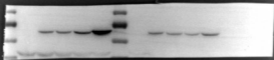

Supplement: Figure 4—figure supplement 1—source data 2. [file elife-85412-fig4-figsupp1-data2.pdf]

$\Delta$ ICP0 virus

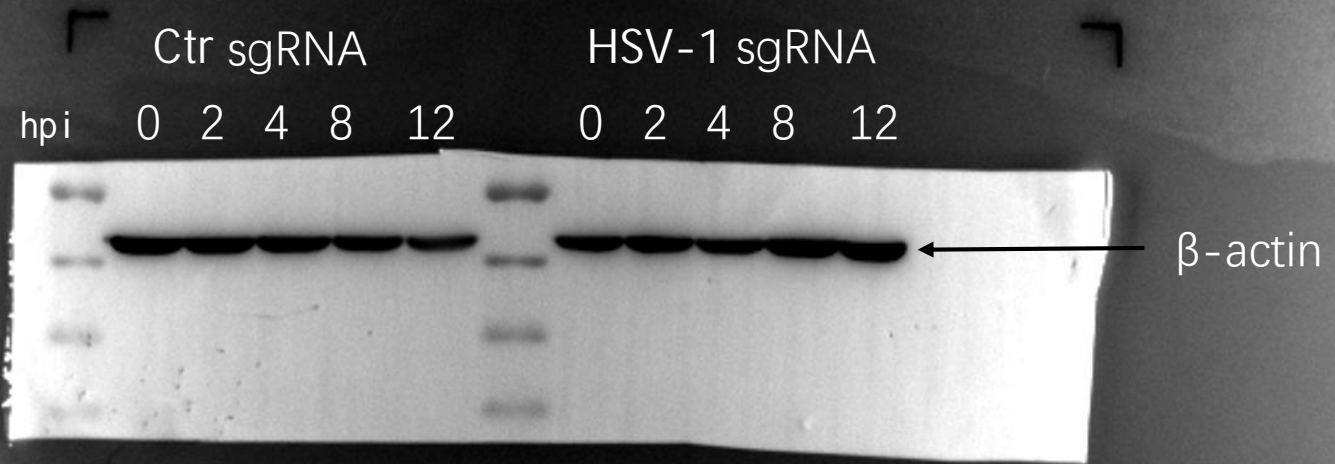

$\Delta$ ICP0 virus

Ctr sgRNA

HSV-1 sgRNA

hpi 0 2 4 8 12

0 2 4 8 12

ICP0

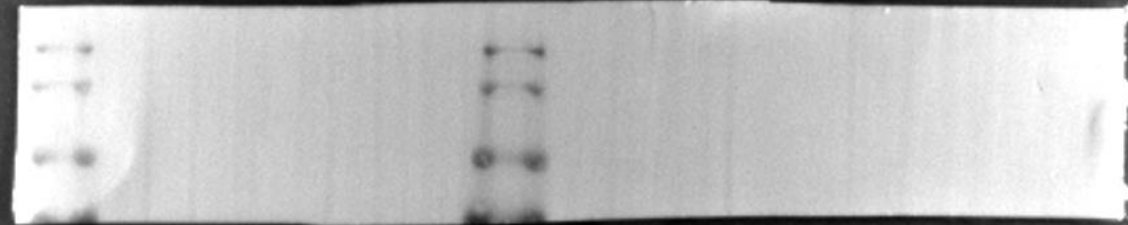

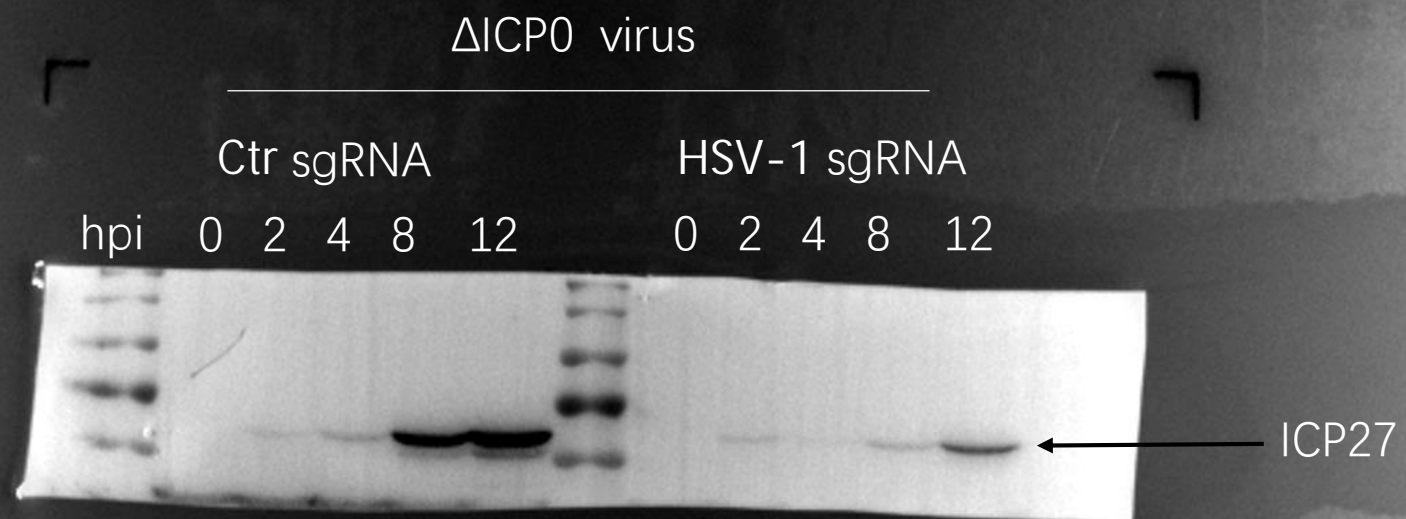

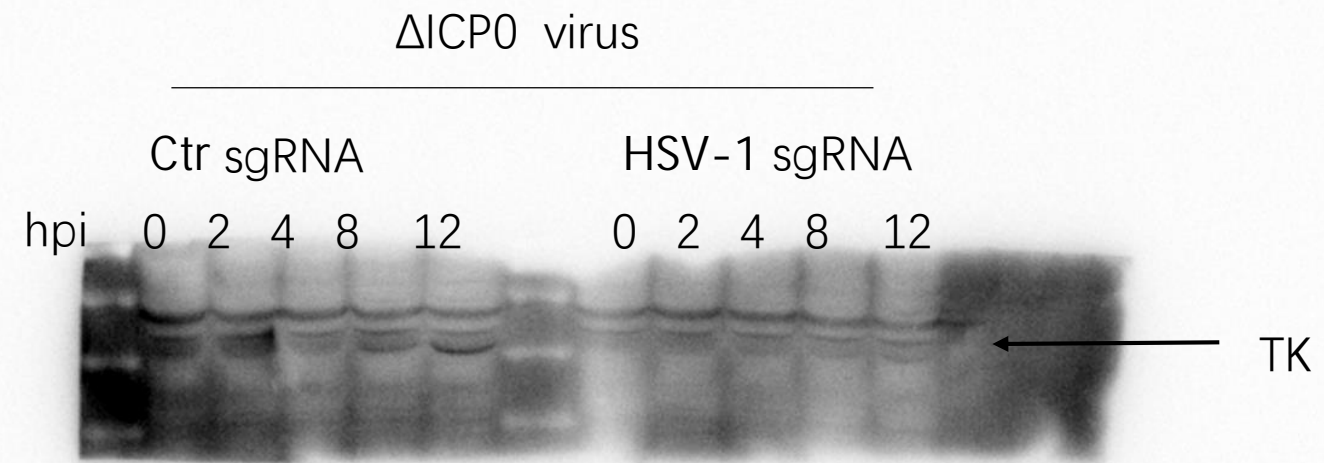

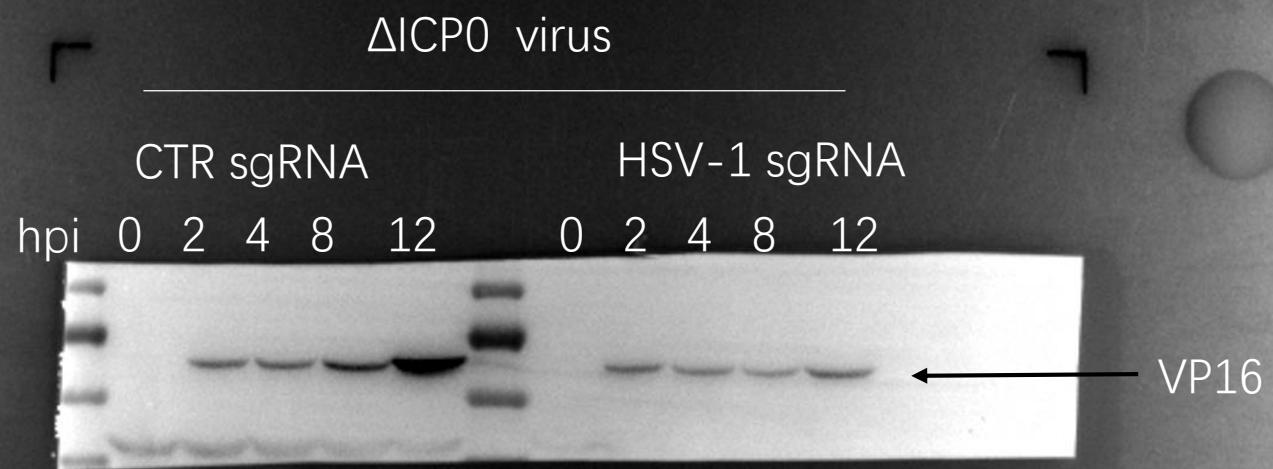

Supplement: Figure 4—figure supplement 1—source data 3. [file elife-85412-fig4-figsupp1-data3.pdf]
